# Taming the Achilles’ Heel: A Chemical and Structural Design to Address Off-Target Effects in siRNA Therapeutics

**DOI:** 10.1021/jacsau.5c01765

**Published:** 2026-02-09

**Authors:** Rohith Pavan Parvathaneni, Nithiyanandan Krishnan, Nikolai Hempel, Oommen P. Oommen, Oommen P. Varghese

**Affiliations:** † Translational Chemical Biology, Science for Life Laboratory, Department of Chemistry, Ångström Laboratory, Uppsala University, 751 21 Uppsala, Sweden; ‡ NATA MRC, Rutherford Appleton Laboratory, Harwell, Oxon OX11 0FA, U.K.; ∥ Department of Chemistry, Johannes Gutenberg University Mainz, Duesbergweg 10-14, 55128 Mainz, Germany; # School of Pharmacy and Pharmaceutical Sciences, 2112 Cardiff University, King Edward VII Avenue, Cardiff CF10 3NB, U.K.

**Keywords:** Chemical modification, Structural modification, RISC loading, Thermodynamic asymmetry, siRNA

## Abstract

Off-target effects represent one of the major bottlenecks for RNA interference (RNAi) technology. To address this issue, we present a novel strategy by combining seed-region chemical modification with an extended 3′-overhang on the sense strand (SS) to mitigate SS-mediated and miRNA-like nontargeted interactions. To modify the seed-region, we developed a novel 2′-diol modification that was selectively installed at different positions within the seed-region of siRNA. For this purpose, we synthesized universal 2′-diacetate phosphoramidites that yielded a free 2′-diol functionality after standard deprotection of oligonucleotides. The 2′-diol moieties with single (positions 3–7) and dual (6 + 7) insertions in the seed-region decreased the melting temperature (*T*_m_) by ca. −1 to −4.0 °C, imposing thermodynamic asymmetry. To improve the end-asymmetry of siRNA, we developed a structurally unsymmetrical siRNA (US-siRNA) design (five-nucleotides at the 3′-overhang region of SS), which together with seed-region modifications significantly increased the relative RISC loading of antisense strand (AS) with respect to their canonical sense variants. Overall, our rational design of chemical modifications of the seed-region with a 2′-diol moiety, in concert with the US-siRNA design, furnishes a simple, modular strategy to minimize off-target effects while maintaining the on-target RNAi activity.

## Graphical abstract

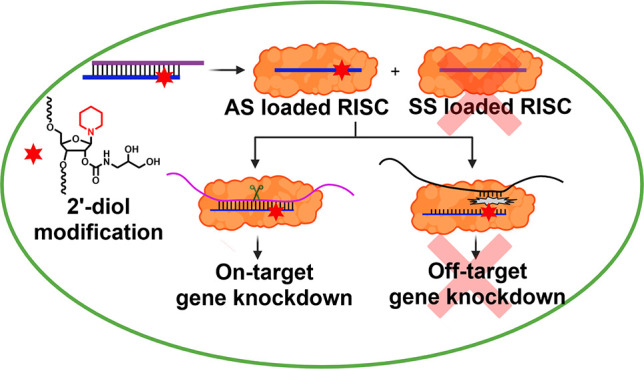

RNA interference (RNAi) employing small interfering RNA (siRNA) and microRNA (miRNA) has emerged as a promising technology for the treatment of a variety of diseases.^1^ Currently, there are seven siRNA-based drug formulations (extensively 2′-F and 2′-OMe modified siRNA) that are approved for clinical use to address rare genetic, metabolic, and hematologic disorders.^2^ Although these strategies are primarily designed to address the in vivo stability aspects rather than siRNA-based off-target effects, it should be noted that such modifications impart a sequence-dependent effect on RNAi potency.^3^ The siRNA structure consists of a double stranded sequence composed of sense (passenger) and antisense (guide) strand that undergoes a natural selection process by interacting with proteins such as DICER, TRBP, and Argonaute2 (Ago2), forming a RNA induced silencing complex (RISC) that is responsible for cleaving the target mRNA (mRNA) in a sequence-dependent manner.^4^ This results in post-transcriptional gene silencing with high efficiency and at very low concentrations due to the catalytic activity of the activated RISC.^5^ In the natural gene silencing event, the sense strand (SS) is degraded by Ago2 while the antisense strand (AS) is selectively recruited into RISC, which then binds to a cognate mRNA in a sequence-specific manner.^6^ The selection of the correct strand by the RISC is dependent on differences in thermodynamic asymmetry at the two ends of the sequence. The MID domain of Ago2 selectively binds to the thermodynamically less stable 5′-end of siRNA, thereby regulating this selection process.^7,8^

Today, there are advanced siRNA design algorithms^9–11^ that enable the selection of highly specific siRNA with natural thermodynamic asymmetry and minimal homology with other targets, thereby limiting nontargeted knockdown of gene transcripts. Nevertheless, the strand selection is not exclusive and SS is always recruited to some extent.^12^ Another concern of RNAi is miRNA-like off-target effects that require partial complementarity with the seed-region (2–8 nucleotides from the 5′-end) of the AS, which governs the target recognition and gene silencing.^13^

To improve the strand selection, several approaches have been pursued, including developing asymmetric siRNA,^14,15^ dicer substrate RNA,^16^ blocking phosphorylation at the 5′-end of the SS,^17^ fork siRNA,^18^ 4′-guanidinium-modified siRNA,^19^ and single-stranded RNA.^20^ We also recently reported that structural modifications of miRNA induce thermodynamic asymmetry to modulate selective strand recruitment (miR or miR*) into RISC by incorporating extended overhangs at the 3′-ends of either strands.^21^ To reduce miRNA-like off-target effects, several approaches were implemented that aimed to lower the melting temperature (*T*_m_) within the seed-region.^22^ Seed-region modifications included incorporating deoxy nucleotides,^23^ unlocked nucleic acid (UNA),^24^ glycol nucleic acid (*S*-GNA),^25^ spacer amidite,^26^ 2′-formamidino,^27^ 2′-deoxy-2′-α-F-2′-β-C-methyl pyrimidine,^28^ alkyl phosphonate,^29^ and amide internucleoside linkages.^30^ These modifications mitigated the off-target effects in a *T*_m_-dependent manner. Incorporation of 2′-*O*-methyl (2′-OMe)^31^ and locked nucleic acids (LNA)^32^ in the seed-region had positional effects with LNA modification showing detrimental impact on the RNAi activity. Thus, we envisaged the design of a modified siRNA that addresses both sense-mediated and miRNA-like off-target effects without compromising the RNAi activity. For this purpose, we developed the 2′-diol chemical modification that was coupled with a structurally modified unsymmetrical siRNA (US-siRNA) design with an extended SS overhang (Figure 1).

**Figure 1:**
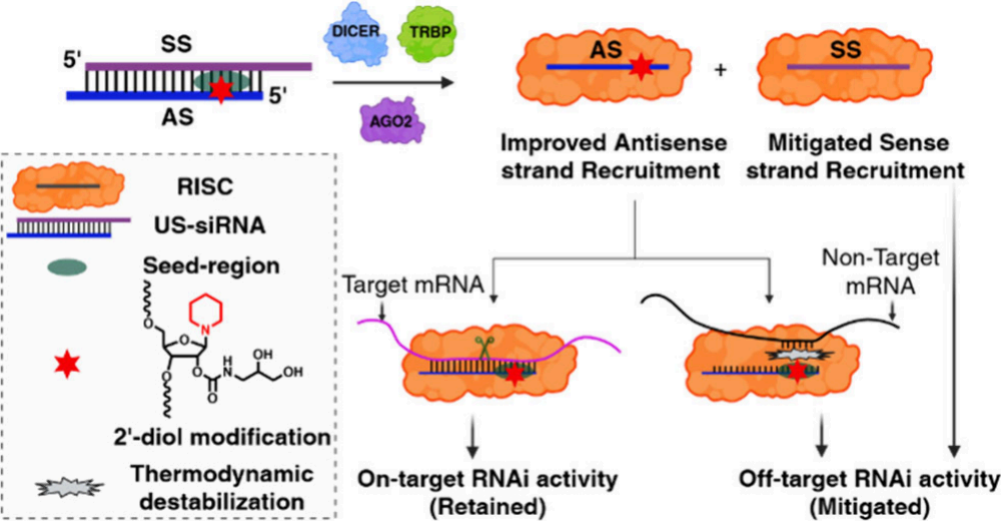
Schematic representation of the 2′-diol modified US-siRNA that mitigate the SS-mediated and miRNA-like off-target effects by improving the relative AS loading ratio and thermally destabilizing the seed-region.

To introduce 2′-diol chemical modifications on oligonucleotides, 2′-diacetate-modified phosphoramidites of adenine (A), uracil (U), guanine (G) and cytosine (C) were synthesized from their respective nucleosides **1** (**A, U, G** and **C**) with *N* ^6^-benzoyl (Bz), *N* ^2^-isobutyryl (iBu), and *N* ^4^-acetyl (Ac) protection on the A, G, and C nucleobases, respectively. Briefly, the 3′-5′ hydroxyls were protected by TIPS (1,1,3,3-tetraisopropyldisiloxane) to obtain nucleosides **2**. The free 2′-hydroxyl of **2** was activated with carbonyldiimidazole (CDI) to yield compounds **3**, which were directly treated with 1-aminoglycerol to obtain **4**. Free diols in compounds **4** were protected with an acetyl protecting group to obtain compounds **5**. The TIPS group was deprotected using triethylamine trihydrofluoride to generate the 5′- and 3′-dihydroxy compounds **6**. The 5′-hydroxyl groups of compounds **6** were exclusively protected using 4,4′-dimethoxytrityl chloride (DMT-Cl) to yield the DMT-protected nucleosides 7, which were subsequently treated with 2-cyanoethyl *N*,*N*-diisopropylchlorophosphoramidite (CEP-Cl) to generate phosphoramidites of the respective nucleobases (compounds **8**, Scheme 1). These phosphoramidite building blocks were purified, dried, and directly used to generate 2′-diol-modified oligonucleotides by standard solid-phase oligonucleotide synthesis. The incorporation of diacetate protecting groups on the 2′-diol moiety streamlines the solid-phase oligonucleotide synthesis, as the acetates are quantitatively removed under standard deprotection conditions to regenerate the free 2′-diol functionality in the fully deprotected oligonucleotide.

**Scheme 1:**
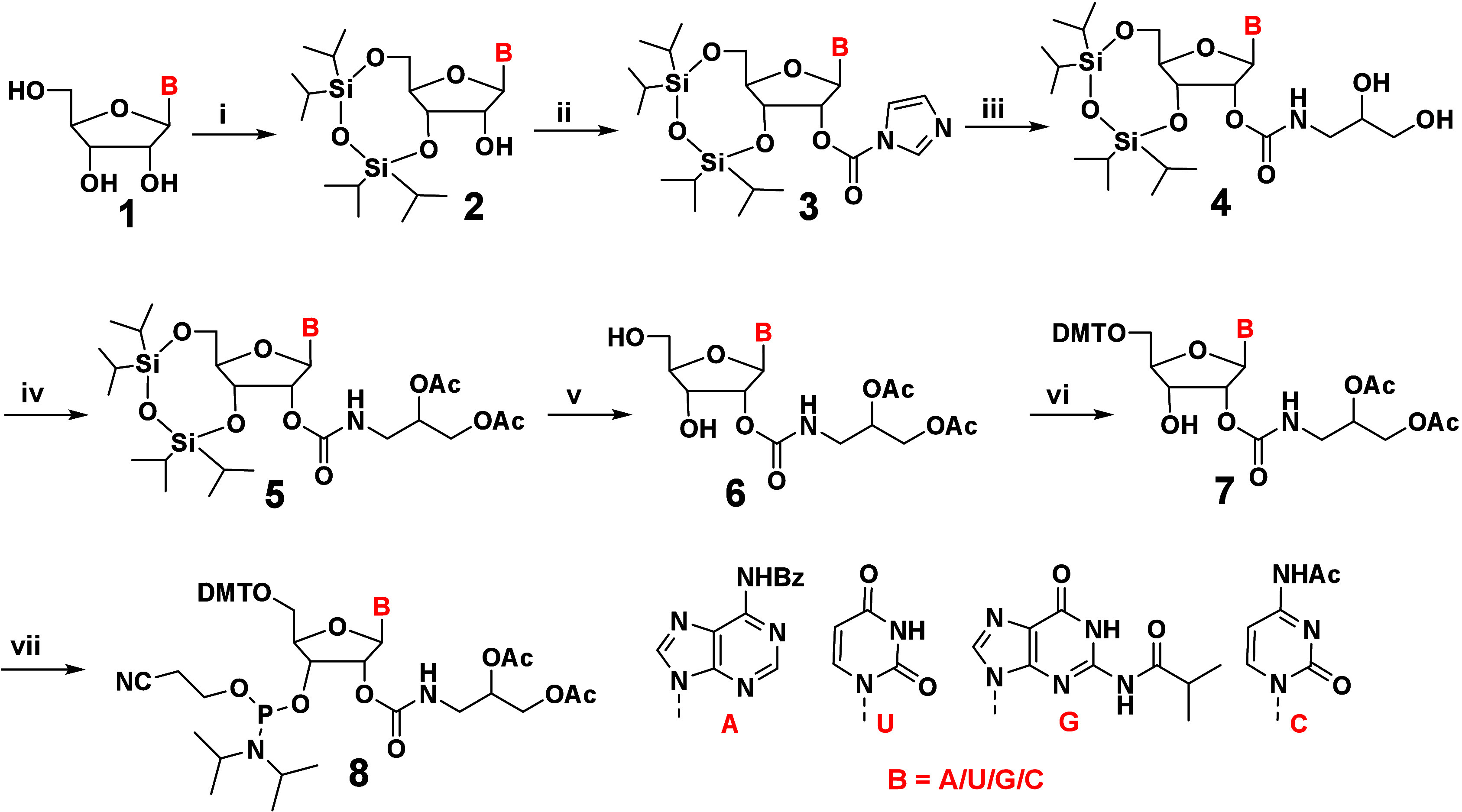
Synthesis of 2′-Diacetate Phosphoramidites^a^

a Reagents and conditions: (i) Cl-TIPS-Cl, dry pyridine (Py), 0 °C to room temperature (rt), 16 h; (ii) CDI, dry DCM, rt, 16 h; (iii) 1-aminoglycerol, Py, DIPEA, rt, 2 h; (iv) acetic anhydride, DMAP, Py, rt, 16 h; (v) triethylamine trihydrofluoride, Py, rt, 16 h; (vi) DMT-Cl, Py, rt, 16 h; (vii) DIPEA, CEP-Cl, DCM, rt, 3 h. B = different nucleobases (A, U, G and C).

As a model siRNA sequence, we chose a therapeutic siRNA against signal transducer and activator of transcription 3 (STAT3) mRNA, which is known for its oncogenic role in various cancer models.^33^ The SS of the *STAT3* siRNA was structurally modified with three additional deoxy-thymidine (dT) overhangs at the 3′-end to induce structural asymmetry, and the AS strands were chemically modified with a single 2′-diol insertion at positions 3, 4, 5, 6, 7 and dual substitutions at positions 6 and 7 to impart thermodynamic asymmetry and selective destabilization of the seed-region (Table 1). The various combinations of siRNA duplexes with SS overhang (SdT_2_ and SdT_5_) and AS seed-region (ASdT_2_, AS3, AS4, AS5, AS6, AS7, and AS67) variants were annealed together for subsequent biophysical and biochemical evaluations (Table S1).

**Table 1:**
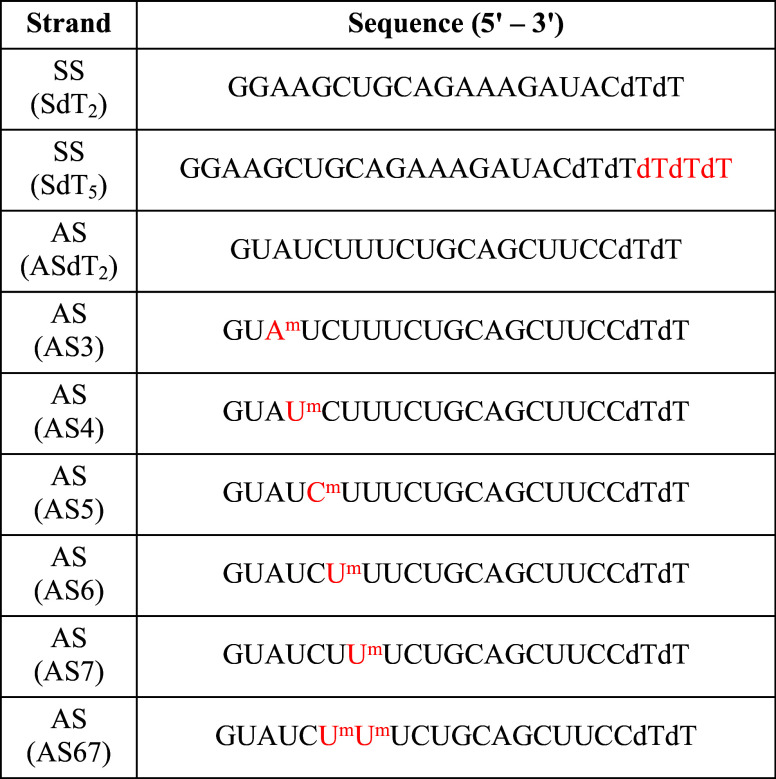
Structural and Chemical Design of Various SS and AS Sequences Designed against STAT3^a^

a A, U, G, and C indicate the RNA bases, whereas dT indicates the deoxy-thymidine nucleotide. A^m^, U^m^, and C^m^ indicate the 2′-diol modification of the respective nucleobases.

To determine the thermal asymmetry, we performed duplex melting studies for various combinations of chemically and structurally modified siRNA duplexes (Table 2). The incorporation of 2′-diol moieties within the AS seed-region progressively induces thermal destabilization, yielding a larger *T*_m_ drop as the modification is moved from position 3 toward position 7 (−1.2 to −2.5 °C) and culminating in a 4 °C drop when dual modifications at positions 6 and 7 are introduced. As anticipated, structurally modified US-siRNA with canonical AS (siR_US_) lowered the *T*_m_ by 0.6 °C, suggesting thermodynamic asymmetry as a result of extended overhang (Table 2).^34^ While the Δ*T*_m_ of −0.6 °C is modest for US-siRNA alone, the convergence of chemical and structural perturbations act synergistically, where the flexible extended overhang amplifies the thermodynamic destabilization introduced by the 2′-diols, thereby exhibiting a greater net *T*_m_ drop of −2.3 to −5.1 °C. Notably, the US-siRNA exhibits a pronounced *T*_m_ drop (up to −1 °C) with respect to their canonical sense variants (structurally unmodified SS), making them particularly interesting candidates for further evaluation of RNAi activity and relative RISC loading.

**Table 2:** Melting Temperatures (*T*_m_) of Chemically and Structurally Modified Duplexes^a^

siRNA	*T*_m_ (°C)	Δ*T*_m_ (°C)	siRNA	*T*_m_ (°C)	Δ*T*_m_ (°C)
**siR**	67.6 ± 0.1		**siR**_**US**_	67.0 ± 0.0	–0.6
**siR3**	66.4 ± 0.3	–1.2	**siR3**_**US**_	65.3 ± 0.1	–2.3
**siR4**	65.9 ± 0.0	–1.7	**siR4**_**US**_	65.2 ± 0.1	–2.4
**siR5**	66.0 ± 0.2	–1.6	**siR5**_**US**_	65.5 ± 0.0	–2.1
**siR6**	65.2 ± 0.1	–2.4	**siR6**_**US**_	64.3 ± 0.0	–3.3
**siR7**	65.1 ± 0.1	–2.5	**siR7**_**US**_	64.7 ± 0.2	–2.9
**siR67**	63.6 ± 0.1	–4.0	**siR67**_**US**_	62.5 ± 0.2	–5.1

a Thermal melting obtained by plotting the absorbance of the siRNA duplex (0.5 μM) at 260 nm against temperature in 10 mM phosphate buffer (pH 7.4) and 50 mM NaCl. *T*_m_ was calculated by determining the maximum of the first derivative of the melting curve. *T*_m_ values with standard deviations were reported as the average of 3 independent experiments. Δ*T*_m_ is the change in *T*_m_ with respect to canonical siRNA (siR). For duplex nomenclature, please see Table S1 in the Supporting Information.

To quantify the effect of chemical and structural modification on RNAi activity, we determined the STAT3 expression levels by quantitative PCR analysis in a human osteosarcoma cell line (MG63). We first quantified the effect of seed-region modification on the RNAi activity. For this purpose, we evaluated *STAT3* knockdown levels of different 2′-diol-modified siRNA sequences siR3, siR4, siR5, siR6, siR7, and siR67, having modifications at the 3, 4, 5, 6, 7, and 6/7 positions, respectively (Table S1). Interestingly, these modifications retained full gene silencing potency across three concentrations (50, 10, and 1 nM) identical to canonical siRNA (siR). However, at 0.1 nM all 2′-diol variants exhibited a position-dependent reduction in knockdown relative to siR (Figure 2A). Interestingly, this position-dependent reduction in knockdown was circumvented when structurally unsymmetrical design was incorporated within these sequences (siR_US_, siR3_US_, siR4_US_, siR5_US_, siR6_US_, siR7_US_, and siR67_US_). This indicates that although the seed-region destabilization can trim the RNAi potency at subnanomolar dose, the structural modification on the SS overhang restores the functional performance and is well tolerated by RNAi machinery (Figure 2B and Figure S32).

**Figure 2:**
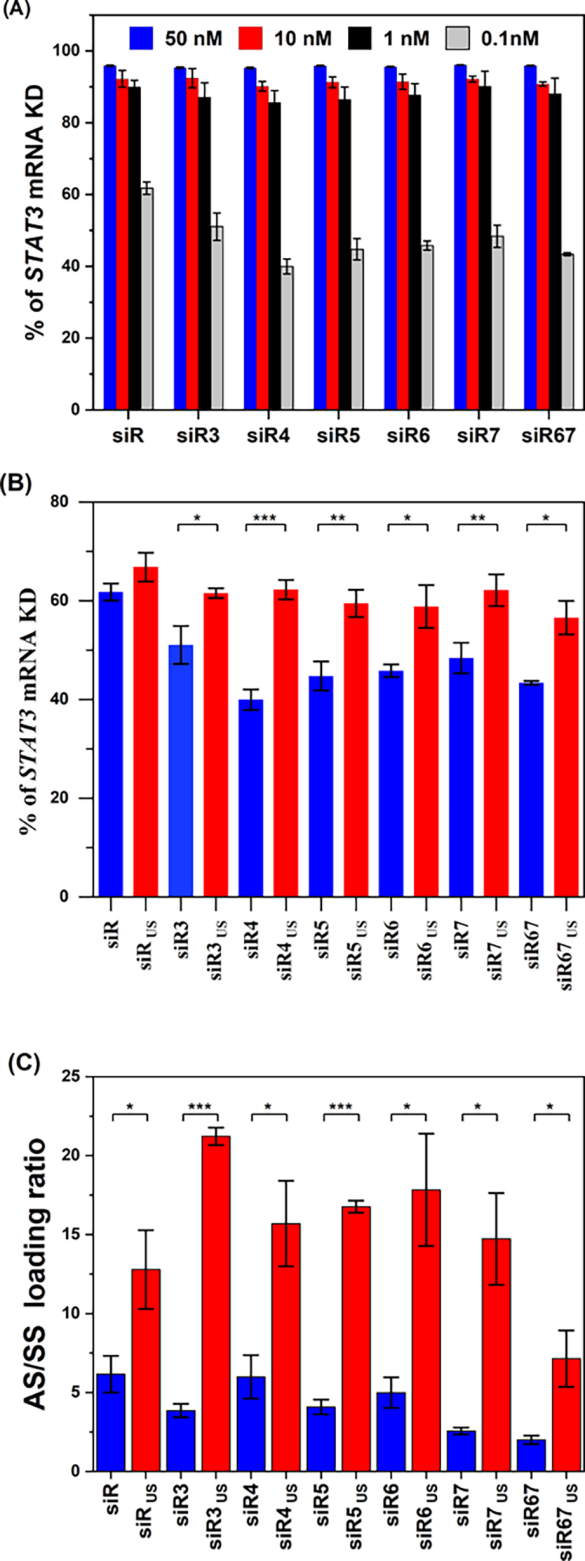
In vitro evaluation of chemically and structurally modified siRNA. (A) % of STAT3 mRNA knockdown (KD) at different concentrations (50, 10, 1, and 0.1 nM) of different 2′-diol AS-modified siRNA sequences (siR, siR3, siR4, siR5, siR6, siR7, and siR67). (B) % of STAT3 mRNA KD at 0.1 nM concentration of all siRNAs with both chemical and structural modifications. (C) AS to SS RISC loading ratio of all chemically and structurally modified siRNAs. All gene KD experiments were carried on MG63 cells for 24 h, and the observed values with standard deviation were reported as the average of 3–4 independent experiments. Statistics: Student’s unpaired Welch’s*t*-test with significance: *P < 0.05, **P < 0.01 and ***P < 0.001.

To probe the mechanism, we employed the stem-loop RT-qPCR assay to determine if our chemical and structural design has any influence in relative abundance of AS or SS post-transfection.^35^ The stem-loop experiment with both chemically and structurally modified siRNA revealed that the incorporation of the 3′-overhang itself resulted in an increase of in AS loading from 6.2 to 12.8 for siR and siR_US_. This suggests that our unsymmetrical design itself has an effect on selective AS loading. However, the insertion of 2′-diol in the seed region resulted in a modest reduction of relative AS/SS ratios to ∼4, ∼6, ∼4, ∼5, ∼2.5, and ∼2 for siR3, siR4, siR5, siR6, siR7, and siR67, respectively, despite their lower *T*_m_ values. This apparent discrepancy suggests that the overall reduction in *T*_m_ does not necessarily create the end-focused thermodynamic asymmetry, which is essential for the recognition by the MID-domain for selective strand selection. Interestingly, structurally modified US-siRNA sequences with 2′-diol insertions further augmented the AS/SS RISC loading ratios significantly with respect to their canonical sense variants by reaching ∼21 for siR3_US_, ∼15.5 for siR4_US_, ∼16.5 for siR5_US_, ∼17.5 for siR6_US_, ∼14.5 for siR7_US_, and ∼7 for siR67_US_ (Figure 2C). Although the relative abundance of a single strand in the cell lysate does not directly demonstrate the RISC loading of that strand, given the low enzymatic stability of unmodified single-stranded RNA, the observed increase implies stabilization of a specific strand by an intracellular mechanism, presumably RISC, imparting catalytic activity. The observed improvement of relative AS recruitment with US-siRNA reveals the preservation of the thermodynamic end-asymmetry that is augmented with 2′-diol substitutions, producing a synergistic enrichment of guide strand recruitment with improved RNAi activity. Such enrichment of guide strand recruitment can potentially minimize SS-mediated off-target effects, analogous to previous observation with UNA.^36^

Furthermore, to assess the capacity of 2′-diol modifications to discriminate against near-complementary off-targets, we introduced a single nucleotide bulge by omitting the uridine nucleoside at position 7 of the SS and annealed each chemically modified AS variant (Figure 3). Although the bulge lies distal to the diol insertions, all 2′-diol modified duplexes exhibited increased the *T*_m_ drop relative to the canonical siRNA, with dual substitutions producing the greatest effect with an exceptional *T*_m_ drop of −10.3 °C. This indicates that the 2′-diol modification amplifies the destabilizing effect even for sequences that has remote bulged regions (Table 3). Introducing a bulge together with an extended SS overhang better models partial miRNA-like pairing and amplified the mismatch discrimination, leading to even stronger thermal penalties for imperfect pairing (Table 3, *T*_m_ drops up to −13.7 °C). Such pronounced destabilization is expected to minimize undesired binding with nontargeted mRNA transcripts, thereby maintaining on-target knockdown and thermodynamically discriminating the off-target effects. While *T*_m_ measurements alone cannot predict the RISC-mediated targeting outcomes, these findings suggest that such chemical and structural modifications enhance the discrimination against imperfectly paired interactions, representing an intriguing avenue for future studies on off-target modulation.

**Figure 3:**
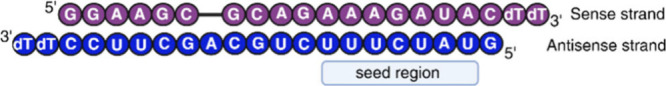
Schematic illustration of the bulged siRNA duplex with a 2-dT overhang.

**Table 3:** Thermal Melting Analysis of Bulged siRNA Duplexes with Chemical and Structural Modifications[Table-fn t3fn1]

AS modification	*T*_m_ (°C) Bulge SSdT_**2**_	Δ*T*_m_ (°C)	*T*_m_ (°C) Bulge SSdT_**5**_	Δ*T*_m_ (°C)
**ASdT2**	61.8 ± 0.2	–5.8	59.4 ± 0.1	–10.8
**AS3**	60.4 ± 0.2	–7.2	56.8 ± 0.0	–11.2
**AS4**	58.9 ± 0.2	–8.7	56.4 ± 0.2	–11.2
**AS5**	59.6 ± 0.4	–8.0	56.4 ± 0.5	–12.1
**AS6**	59.3 ± 0.3	–8.3	55.5 ± 0.1	–11.5
**AS7**	59.2 ± 0.2	–8.4	56.1 ± 0.1	–13.7
**AS67**	57.3 ± 0.1	–10.3	53.9 ± 0.2	–13.7

c Δ*T*_m_ is change in *T*_m_ with respect to canonical siRNA (siR).

In summary, we developed a rational siRNA design by combining chemical modifications in the seed region with a structural modification at the 3′-end of the SS. This strategy not only enhanced thermodynamic asymmetry but also improved the biophysical and biochemical properties of the siRNA. The 2′-diol substitutions thermodynamically destabilized the seed region and increased discrimination against imperfect duplex pairing, but when applied alone they lowered the relative RISC loading of the AS and reduced RNAi on-target activity. Remarkably, introducing an extended 3′-overhang on the SS restored end-focused thermodynamic asymmetry, thereby boosting AS loading while maintaining on-target activity. Together, these complementary modifications represent a promising design framework for future studies to evaluate their applicability across diverse siRNA sequences, enabling safer and more efficient gene silencing. Future work will focus on systematically exploring the sequence dependence of these modifications, elucidating their RISC-mediated off-target effects, and assessing their impact through transcriptome-wide assays. Such chemically and structurally engineered siRNAs could be readily integrated into current clinical modalities to achieve sustained, target-specific silencing with reduced side effects.

## Supplementary Material



## Notes

Materials and methods, experimental section, NMR analysis data, oligonucleotide mass analysis data, and melting curves (PDF)
