# Supplementary material for: Taming the Achilles’ Heel: A Chemical and Structural Design to Address Off-Target Effects in siRNA Therapeutics
Source: JACS Au. 2026 Feb 9;6(2):795–800. doi: 10.1021/jacsau.5c01765 (PMC12933297; doi:10.1021/jacsau.5c01765)
Supplement: Supplementary file 1 [file au5c01765_si_001.pdf]

SUPPORTING INFORMATION

Taming the Achilles' Heel: A Chemical and Structural Design to Address Off-Target Effects in siRNA Therapeutics

Rohith Pavan Parvathaneni[a], Nithiyanandan krishnan[b], Nikolai Hempel[c], Oommen P. Oommen[d] and Oommen P. Varghese[a]*

[a]Translational Chemical Biology, Science for Life Laboratory, Department of Chemistry, Ångström Laboratory, Uppsala University, 751 21, Uppsala, Sweden. E-mail: oommen.varghese@kemi.uu.se

[b]NATA MRC, Rutherford Appleton Laboratory, Harwell, Oxon, OX11 0FA, UK

[c]Department of Chemistry, Johannes Gutenberg University Mainz, Duesbergweg 10-14, 55128 Mainz, Germany

[d]School of Pharmacy and Pharmaceutical Sciences, Cardiff University, King Edward VII Avenue, Cardiff, CF10 3NB, UK

SUPPORTING INFORMATION

Table of Contents

General………………………………………………………………………………………. 3

Oligonucleotide synthesis and purification……………………………………….…………………... 3

Thermodynamic denaturing measurements…………………….……………….……………………. 4

Cell culture and siRNA transfection……………………………….…………………………………. 4

Real-Time qPCR………………………….……………………………………………….………….. 4

Stem-loop RT-qPCR……….……………….………………………………………….…………... 5

Table S1……….……………………………………………………………………………….... 6

Synthesis of 2'-diacetate phosphoramidites………………………………………………………....... 7

NMR characterization of the synthesized compounds………………………………………………… 40

Deconvoluted ESI-MS spectra and HPLC analysis of synthesized oligonucleotides…………………… 107

HRMS Analysis ….………………………………………………………….…………………… 114

In Vitro analysis ………………………….…………………………………………………………... 116

Thermal analysis - UV plots and Melting curves……………………………………………………... 117

General information:

All chemicals and reagents were purchased from Sigma-Aldrich. All solvents and dry solvents were purchased from Fisher-Scientific-Sweden. Universal UnyLinker Support 1000 Å, 2'-O-TBDMS protected Nucleoside phosphoramidites, Uridine, Adenosine (NBz), Cytidine (NAc), Guanosine (iBu), DMT deprotecting reagent (3% Trichloroacetic acid/DCM), Activation Reagent (0.3 M Benzyl thio tetrazole/Acetonitrile), CAP A (Acetic Anhydride/Pyridine/THF), CAP B (16% N-Methylimidazole in THF), Oxidation solution (0.02 M Iodine/Pyridine/Water/THF) were purchased from ChemGenes. MG63 cell line is obtained from ATCC. All reagents, media and primers for cell culture and cell experiments were purchased from Thermo Fisher Scientific. RNeasy plus mini kit for mRNA isolation is purchased from Qiagen. The concentration of oligonucleotides was measured at 260 nm using Cary 3500 Multicell UV-Vis Spectrophotometer. The mass analysis of the oligonucleotides was recorded on a Waters LC-MS system with Acquity UPLC BEH C18 column and an Acquity QDa detector in negative ion detection mode (ESI^-) by deconvoluting the *m/z* spectra using MassLynx software. The ^{1}H NMR (400 MHz), ^{13}C NMR (101 MHz), and ^{31}P NMR (162 MHz) were recorded on 400 MHz Jeol JNM-ECP Series FT NMR instrument. Multiplicities of ^{1}H NMR spin couplings are reported as s for singlet, bs for broad singlet, d for the doublet, t for the triplet, q for the quartet, dd for a doublet of the doublet, dt for a doublet of triplet, ddd for a doublet of doublet of doublets, and m for multiplet and overlapping spin systems. Values for apparent coupling constants (J) are reported in Hz. High-resolution mass spectra (HRMS) for the nucleoside intermediates were obtained in positive ion electrospray ionization (ESI) mode in UHPLC-Q-Exactive HF orbitrap ESI-MS (ThermoFinnigan).

Oligonucleotide synthesis, purification and analysis:

All oligonucleotides were synthesized using solid phase oligonucleotide synthesis by H8 K&A synthesizer with standard 2'-O-TBDMS protected monomers through phosphoramidite chemistry. Cleavage from solid support and deprotection of cyanoethyl and nucleotide protecting groups was carried out by adding 1 mL (1:1 V/V) of ammonium hydroxide and methylamine (AMA) solution to solid beads at

room temperature for 4 hrs. The AMA solution was evaporated using speedVac overnight to obtain dry 2'-O-TBDMS protected oligonucleotides. 2'-O-TBDMS was deprotected by treating with triethylamine trihydrofluoride and the reaction was quenched with isopropoxytrimethylsilane. Crude oligo is precipitated in cold ether and purified by 20% denaturing polyacrylamide gel electrophoresis (PAGE) (7 M Urea) and recovered with Tris-EDTA-NaCl (TEN) buffer. RNA samples were desalted using Waters (WAT020515) Sep-Pak column. The pure RNA pellet was resuspended in water and stored in RNAse free water at -20 °C. siRNA duplexes were formed by adding equimolar ratio of sense and antisense strand in RNAse free milli-Q water through heating at 90 °C for 10 min and gradually cooling down to room temperature.

Thermodynamic denaturing measurements:

0.5 μM siRNA duplex, 10 mM phosphate buffer and 50 mM NaCl were used to prepare the sample for the melting experiment. The melting temperatures (T_m) of siRNA were obtained by measuring the UV absorbance at 260 nm from 37 °C to 85 °C with a ramp rate of 0.5 °C per minute in the Cary 3500 Multicell UV-Vis spectrophotometer. T_m was calculated by plotting the change in absorbance with temperature in origin 2019 by utilizing the Boltzmann fitting and first order derivative function. All T_m values with standard deviation were reported as the average of three independent measurements. ΔT_m is the change in Tm of different duplexes with respect to the unmodified siRNA duplex (2-dT overhangs on sense strand (SS) and antisense strand (AS)).

Cell culture and siRNA transfection:

The MG63 cell line was cultured in 10% heat-inactivated fetal bovine serum (HI-FBS) with 1% AntiAnti (AA) in cell culture media (DMEM with high glucose). 35000 cells were seeded in a 24 well plate one day prior to the transfection (500 μL/well). The adhered cells were washed once with 350 μL PBS and 400 μL of fresh working media (10% HI-FBS in DMEM) is added to each well. The siRNAs were transfected with commercially available RNAimax (cat no: 13778150, Thermo Fisher Scientific, Sweden) using manufacturers protocol and incubated at 37 °C and 5% CO_2 for 24h before total RNA isolation.

Real-Time qPCR:

After 24 hrs of transfection, total RNA was isolated using Qiagen RNeasy plus mini kit using the manufactures protocol. The mRNA to cDNA reverse transcription reaction was performed using high-capacity RNA to cDNA kit (cat no: 4387406, Thermo Fisher Scientific, Sweden) in a 20 µl reaction using manufacturers protocol. Real Time-qPCR measurements was performed using TaqMan primers (STAT3 - Hs00374280_m1 and ACTB - Hs01060665_g1 from Thermo Fisher Scientific, Sweden) and TaqMan fast advanced master mix (cat no: 4444963, Thermo Fisher Scientific, Sweden) in a 20 µL reaction volume. Actin B gene was used as internal control for the analysis of qPCR data, where as non-treated well was used as experimental control. Amplification was carried out using the CFX connect system (Biorad) using a 40-cycle program. The raw Ct values were automatically calculated by the CFX connect software, which was used in calculating the relative mRNA levels using $2^{-\Delta\Delta CT}$ method, which is commonly used for analysing the relative gene expressions. The percentage of knockdown was calculated using the following formula $100 * (1-2^{-\Delta\Delta CT})$. Here $2^{-\Delta\Delta CT}$ is a fold change in levels of STAT3 mRNA.

Stem-loop RT-qPCR:

For the stem-loop qPCR, similar transfection protocols were followed for transfection and 10nM concentration of siRNAs were used to transfect. After transfection, small RNA was isolated using the Mirvana miRNA isolation kit (Thermo Fisher Scientific, Sweden) following the manufacturers protocol. Custom Taqman small RNA assay stem-loop primers for both sense strand and antisense strand were ordered from Thermo Fisher Scientific, Sweden (cat no: 4398987), with U6 snRNA as internal control. The qPCR experiment was performed similar as described in the above section and the relative AS/SS ratios were calculated using the previously mentioned ΔΔCT method.

Table S1. The duplex siRNA nomenclatures with SS and AS designs.

siRNA name	SS variant	AS Variant
siR	SdT_2	$ASdT_2$
siR3	SdT_2	AS3
siR4	SdT_2	AS4
siR5	SdT_2	AS5
siR6	SdT_2	AS6
siR7	SdT_2	AS7
siR67	SdT_2	AS67
siR_{US}	SdT_5	$ASdT_2$
$siR3_{US}$	SdT_5	AS3
$siR4_{US}$	SdT_5	AS4
$siR5_{US}$	SdT_5	AS5
$siR6_{US}$	SdT_5	AS6
$siR7_{US}$	SdT_5	AS7
$siR67_{US}$	SdT_5	AS67

For single strands nomenclature, please see **Table 1** from the main article.

Synthesis of 2'diacetate phosphoramidites:

Compound 2-U

(3',5'-O-(1,1,3,3-Tetraisopropyldisilox-1,3-diyl) uridine)

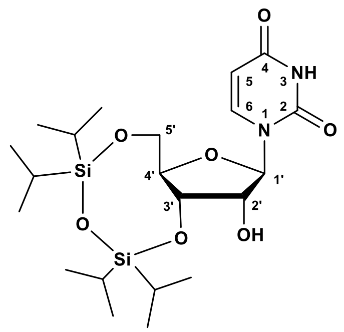

Uridine (**1-U**) (1254 mg, 5.14 mmol, 1.0 eq.) was dissolved in dry pyridine (27 mL) and 1,3-dichloro-1,1,3,3-tetraisopropyldisiloxane (1701 mg, 5.39 mmol, 1.05 eq.) was added dropwise to the solution over 40 min. The reaction mixture was stirred at room temperature for 2 h. At the end of the reaction, a colourless precipitate was formed in the yellow solution. The solvent was removed under reduced pressure and the residue was purified *via* column chromatography on silica (Hex:EA, 9:1 → 3:1) to yield **2-U** as a colourless foam (Yield: 1931.2 mg, 3.97 mmol, 77.2%).

MF: $C_{21}H_{38}N_2O_7Si_2$ **MW:** 486.71. R_F: 0.77 (SiO_2, EA).

LCMS (pos., ACN) (*m/z*): Calculated for $[C_{21}H_{38}N_2O_7Si_2H]^+$: 487.23, found: 487.20.

^{1}H-NMR (400 MHz, $CDCl_3$, 298 K, COSY): δ/ppm = 9.44 (s, 1H, H-3), 7.74 (d, J = 8.1 Hz, 1H, H-6), 5.74 (s, 1H, H-1'), 5.70 (dd, J = 8.1 Hz, J = 1.7 Hz, 1H, H-5), 4.33 (dd, J = 8.8 Hz, J = 4.8 Hz, 1H, H-3'), 4.23 – 4.17 (m, 2H, H-2', H-5'), 4.13 (dt, J = 8.9 Hz, J = 2.4 Hz, 1H, H-4'), 4.00 (dd, J = 13.2 Hz, J = 2.7 Hz, 1H, H-5'), 3.48 (s, 1H, *H*O-2'), 1.12 – 0.97 (m, 28H, TIPDS).

^{13}C-NMR (101 MHz, $CDCl_3$, 298 K, HSQC, HMBC, DEPT): δ/ppm = 163.62 (C-4), 150.27 (C-2), 140.09 (C-6), 102.09 (C-5), 91.02 (C-1'), 82.01 (C-4'), 75.28 (C-2'), 68.88 (C-3'), 60.26 (C-5'), 17.58 ($CH3^{TIPDS}$), 17.49 ($CH3^{TIPDS}$), 17.41 ($CH3^{TIPDS}$), 17.35 ($CH3^{TIPDS}$), 17.14 ($CH3^{TIPDS}$), 17.08 ($CH3^{TIPDS}$), 17.03 ($CH3^{TIPDS}$), 16.92 ($CH3^{TIPDS}$), 13.48 (CH^{TIPDS}), 13.06 (CH^{TIPDS}), 13.02 (CH^{TIPDS}), 12.56 (CH^{TIPDS}).

Compound 3-U

(2’-O-Carboimidazolyl-3',5'-O-(1,1,3,3-tetraisopropyldisilox-1,3-diyl) uridine)

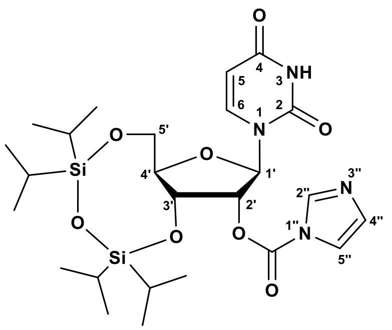

3',5'-O-(1,1,3,3-Tetraisopropyldisilox-1,3-diyl) uridine (**2-U**) (1987 mg, 4.08 mmol, 1.0 eq.) was dissolved in dry DCM (15 mL) and a solution of CDI (789 mg, 4.87 mmol, 1.2 eq.) in dry DCM (10 mL) was added. The reaction mixture was stirred at room temperature for 4 h. After completion of the reaction the reaction mixture was diluted with DCM and washed with water (2x40 mL) and a saturated solution of sodium bicarbonate (1x40 mL). The organic layer was dried over sodium sulfate and the solvent was removed under reduced pressure to yield **3-U** as a colourless foam (Yield: 2055 mg, 3.54 mmol, 86.7%).

MF: $C_{25}H_{40}N_4O_8Si_2$ **MW:** 580.79. R_F: 0.60 (SiO_2, EA).

LCMS (pos., ACN) (*m/z*): Calculated for $[C_{25}H_{40}N_4O_8Si_2H]^+$: 581.25, found: 581.20.

^{1}H-NMR (400 MHz, $CDCl_3$, 298 K, COSY): δ/ppm = 9.37 (s, 1H, H-3), 8.19 (d, J = 1.1 Hz, 1H, H-2’’), 7.71 (dd, J = 8.2 Hz, J = 0.8 Hz, 1H, H-6), 7.47 – 7.42 (m, 1H, H-5’’), 7.13 – 7.08 (m, 1H, H-4’’), 5.95 (s, 1H, H-1’), 5.75 (dd, J = 8.2 Hz, J = 1.8 Hz, 1H, H-5), 5.56 (d, J = 4.8 Hz, 1H, H-2’), 4.53 (dd, J = 9.1 Hz, J = 4.8 Hz, 1H, H-3’), 4.30 – 4.22 (m, 1H, H-5’), 4.09 – 3.98 (m, 2H, H-5’, H-4’), 1.10 – 0.83 (m, 28H, TIPDS).

^{13}C-NMR (101 MHz, $CDCl_3$, 298 K, HSQC, HMBC, DEPT): δ/ppm = 163.21 (C-4), 149.87 (C-2), 147.30 (C-$1^{Carbamate}$), 139.21 (C-6), 137.27 (C-2’’), 130.96 (C-4’’), 117.31 (C-5’’), 102.69 (C-5), 88.22 (C-1’), 82.48 (C-4’), 79.42 (C-2’), 68.07 (C-3’), 59.58 (C-5’), 17.50 (CH3^{TIPDS}), 17.45 (CH3^{TIPDS}), 17.34

($CH3^{TIPDS}$), 17.32 ($CH3^{TIPDS}$), 16.87 ($CH3^{TIPDS}$), 16.84 ($CH3^{TIPDS}$), 16.78 ($CH3^{TIPDS}$), 16.72 ($CH3^{TIPDS}$), 13.49 (CH^{TIPDS}), 12.96 (2x CH^{TIPDS}), 12.59 (CH^{TIPDS}).

Compound 4-U

(2’-O-((2’’,3’’-Dihydroxypropyl) carbamoyl)-3',5'-O-(1,1,3,3-tetraisopropyldisilox-1,3-diyl) uridine)

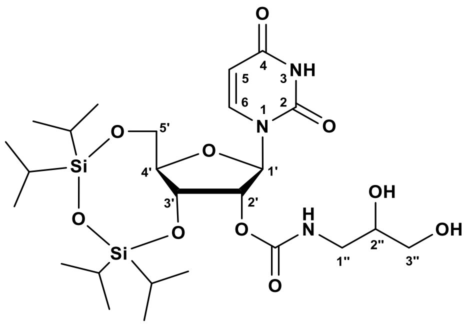

1-Aminoglycerol (1200 mg, 13.17 mmol, 2.6 eq.), a molecular sieve (4Å) and 2’-O-carboimidazolyl-3',5'-O-(1,1,3,3-tetraisopropyldisilox-1,3-diyl) uridine (**3-U**) (2980 mg, 5.13 mmol, 1.0 eq.) were dried in vacuo for two days before the reaction was started. 1-Aminoglycerol was dissolved in dry pyridine (14 mL), the molecular sieve (4Å) was added and it was stirred for 30 min at room temperature before DIPEA (1989 mg, 15.39 mmol, 3.0 eq.) was added. **3-U** was dissolved separately in dry pyridine (4 mL) and added to the reaction mixture. The reaction was completed after stirring for 2 h at room temperature and the solvent was removed under reduced pressure. The residue was diluted with DCM (150 mL) and washed with a saturated solutions of sodium bicarbonate (2x40 mL) and brine (40 mL). The aqueous layer was then extracted with DCM (25 mL) and the combined organic layer was dried over sodium sulfate and the solvent was removed under reduced pressure. The residue was purified *via* column chromatography on silica (DCM:MeOH, 49:1 → 15:1) to yield **4-U** as a colourless foam (Yield: 2641 mg, 4.37 mmol, 85.2%).

MF: $C_{25}H_{45}N_3O_{10}Si_2$ **MW:** 603.82. R_F: 0.16 (SiO_2, DCM:MeOH, 19:1).

LCMS (neg., ACN) (*m/z*): Calculated for $[C_{25}H_{44}N_3O_{10}Si_2]^-$: 602.25, found: 602.20.

^{1}H-NMR (400 MHz, $CDCl_3$, 298 K, COSY): δ/ppm = 7.71 (dd, J = 8.2 Hz, J = 3.2 Hz, 1H, H-6), 5.91 (t, J = 6.2 Hz, 1H, *H*N-1''), 5.82 (s, 1H, H-1'), 5.70 (d, J = 8.1 Hz, 1H, H-5), 5.26 (d, J = 5.1 Hz, 1H, H-2'), 4.33 – 4.27 (m, 1H, H-3'), 4.19 (d, J = 13.0 Hz, 1H, H-5'), 4.01 – 3.94 (m, 2H, H-4', H-5'), 3.90 – 3.76 (m, 1H, H-2''), 3.65 (dt, J = 10.3 Hz, J = 4.6 Hz, 1H, H-3''), 3.56 (ddd, J = 17.2 Hz, J = 11.6 Hz, J = 5.8 Hz, 1H, H-3''), 3.48 – 3.37 (m, 1H, H-1''), 3.31 – 3.09 (m, 1H, H-1''), 1.11 – 1.00 (m, 28H, TIPDS).

^{13}C-NMR (101 MHz, $CDCl_3$, 298 K, HSQC, HMBC, DEPT): δ/ppm = 163.70 (C-4), 155.88 (C=O^{Carbamate}), 150.50 (C-2), 139.18 (C-6), 102.53 (C-5), 88.68 (C-1'), 82.33 (C-4'), 76.24 (C-2'), 71.27 (C-2''), 67.76 (C-3'), 64.17 (C-3''), 59.68 (C-5'), 43.75 (C-1''), 17.57 (CH_3^{TIPDS}), 17.49 (CH_3^{TIPDS}), 17.41 (2xCH_3^{TIPDS}), 17.04 (2xCH_3^{TIPDS}), 16.95 (2xCH_3^{TIPDS}), 13.54 (CH^{TIPDS}), 13.02 (2xCH^{TIPDS}), 12.62 (CH^{TIPDS}).

Compound 5-U

(2'-O-((2'',3''-Diacetoxypropyl) carbamoyl)-3',5'-O-(1,1,3,3-tetraisopropyldisilox-1,3-diyl) uridine)

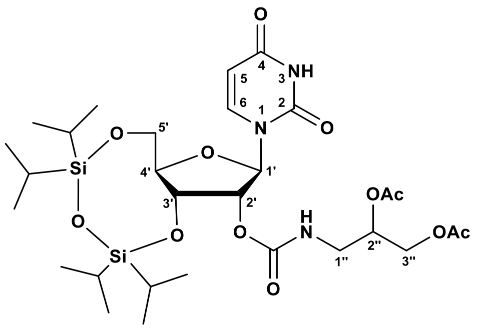

Compound **4-U** (2400 mg, 3.97 mmol, 1.0 eq.) was dissolved in dry pyridine (12 mL). DMAP (5.3 mg, 0.04 mmol, 0.01 eq.) and acetic anhydride (2638 mg, 25.84 mmol, 6.5 eq.) were added to the solution. After stirring the reaction mixture for 15 h, the solvent was removed under reduced pressure, the residue was diluted with DCM (150 mL) and washed with twice with solution of saturated sodium bicarbonate (2x40 mL). The organic layer was dried over sodium sulfate, the solvent was evaporated and the residue was dissolved in EA (75 mL). After washing with diluted hydrochloric acid (1N, 20 mL), the organic layer was dried over sodium sulfate and the solvent was removed under reduced pressure to obtain **5-U** as a colourless foam (Yield: 2611 mg, 3.80 mmol, 95.7%).

MF: $C_{29}H_{49}N_3O_{12}Si_2$ **MW:** 687.89. R_F: 0.37 (SiO_2, DCM:MeOH, 19:1).

LCMS (pos., ACN) (*m/z*): Calculated for $[C_{29}H_{49}N_3O_{12}Si_2H]^+$: 688.30, found: 688.25.

^{1}H-NMR (400 MHz, $CDCl_3$, 298 K, COSY): *δ*/ppm = 8.75 (s, 1H, H-3), 7.65 (dd, *J* = 8.1 Hz, *J* = 3.0 Hz, 1H, H-6), 5.79 (s, 1H, H-1'), 5.69 (dd, *J* = 8.0 Hz, *J* = 2.2 Hz, 1H, H-5), 5.26 (d, *J* = 5.1 Hz, 1H, H-2'), 5.20 (dt, *J* = 13.4 Hz, *J* = 5.8 Hz, 1H, *H*N-1''), 5.07 (p, *J* = 5.4 Hz, 1H, H-2''), 4.38 (t, *J* = 5.9 Hz, 1H, H-3'), 4.32 – 4.07 (m, 3H, H-5', H-3''), 4.02 – 3.94 (m, 2H, H-4', H-5'), 3.50 – 3.38 (m, 2H, H-1''), 2.11 – 2.06 (m, 6H, CH_3 (AcO-2''), CH_3 (AcO-3'')), 1.12 – 0.98 (m, 28H, TIPDS).

^{13}C-NMR (101 MHz, $CDCl_3$, 298 K, HSQC, HMBC, DEPT): *δ*/ppm = 170.86 (*C*=O (AcO-3'')), 170.46 (*C*=O (AcO-2'')), 163.09 (C-4), 154.88 (*C*=O^{Carbamate}), 149.71 (C-2), 139.77 (C-6), 102.32 (C-5), 89.07 (C-1'), 82.27 (C-4'), 76.18 (C-2'), 70.43 (C-2''), 68.04 (C-3'), 62.83 (C-3''), 59.87 (C-5'), 41.59 (C-1''), 21.08 (*C*H$_3$ (AcO-2'')), 20.86 (*C*H$_3$ (AcO-3'')), 17.57 (*C*H$_3^{TIPDS}$), 17.48 (*C*H$_3^{TIPDS}$), 17.40 (*C*H$_3^{TIPDS}$), 17.35 (*C*H$_3^{TIPDS}$), 17.04 (2x*C*H$_3^{TIPDS}$), 16.94 (2x*C*H$_3^{TIPDS}$), 13.52 (*C*H^{TIPDS}), 13.02 (2x*C*H^{TIPDS}), 12.65 (*C*H^{TIPDS}).

Compound 6-U

(2'-O-((2'',3''-Diacetoxypropyl) carbamoyl) uridine)

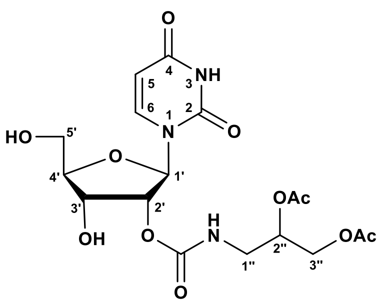

Compound **5-U** (2600 mg, 3.78 mmol, 1.0 eq.) was dissolved in dry pyridine (15 mL) and triethylamine trihydrofluoride (1828 mg, 11.34 mmol, 3.0 eq.) was added to the solution. After stirring for 16 h, the solvent was removed under reduced pressure and the residue was purified *via* column chromatography on silica (DCM:MeOH, 32:1 → 15:1) to yield **6-U** as a colourless foam (Yield: 1254 mg, 2.82 mmol, 74.6%).

MF: $C_{17}H_{23}N_3O_{11}$ **MW:** 445.38. *R*F: 0.15 (SiO_2, DCM:MeOH, 15:1).

LCMS (pos., ACN) (*m/z*): Calculated for $[C_{17}H_{23}N_3O_{11}H]^+$: 446.14, found: 446.10.

^{1}H-NMR (400 MHz, ACN-*d*3, 298 K, COSY): *δ*/ppm = 9.14 (s, 1H, H-3), 7.84 – 7.74 (m, 1H, H-6), 5.93 (m, 2H, H-1', *H*N-1''), 5.66 – 5.60 (m, 1H, H-5), 5.12 – 5.01 (m, 2H, H-2', H-2''), 4.30 (q, *J* = 3.8 Hz, 1H, H-3'), 4.21 – 4.02 (m, 2H, H-3''), 3.97 (t, *J* = 4.1 Hz, 1H, H-4'), 3.76 (dd, *J* = 11.7 Hz, *J* = 2.0 Hz, 1H, H-5'), 3.68 (dd, *J* = 11.9 Hz, *J* = 3.0 Hz, 1H, H-5'), 3.39 – 3.21 (m, 2H, H-1''), 2.03 – 1.97 (m, 6H, C*H*3 (AcO-2''), C*H*3 (AcO-3'')).

^{13}C-NMR (101 MHz, $CDCl_3$, 298 K, HSQC, HMBC, DEPT): *δ*/ppm = 171.59 (*C*=O (AcO-3'')), 171.42 (*C*=O (AcO-2'')), 163.92 (C-4), 156.36 (*C*=O^{Carbamate}), 151.55 (C-2), 141.63 (C-6), 102.98 (C-5), 87.61 (C-1'), 86.17 (C-4'), 76.57 (C-2'), 71.02 (C-2''), 70.57 (C-3'), 63.60 (C-3''), 61.98 (C-5'), 41.64 (C-1''), 21.16 (*C*H3 (AcO-2'')), 20.84 (*C*H3 (AcO-3'')).

Compound 7-U

(2'-O-((2'',3''-Diacetoxypropyl) carbamoyl)-5'-O-DMT-uridine)

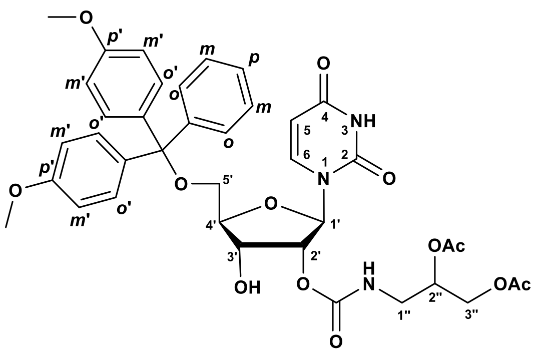

Compound **6-U** (1214 mg, 2.73 mmol, 1.0 eq.) was co-evaporated twice with dry pyridine (3 mL) before it was dissolved in dry pyridine (25 mL). 4,4'-Dimethoxytritylchloride (1110 mg, 3.28 mmol, 1.2 eq.) was added in two portions and the reaction mixture was stirred for 16 h at room temperature under argon atmosphere. The solvent was removed under reduced pressure and the residue was purified via column

chromatography on silica (Hex:EA, 3:1 → 0:1, with 1% Et_3N) to yield **7-U** as a colourless foam (Yield: 1976 mg, 2.64 mmol, 96.7%).

MF: $C_{38}H_{41}N_3O_{13}$ **MW:** 747.75. R_F: 0.39 (SiO_2, DCM:MeOH, 15:1).

LCMS (neg., ACN) (*m/z*): Calculated for $[C_{38}H_{40}N_3O_{13}]^-$: 746.25, found: 746.20.

HRMS (ESI-MS, pos. mode): calculated for $[C_{38}H_{41}N_3O_{13}H]^+$: 748.2712, found: 748.2692

^{1}H-NMR (400 MHz, DMSO-d_6, 298 K, COSY): δ/ppm = 11.39 (d, J = 12.1 Hz, 1H, H-3), 7.73 – 7.65 (m, 1H, H-6), 7.59 (t, J = 8.1 Hz, 1H, *H*N-1''), 7.41 – 7.18 (m, 9H, 4xH-*o*', 2xH-*o*, 2xH-*m*, H-*p*), 6.92 – 6.84 (m, 4H, 4xH-*m*'), 5.90 (d, J = 10.4 Hz, 1H, H-1'), 5.55 (dt, J = 13.7 Hz, J = 5.7 Hz, 1H, *H*O-3'), 5.41 – 5.32 (m, 1H, H-5), 5.15 (t, J = 7.8 Hz, 1H, H-2'), 4.99 – 4.90 (m, 1H, H-2''), 4.33 (dt, J = 11.2 Hz, J = 5.8 Hz, 1H, H-3'), 4.21 – 4.11 (m, 1H, H-3''), 4.07 – 3.93 (m, 2H, H-4', H-3''), 3.74 (s, 3H, CH_3O-*p*'), 3.70 (s, 3H, CH_3O-*p*'), 3.35 – 3.14 (m, 4H, 2xH-5', 2xH-1''), 2.03 – 1.92 (m, 6H, (CH_3 (AcO-2''), CH_3 (AcO-3'')).

^{13}C-NMR (101 MHz, DMSO-d_6, 298 K, HSQC, HMBC, DEPT): δ/ppm = 170.21 (*C*=O (AcO-3'')), 169.93 (*C*=O (AcO-2'')), 162.98 (C-4), 158.17 (2xC-*p*'), 155.50 (C=$O^{Carbamate}$), 150.38 (C-2), 144.64 (C-*ipso*), 140.84 (C-6), 135.36 (C-*ipso*'), 135.12 (C-*ipso*'), 129.82 (4xC-*o*'), 127.94 (2xC-*o*), 127.75 (2xC-*m*), 126.82 (C-*p*), 113.29 (4xC-*m*'), 101.92 (C-5), 86.73 (C-1'), 85.94 (C_q^{DMT}), 83.23 (C-4'), 74.75 (C-2'), 70.15 (C-2''), 68.57 (C-3'), 63.16 (C-5'), 62.75 (C-3''), 55.07 (2x*C*H_3O-*p*'), 40.29 (C-1''), 20.80 (*C*H_3 (AcO-3'')), 20.59 (*C*H_3 (AcO-2'')).

Compound 8-U

(2'-O-((2,3-Diacetoxypropyl) carbamoyl)-5'-O-DMT-3'-O-((2-cyanoethoxy, diisopropyl-amino, phosphanyl) uridine)

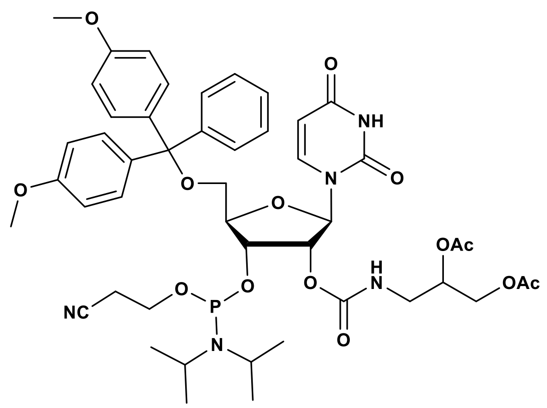

Compound **7-U** (500 mg, 0.65 mmol, 1.0 eq.) was co-evaporated together with dry pyridine (15mL) to remove the traces of water thrice. Under argon atmosphere, **7-U** was dissolved in dry DCM (15 mL), DIPEA (207.6 mg, 2 mmol, 3.0 eq.) was added and 2-Cyanoethyl *N*,*N*-diisopropylchlorophosphoramidite (310 mg, 1.3 mmol, 2 eq.) was added dropwise over 5 min and reaction mixture was stirred for 3 h at room temperature under argon atmosphere. After removal of the solvent, the residue was purified via column chromatography on silica (Hex:EA, 1:1 → 0:1, with 1% Et_3N) under argon atmosphere. The slightly yellowish foam obtained in this way was dissolved in dry DCM (3 mL) and precipitated with cold hexane (40 mL) while stirring. This procedure was repeated three times and the product was collected by centrifugation and drying in vacuo to yield a colourless foam (Yield: 380 mg, 0.4 mmol, 61.7%).

MF: $C_{47}H_{58}N_5O_{14}P$ **MW:** 947.98.

^{31}P-NMR (162 MHz, $CDCl_3$, 298 K): δ/ppm = 151.12, 150.9, 150.7, 150.5 and 150.4.

Compound 2-C

(3',5'-O-(1,1,3,3-Tetraisopropyldisilox-1,3-diyl) cytidine (NAc))

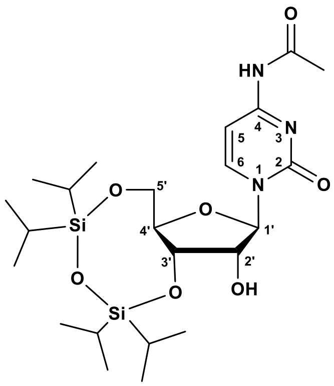

N-acetyl-Cytidine (**1-C**) (1352 mg, 4.74 mmol, 1.0 eq.) was dissolved in dry pyridine (24 mL) and 1,3-dichloro-1,1,3,3-tetraisopropyldisiloxane (1735 mg, 5.50 mmol, 1.16 eq.) was added dropwise to the solution over 60 min while it was cooled on ice. The reaction mixture was stirred at room temperature for 15 h, another portion of 1,3-dichloro-1,1,3,3-tetraisopropyldisiloxane (149 mg, 0.474 mmol, 0.1 eq.) was added and the mixture was stirred for another 60 min. The solvent was removed under reduced pressure and the residue was purified *via* column chromatography on silica (Hex:EA, 2:1 → 0:1) to yield **2-C** as a colourless foam (Yield: 2154 mg, 4.08 mmol, 86.1%).

MF: $C_{23}H_{41}N_3O_7Si_2$ **MW:** 527.77. R_F: 0.40 (SiO_2, EA).

LCMS (pos., ACN) (*m/z*): Calculated for $[C_{23}H_{41}N_3O_7Si_2H]^+$: 528.26, found: 528.20.

^{1}H-NMR (400 MHz, $CDCl_3$, 298 K, COSY): δ/ppm = 10.09 (s, 1H, *H*N-4), 8.19 (dd, 1H, J = 7.5 Hz, J = 0.9 Hz, H-6), 7.46 – 7.40 (m, 1H, H-5), 5.81 (t, J = 18.6 Hz, 1H, H-1'), 4.29 – 4.17 (m, 4H, H-2', H-3', H-4', H-5'), 4.00 (dd, J = 13.4 Hz, J = 2.6 Hz, 1H, H-5'), 3.09 (s, 1H, *H*O-2'), 2.29 (t, J = 17.7 Hz, 3H, CH_3 (AcNH-4)), 1.14 – 0.90 (m, 28H, TIPDS).

^{13}C-NMR (101 MHz, $CDCl_3$, 298 K, HSQC, HMBC, DEPT): δ/ppm = 171.19 (*C*=O (AcNH-4)), 163.18 (C-4), 154.95 (C-2), 144.49 (C-6), 96.63 (C-5), 91.54 (C-1'), 82.09 (C-4'), 75.31 (C-2'), 68.60 (C-3'), 60.05 (C-5'), 25.06 (*C*H_3 (AcNH-4)), 17.54 (*C*H_3^{TIPDS}), 17.47 (*C*H_3^{TIPDS}), 17.38 (*C*H3^{TIPDS}), 17.35 (*C*H_3^{TIPDS}), 17.06 (*C*H_3^{TIPDS}), 17.00 (*C*H_3^{TIPDS}), 16.96 (*C*H_3^{TIPDS}), 16.89 (*C*H_3^{TIPDS}), 13.42 (*C*H^{TIPDS}), 13.02 (*C*H^{TIPDS}), 12.99 (*C*H^{TIPDS}), 12.53 (*C*H^{TIPDS}).

Compound 3-C

(2’-O-Carboimidazolyl-3',5'-O-(1,1,3,3-tetraisopropyldisilox-1,3-diyl) cytidine (NAc))

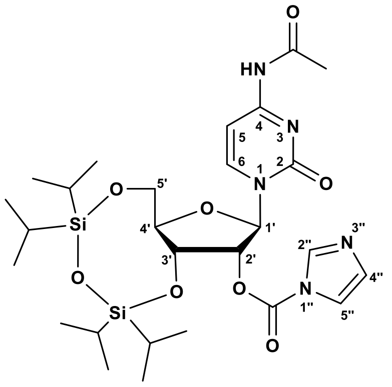

Compound **2-C** (2130 mg, 4.04 mmol, 1.0 eq.) was dissolved in dry DCM (24 mL) and CDI (1309 mg, 8.07 mmol, 2.0 eq.) was added. The reaction mixture was stirred for 18 h. The reaction mixture was diluted with DCM (40 mL) and washed with water (40 mL) and a saturated solution of sodium bicarbonate (2x40 mL). The organic layer was dried over sodium sulfate and the solvent was removed under reduced pressure to yield **3-C** as a colourless foam (Yield: 2466 mg, 3.97 mmol, 98.3%).

MF: $C_{27}H_{43}N_5O_8Si_2$ **MW:** 621.84. R_F: 0.30 (SiO_2, EA).

LCMS (pos., ACN) (*m/z*): Calculated for $[C_{27}H_{43}N_5O_8Si_2H]^+$: 622.28, found: 622.30.

^{1}H-NMR (400 MHz, $CDCl_3$, 298 K, COSY): *δ*/ppm = 10.08 (s, 1H, *H*N-4), 8.19 – 8.12 (m, 2H, H-2’’, H-6), 7.50 – 7.40 (m, 2H, H-5’’, H-5), 7.10 – 7.06 (m, 1H, H-4’’), 6.01 (d, *J* = 11.7 Hz, 1H, H-1’), 5.57 (dd, *J* = 11.6 Hz, *J* = 4.7 Hz, 1H, H-2’), 4.44 (ddd, *J* = 11.7 Hz, *J* = 9.3 Hz, *J* = 4.6 Hz, 1H, H-3’), 4.30 (t, *J* = 12.3 Hz, 1H, H-5’), 4.15 – 4.09 (m, 1H, H-4’), 4.00 (td, 1H, *J* = 11.7 Hz, *J* = 2.8 Hz, H-5’), 2.28 (s, 3H, C*H*$_3$ (AcNH-4)), 1.11 – 0.79 (m, 28H, TIPDS).

^{13}C-NMR (101 MHz, $CDCl_3$, 298 K, HSQC, HMBC, DEPT): *δ*/ppm = 171.20 (*C*=O (AcNH-4)), 163.53 (C-4), 154.87 (C-2), 147.19 (*C*=O^{Carbamate}), 143.75 (C-6), 137.29 (C-2’’), 130.96 (C-4’’), 117.31 (C-5’’), 97.16 (C-5), 88.87 (C-1’), 82.68 (C-4’), 79.26 (C-2’), 67.71 (C-3’), 59.44 (C-5’), 25.04 (*C*H$_3$ (AcNH-4)),

17.52 (CH_3^{TIPDS}), 17.48 (CH_3^{TIPDS}), 17.45 (CH_3^{TIPDS}), 17.36 (CH_3^{TIPDS}), 16.86 (CH_3^{TIPDS}), 16.80 (CH_3^{TIPDS}), 16.77 (CH_3^{TIPDS}), 16.73 (CH_3^{TIPDS}), 13.47 (CH^{TIPDS}), 12.99 (CH^{TIPDS}), 12.97 (CH^{TIPDS}), 12.58 (CH^{TIPDS}).

Compound 4-C

(2’-O-((2’’,3’’-Dihydroxypropyl) carbamoyl)-3',5'-O-(1,1,3,3-tetraisopropyldisilox-1,3-diyl) cytidine (NAc))

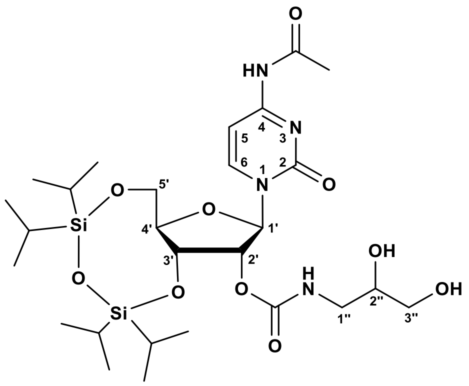

Compound **3-C** (2447 mg, 3.94 mmol, 1.0 eq.) was dissolved in dry DCM (22 mL) and 1-aminoglycerol (538 mg, 5.90 mmol, 1.5 eq.) was added to the reaction mixture. DIPEA (1526 mg, 11.81 mmol, 3.0 eq.) was added and the reaction mixture was stirred for 20 h. After 3.5 h of stirring, another portion of 1-aminoglycerol (117 mg, 1.28 mmol, 0.32 eq.) was added. The reaction mixture was diluted with DCM (50 mL) and washed with water (25 mL) and a saturated solution of sodium bicarbonate (2x25 mL). The organic layer was dried over sodium sulfate and the solvent was removed under reduced pressure. The residue was purified *via* column chromatography on silica (EA) to yield **4-C** as a colourless foam (Yield: 1426.1 mg, 2.21 mmol, 56.1%).

MF: $C_{27}H_{48}N_4O_{10}Si_2$ **MW:** 644.87. R_F: 0.27 (SiO_2, DCM:MeOH, 9:1).

LCMS (neg., ACN) (*m/z*): Calculated for $[C_{27}H_{47}N_4O_{10}Si_2]^-$: 643.28, found: 643.30.

^{1}H-NMR (400 MHz, DMSO-d_6, 298 K): δ/ppm = 10.97 (s, 1H, *H*N-4), 8.08 (dd, J = 7.6 Hz, J = 2.2 Hz, 1H, H-6), 7.22 (m, 2H, H-5^{Cytosine}, *H*N-1’’), 5.73 (d, J = 2.0 Hz, 1H, H-1’), 5.27 (d, J = 4.2 Hz, 1H, H-2’),

4.66 (ddd, J = 17.0 Hz, J = 5.0 Hz, J = 2.3 Hz, 1H, H-3’), 4.45 (dtd, J = 8.3 Hz, J = 5.7 Hz, J = 2.3 Hz, 1H, *H*O-2’’), 4.38 (ddt, J = 8.4 Hz, J = 5.1 Hz, J = 2.5 Hz, 1H, *H*O-3’’), 4.18 (dd, J = 13.0 Hz, J = 3.1 Hz, 1H, H-5’), 4.06 – 3.89 (m, 3H, H-4’, H-5’, H-2’’), 3.53 – 3.42 (m, 1H, H-3’’), 3.29 (qd, J = 5.8 Hz, J = 2.1 Hz, 1H, H-3’’), 3.15 – 3.01 (m, 1H, H-1’’), 3.01 – 2.85 (m, 1H, H-1’’), 2.10 (s, 3H, CH_3 (AcNH-4)), 1.08 – 0.95 (m, 28H, TIPDS).

^{13}C-NMR (101 MHz, DMSO-d_6, 298 K, HSQC, HMBC, DEPT): δ/ppm = 171.12 (*C*=O (AcNH-4), 162.73 (C-4), 155.05 (C=$O^{Carbamate}$), 154.41 (C-2), 142.13 (C-6), 94.00 (C-5), 89.18 (C-1’), 81.56 (C-4’), 74.69 (C-2’), 70.64 (C-2’’), 68.91 (C-3’), 64.24 (C-3’’), 60.64 (C-5’), 43.96 (C-1’’), 27.49 (CH$_3$ (AcNH-4)), 17.35 (CH_3^{TIPDS}), 17.25 (CH_3^{TIPDS}), 17.18 (CH_3^{TIPDS}), 17.15 (CH_3^{TIPDS}), 16.79 (2xCH_3^{TIPDS}), 16.75 (2xCH_3^{TIPDS}), 12.72 (CH^{TIPDS}), 12.37 (CH^{TIPDS}), 12.23 (CH^{TIPDS}), 12.06 (CH^{TIPDS}).

Compound 5-C

(2’-O-((2’’,3’’-Diacetoxypropyl) carbamoyl)-3',5'-O-(1,1,3,3-tetraisopropyldisilox-1,3-diyl) cytidine (NAc))

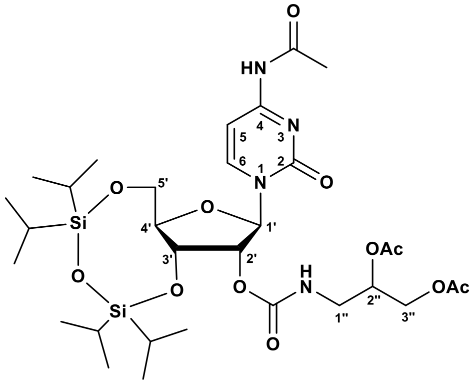

Compound **4-C** (1250 mg, 1.94 mmol, 1.0 eq.) was dissolved in dry pyridine (12 mL). DMAP (2.5 mg, 0.02 mmol, 0.01 eq.) and acetic anhydride (617 mg, 6.05 mmol, 3.0 eq.) were added to the solution. After stirring the reaction mixture for 24 h, the solvent was removed under reduced pressure and the residue was

purified *via* column chromatography on silica (DCM:MeOH, 1:0 → 19:1) to yield **5-C** as a colourless solid (Yield: 1238 mg, 1.70 mmol, 87.6%).

MF: $C_{31}H_{52}N_4O_{12}Si_2$ **MW:** 728.94. R_F: 0.70 (SiO_2, DCM:MeOH, 9:1).

LCMS (neg., ACN) (*m/z*): Calculated for $[C_{31}H_{51}N_4O_{12}Si_2]^-$: 727.30, found: 727.30.

^{1}H-NMR (400 MHz, DMSO-d_6, 298 K, COSY): δ/ppm = 10.96 (s, 1H, *H*N-4), 8.06 (d, J = 7.6 Hz, 1H, H-6), 7.63 (q, J = 6.4 Hz, 1H, *H*N-1''), 7.22 (d, J = 7.4 Hz, 1H, H-5), 5.72 (s, 1H, H-1'), 5.30 (d, J = 5.1 Hz, 1H, H-2'), 4.93 (pd, J = 12.1 Hz, J = 3.1 Hz, 1H, H-2''), 4.39 (t, J = 6.1 Hz, 1H, H-3'), 4.21 – 4.14 (m, 2H, H-5', H-3''), 4.06 – 3.92 (m, 3H, H-4', H-5', H-3''), 3.31 – 3.15 (m, 2H, H-1''), 2.10 (d, J = 0.9 Hz, 3H, CH_3 (AcNH-4)), 2.00 (s, 3H, CH_3 (AcO-2'')), 2.00 (s, 3H, CH_3 (AcO-2'')), 1.06 – 0.93 (m, 28H, TIPDS).

^{13}C-NMR (101 MHz, $CDCl_3$, 298 K, HSQC, HMBC, DEPT): δ/ppm = 171.06 (*C*=O (AcNH-4), 170.08 (*C*=O (AcO-3'')), 169.82 (*C*=O (AcO-2'')), 162.74 (C-4), 155.06 (C=O^{Carbamate}), 154.07 (C-2), 144.61 (C-6), 95.37 (C-5), 89.60 (C-1'), 81.71 (C-4'), 74.97 (C-2'), 70.21 (C-2''), 68.38 (C-3'), 62.71 (C-3''), 60.32 (C-5'), 40.38 (C-1''), 24.38 (CH$_3$ (AcNH-4)), 20.83 (CH$_3$ (AcO-2'')), 20.53 (CH$_3$ (AcO-3'')), 17.32 (CH_3^{TIPDS}), 17.19 (CH_3^{TIPDS}), 17.15 (CH_3^{TIPDS}), 17.09 (CH_3^{TIPDS}), 16.75 (2xCH_3^{TIPDS}), 16.71 (CH_3^{TIPDS}), 16.68 (CH_3^{TIPDS}), 12.69 (CH^{TIPDS}), 12.36 (CH^{TIPDS}), 12.25 (CH^{TIPDS}), 11.98 (CH^{TIPDS}).

Compound 6-C

(2'-O-((2'',3''-Diacetoxypropyl) carbamoyl) cytidine (NAc))

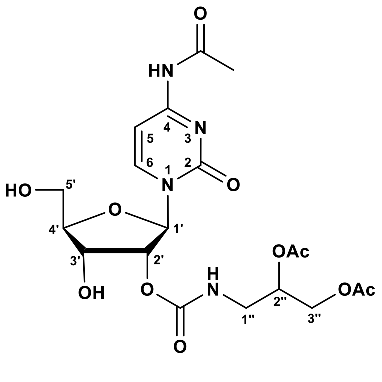

Compound **5-C** (1521 mg, 2.09 mmol, 1.0 eq.) was dissolved in dry pyridine (20 mL) and triethylamine trihydrofluoride (1009 mg, 6.26 mmol, 3.0 eq.) was added to the solution. After stirring for 24 h, the solvent was removed under reduced pressure and the residue was purified *via* column chromatography on silica (DCM:MeOH, 32:1 → 9:1) to yield **6-C** as a yellowish solid (Yield: 791 mg, 1.63 mmol, 78.0%).

MF: $C_{19}H_{26}N_4O_{11}$ **MW:** 486.43. R_F: 0.16 (SiO_2, DCM:MeOH, 15:1).

LCMS (pos., ACN) (*m/z*): Calculated for $[C_{19}H_{26}N_4O_{11}H]^+$: 487.17, found: 487.15.

^{1}H-NMR (400 MHz, DMSO-d_6, 298 K, COSY): δ/ppm = 10.91 (s, 1H, *H*N-4), 8.38 (t, J = 6.4 Hz, 1H, H-6), 7.53 (q, J = 5.8 Hz, 1H, *H*N-1''), 7.19 (t, J = 6.3 Hz, 1H, H-5), 5.97 – 5.94 (m, 1H, H-1'), 5.46 (dt, J = 14.0 Hz, J = 5.3 Hz, 1H, *H*O-3'), 5.22 (q, J = 5.1 Hz, 1H, *H*O-5'), 5.05 (t, J = 4.8 Hz, 1H, H-2'), 4.93 (p, J = 5.7 Hz, 1H, H-2''), 4.23 – 4.13 (m, 2H, H-3', H-3''), 4.02 (ddt, J = 12.5 Hz, J = 6.0 Hz, J = 3.0 Hz, 1H, H-3''), 3.90 (q, J = 4.5 Hz, 1H, H-4'), 3.73 (d, J = 11.7 Hz, 1H, H-5'), 3.63 – 3.57 (m, 1H, H-5'), 3.27 – 3.14 (m, 2H, 2xH-1''), 2.10 (d, J = 5.1 Hz, 3H, CH_3 (AcNH-4)), 2.01 – 1.97 (m, 6H, CH_3 (AcO-2''), CH_3 (AcO-3'')).

^{13}C-NMR (101 MHz, DMSO-d_6, 298 K, HSQC, HMBC, DEPT): δ/ppm = 171.15 (*C*=O (AcNH-4), 170.23 (*C*=O (AcO-3'')), 169.97 (*C*=O (AcO-2'')), 162.59 (C-4), 155.43 ($C{=}O^{Carbamate}$), 154.57 (C-2), 145.50 (C-6), 95.65 (C-5), 87.80 (C-1'), 85.07 (C-4'), 75.95 (C-2'), 70.13 (C-2''), 67.94 (C-3'), 62.75 (C-3''), 60.11 (C-5'), 40.31 (C-1''), 24.42 (CH_3 (AcNH-4)), 20.85 (CH_3 (AcO-3'')), 20.60 (CH_3 (AcO-2'')).

Compound 7-C

(2'-O-((2'',3''-Diacetoxypropyl) carbamoyl)-5'-O-DMT-cytidine (NAc))

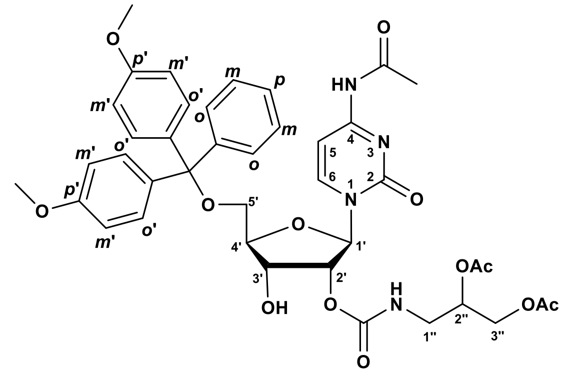

Compound **6-C** (600 mg, 1.23 mmol, 1.0 eq.) was dried in vacuo overnight and co-evaporated four times with dry pyridine (2 mL), before it was dissolved in dry pyridine (24 mL). DIPEA (594 mg, 4.60 mmol, 3.7 eq.), a molecular sieve (4 Å) and 4,4'-dimethoxytritylchloride (2000 mg, 5.90 mmol, 4.8 eq.) was added in one portion and the reaction mixture was stirred for 15 h at room temperature under argon atmosphere. The solvent was removed under reduced pressure and the residue was purified via column chromatography on silica (DCM:MeOH, 1:0 → 49:1, with 1% Et_3N) to yield **7-C** as a slightly yellowish foam (Yield: 953.5 mg, 1.21 mmol, 98.4%).

MF: $C_{40}H_{44}N_4O_{13}$ **MW:** 788.81. R_F: 0.43 (SiO_2, DCM:MeOH, 19:1).

LCMS (pos., ACN) (*m/z*): Calculated for $[C_{40}H_{44}N_4O_{13}H]^+$: 789.30, found: 789.30.

HRMS (ESI-MS, pos. mode): calculated for $[C_{40}H_{44}N_4O_{13}H]^+$: 789.2977, found: 789.2949

^{1}H-NMR (400 MHz, DMSO-d_6, 298 K, COSY): δ/ppm = 10.90 (s, 1H, *H*N-4), 8.16 (dd, J = 7.6 Hz, J = 2.1 Hz, 1H, H-6), 7.52 (t, J = 6.1 Hz, 1H, *H*N-1''), 7.36 – 7.18 (m, 9H, 4xH-*o*', 2xH-*o*, 2xH-*m*, H-*p*), 6.98 (dd, J = 7.5 Hz, J = 2.0 Hz, 1H, H-5), 6.86 – 6.83 (m, 4H, 2xH-*m*'), 5.86 (t, J = 2.5 Hz, 1H, H-1'), 5.51 (ddd, J = 15.2 Hz, J = 6.0 Hz, J = 2.1 Hz, 1H, *H*O-3'), 5.09 (dt, J = 5.1 Hz, J = 2.4 Hz, 1H, H-2'),4.92 – 4.87 (m, 1H, H-2''), 4.33 (q, J = 6.5 Hz, 1H, H-3'), 4.15 – 4.09 (m, 1H, H-3''), 4.01 –3.95 (m, 2H, H-4', H-3''), 3.70-3.68 (m, 6H, 2xC*H*3O-*p*'), 3.34 – 3.11 (m, 4H, 2xH-5', 2xH-1''), 2.04 (d, J = 2.1 Hz, 3H, C*H*3 (AcNH-4)), 1.95 – 1.93 (m, 6H, 2xC*H*3 (AcO-2'', AcO-3'')).

^{13}C-NMR (101 MHz, DMSO-d_6, 298 K, HSQC, HMBC, DEPT): δ/ppm = 171.07 (*C*=O (AcNH-4), 170.20 (*C*=O (AcO'')), 169.96 (*C*=O (AcO'')), 162.61 (C-4), 158.18 (2xC-*p*'), 155.40 (C=O^{Carbamate}), 154.28 (C-2), 145.10 (C-6), 144.52 (C-*ipso*), 135.42 (C-*ipso*'), 135.15 (C-*ipso*'), 129.81 (4xC-*o*'), 127.97 (2xC-*o*), 127.77 (2xC-*m*), 126.86 (C-*p*), 113.29 (4xC-*m*'), 95.57 (C-5), 88.82 (C-1'), 85.98 (C_q^{DMT}), 82.61 (C-4'), 75.73 (C-2'), 70.14 (C-2''), 67.82 (C-3'), 62.73 (C-3''), 62.28 (C-5'), 55.04 (2xCH$_3$O-*p*'), 40.29 (C-1''), 24.39 (CH$_3$ (AcNH-4)), 20.85 (CH$_3$ (AcO'')), 20.58 (CH$_3$ (AcO'')).

Compound 8-C

(2'-O-((2,3-Diacetoxypropyl) carbamoyl)-5'-O-DMT-3'-O-((2-cyanoethoxy, diisopropyl-amino, phosphanyl) Cytidine (NAc))

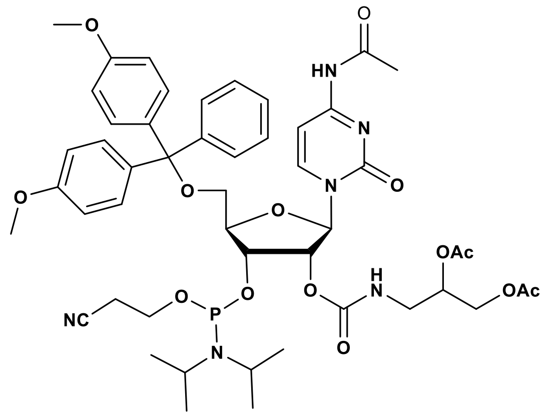

Compound **7-C** (250 mg, 0.32 mmol, 1.0 eq.) was co-evaporated together with dry pyridine (10mL) to remove the traces of water thrice. Under argon atmosphere, **7-C** was dissolved in dry DCM (10 mL), DIPEA (128 mg, 1 mmol, 3.0 eq.) was added and 2-Cyanoethyl *N*,*N*-diisopropylchlorophosphoramidite (151.5 mg, 0.64 mmol, 2 eq.) was added dropwise over 5 min and reaction mixture was stirred for 3 h at room temperature under argon atmosphere. After removal of the solvent, the residue was purified via column chromatography on silica (Hex:EA, 1:1 → 0:1, with 1% Et_3N) under argon atmosphere. The slightly yellowish foam obtained in this way was dissolved in dry DCM (3 mL) and precipitated with cold hexane (40 mL) while stirring. This procedure was repeated three times and the product was collected by centrifugation and drying in vacuo to yield a colourless foam (Yield: 162 mg, 0.16 mmol, 52.3%).

MF: $C_{49}H_{61}N_6O_{14}P$ **MW:** 989.00.

^{31}P-NMR (162 MHz, $CDCl_3$, 298 K): δ/ppm = 151.16, 151.11, 150.87, and 150.74.

Compound 2-A

(3',5'-O-(1,1,3,3-Tetraisopropyldisilox-1,3-diyl) adenosine (NBz))

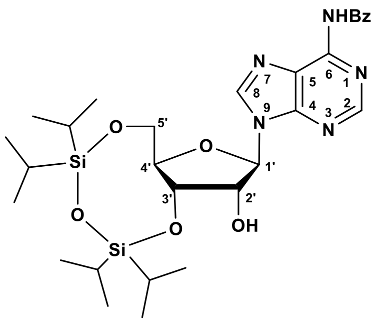

The benzoyl protected adenosine (**1-A**) (1512 mg, 4.07 mmol, 1.0 eq.) was dissolved in dry pyridine (20 mL) and 1,3-dichloro-1,1,3,3-tetraisopropyldisiloxane (1380 mg, 4.38 mmol, 1.08 eq.) was added dropwise to the solution over 60 min. The reaction mixture was stirred at room temperature for 18 h before the solvent was removed under reduced pressure and the residue was purified *via* column chromatography on silica (Hex:EA, 3:1 → 1:1) to yield **2-A** as a colourless solid (Yield: 1714.5 mg, 2.79 mmol, 68.6%).

MF: $C_{29}H_{43}N_5O_6Si_2$ **MW:** 613.86. R_F: 0.71 (SiO_2, EA).

LCMS (pos., ACN) (*m*/*z*): Calculated for $[C_{29}H_{43}N_5O_6Si_2H]^+$: 614.2878, found: 614.25.

^{1}H-NMR (400 MHz, $CDCl_3$, 298 K, COSY): δ/ppm = 9.17 (s, 1H, *H*N-6), 8.74 (s, 1H, H-2), 8.15 (s, 1H, H-8), 8.02 (dd, J = 7.0 Hz, J = 1.6 Hz, 2H, H-o^{Bz}), 7.59 (tt, J = 7.4 Hz, J = 1.3 Hz, 1H, H-p^{Bz}), 7.51 (tt, J = 7.8 Hz, J = 1.6 Hz, 2H, H-m^{Bz}, H-m^{Bz}), 6.03 (d, J = 1.3 Hz, 1H, H-1'), 5.11 (dd, J = 7.9 Hz, J = 5.5 Hz, 1H, H-3'), 4.62 (dd, J = 5.5 Hz, J = 1.3 Hz, 1H, H-2'), 4.16 – 4.09 (m, 2H, H-4', H-5'), 4.07 – 4.01 (m, 1H, H-5'), 3.34 (s, 1H, *H*O-2'), 1.14 – 1.03 (m, 28H, TIPDS).

^{13}C-NMR (101 MHz, $CDCl_3$, 298 K, HSQC, HMBC, DEPT): δ/ppm = 164.81 (*C*=O^{Bz}), 152.78 (C-2), 151.12 (C-4), 149.81 (C-6), 142.12 (C-8), 133.78 (C-p^{Bz}), 132.92 (C-*ipso*^{Bz}), 128.98 (C-m^{Bz}), 128.02

(C-o^{Bz}), 123.77 (C-5), 89.93 (C-1'), 82.36 (C-4'), 75.19 (C-2'), 70.83 (C-3'), 61.69 (C-5'), 17.56 (CH_3^{TIPDS}), 17.47 (2xCH_3^{TIPDS}), 17.39 (CH_3^{TIPDS}), 17.22 (CH_3^{TIPDS}), 17.11 (CH_3^{TIPDS}), 17.08 (CH_3^{TIPDS}), 17.02 (CH_3^{TIPDS}), 13.38 (CH^{TIPDS}), 13.15 (CH^{TIPDS}), 12.87 (CH^{TIPDS}), 12.72 (CH^{TIPDS}).

Compound 3-A

(2'-O-Carboimidazolyl-3',5'-O-(1,1,3,3-tetraisopropyldisilox-1,3-diyl) adenosine (NBz))

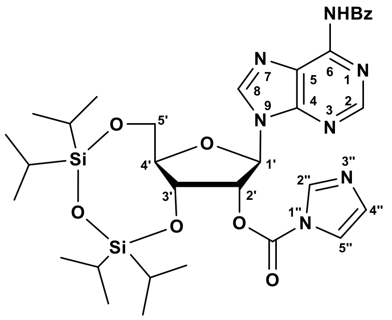

Compound **2-A** (1700 mg, 2.77 mmol, 1.0 eq.) was dissolved in dry DCM (25 mL) and CDI (1250 mg, 7.71 mmol, 2.8 eq.) was added. The reaction mixture was stirred for 100 min before it was diluted with DCM (40 mL) and washed with water (30 mL) and a saturated solution of sodium bicarbonate (2x30 mL). The aqueous phase was extracted with DCM (20 mL), the combined organic phase was dried over sodium sulfate and the solvent was removed under reduced pressure to yield **3-A** as a colourless foam (Yield: 1806 mg, 2.55 mmol, 92.1%).

MF: $C_{33}H_{45}N_7O_7Si_2$ **MW:** 707.94. R_F: 0.28 (SiO_2, EA).

LCMS (pos., ACN) (*m/z*): Calculated for $[C_{33}H_{45}N_7O_7Si_2H]^+$: 708.2978, found: 708.30.

^{1}H-NMR (400 MHz, $CDCl_3$, 298 K, COSY): δ/ppm = 9.06 (s, 1H, *H*N-6), 8.76 (s, 1H, H-2), 8.21 (s, 1H, H-2''), 8.20 (s, 1H, H-8), 8.02 (dd, J = 7.0 Hz, J = 1.4 Hz, 2H, H-o^{Bz}), 7.62 (tt, J = 7.2 Hz, J = 1.5 Hz, 1H, H-p^{Bz}), 7.53 (tt, J = 7.9 Hz, J = 1.2 Hz, 2H, H-m^{Bz}), 7.48 (t, J = 1.5 Hz, 1H, H-5''), 7.13 (dd, J = 1.7 Hz, J = 0.8 Hz, 1H, H-4''), 6.26 (s, 1H, H-1'), 6.04 (d, J = 5.2 Hz, 1H, H-2'), 5.31 (dd, J = 8.7 Hz, J = 5.3 Hz,

1H, H-3’), 4.20 (dd, J = 12.8 Hz, J = 2.9 Hz, 1H, H-5’), 4.12 (dt, J = 10.1 Hz, J = 3.6 Hz, 1H, H-4’), 4.07 (dd, J = 12.8 Hz, J = 2.8 Hz, 1H, H-5’), 1.12 – 0.86 (m, 28H, TIPDS).

^{13}C-NMR (101 MHz, $CDCl_3$, 298 K, HSQC, HMBC, DEPT): δ/ppm = 164.71 (C=O^{Bz}), 153.05 (C-2), 151.08 (C-4), 149.95 (C-6), 147.61 (C=$O^{Carbamate}$), 141.76 (C-8), 137.28 (C-2’’), 133.63 (C-p^{Bz}), 133.08 (C-$ipso^{Bz}$), 131.16 (C-4’’), 129.08 (C-m^{Bz}), 128.02 (C-o^{Bz}), 123.75 (C-5), 117.30 (C-5’’), 87.21 (C-1’), 82.47 (C-4’), 79.66 (C-2’), 69.57 (C-3’), 60.50 (C-5’), 17.52 (CH_3^{TIPDS}), 17.48 (CH_3^{TIPDS}), 17.43 (CH_3^{TIPDS}), 17.37 (CH_3^{TIPDS}), 16.99 (CH_3^{TIPDS}), 16.94 (CH_3^{TIPDS}), 16.81 (CH_3^{TIPDS}), 16.76 (CH_3^{TIPDS}), 13.38 (CH^{TIPDS}), 13.06 (CH^{TIPDS}), 12.87 (CH^{TIPDS}), 12.74 (CH^{TIPDS}).

Compound 4-A

(2’-O-((2’’,3’’-Dihydroxypropyl) carbamoyl)-3',5'-O-(1,1,3,3-tetraisopropyldisilox-1,3-diyl) adenosine (NBz))

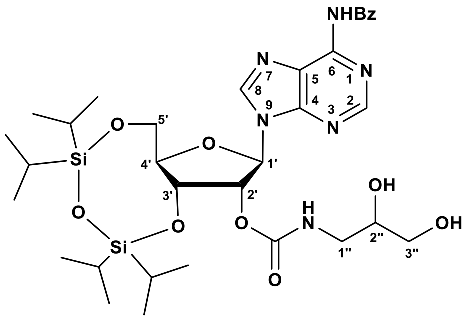

Aminoglycerol (470 mg, 5.16 mmol, 2.1 eq.) was co-evaporated with dry pyridine (4x1 mL) and dissolved in dry pyridine (18 mL) under argon atmosphere. A molecular sieve (pore size 4 Å) and DIPEA (393 mg, 3.04 mmol, 3.0 eq.) was added. Compound **3-A** (1750 mg, 2.47 mmol, 1.0 eq.) was dissolved separately in dry pyridine (2 mL) and added to the reaction mixture. After stirring for 90 min, the solvent was removed under reduced pressure, the residue was diluted with DCM (100 mL) and washed with a saturated solutions of sodium bicarbonate (2x40 mL) and Brine (40 mL). The organic layer was dried over sodium sulfate and the solvent was removed under reduced pressure. The residue was purified *via* column

chromatography on silica (DCM:MeOH, 32:1 → 9:1) to yield **4-A** as a colourless foam (Yield: 734 mg, 1.00 mmol, 40.5%).

MF: $C_{33}H_{50}N_6O_9Si_2$ **MW:** 730.97. R_F: 0.52 (SiO_2, DCM:MeOH, 9:1).

^{1}H-NMR (400 MHz, $CDCl_3$, 298 K, COSY): δ/ppm = 9.35 (s, 1H, *H*N-6), 8.76 (s, 1H, H-2), 8.23 (d, *J* = 3.2 Hz, 1H, H-8), 8.03 – 8.00 (m, 2H, H-o^{Bz}), 7.58 (dd, *J* = 7.3 Hz, *J* = 1.4 Hz, 1H, H-p^{Bz}), 7.49 (dd, *J* = 8.3 Hz, *J* = 6.8 Hz, 2H, H-m^{Bz}), 6.08 (d, *J* = 2.9 Hz, 1H, H-1'), 5.76 (dd, *J* = 27.2 Hz, *J* = 5.8 Hz, 1H, *H*N-1''), 5.65 (d, *J* = 5.4 Hz, 1H, H-2'), 5.07 – 5.01 (m, 1H, H-3'), 4.13 (dd, *J* = 13.8 Hz, *J* = 2.9 Hz, 1H, H-5'), 4.03 – 3.96 (m, 2H, H-4', H-5'), 3.80 (ddq, *J* = 15.3 Hz, *J* = 10.2 Hz, *J* = 4.7 Hz, 1H, H-2''), 3.65 – 3.59 (m, 1H, H-3''), 3.54 (td, *J* = 11.1 Hz, *J* = 5.5 Hz, 1H, H-3''), 3.44 – 3.14 (m, 2H, 2xH-1''), 1.12 – 0.89 (m, 28H, TIPDS).

^{13}C-NMR (101 MHz, $CDCl_3$, 298 K, HSQC, HMBC, DEPT): δ/ppm = 165.15 (*C*=O^{Bz}), 156.15 (*C*=$O^{Carbamate}$), 152.84 (C-2), 151.06 (C-4), 149.75 (C-6), 142.10 (C-8), 133.67 (C-p^{Bz}), 132.99 (C-$ipso^{Bz}$), 128.95 (C-m^{Bz}), 128.11 (C-o^{Bz}), 123.48 (C-5), 88.02 (C-1'), 82.24 (C-4'), 76.35 (C-2'), 71.13 (C-2''), 69.11 (C-3'), 64.01 (C-3''), 60.48 (C-5'), 43.68 (C-1''), 17.55 (CH_3^{TIPDS}), 17.49 (CH_3^{TIPDS}), 17.44 (CH_3^{TIPDS}), 17.40 (CH_3^{TIPDS}), 17.07 (2xCH_3^{TIPDS}), 17.02 (CH_3^{TIPDS}), 16.99 (CH_3^{TIPDS}), 13.43 (CH^{TIPDS}), 13.08 (CH^{TIPDS}), 12.91 (CH^{TIPDS}), 12.76 (CH^{TIPDS}).

Compound 5-A

(2'-O-((2'',3''-Diacetoxypropyl) carbamoyl)-3',5'-O-(1,1,3,3-tetraisopropyldisilox-1,3-diyl) adenosine (NBz))

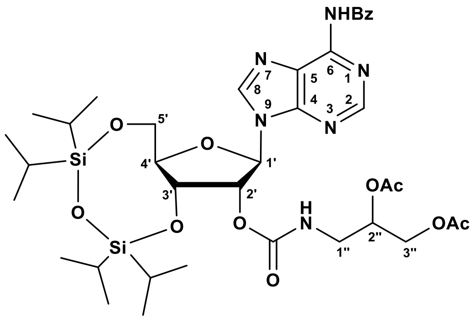

Compound **4-A** (700 mg, 0.96 mmol, 1.0 eq.) was dissolved in dry pyridine (10 mL). DMAP (1.2 mg, 0.01 mmol, 0.01 eq.) and acetic anhydride (756 mg, 7.41 mmol, 7.7 eq.) were added to the solution. After stirring the reaction mixture for 17 h, the solvent was removed under reduced pressure and the residue was dissolved in DCM (50 mL). The solution was washed with a saturated solution of sodium bicarbonate (2x20 mL) and the aqueous phase was extracted with DCM (20 mL). The combined organic fractions were dried over sodium sulfate and the solvent was removed under reduced pressure. The residue was purified *via* column chromatography on silica (DCM:EA, 1:1 → 0:1) to yield **5-A** as a colourless foam (Yield: 688 mg, 0.84 mmol, 87.5%).

MF: $C_{37}H_{54}N_6O_{11}Si_2$ **MW:** 815.04. R_F: 0.82 (SiO_2, DCM:MeOH, 9:1).

LCMS (neg., ACN) (*m*/*z*): Calculated for $[C_{37}H_{53}N_6O_{11}Si_2]^-$: 813.33, found: 813.30.

^{1}H-NMR (400 MHz, $CDCl_3$, 298 K, COSY): (A: NHBz, B: NAcBz), A:B, 1:1, δ/ppm = 9.12 (s, 0.5H, *H*N-6-A), 8.75 (s, 0.5H, H-2-B), 8.63 (d, *J* = 0.6 Hz, 0.5H, H-2-A), 8.25 (d, *J* = 2.4 Hz, 0.5H, H-8-B), 8.19 (d, *J* = 3.4 Hz, 0.5H, H-8-A), 8.03 (dq, *J* = 7.0 Hz, *J* = 1.8 Hz, 1H, H-o^{Bz}-B), 7.72 – 7.68 (m, 1H, 1H, H-o^{Bz}-A), 7.63 – 7.57 (m, 0.5H, H-p^{Bz}-B), 7.54 – 7.49 (m, 1H, H-m^{Bz}-B), 7.44 – 7.39 (m, 0.5H, H-p^{Bz}-A), 7.31 – 7.27 (m, 1H, H-m^{Bz}-A), 6.06 (d, *J* = 1.3 Hz, 0.5H, H-1'-A), 6.02 (d, *J* = 1.2 Hz, 0.5H, H-1'-B), 5.70 – 5.65 (m, 1H, H-2'), 5.23 (p, *J* = 6.0 Hz, 1H, *H*N-1''), 5.17 – 5.02 (m, 2H, H-3', H-2''), 4.32 – 4.22 (m, 1H, H-3''), 4.19 – 4.10 (m, 2H, H-5', H-3''), 4.07 – 3.98 (m, 2H, H-4', H-5'), 3.56 – 3.28 (m, 2H, 2xH-1''), 2.59 (s, 1.5H, CH_3 (AcN-6-B)), 2.10 – 2.06 (m, 6H, CH_3 (AcO-3''), CH_3 (AcO-2'')), 1.09 – 1.04 (m, 28H, TIPDS).

^{13}C-NMR (101 MHz, $CDCl_3$, 298 K, HSQC, HMBC, DEPT): (A: NHBz, B: NAcBz), A:B, 1:1, δ/ppm = 172.72 (*C*=O (AcN-6-B)), 172.07 (*C*=O^{Bz}-A), 170.87 (*C*=O (AcO-3'')), 170.47 (*C*=O (AcO-2'')), 164.68 (*C*=O^{Bz}-B), 155.19 (*C*=$O^{Carbamate}$), 152.94 (C-2-B), 152.43 (C-4-B), 152.33 (C-2-A), 151.12 (C-6-A), 151.04 (C-4-A), 149.77 (C-6-B), 144.31 (C-8-B), 142.09 (C-8-A), 134.26 (C-*ipso*^{Bz}-A), 133.81 (C-*ipso*^{Bz}-B), 133.01 (C-*p*^{Bz}-A), 132.90 (C-*p*^{Bz}-B), 129.39 (C-*o*^{Bz}-A), 129.24 (C-5-B), 128.98 (C-*m*^{Bz}-B), 128.76 (C-*m*^{Bz}-A), 128.03 (C-*o*^{Bz}-B), 123.59 (C-5-A), 88.11 (C-1'), 82.22 (C-4'), 76.28 (C-2'), 70.46 (C-2''), 69.22 (C-3'), 62.63 (C-3''), 60.65 (C-5'), 60.49 (C-5'), 41.50 (C-1''), 41.31 (C-1''), 25.54 (CH_3 (AcN-6-B)), 21.08 (CH_3 (AcO-2'')), 20.86 (CH_3 (AcO-3'')), 17.52 (CH_3^{TIPDS}), 17.48 (CH_3^{TIPDS}), 17.44 (CH_3^{TIPDS}), 17.40 (CH_3^{TIPDS}), 17.07 (2xCH_3^{TIPDS}), 17.03 (2xCH_3^{TIPDS}), 13.41 (CH^{TIPDS}), 13.07 (CH^{TIPDS}), 12.91 (CH^{TIPDS}), 12.77 (CH^{TIPDS}).

Compound 6-A

(2'-O-((2'',3''-Diacetoxypropyl) carbamoyl) adenosine (NBz))

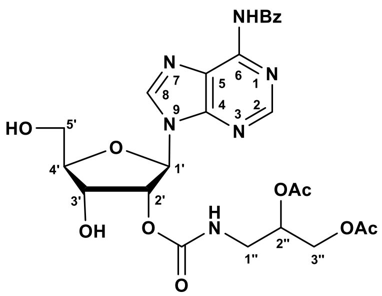

Compound **5-A** (670 mg, 0.82 mmol, 1.0 eq.) was dissolved in dry pyridine (6 mL) and triethylamine trihydrofluoride (398 mg, 2.47 mmol, 3.0 eq.) was added to the solution. After stirring for 24 h, the solvent was removed under reduced pressure and the residue was purified *via* column chromatography on silica (DCM:MeOH, 24:1) to yield **6-A** as a colourless foam (Yield: 292.0 mg, 0.62 mmol, 75.6%).

MF: $C_{25}H_{28}N_6O_{10}$ **MW:** 572.53. R_F: 0.49 (SiO_2, DCM:MeOH, 9:1).

LCMS (pos., ACN) (*m/z*): Calculated for $[C_{25}H_{28}N_6O_{10}H]^+$: 573.20, found: 573.20.

^{1}H-NMR (400 MHz, DMSO-d_6, 298 K, COSY): δ/ppm = 11.24 (s, 1H, *H*N-6), 8.76 (d, J = 1.1 Hz, 1H, H-2), 8.74 (dd, J = 2.4 Hz, J = 1.1 Hz, 1H, H-8), 8.05 (dt, J = 8.5 Hz, J = 1.3 Hz, 2H, H-o^{Bz}), 7.68 – 7.59 (m, 2H, *H*N-1'', H-p^{Bz}), 7.56 (td, J = 7.5 Hz, J = 1.3 Hz, 2H, , H-m^{Bz}), 6.25 (dd, J = 5.8 Hz, J = 1.1 Hz, 1H, H-1'), 5.68 – 5.60 (m, 2H, H-2', *H*O-3'), 5.19 (t, J = 5.4 Hz, 1H, *H*O-5'), 4.90 (tt, J = 6.3 Hz, J = 3.9 Hz, 1H, H-2''), 4.52 – 4.47 (m, 1H, H-3'), 4.13 (dt, J = 12.0 Hz, 4.1 Hz, 1H, H-3''), 4.04 – 3.96 (m, 2H, H-4', H-3''), 3.72 (dt, J = 12.0 Hz, J = 4.5 Hz, 1H, H-5'), 3.60 (dt, J = 11.6 Hz, J = 5.1 Hz, 1H, H-5'), 3.26 – 3.08 (m, 2H, 2xH-1''), 1.98 (dd, J = 2.3 Hz, J = 1.1 Hz, 3H, CH_3 (AcO-3'')), 1.93 (t, J = 1.3 Hz, 3H, CH_3 (AcO-2'')).

^{13}C-NMR (101 MHz, DMSO-d_6, 298 K, HSQC, HMBC, DEPT): δ/ppm = 170.18 (*C*=O (AcO-3'')), 169.88 (*C*=O (AcO-2'')), 165.66 (*C*=O^{Bz}), 155.38 (*C*=$O^{Carbamate}$), 152.11 (C-4), 151.83 (C-2), 150.55 (C-6), 143.20 (C-8), 133.32 (C-p^{Bz}), 132.54 (C-$ipso^{Bz}$), 128.53 (C-o^{Bz}, C-m^{Bz}), 125.76 (C-5), 86.29 (C-4'), 85.31 (C-1'), 75.30 (C-2'), 69.88 (C-2''), 68.97 (C-3'), 62.68 (C-3''), 61.15 (C-5'), 40.28 (C-1''), 20.77 (*C*H_3 (AcO-2'')), 20.56 (*C*H_3 (AcO-3'')).

Compound 7-A

(2'-O-((2'',3''-Diacetoxypropyl) carbamoyl)-5'-O-DMT-adenosine (NBz))

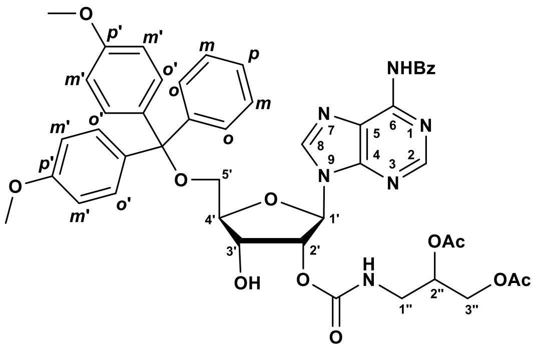

Compound **6-A** (350 mg, 0.61 mmol, 1.0 eq.) was co-evaporated three times with dry pyridine (2 mL), before it was dissolved in dry pyridine (15 mL). DIPEA (237 mg, 1.83 mmol, 3.0 eq.), a molecular sieve (4 Å) and 4,4'-dimethoxytritylchloride (1100 mg, 3.25 mmol, 5.3 eq.) was added in one portion and the

reaction mixture was stirred for 4 h at room temperature under argon atmosphere. The solvent was removed under reduced pressure and the residue was purified via column chromatography on silica (DCM:MeOH, 1:0 → 24:1, with 1% Et_3N) to yield **7-A** as a colourless foam (Yield: 490 mg,0.56 mmol, 91.8%).

MF: $C_{46}H_{46}N_6O_{12}$ **MW:** 874.90. R_F: 0.34 (SiO_2, DCM:MeOH, 19:1).

LCMS (pos., ACN) (*m/z*): Calculated for $[C_{46}H_{46}N_6O_{12}H]^+$: 875.33, found: 875.25.

^{1}H-NMR (400 MHz, DMSO-d_6, 298 K, COSY): *δ*/ppm = 10.76 (s, 1H, *H*N-6), 8.69 (d, *J* = 2.2 Hz, 1H, H-2), 8.62 (d, *J* = 2.5 Hz, 1H, H-8), 8.04 (ddd, *J* = 8.6 Hz, *J* = 2.9 Hz, *J* = 1.4 Hz, 2H, 2xH-o^{Bz}), 7.71 – 7.62 (m, 2H, *H*N-1'', H-p^{Bz}), 7.59 – 7.51 (m, 2H, 2xH-m^{Bz}), 7.34 (ddd, *J* = 10.5 Hz, *J* = 7.2 Hz, *J* = 4.5 Hz, 2H, 2xH-*o*), 7.26 – 7.17 (m, 7H, 4xH-*o*', 2xH-*m*, H-*p*), 6.85 – 6.79 (m, 4H, 4xH-*m*'), 6.27 – 6.23 (m, 1H, H-1'), 5.80 (t, *J* = 5.3 Hz, 1H, H-2'), 5.75 – 5.67 (m, 1H, *H*O-3'), 4.94 (dt, *J* = 8.0 Hz, *J* = 4.2 Hz, 1H, H-2''), 4.72 (d, *J* = 5.5 Hz, 1H, H-3'), 4.18 – 4.10 (m, 2H, H-4', H-3''), 4.02 (dtd, *J* = 12.4 Hz, *J* = 6.3 Hz, *J* = 2.4 Hz, 1H, H-3''), 3.71 (s, 6H, CH_3O-*p*'), 3.29 – 3.11 (m, 4H, 2xH-5', 2xH-1''), 1.99 – 1.97 (m, 3H, CH_3 (AcO-3''), 1.97 – 1.93 (m, 3H, CH_3 (AcO-2'')).

^{13}C-NMR (101 MHz, DMSO-d_6, 298 K, HSQC, HMBC, DEPT): *δ*/ppm = 170.17 (*C*=O (AcO-3'')), 169.90 (*C*=O (AcO-2'')), 165.73 (*C*=O^{Bz}), 158.05 (2xC-*p*'), 155.50 (*C*=$O^{Carbamate}$), 151.86 (C-4), 151.74 (C-2), 150.61 (C-6), 144.78 (C-*ipso*), 143.53 (C-8), 135.51 (C-*ipso*'), 135.40 (C-*ipso*'), 133.34 (C-p^{Bz}), 132.51 (C-$ipso^{Bz}$), 129.70 (4xC-*o*'), 128.55 (2xC-o^{Bz}), 128.49 (2xC-m^{Bz}), 127.80 (2xC-*o*), 127.67 (2xC-*m*), 126.67 (C-*p*), 125.79 (C-5), 113.15 (4xC-*m*'), 86.07 (C-1'), 85.54 (C_q^{DMT}), 83.63 (C-4'), 74.70 (C-2'), 70.10 (C-2''), 68.79 (C-3'), 63.28 (C-5'), 62.69 (C-3''), 55.01 (2x*C*H_3O-*p*'), 40.37 (C-1''), 20.79 (*C*H_3 (AcO-3'')), 20.55 (*C*H_3 (AcO-2'')).

Compound 8-A

(2'-O-((2,3-Diacetoxypropyl) carbamoyl)-5'-O-DMT-3'-O-((2-cyanoethoxy, diisopropyl-amino, phosphanyl) adenosine (NBz))

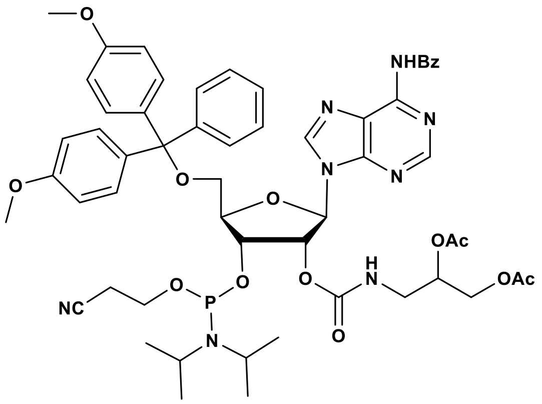

Compound **7-A** (200 mg, 0.23 mmol, 1.0 eq.) was co-evaporated together with dry pyridine (10mL) to remove the traces of water thrice. Under argon atmosphere, **7-A** was dissolved in dry DCM (10 mL), DIPEA (87.9 mg, 0.69 mmol, 3.0 eq.) was added and 2-Cyanoethyl *N*,*N*-isopropylchlorophosphoramidite (81.2 mg, 0.34 mmol, 1.5 eq.) was added dropwise over 5 min and reaction mixture was stirred for 3 h at room temperature under argon atmosphere. After removal of the solvent, the residue was purified via column chromatography on silica (Hex:EA, 1:1 → 0:1, with 1% Et_3N) under argon atmosphere. The slightly yellowish foam obtained in this way was dissolved in dry DCM (3 mL) and precipitated with cold hexane (40 mL) while stirring. This procedure was repeated three times and the product was collected by centrifugation and drying in vacuo to yield **8-A** a colourless foam (Yield: 156 mg, 0.145 mmol, 63.1%).

MF: $C_{55}H_{63}N_8O_{13}P$ **MW:** 1075.1.

^{31}P-NMR (162 MHz, $CDCl_3$, 298 K): δ/ppm = 151.31, 150.63, and 150.43.

Compound 2-G

(3',5'-O-(1,1,3,3-Tetraisopropyldisilox-1,3-diyl) guanosine (iBu))

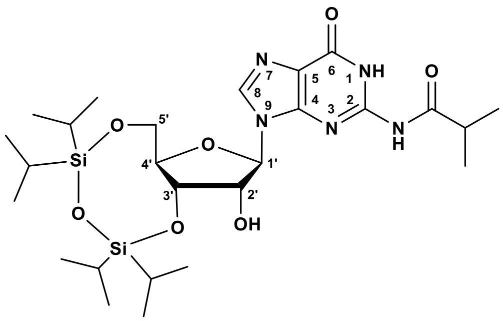

Isobutyryl protected Guanosine **1-G** (1484 mg, 4.20 mmol, 1.0 eq.) was dissolved in dry pyridine (24 mL) and 1,3-dichloro-1,1,3,3-tetraisopropyldisiloxane (1518 mg, 4.81 mmol, 1.15 eq.) was added dropwise to the solution over 60 min while it was cooled on ice. The reaction mixture was stirred at room temperature for 15 h, another portion of 1,3-dichloro-1,1,3,3-tetraisopropyldisiloxane (132 mg, 0.42 mmol, 0.1 eq.) was added and the mixture was stirred for another 60 min. The solvent was removed under reduced pressure and the residue was purified *via* column chromatography on silica (Hex:EA, 2:1 → 0:1) to yield **2-G** as a colourless solid (Yield: 1788 mg,3.00 mmol, 71.5%).

MF: $C_{26}H_{45}N_5O_7Si_2$ **MW:** 595.84. R_F: 0.67 (SiO_2, EA).

LCMS (pos., ACN) (*m/z*): Calculated for $[C_{26}H_{45}N_5O_7Si_2H]^+$: 596.30, found: 596.30.

^{1}H-NMR (400 MHz, $CDCl_3$, 298 K, COSY): δ/ppm = 12.11 (d, J = 6.6 Hz, 1H, H-1), 9.18 (d, J = 6.0 Hz, 1H, *H*N-2), 7.94 (s, 1H, H-8), 5.85 (s, 1H, H-1'), 4.53 (dd, J = 8.1 Hz, J = 5.1 Hz, 1H, H-3'), 4.32 (d, J = 5.4 Hz, 1H, H-2'), 4.19 – 4.00 (m, 3H, H-4', H-5'), 3.48 (s, 1H, *H*O-2'), 2.74 (hept, J = 6.9 Hz, 1H, CH^{Ibu}), 1.29 – 1.24 (m, 6H, 2xCH_3^{Ibu}), 1.08 – 0.98 (m, 28H, TIPDS).

^{13}C-NMR (101 MHz, $CDCl_3$, 298 K, HSQC, HMBC, DEPT): δ/ppm = 178.95 (C=O^{Ibu}), 155.65 (C-6), 147.97 (C-2), 147.68 (C-4), 136.81 (C-8), 121.67 (C-5), 88.95 (C-1'), 82.02 (C-4'), 75.39 (C-2'), 70.00 (C-3'), 61.00 (C-5'), 36.56 (CH^{Ibu}), 19.14 (CH_3^{Ibu}), 17.59 (CH_3^{TIPDS}), 17.55 (CH_3^{TIPDS}), 17.43 (CH_3^{TIPDS}), 17.38 (CH_3^{TIPDS}), 17.25 (CH_3^{TIPDS}), 17.20 (CH_3^{TIPDS}), 17.12 (CH_3^{TIPDS}), 17.01 (CH_3^{TIPDS}), 13.47 (CH^{TIPDS}), 13.13 (CH^{TIPDS}), 13.01 (CH^{TIPDS}), 12.70 (CH^{TIPDS}).

Compound 3-G

(2'-O-Carboimidazolyl-3',5'-O-(1,1,3,3-tetraisopropyldisilox-1,3-diyl) guanosine (iBu))

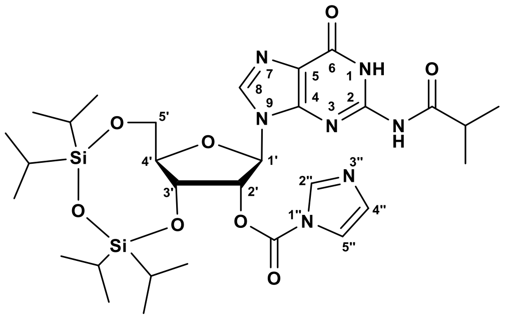

Compound **2-G** (1780 mg, 2.99 mmol, 1.0 eq.) was dissolved in dry DCM (22 mL) and CDI (1211 mg, 7.47 mmol, 2.5 eq.) was added. The reaction mixture was stirred for 5 h before it was diluted with DCM (40 mL) and washed with water (40 mL) and a saturated solution of sodium bicarbonate (2x40 mL). The aqueous phase was extracted with DCM (20 mL), the combined organic phase was dried over sodium sulfate and the solvent was removed under reduced pressure to yield **3-G** as a colourless solid (Yield: 1975 mg, 2.86 mmol, 95,7%).

MF: $C_{30}H_{47}N_7O_8Si_2$ **MW:** 689.92. *R*F: 0.26 (SiO_2, EA).

LCMS (neg., ACN) (*m/z*): Calculated for $[C_{30}H_{46}N_7O_8Si_2]^-$: 688.29, found: 688.30.

^{1}H-NMR (400 MHz, $CDCl_3$, 298 K, COSY): *δ*/ppm = 12.13 (s, 1H, H-1), 10.11 (s, 1H, *H*N-2), 8.29 – 8.24 (m, 1H, H-2''), 7.92 (s, 1H, H-8), 7.40 (d, *J* = 1.5 Hz, 1H, H-5''), 7.14 (d, *J* = 1.5 Hz, 1H, H-4''), 6.10 (s, 1H, H-1'), 5.72 (d, *J* = 4.8 Hz, 1H, H-2'), 4.70 (dd, *J* = 9.0 Hz, *J* = 4.8 Hz, 1H, H-3'), 4.22 (dd, *J* = 13.3 Hz, *J* = 2.3 Hz, 1H, H-5'), 4.12 (dt, *J* = 9.0 Hz, *J* = 2.6 Hz, 1H, H-4'), 4.03 (dd, *J* = 13.3 Hz, *J* = 2.9 Hz, 1H, H-5'), 2.65 (hept, *J* = 6.1 Hz, 1H, CH^{Ibu}), 1.26 (d, *J* = 6.9 Hz, 3H, CH_3^{Ibu}), 1.22 (d, *J* = 6.9 Hz, 3H, CH_3^{Ibu}), 1.08 – 0.95 (m, 28H, TIPDS).

^{13}C-NMR (101 MHz, $CDCl_3$, 298 K, HSQC, HMBC, DEPT): *δ*/ppm = 179.05 (C=O^{Ibu}), 155.51 (C-6), 148.45 (C-2), 147.35 (C-4), 147.16 (C=$O^{Carbamate}$), 137.31 (C-2''), 136.13 (C-8), 130.68 (C-4''), 121.97

(C-5), 117.44 (C-5''), 86.60 (C-1'), 82.40 (C-4'), 79.89 (C-2'), 68.92 (C-3'), 60.12 (C-5'), 36.43 (CH^{Ibu}), 19.10 (CH_3^{Ibu}), 19.01 (CH_3^{Ibu}), 17.48 (CH_3^{TIPDS}), 17.40 (CH_3^{TIPDS}), 17.33 (2xCH_3^{TIPDS}), 16.90 (2xCH_3^{TIPDS}), 16.69 (2xCH_3^{TIPDS}), 13.44 (CH^{TIPDS}), 12.98 (CH^{TIPDS}), 12.92 (CH^{TIPDS}), 12.68 (CH^{TIPDS}).

Compound 4-G

(2'-O-((2'',3''-Dihydroxypropyl) carbamoyl)-3',5'-O-(1,1,3,3-tetraisopropyldisilox-1,3-diyl) guanosine (iBu))

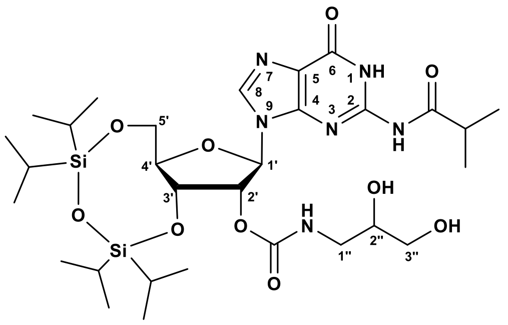

Aminoglycerol (176 mg, 1.93 mmol, 1.9 eq.) was coevaporated with dry pyridine (4x1 mL) and dissolved in dry pyridine (11 mL) under argon atmosphere. A molecular sieve (pore size 4 Å) and DIPEA (393 mg, 3.04 mmol, 3.0 eq.) was added. Compound **3-G** (700 mg, 1.01 mmol, 1.0 eq.) was dissolved separately in dry pyridine (2 mL) and added to the reaction mixture. After stirring for 5 h, the solvent was removed under reduced pressure, the residue was diluted with DCM (100 mL) and washed with a saturated solutions of sodium bicarbonate (2x40 mL) and Brine (40 mL). The organic layer was dried over sodium sulfate and the solvent was removed under reduced pressure. The residue was purified *via* column chromatography on silica (DCM:MeOH, 32:1 → 9:1) to yield **4-G** as a colourless foam (Yield: 408 mg,0.57 mmol, 56.7%).

MF: $C_{30}H_{52}N_6O_{10}Si_2$ **MW:** 712.95. R_F: 0.55 (SiO_2, DCM:MeOH, 9:1).

LCMS (neg., ACN) (*m/z*): Calculated for $[C_{30}H_{51}N_6O_{10}Si_2]^-$: 711.32, found: 711.30.

^{1}H-NMR (400 MHz, $CDCl_3$, 298 K, COSY): δ/ppm = 12.23 (s, 1H, H-1), 10.56 (s, 1H, *H*N-2), 8.00 (d, J = 2.6 Hz, 1H, H-8), 5.77 (d, J = 15.5 Hz, 1H, H-1'), 5.33 – 5.24 (m, 2H, H-2', *H*N-1''), 4.56 – 4.45 (m,

2H, H-3’, H-2’’), 4.26 – 4.19 (m, 2H, H-5’, H-3’’), 4.07 – 3.96 (m, 3H, H-4’, H-5’, H-3’’), 3.81 – 3.57 (m, 2H, H-1’’), 2.68 (hept, J = 6.7 Hz, 1H, CH^{Ibu}), 1.26 (s, 3H, CH_3^{Ibu}), 1.24 (s, 3H, CH_3^{Ibu}), 1.12 – 0.89 (m).

^{13}C-NMR (101 MHz, DMSO-d_6, 298 K, HSQC, HMBC, DEPT): δ/ppm = 180.18 (C=O^{Ibu}), 154.92 (C=O^{Carbamate}), 154.84 (C-6), 148.51 (C-4), 148.18 (C-2), 136.86 (C-8), 120.25 (C-5), 85.20 (C-1’), 82.27 (C-4’), 74.95 (C-2’), 70.54 (C-2’’), 69.73 (C-3’), 63.88 (C-3’’), 61.30 (C-5’), 44.07 (C-1’’), 34.80 (CH^{Ibu}), 18.88 (CH$_3^{Ibu}$), 17.36 (CH$_3^{TIPDS}$), 17.25 (CH$_3^{TIPDS}$), 17.20 (2xCH$_3^{TIPDS}$), 16.83 (2xCH$_3^{TIPDS}$), 16.75 (2xCH$_3^{TIPDS}$), 12.68 (CH^{TIPDS}), 12.49 (CH^{TIPDS}), 12.26 (CH^{TIPDS}), 12.10 (CH^{TIPDS}).

Compound 5-G

(2’-O-((2’’,3’’-Diacetoxypropyl) carbamoyl)-3',5'-O-(1,1,3,3-tetraisopropyldisilox-1,3-diyl) guanosine (iBu))

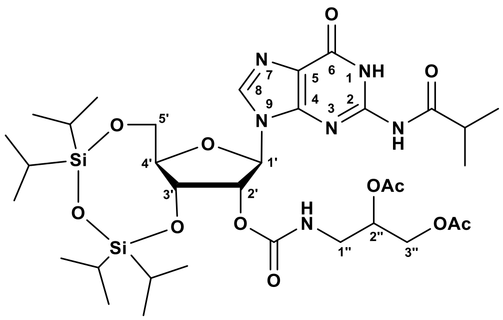

Compound **4-G** (1114 mg, 1.56 mmol, 1.0 eq.) was dissolved in dry pyridine (15 mL). DMAP (1.9 mg, 0.02 mmol, 0.01 eq.) and acetic anhydride (1080 mg, 10.58 mmol, 6.8 eq.) were added to the solution. After stirring the reaction mixture for 67 h, the solvent was removed under reduced pressure, the residue dissolved in EA (50 mL) and washed with a saturated solutions of sodium bicarbonate (2x20 mL) and Brine (20 mL). The aqueous phase was then extracted with EA (20 mL). The combined organic phase was dried over sodium sulfate and the solvent was removed under reduced pressure. The residue was purified *via* column chromatography on silica (DCM:MeOH, 1:0 → 19:1) to yield **5-G** as a colourless foam (Yield: 1224 mg, 1.54 mmol, 98.7%).

MF: $C_{34}H_{56}N_6O_{12}Si_2$ **MW:** 797.02. R_F: 0.78 (SiO_2, DCM:MeOH, 9:1).

LCMS (neg., ACN) (*m/z*): Calculated for $[C_{34}H_{55}N_6O_{12}Si_2]^-$: 795.34, found: 795.30.

^{1}H-NMR (400 MHz, $CDCl_3$, 298 K, COSY): δ/ppm = 12.16 – 12.05 (m, 1H, H-1), 9.67 – 9.57 (m, 1H, *H*N-2), 7.98 – 7.90 (m, 1H, H-8), 5.88 – 5.80 (m, 1H, H-1’), 5.39 (t, *J* = 5.2 Hz, 1H, H-2’), 5.36 – 5.28 (m, 1H, *H*N-1’’), 5.14 (p, *J* = 5.0 Hz, 1H, H-2’’), 4.63 – 4.46 (m, 1H, H-3’), 4.29 (ddd, *J* = 12.5 Hz, *J* = 8.4 Hz, *J* = 4.3 Hz, 1H, H-3’’), 4.24 – 4.19 (m, 1H, H-3’’), 4.18 (dd, *J* = 12.1 Hz, *J* = 5.7 Hz, 1H, H-5’), 4.06 – 3.97 (m, 2H, H-4’, H-5’), 3.74 – 3.57 (m, 1H, H-1’’), 3.36 – 3.15 (m, 1H, H-1’’), 2.64 (hept, *J* = 6.9 Hz, 1H, CH^{Ibu}), 2.15 – 2.06 (m, 6H, CH_3 (AcO-2’’), CH_3 (AcO-3’’)), 1.25 – 1.19 (m, 6H, 2xCH_3^{Ibu}), 1.09 – 0.96 (m, 28H, TIPDS).

^{13}C-NMR (101 MHz, $CDCl_3$, 298 K, HSQC, HMBC, DEPT): δ/ppm = 179.18 (C=O^{Ibu}), 171.28 (*C*=O (AcO-3’’)), 170.33 (*C*=O (AcO-2’’)), 155.72 (C-6), 155.01 (C=$O^{Carbamate}$), 148.15 (C-4), 147.47 (C-2), 135.97 (C-8), 121.89 (C-5), 87.82 (C-1’), 82.03 (C-4’), 77.36 (C-2’), 70.11 (C-2’’), 68.02 (C-3’), 62.57 (C-3’’), 60.15 (C-5’), 41.26 (C-1’’), 36.21 (CH^{Ibu}), 21.00 (CH_3 (AcO-3’’)), 20.88 (CH_3 (AcO-2’’)), 19.31 (CH_3^{Ibu}), 19.24 (CH_3^{Ibu}), 17.57 (CH_3^{TIPDS}), 17.42 (CH_3^{TIPDS}), 17.40 (CH_3^{TIPDS}), 17.35 (CH_3^{TIPDS}), 17.04 (CH_3^{TIPDS}), 16.97 (2xCH_3^{TIPDS}), 16.93 (CH_3^{TIPDS}), 13.47 (CH^{TIPDS}), 13.04 (2xCH^{TIPDS}), 12.64 (CH^{TIPDS}).

Compound 6-G

(2’-O-((2’’,3’’-Diacetoxypropyl) carbamoyl) guanosine(iBu))

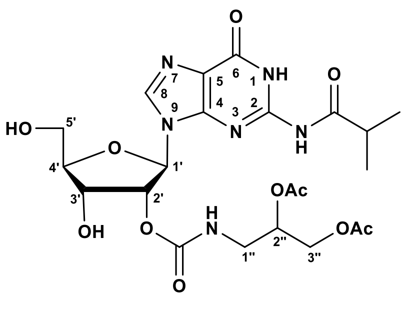

Compound **5-G** (1224 mg, 1.54 mmol, 1.0 eq.) was dissolved in dry pyridine (16 mL) and triethylamine trihydrofluoride (743 mg, 4.61 mmol, 3.0 eq.) was added to the solution. After stirring for 16 h, the solvent

was removed under reduced pressure and the residue was purified *via* column chromatography on silica (DCM:MeOH, 19:1 → 11:1) to yield **6-G** as a colourless foam (Yield: 843 mg, 1.52 mmol, 99.0%).

MF: $C_{22}H_{30}N_6O_{11}$ **MW:** 554.51. R_F: 0.51 (SiO_2, DCM:MeOH, 9:1).

LCMS (pos., ACN) (*m/z*): Calculated for $[C_{22}H_{30}N_6O_{11}H]^+$: 555.21, found: 555.20.

^{1}**H-NMR** (400 MHz, DMSO-d_6, 298 K, COSY): δ/ppm = 12.10 (s, 1H, H-1), 11.64 (d, J = 1.8 Hz, 1H, *H*N-2), 8.29 (d, J = 1.6 Hz, 1H, H-8), 7.49 (t, J = 6.0 Hz, 1H, *H*N-1''), 6.00 (d, J = 6.1 Hz, 1H, H-1'), 5.56 (dd, J = 17.6 Hz, J = 4.8 Hz, 1H, *H*O-3'), 5.40 (dt, J = 7.6 Hz, J = 5.6 Hz, 1H, H-2'), 5.12 (t, J = 5.2 Hz, 1H, *H*O-5'), 4.91 (pd, J = 5.8 Hz, J = 3.4 Hz, 1H, H-2''), 4.41 (q, J = 4.8 Hz, 1H, H-3'), 4.12 (dt, J = 12.1 Hz, J = 3.9 Hz, 1H, H-3''), 4.02 – 3.96 (m, 1H, H-3''), 3.93 (q, J = 3.7 Hz, 1H, H-4'), 3.69 – 3.63 (m, 1H, H-5'), 3.56 (dt, J = 11.8 Hz, J = 4.7 Hz, 1H, H-5'), 3.22 (dt, J = 15.3 Hz, J = 3.2 Hz, 1H, H-1''), 3.13 (dt, J = 14.1 Hz, J = 6.3 Hz, 1H, H-1''), 2.76 (hept, J = 6.9 Hz, 1H, CH^{Ibu}), 1.98 – 1.93 (m, 6H, CH_3 (AcO-2''), CH_3 (AcO-3'')), 1.13 (s, 3H, CH_3^{Ibu}), 1.11 (s, 3H, CH_3^{Ibu}).

^{13}**C-NMR** (101 MHz, DMSO-d_6, 298 K, HSQC, HMBC, DEPT): δ/ppm = 180.17 (C=O^{Ibu}), 170.16 (C=O (AcO-3'')), 169.87 (C=O (AcO-2'')), 155.28 (C=$O^{Carbamate}$), 154.80 (C-6), 148.77 (C-4), 148.33 (C-2), 137.72 (C-8), 120.13 (C-5), 86.03 (C-4'), 84.09 (C-1'), 75.46 (C-2'), 70.12 (C-2''), 68.89 (C-3'), 62.66 (C-3''), 60.97 (C-5'), 40.39 (C-1''), 34.79 (CH^{Ibu}), 20.77 (CH_3 (AcO-3'')), 20.54 (CH_3 (AcO-2'')), 18.89 (CH_3^{Ibu}), 18.84 (CH_3^{Ibu}).

Compound 7-G

(2'-O-((2'',3''-Diacetoxypropyl) carbamoyl)-5'-O-DMT-guanosine (iBu))

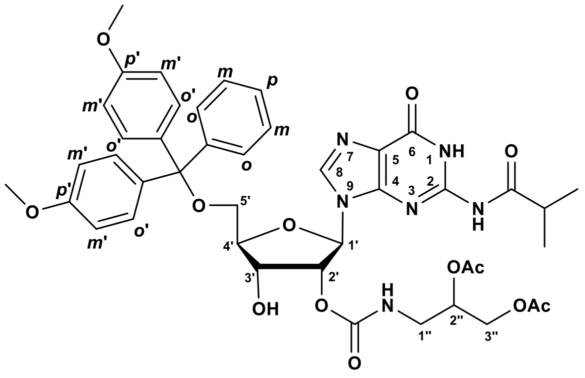

Compound **6-G** (800 mg, 1.44 mmol, 1.0 eq.) was co-evaporated four times with dry pyridine (2 mL), before it was dissolved in dry pyridine (30 mL). DIPEA (1484 mg, 11.48 mmol, 7.8 eq.), a molecular sieve (4 Å) and 4,4'-dimethoxytritylchloride (2500 mg, 7.38 mmol, 5.1 eq.) was added in one portion and the reaction mixture was stirred for 13 h at room temperature under argon atmosphere. The solvent was removed under reduced pressure and the residue was purified via column chromatography on silica (Hex:EA, 3:1 → 0:1, with 1% Et_3N) to yield **7-G** as a colourless foam (Yield: 737 mg, 0.86 mmol, 59.7%).

MF: $C_{43}H_{48}N_6O_{13}$ **MW:** 856.89. R_F: 0.40 (SiO_2, DCM:MeOH, 19:1).

LCMS (pos., ACN) (*m/z*): Calculated for $[C_{43}H_{48}N_6O_{13}H]^+$: 857.34, found: 857.30.

HRMS (ESI-MS, pos. mode): calculated for $[C_{43}H_{48}N_6O_{13}H]^+$: 857.3352, found: 857.3326.

^{1}H-NMR (400 MHz, DMSO-d_6, 298 K, COSY): δ/ppm = 10.69 (s, 1H, H-1), 9.82 (s, 1H, *H*N-2), 8.13 (d, *J* = 5.3 Hz, 1H, H-8), 7.62 (t, *J* = 8.4 Hz, 1H, *H*N-1''), 7.31 (d, *J* = 7.7 Hz, 2H, 2xH-*o*), 7.24 – 7.15 (m, 7H, 4xH-*o*', 2xH-*m*, H-*p*), 6.84 – 6.75 (m, 4H, 4xH-*m*'), 6.04 – 5.99 (m, 1H, H-1'), 5.60 (d, *J* = 6.4 Hz, 1H, *H*O-3'), 5.54 (d, *J* = 3.8 Hz, 1H, H-2'), 4.97 – 4.88 (m, 1H, H-2''), 4.62 – 4.55 (m, 1H, H-3'), 4.12 (d, *J* = 10.3 Hz, 1H, H-3''), 4.08 – 3.97 (m, 2H, H-4', H-3''), 3.72 (s, 3H, CH_3O-*p*'), 3.70 (s, 3H, CH_3O-*p*'), 3.39 – 3.10 (m, 4H, 2xH-5', 2xH-1''), 2.84 – 2.75 (m, 1H, CH^{Ibu}), 2.01 – 1.93 (m, 6H, CH_3 (AcO-2''), CH_3 (AcO-3'')), 1.27 – 1.23 (m, 6H, CH_3^{Ibu}).

^{13}C-NMR (101 MHz, DMSO-d_6, 298 K, HSQC, HMBC, DEPT): δ/ppm = 180.12 (C=O^{Ibu}), 170.17 (C=O (AcO-3'')), 169.90 (C=O (AcO-2'')), 158.04 (2xC-*p*'), 155.44 (C=O^{Carbamate}), 154.82 (C-6), 148.58 (C-2,

C-4), 144.73 (C-*ipso*), 138.00 (C-8), 135.45 (2xC-*ipso*'), 129.76 (4xC-*o*'), 127.75 (2xC-*o*, 2xC-*m*), 126.66 (C-*p*), 120.43 (C-5), 113.07 (4xC-*m*'), 85.55 (C_q^{DMT}), 85.11 (C-1'), 83.84 (C-4'), 75.04 (C-2'), 69.94 (C-2''), 68.88 (C-3'), 63.70 (C-5'), 62.70 (C-3''), 54.97 (2xCH$_3$O-*p*'), 40.29 (C-1''), 34.72 (CH^{Ibu}), 20.80 (CH_3 (AcO-2'')), 20.55 (CH_3 (AcO-3'')), 18.90 (CH_3^{Ibu}), 18.81 (CH_3^{Ibu}).

Compound 8-G

(2'-O-((2'',3''-Diacetoxypropyl) carbamoyl)-5'-O-DMT-3'-O-((2-cyanoethoxy, diisopropyl-amino, phosphanyl) guanosine (iBu))

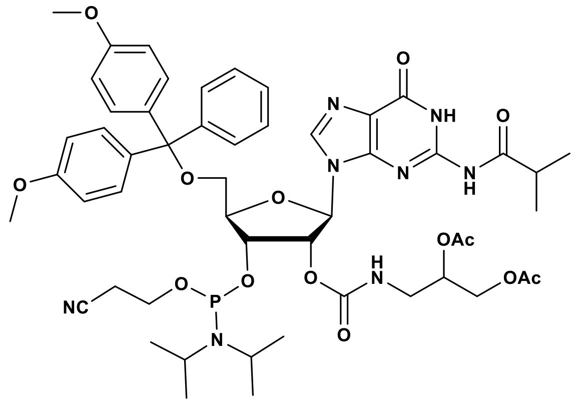

Compound **7-G** (162 mg, 0.19 mmol, 1.0 eq.) was co-evaporated together with dry pyridine (10mL) to remove the traces of water thrice. Under argon atmosphere, **7-G** was dissolved in dry DCM (10 mL), DIPEA (74 mg, 0.57 mmol, 3.0 eq.) was added and 2-Cyanoethyl *N,N*-diisopropylchlorophosphoramidite (91 mg, 0.38 mmol, 2 eq.) was added dropwise over 5 min and reaction mixture was stirred for 3 h at room temperature under argon atmosphere. After removal of the solvent, the residue was purified via column chromatography on silica (Hex:EA, 1:1 → 0:1, with 1% Et_3N) under argon atmosphere. The slightly yellowish foam obtained in this way was dissolved in dry DCM (3 mL) and precipitated with cold hexane (40 mL) while stirring. This procedure was repeated three times and the product was collected by centrifugation and drying in vacuo to yield a colourless foam (Yield: 105 mg, 0.1 mmol, 52.2%).

MF: $C_{52}H_{65}N_8O_{14}P$ **MW:** 1057.1.

^{31}P-NMR (162 MHz, $CDCl_3$, 298 K): δ/ppm = 151.12, 150.04, 150.87 and 150.69.

NMR characterization of the synthesized compounds:

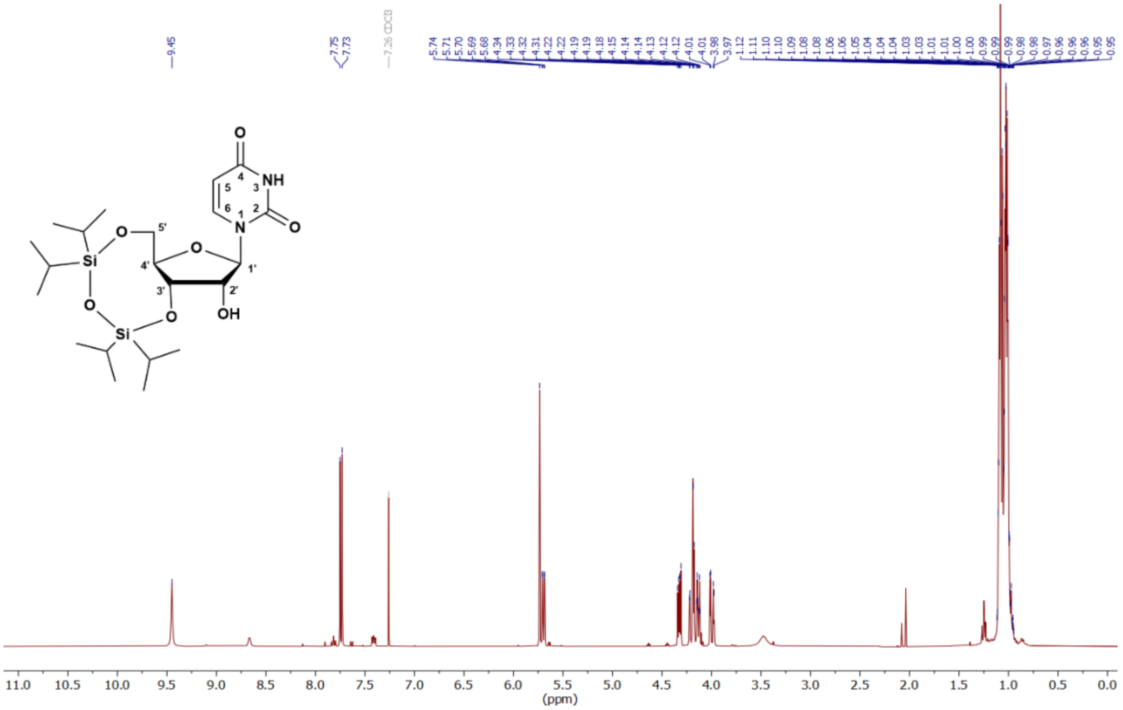

Figure S1.1: ^{1}H NMR spectrum (400 MHz, $CDCl_3$) of compound **2-U**.

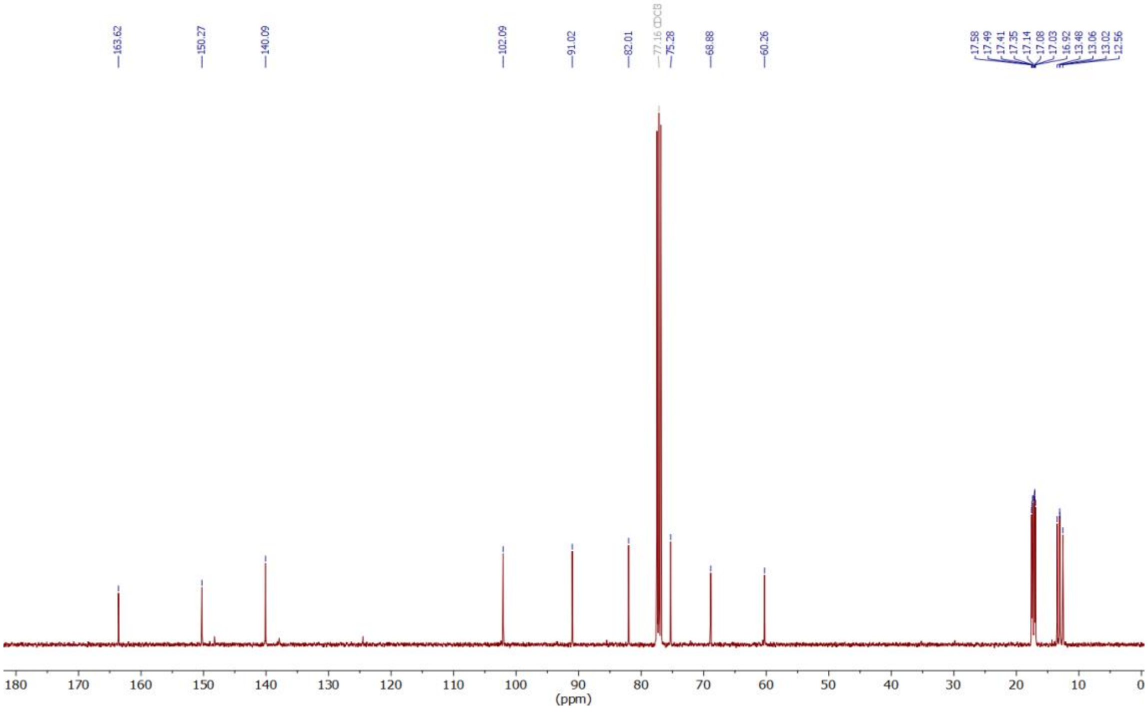

Figure S1.2: ^{13}C NMR spectrum (101 MHz, $CDCl_3$) of compound **2-U**.

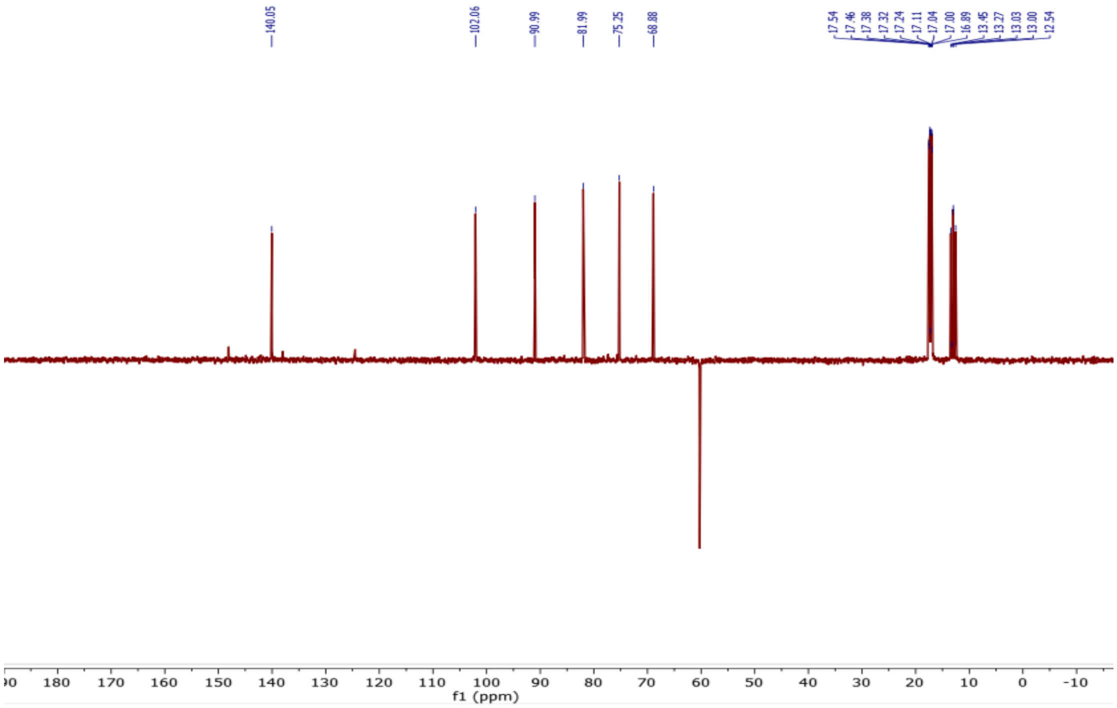

Figure S1.3: ^{13}C DEPT with decoupling NMR spectrum of compound **2-U**.

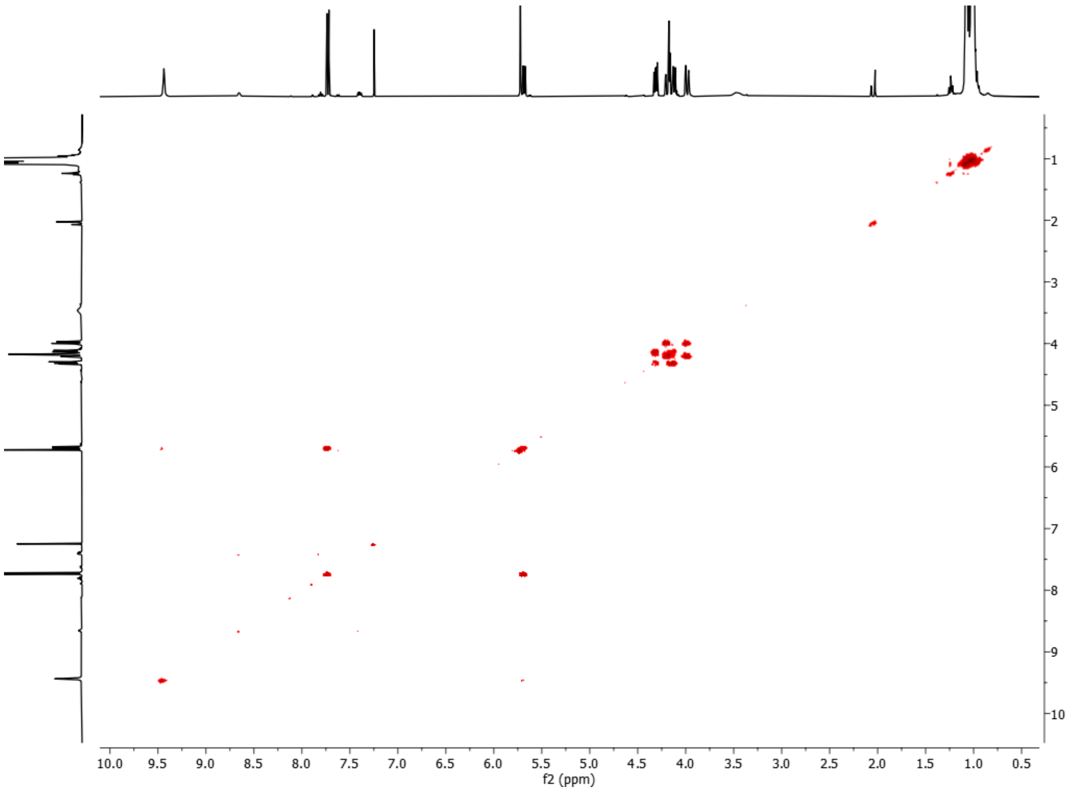

Figure S1.4: gCOSY spectra of compound **2-U**.

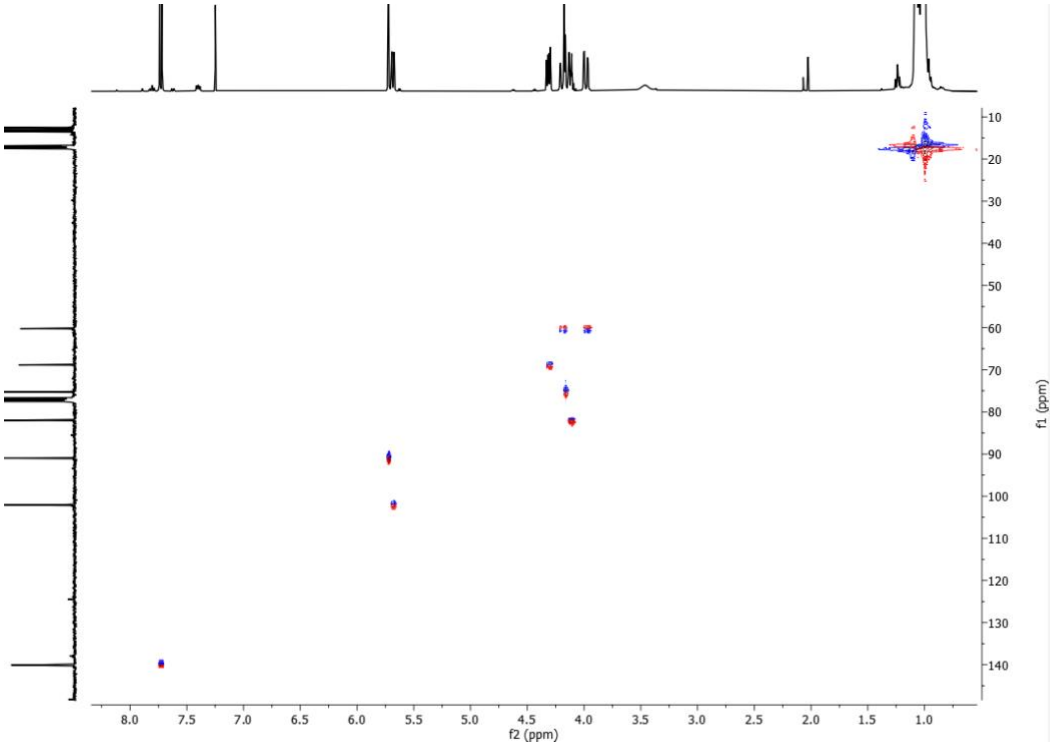

Figure S1.5: HSQC spectra of compound **2-U**.

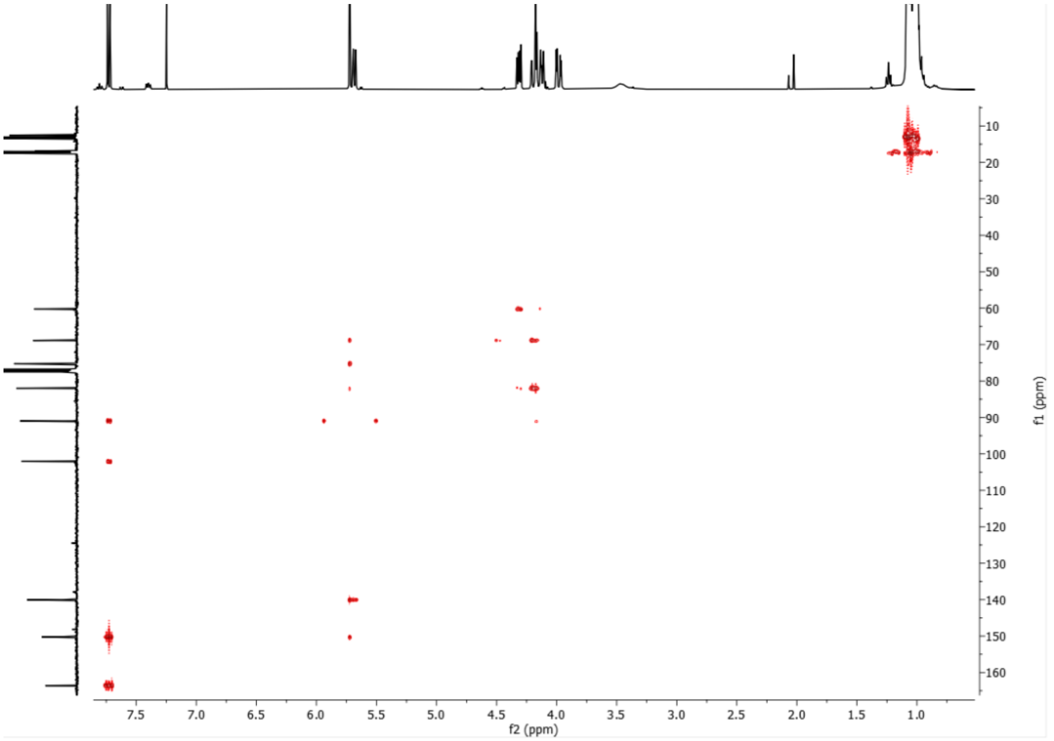

Figure S1.6: HMBC spectra of compound **2-U**.

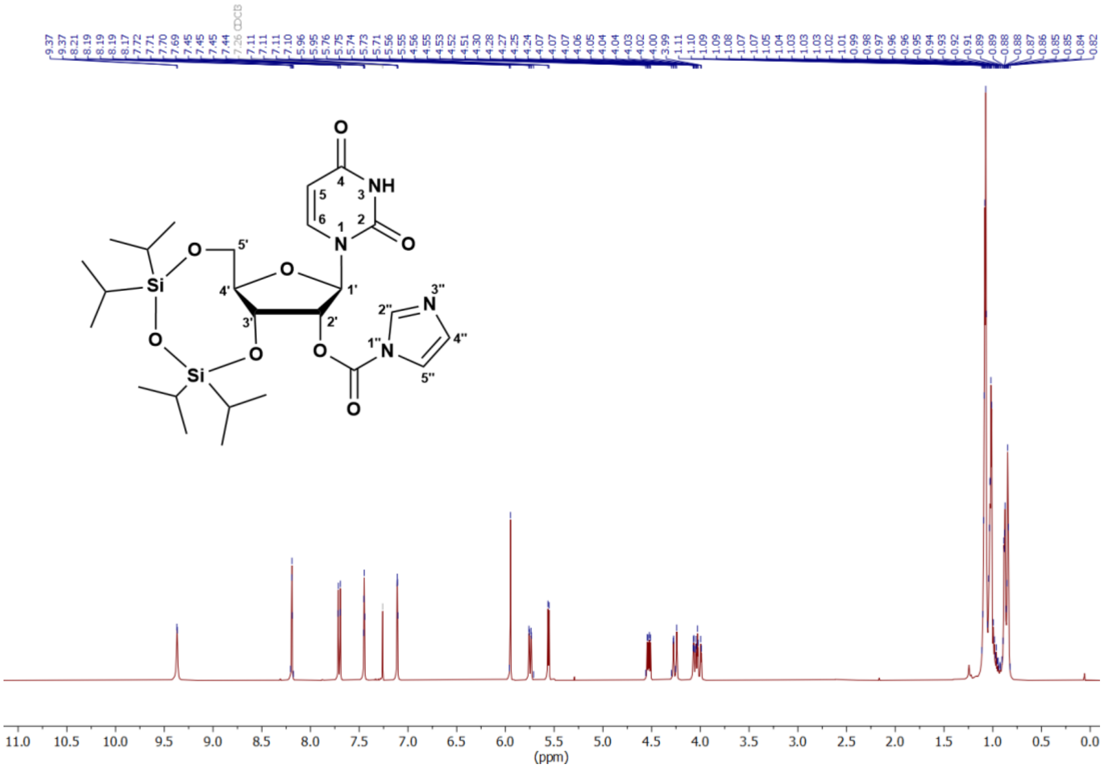

Figure S2.1: ^{1}H NMR spectrum (400 MHz, $CDCl_3$) of compound **3-U**.

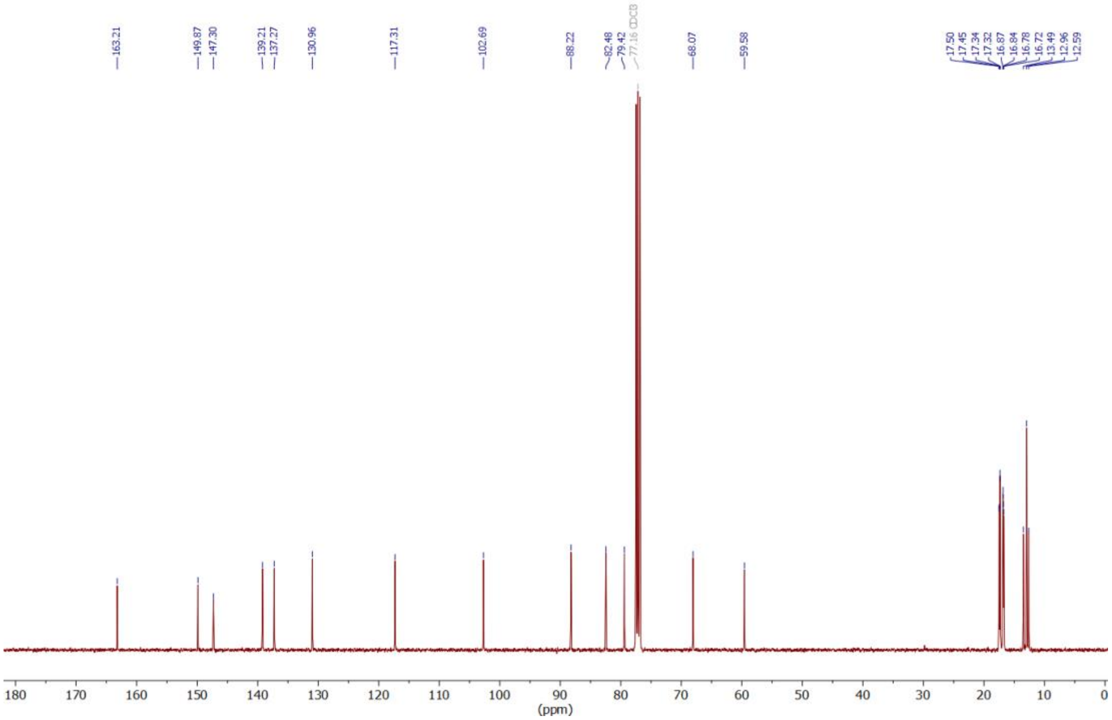

Figure S2.2: ^{13}C NMR spectrum (101 MHz, $CDCl_3$) of compound **3-U**.

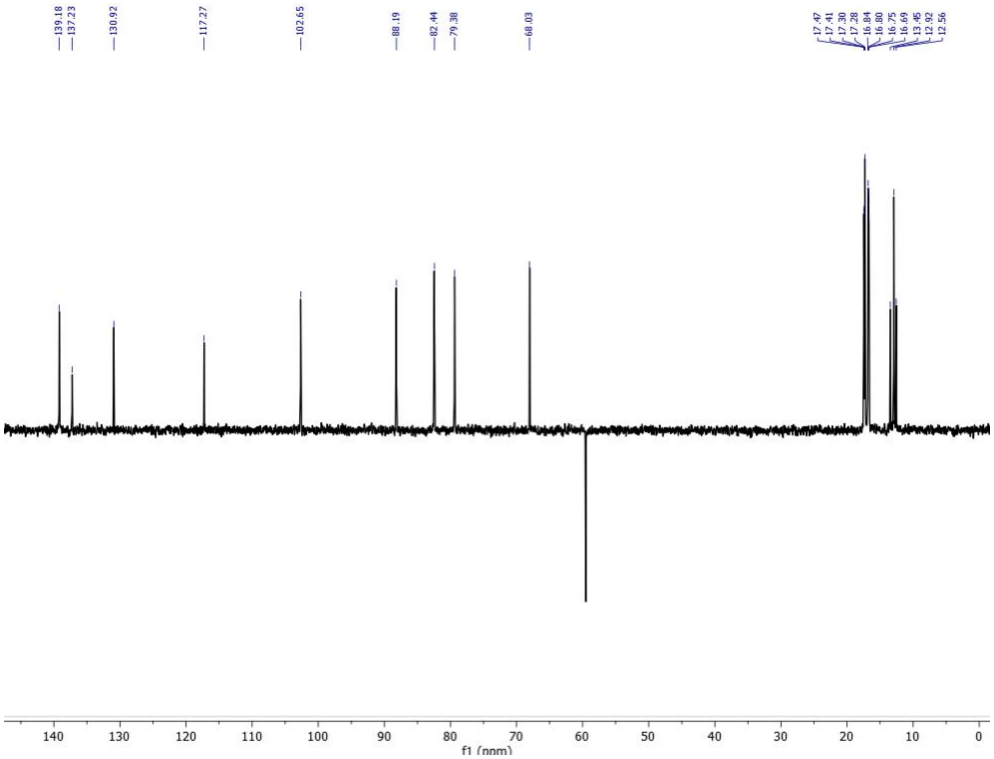

Figure S2.3: ^{13}C DEPT with decoupling NMR spectrum of compound **3-U**.

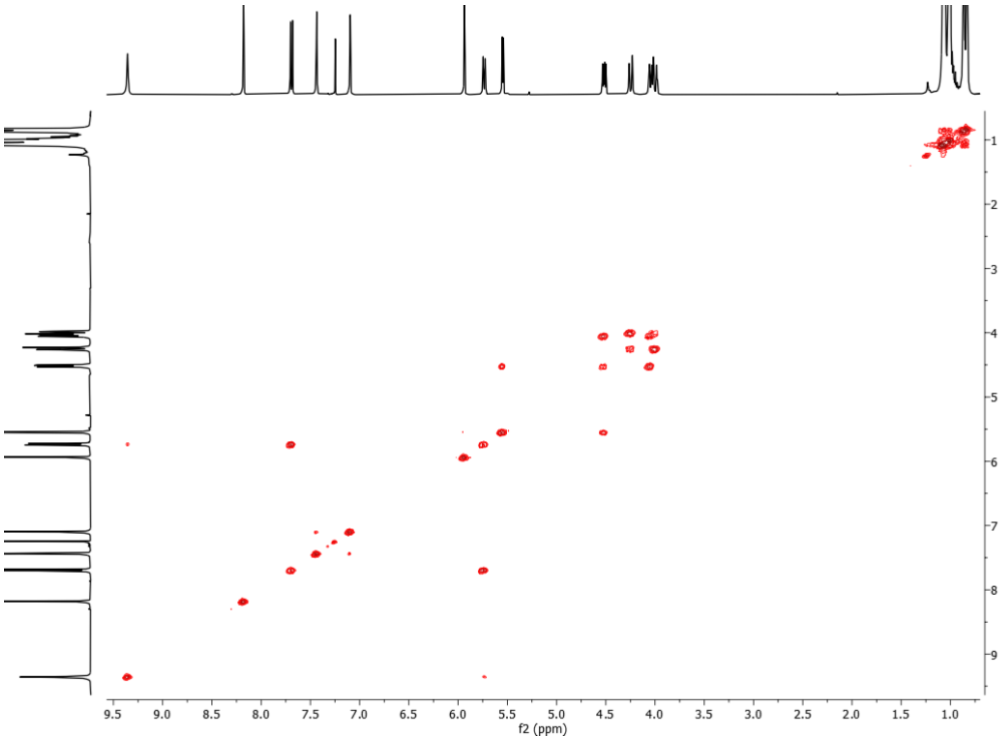

Figure S2.4: gCOSY spectra of compound **3-U**.

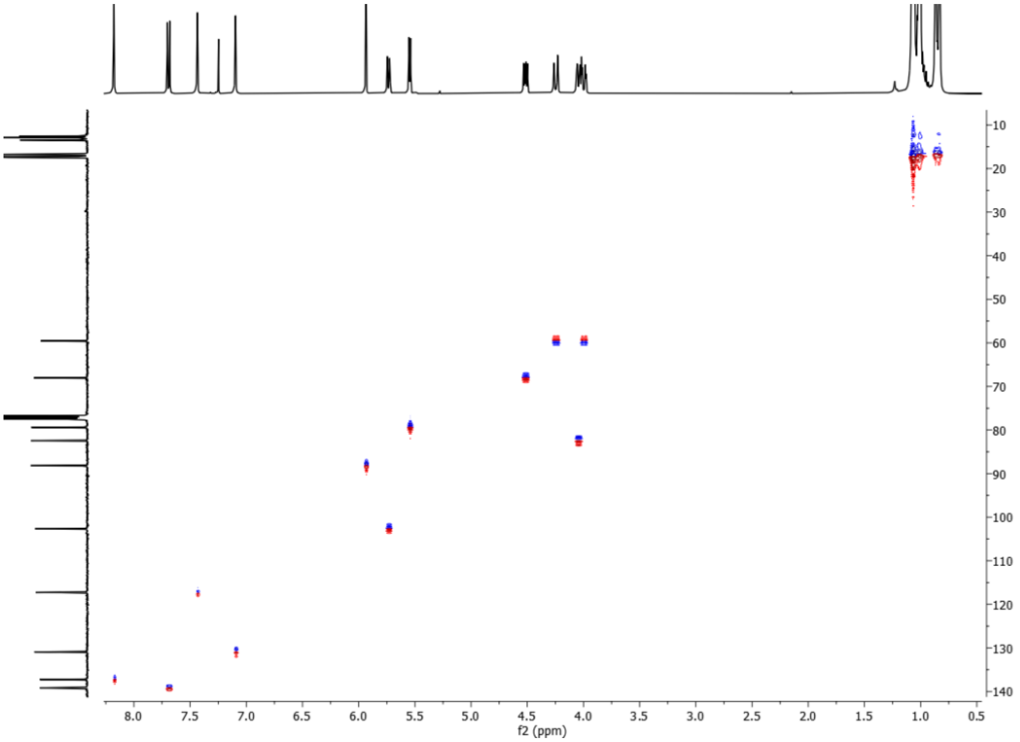

Figure S2.5: HSQC spectra of compound **3-U**.

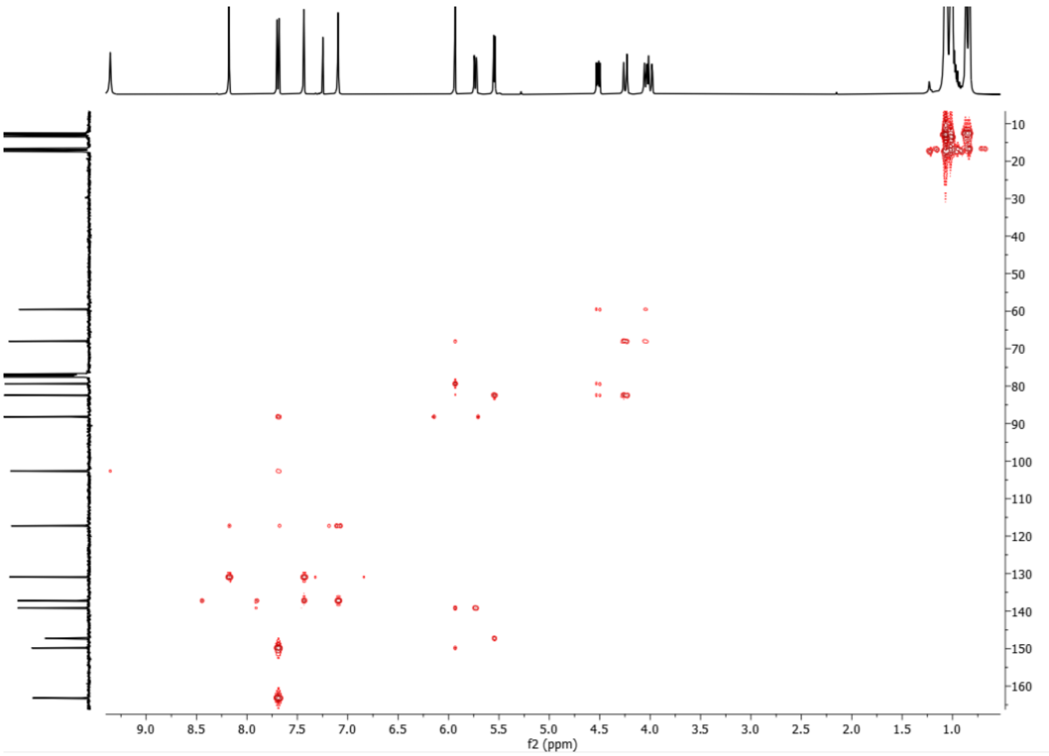

Figure S2.6: HMBC spectra of compound **3-U**.

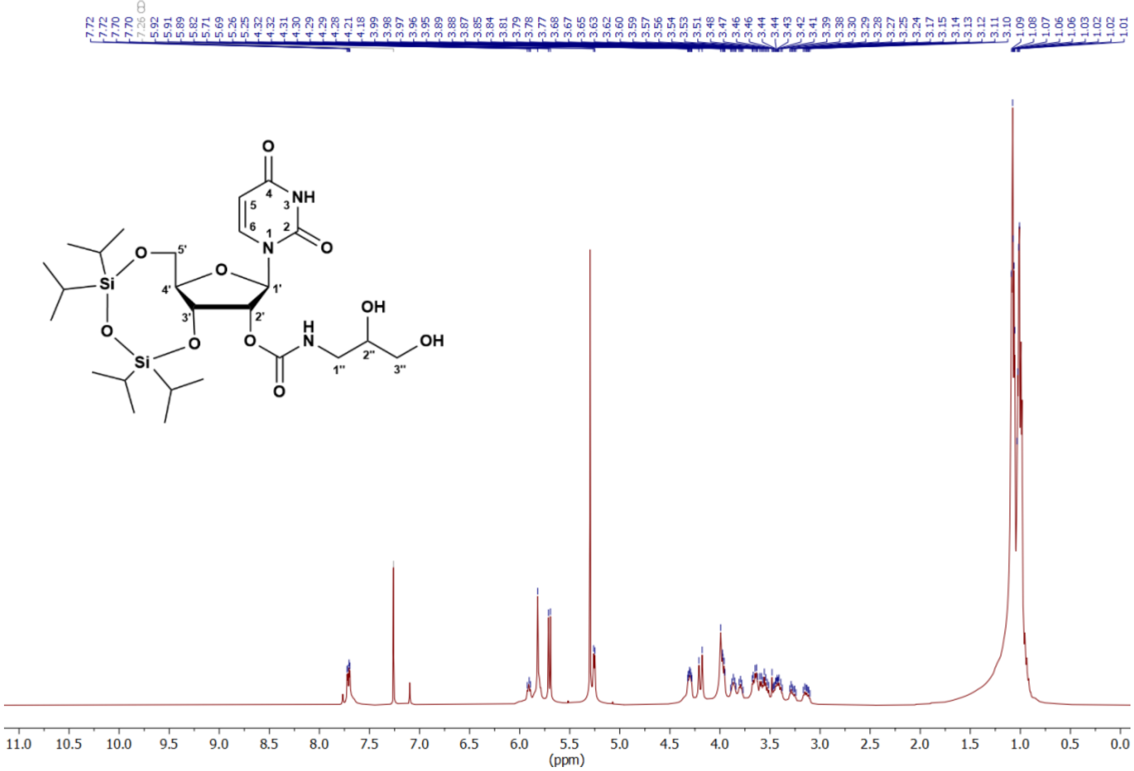

Figure S3.1: ^{1}H NMR spectrum (400 MHz, $CDCl_3$) of compound **4-U**.

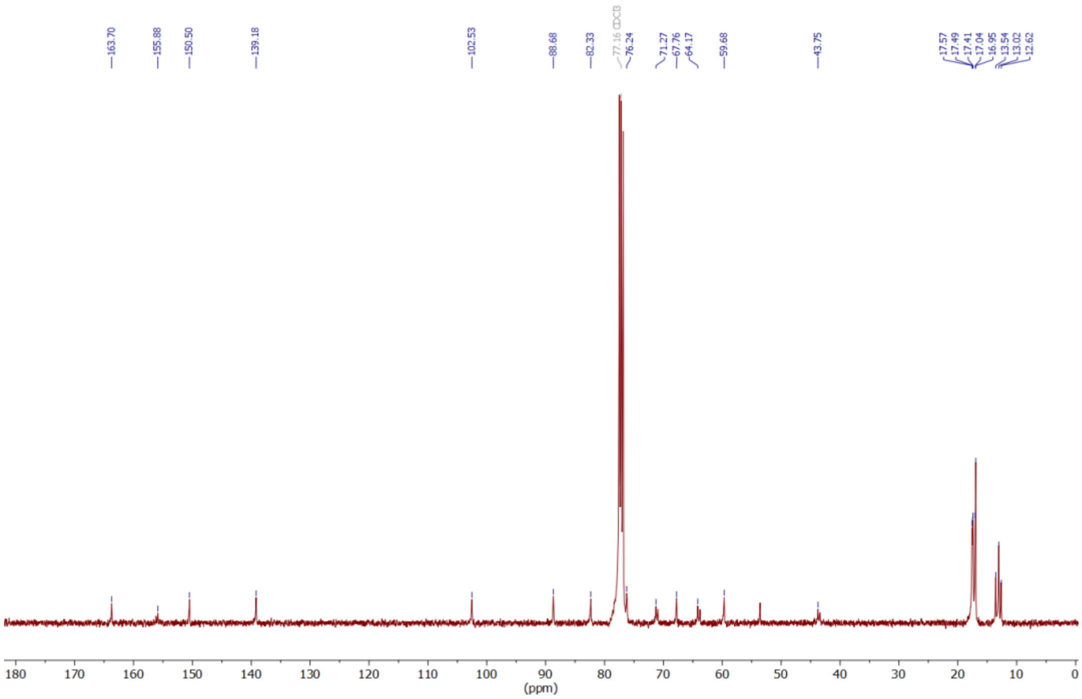

Figure S3.2: ^{13}C NMR spectrum (101 MHz, $CDCl_3$) of compound **4-U**.

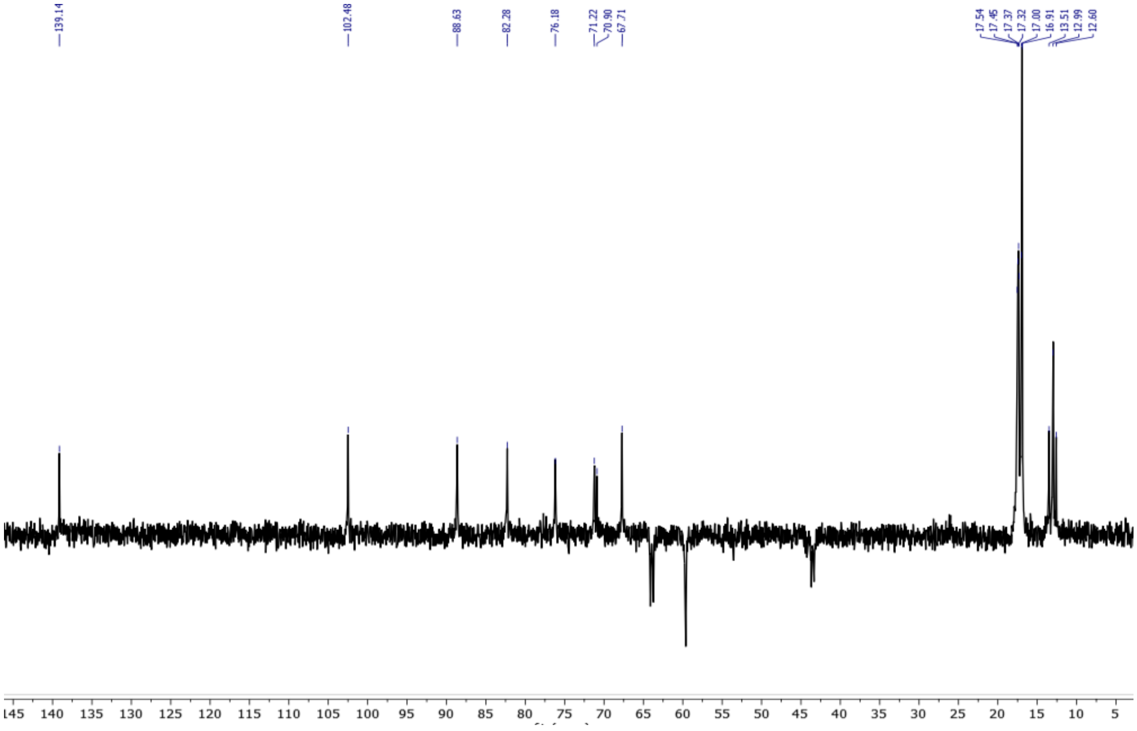

Figure S3.3: ^{13}C DEPT with decoupling NMR spectrum of compound **4-U**.

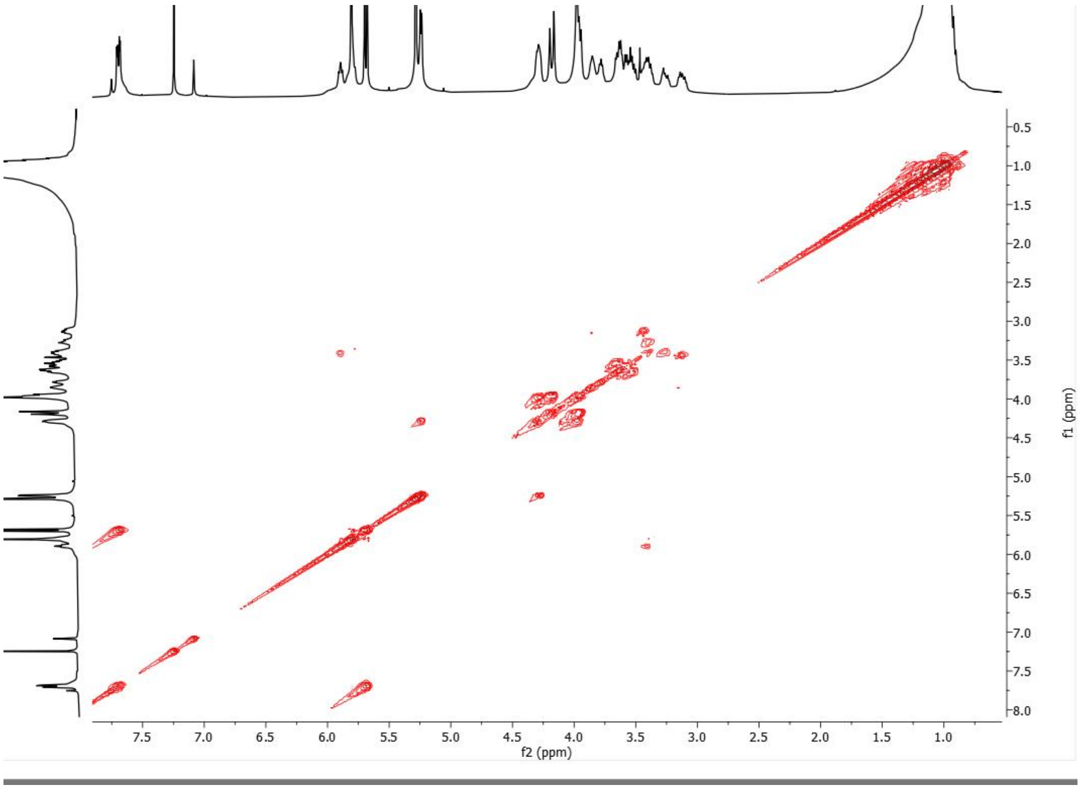

Figure S3.4: gCOSY spectra of compound **4-U**.

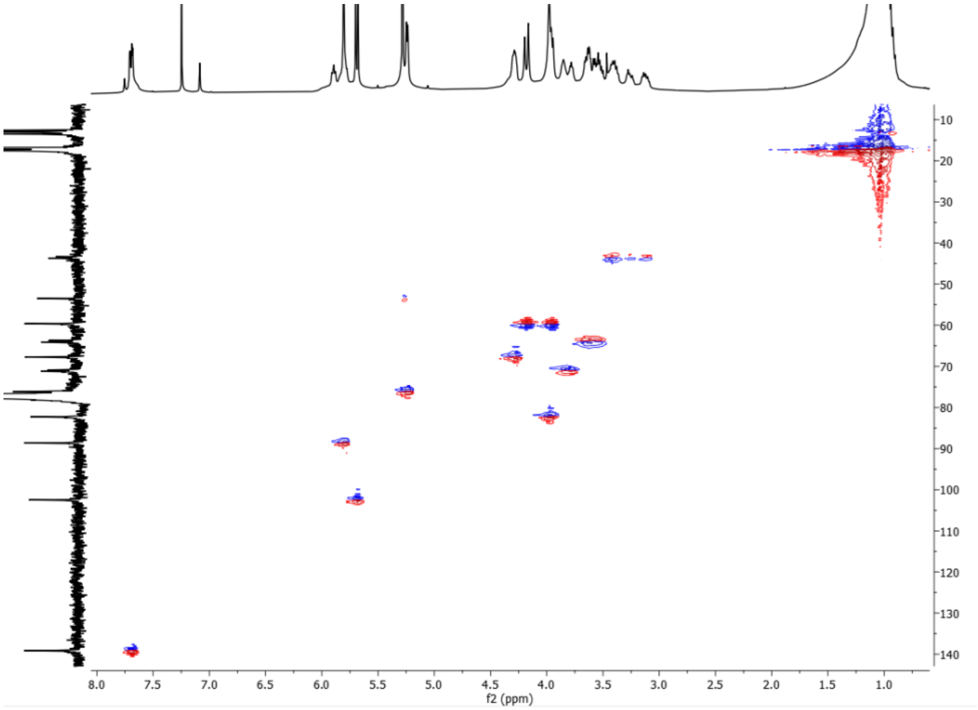

Figure S3.5: HSQC spectra of compound **4-U**.

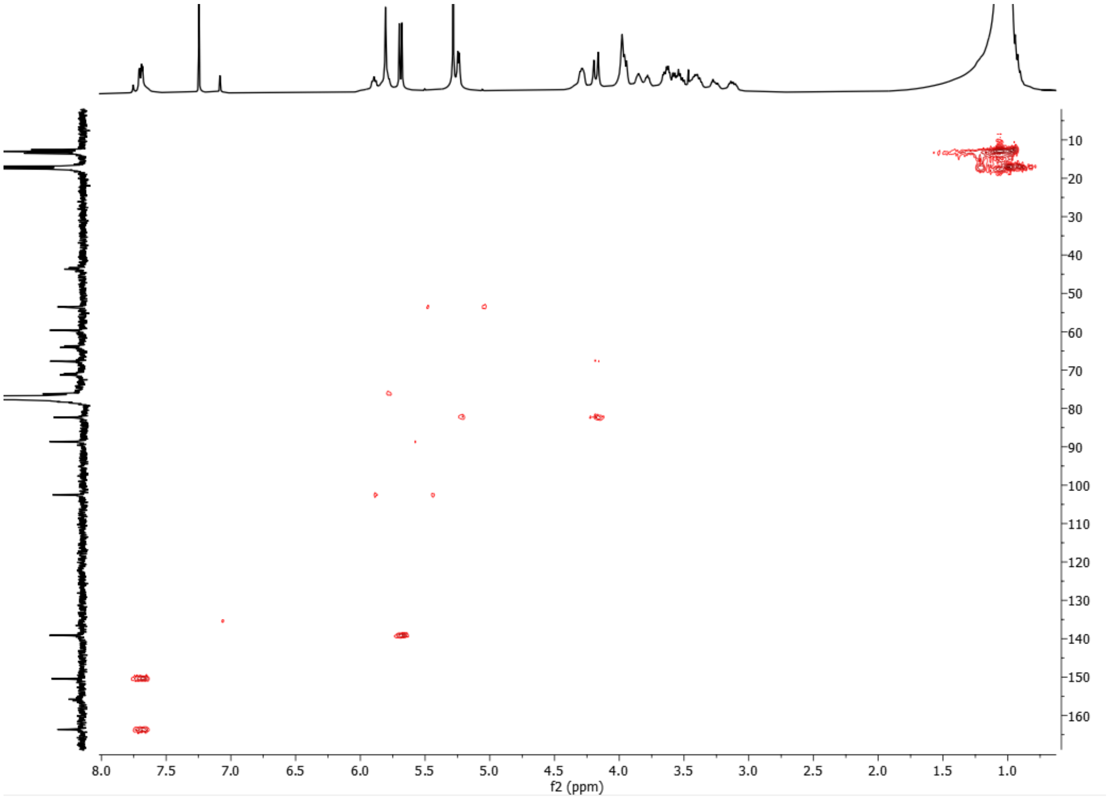

Figure S3.6: HMBC spectra of compound **4-U**.

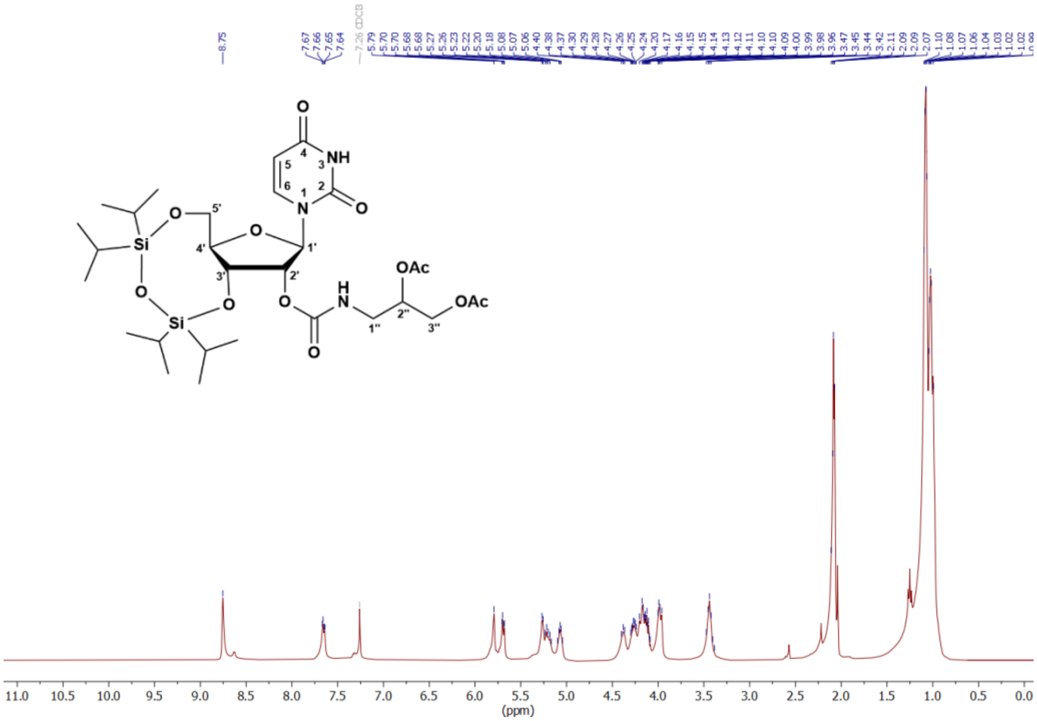

Figure S4.1: ^{1}H NMR spectrum (400 MHz, $CDCl_3$) of compound **5-U**.

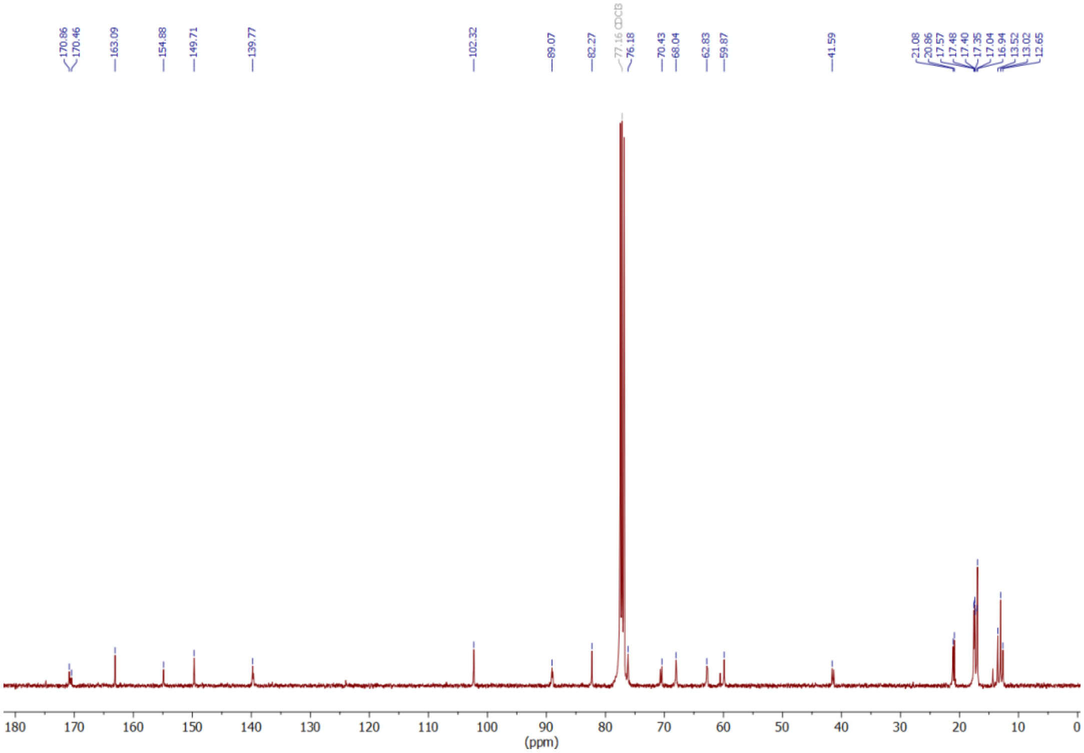

Figure S4.2: ^{13}C NMR spectrum (101 MHz, $CDCl_3$) of compound **5-U**.

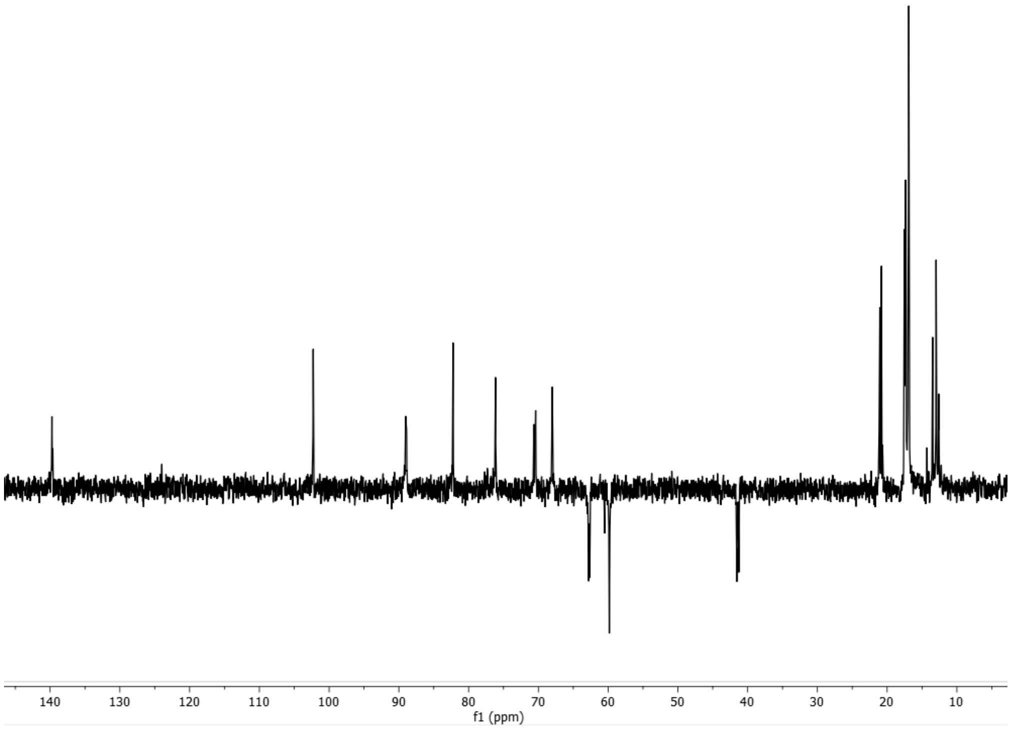

Figure S4.3: ^{13}C DEPT with decoupling NMR spectrum of compound **5-U**.

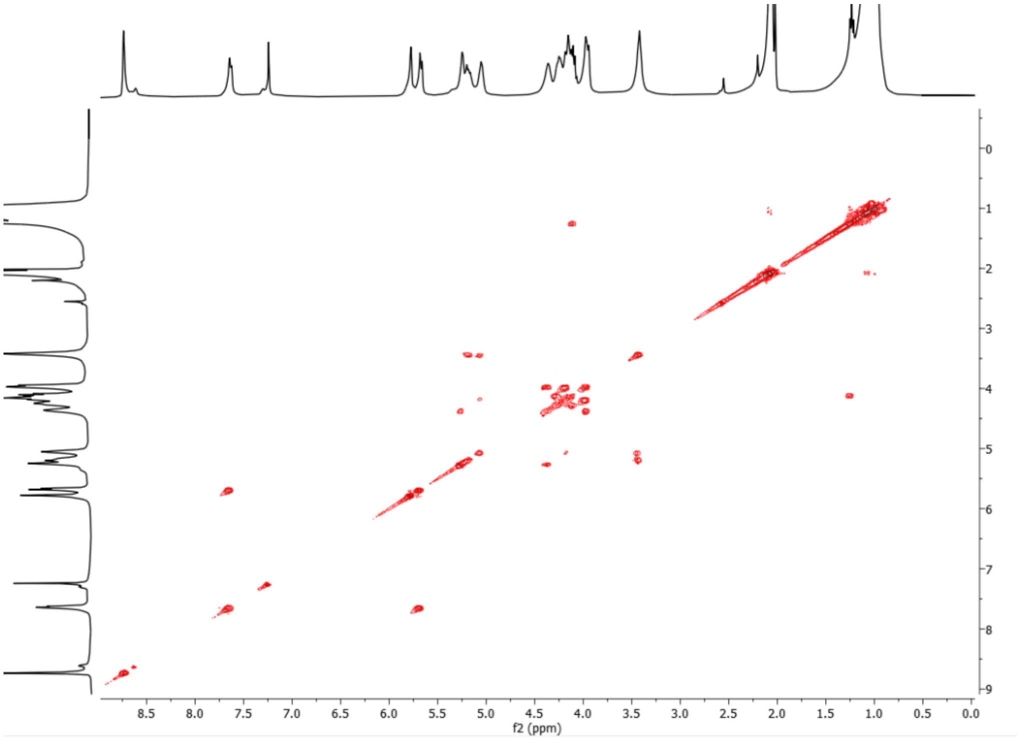

Figure S4.4: gCOSY spectra of compound **5-U**.

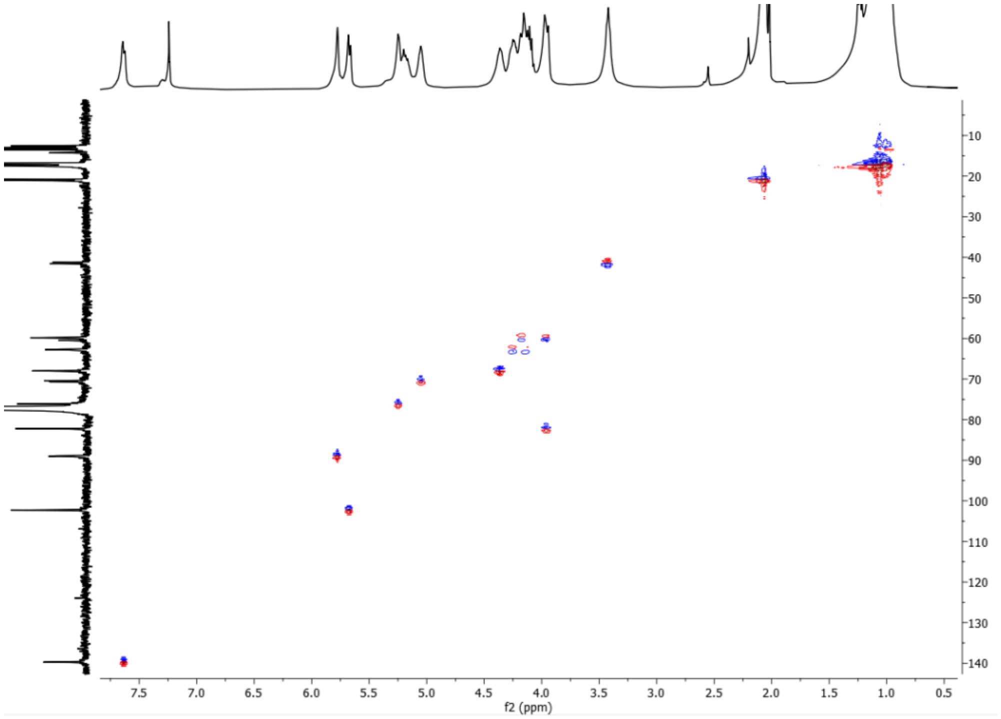

Figure S4.5: HSQC spectra of compound **5-U**.

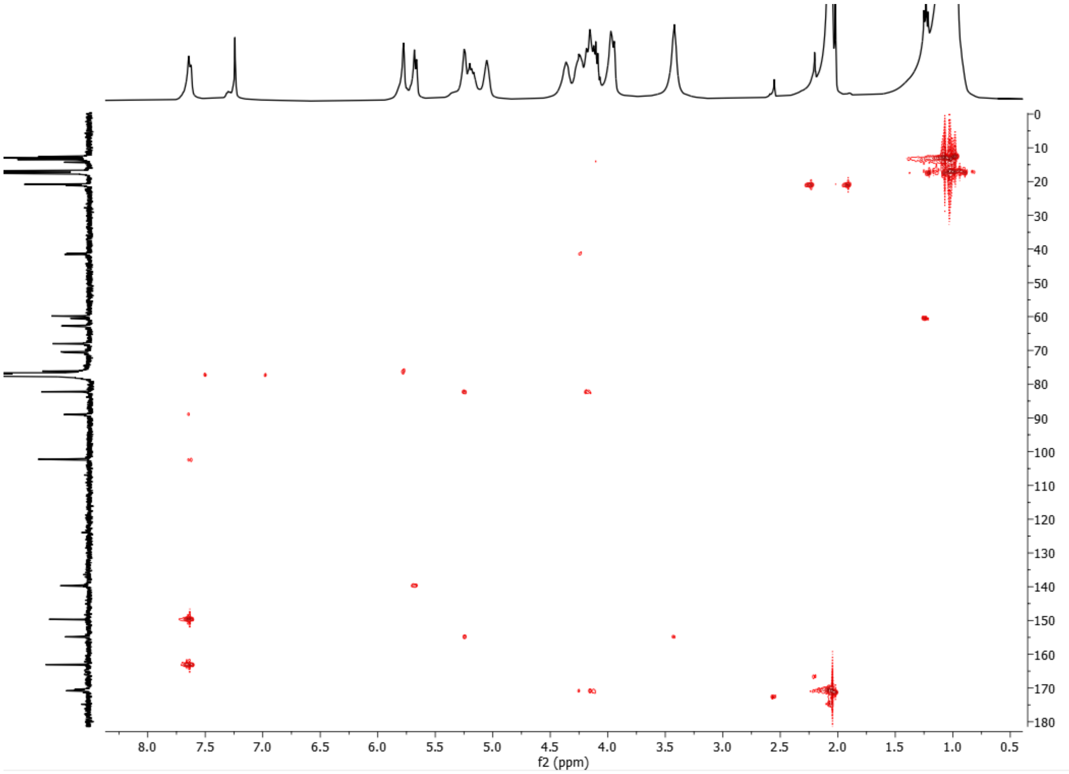

Figure S4.6: HMBC spectra of compound **5-U**.

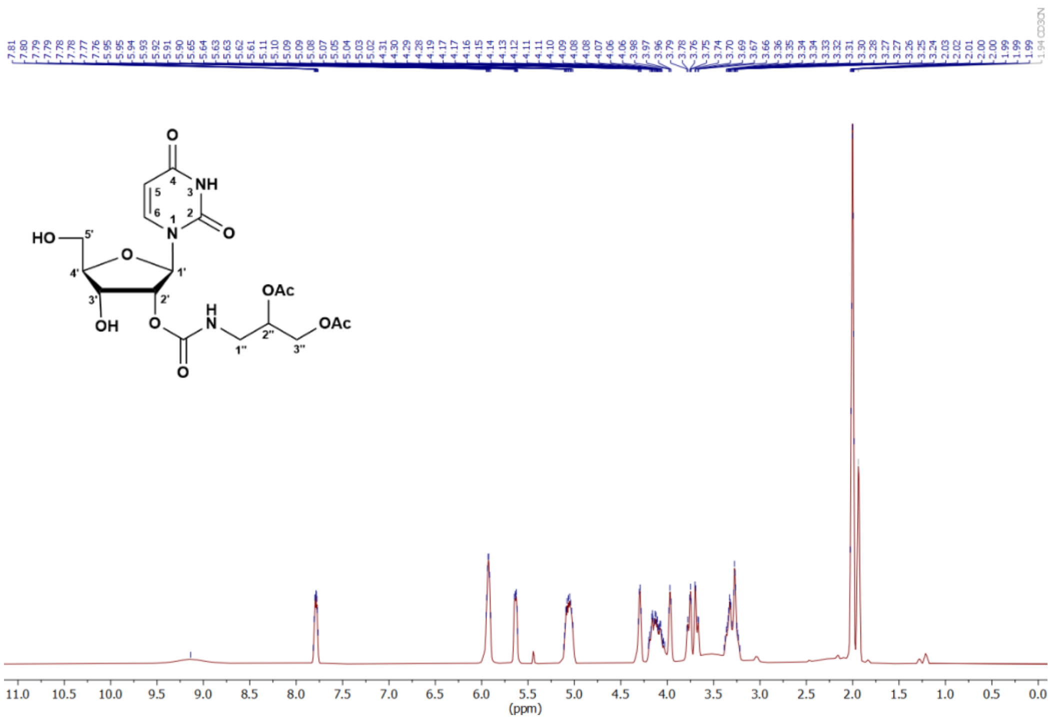

Figure S5.1: ^{1}H NMR spectrum (400 MHz, ACN-d_3) of compound **6-U**.

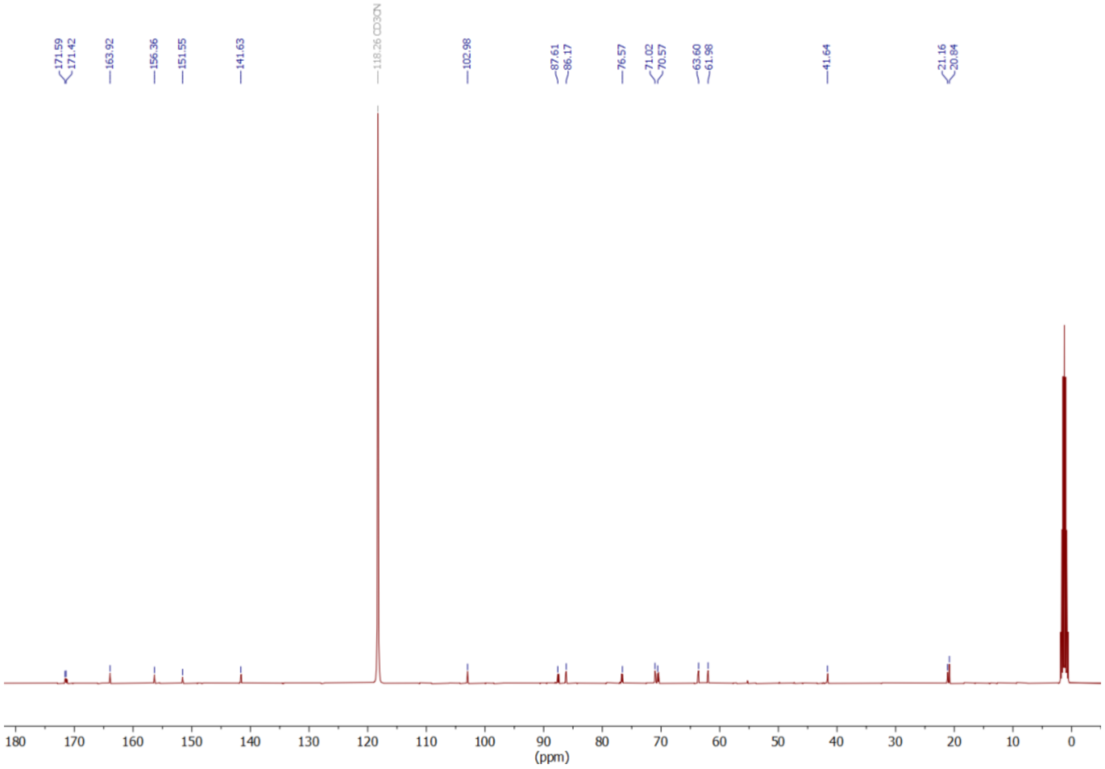

Figure S5.2: ^{13}C NMR spectrum (101 MHz, ACN-d_3) of compound **6-U**.

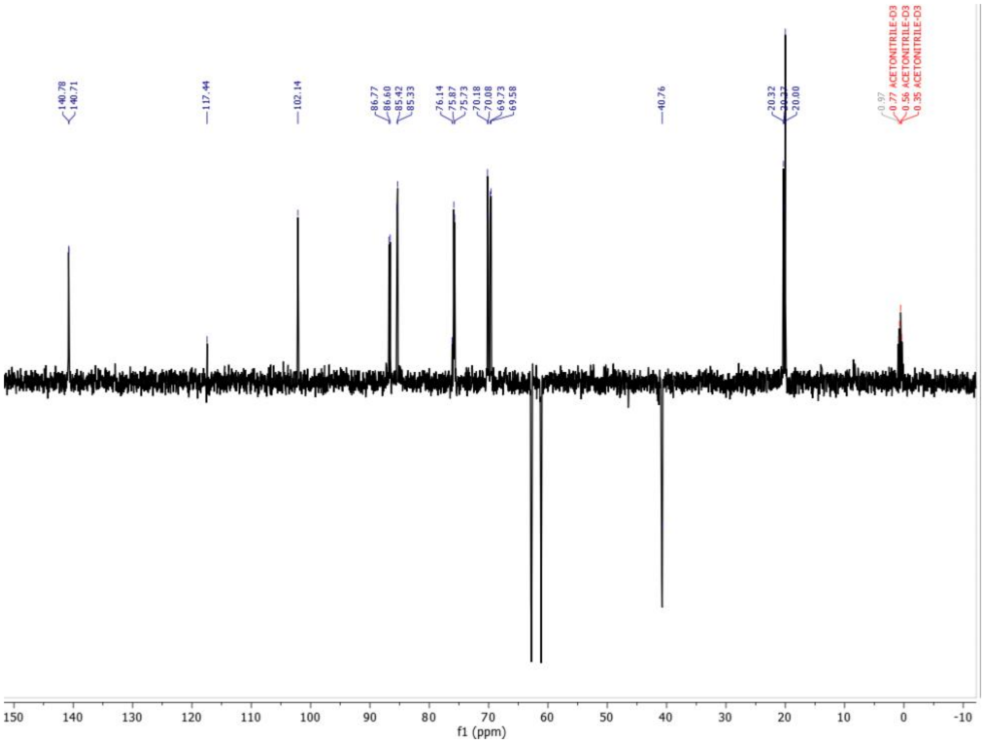

Figure S5.3: ^{13}C DEPT with decoupling NMR spectrum of compound **6-U**.

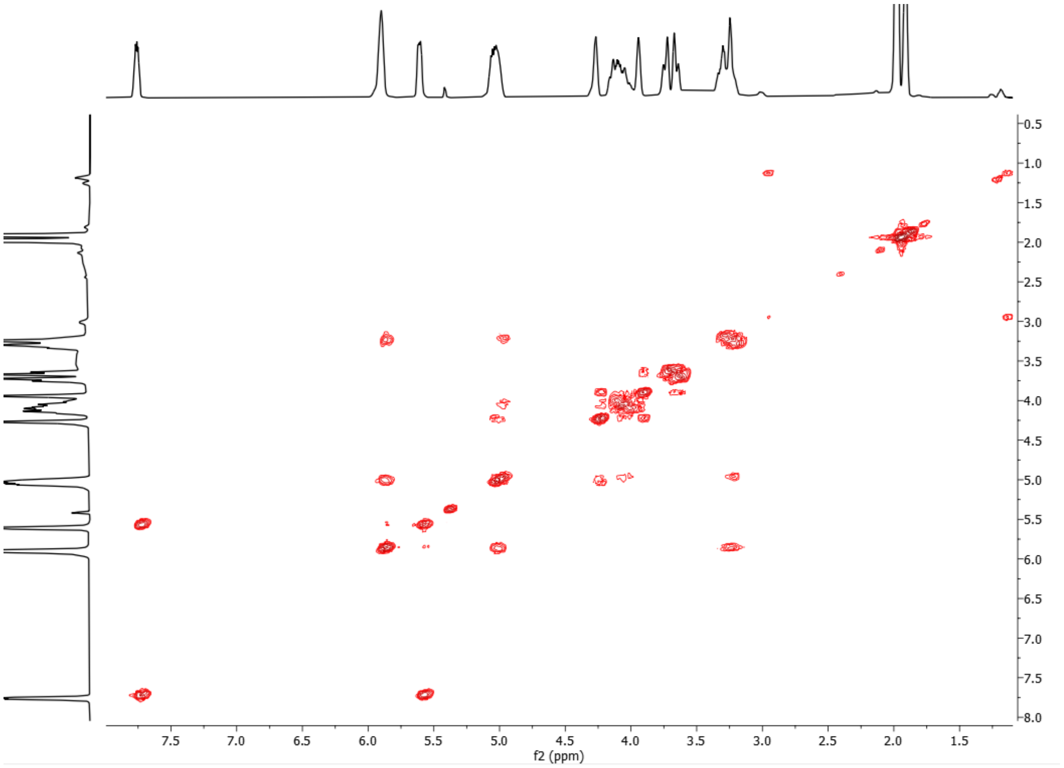

Figure S5.4: gCOSY spectra of compound **6-U**.

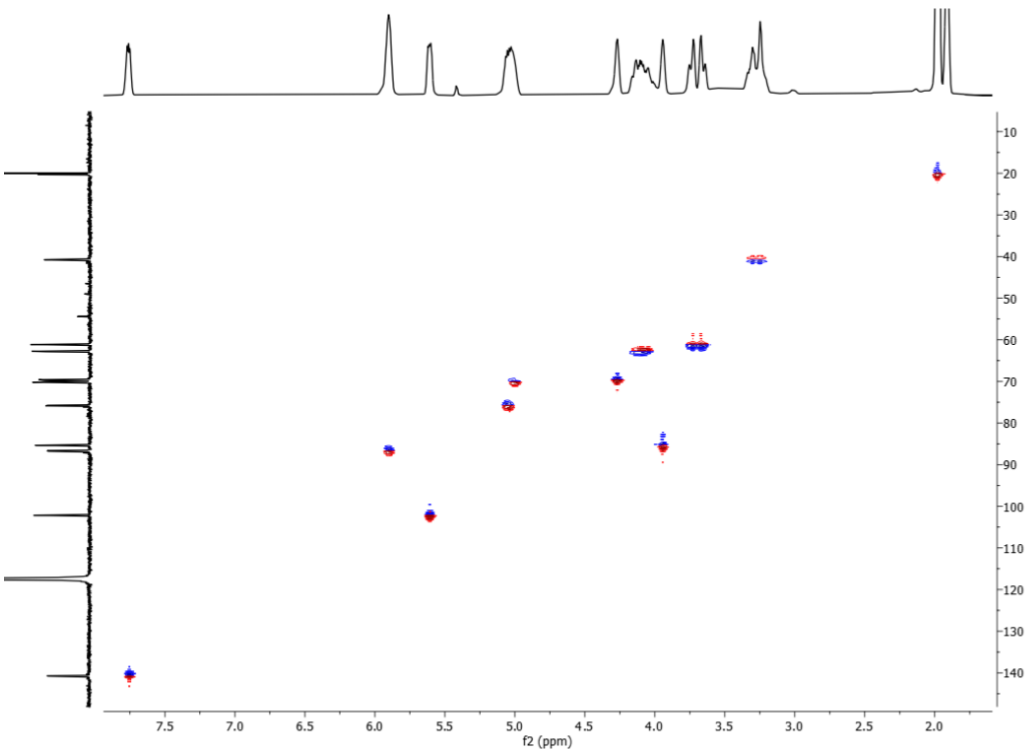

Figure S5.5: HSQC spectra of compound **6-U**.

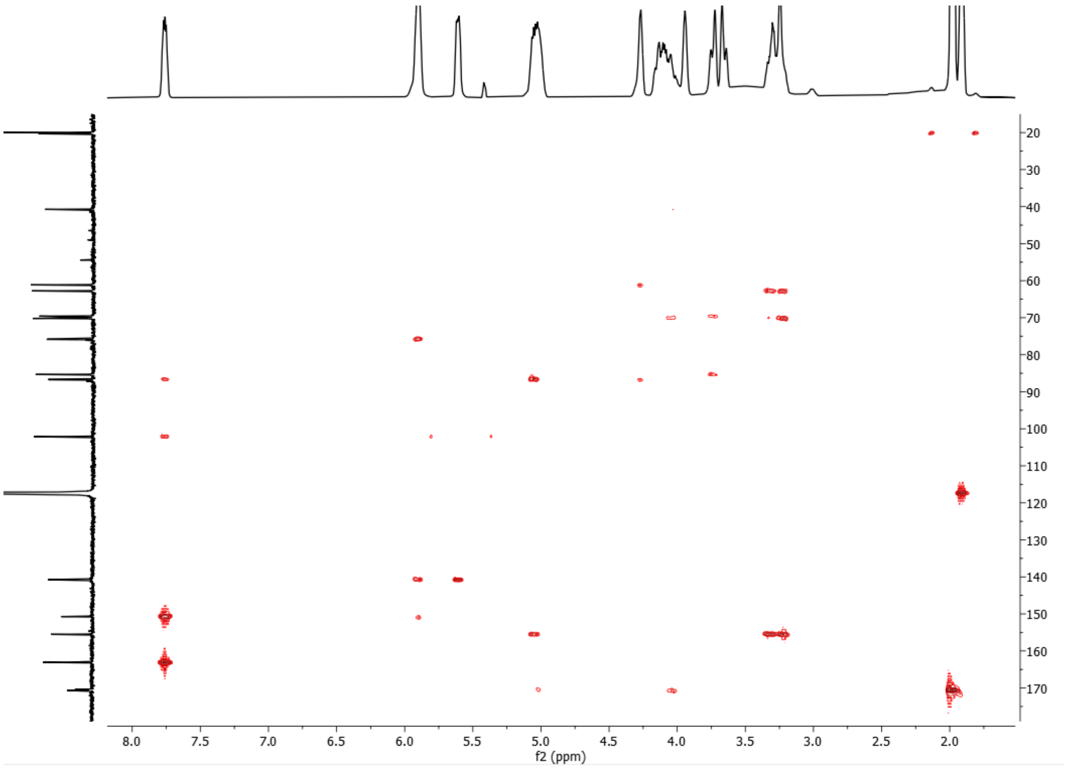

Figure S5.6: HMBC spectra of compound **6-U**.

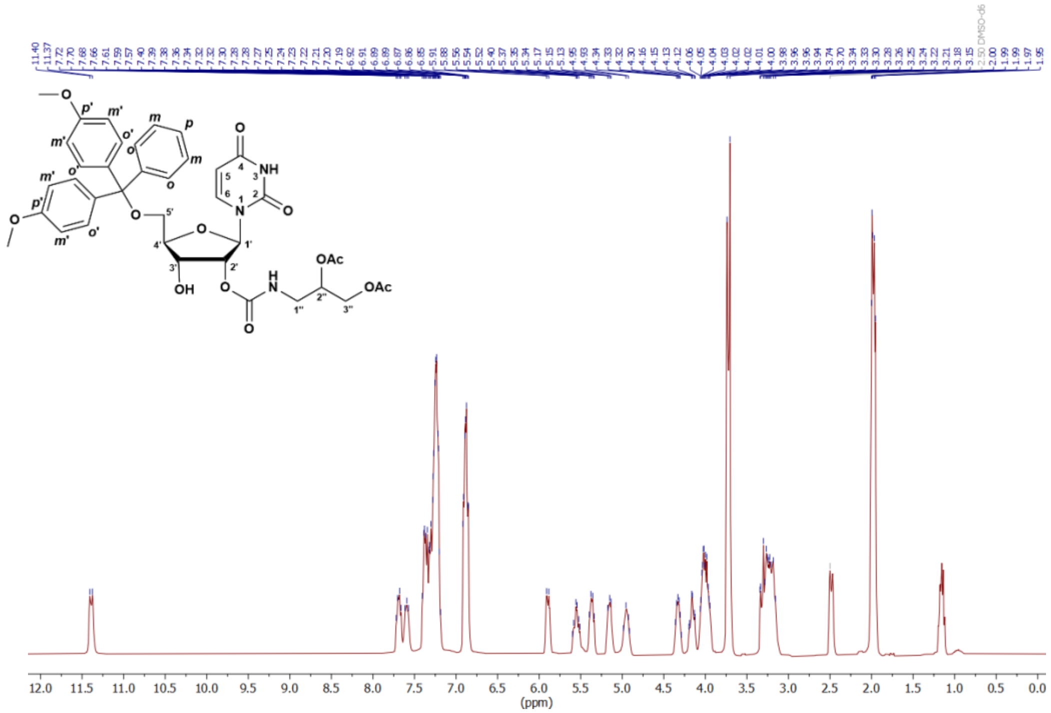

Figure S6.1: ^{1}H NMR spectrum (400 MHz, DMSO-d_6) of compound **7-U**.

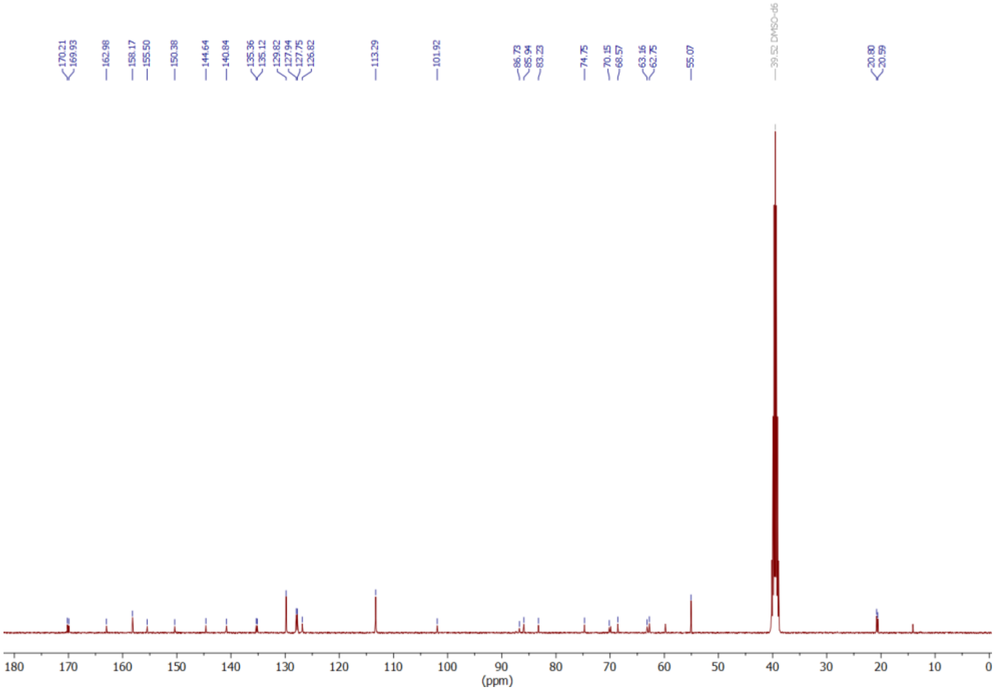

Figure S6.2: ^{13}C NMR spectrum (101 MHz, DMSO-d_6) of compound **7-U**.

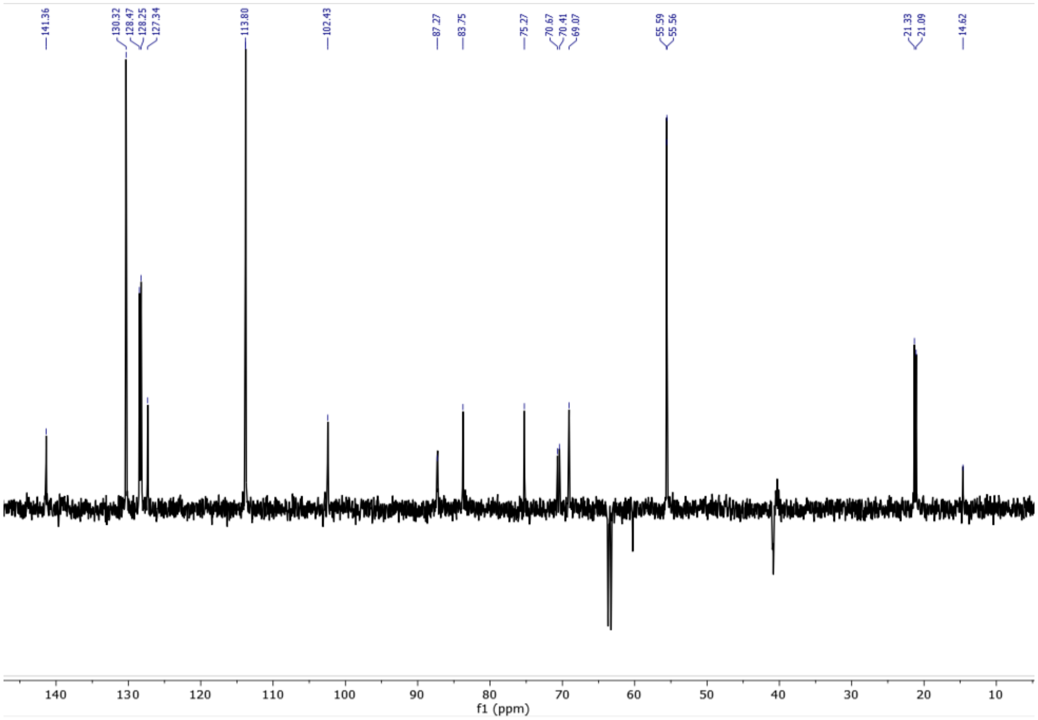

Figure S6.3: ^{13}C DEPT with decoupling NMR spectrum of compound **7-U**.

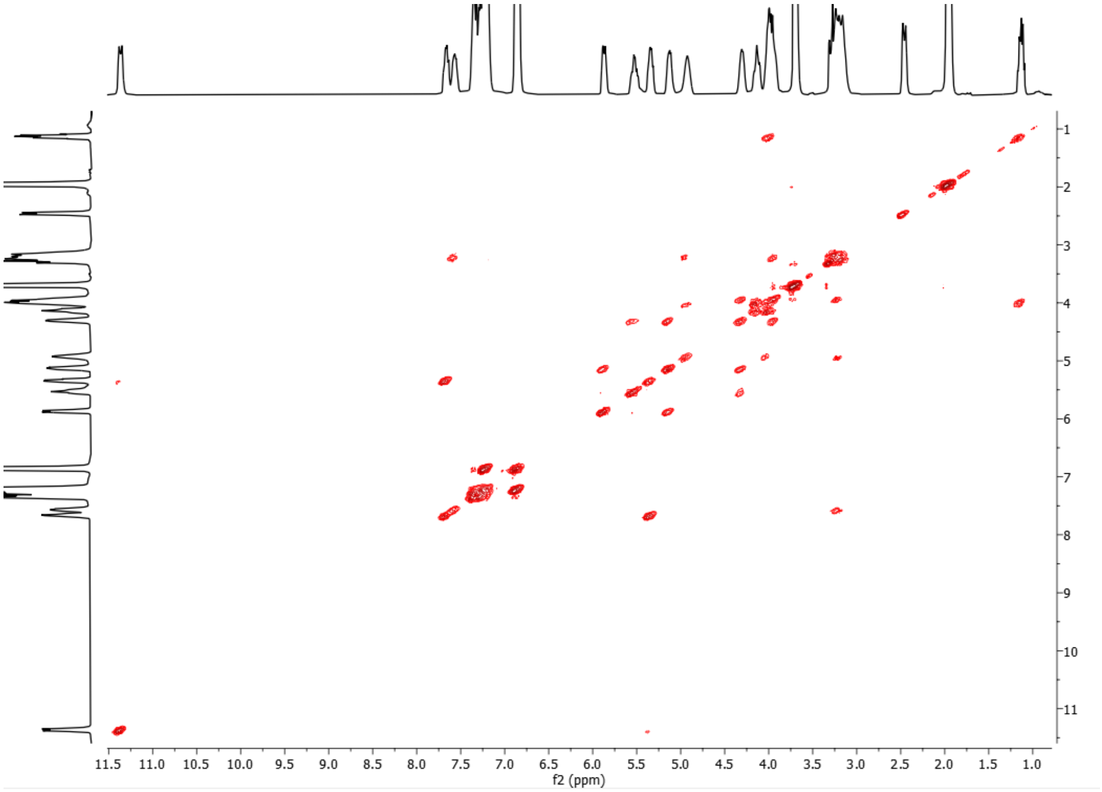

Figure S6.4: gCOSY spectra of compound **7-U**.

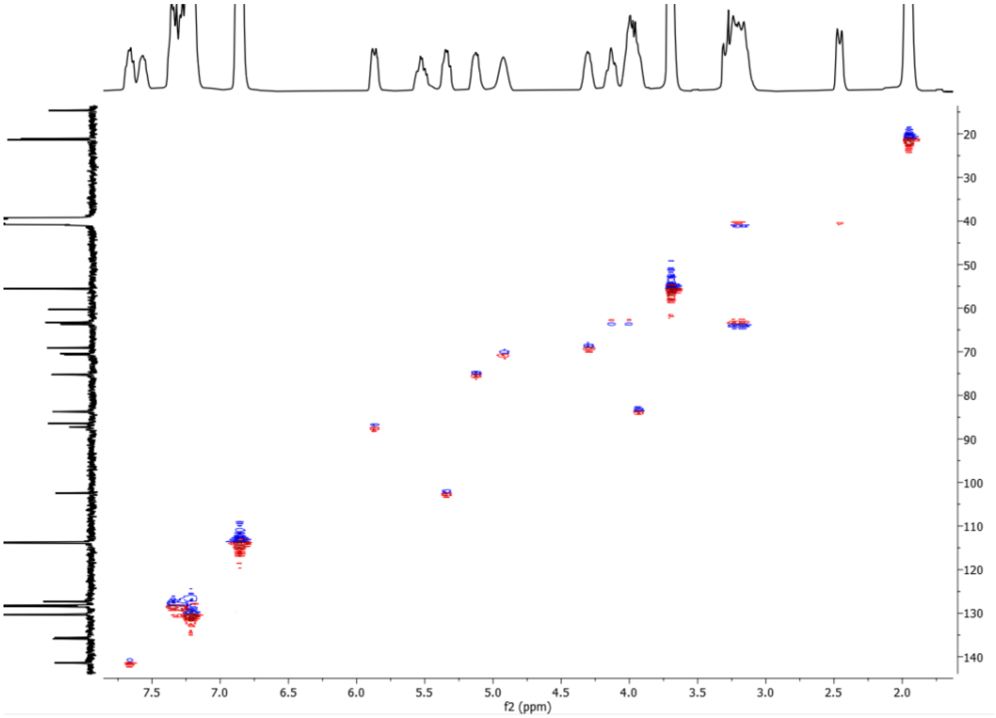

Figure S6.5: HSQC spectra of compound **7-U**.

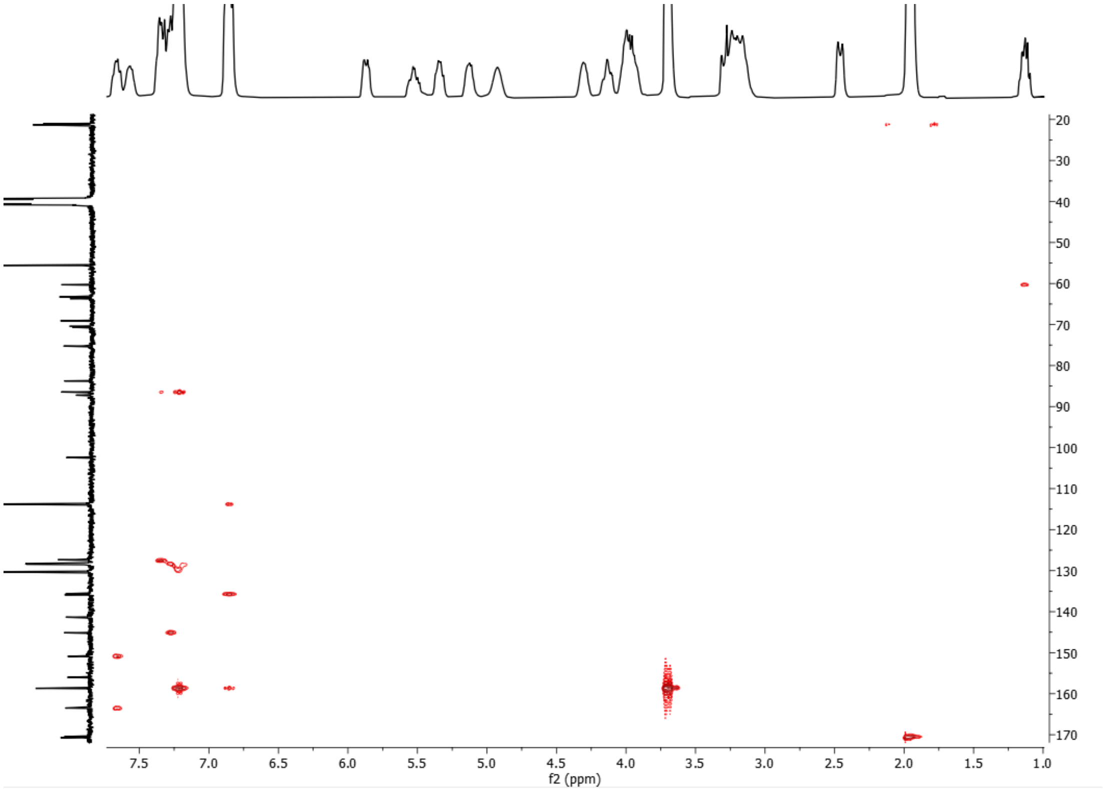

Figure S6.6: HMBC spectra of compound **7-U**.

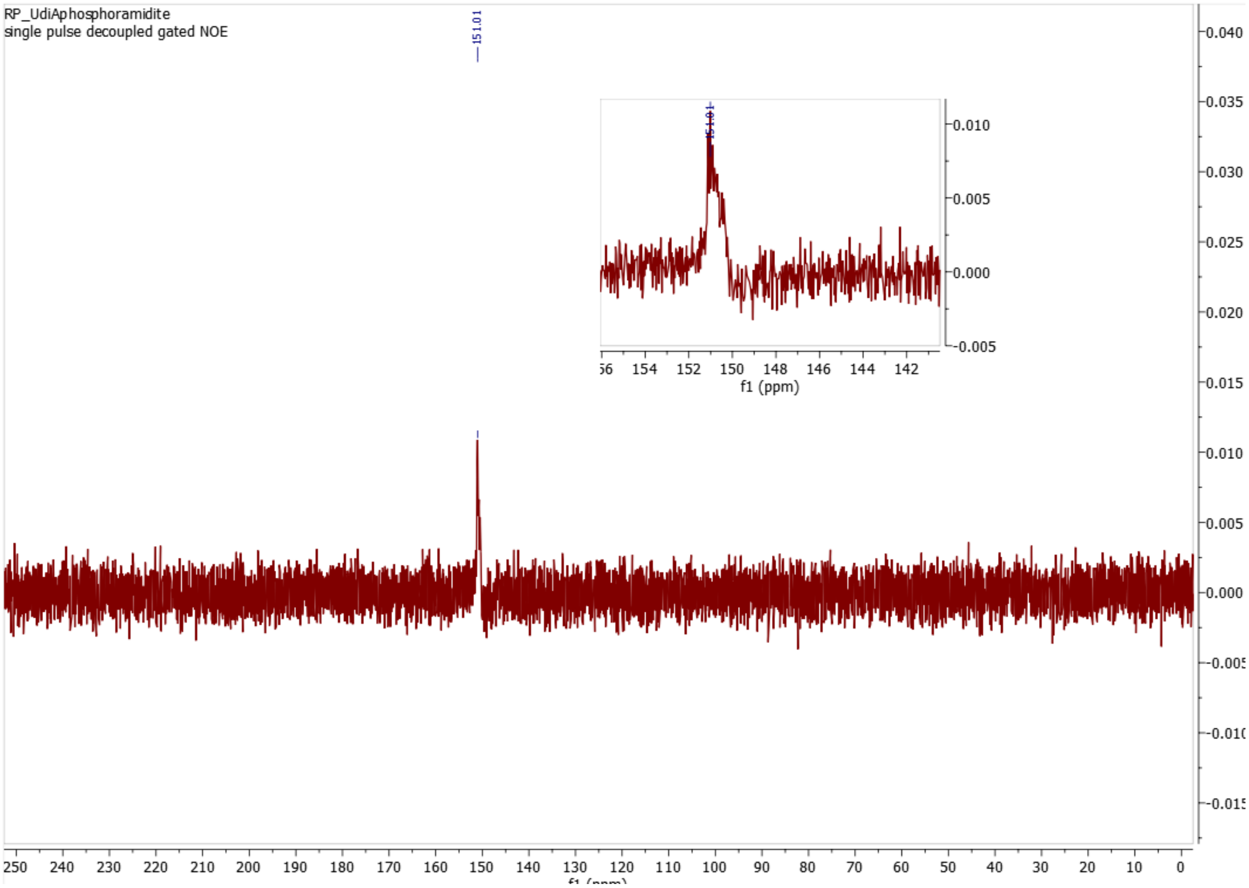

Figure S7: ^{31}P NMR spectrum (162 MHz, $CDCl_3$) of compound **8-U**.

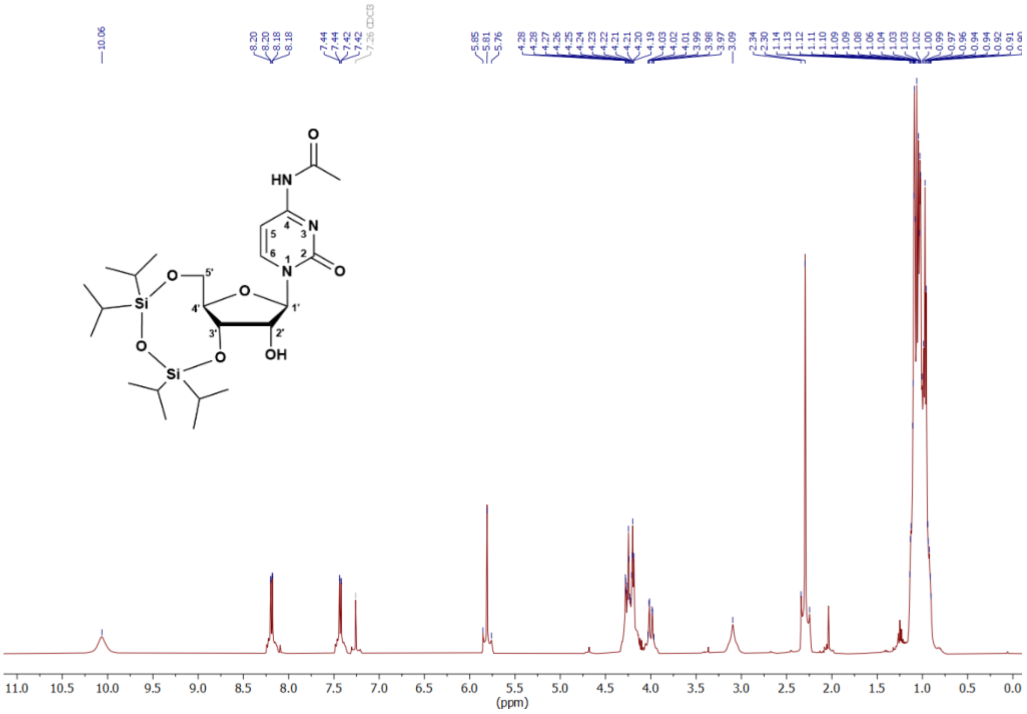

Figure S8.1: ^{1}H NMR spectrum (400 MHz, $CDCl_3$) of compound **2-C**.

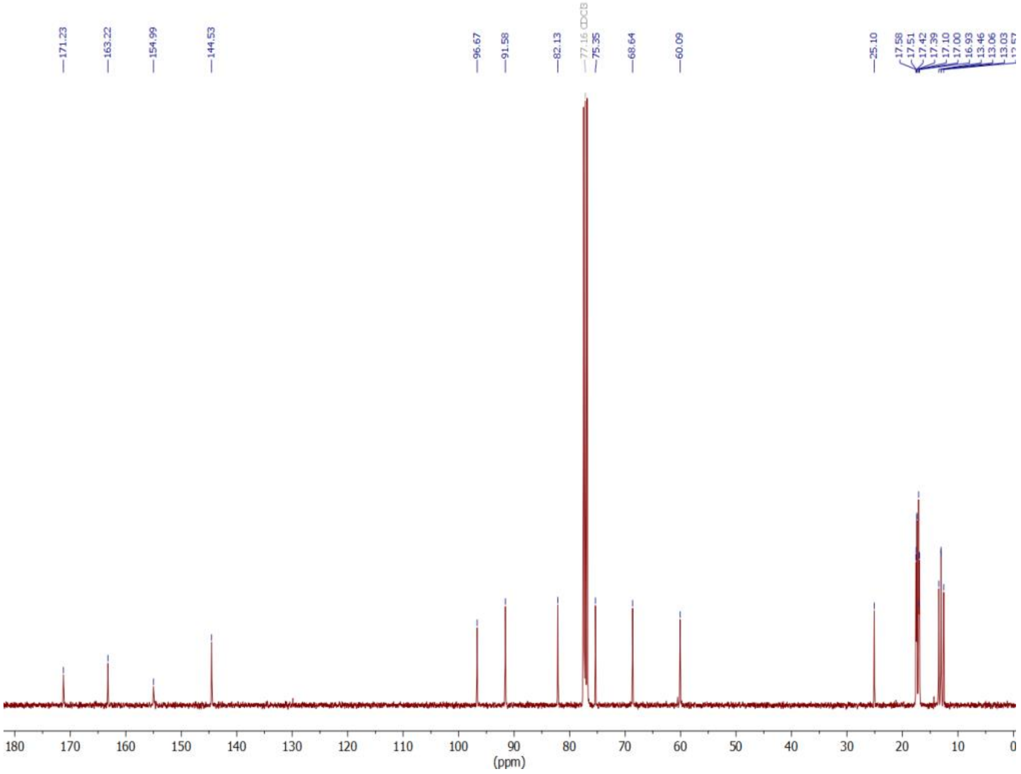

Figure S8.2: ^{13}C NMR spectrum (101 MHz, $CDCl_3$) of compound **2-C**.

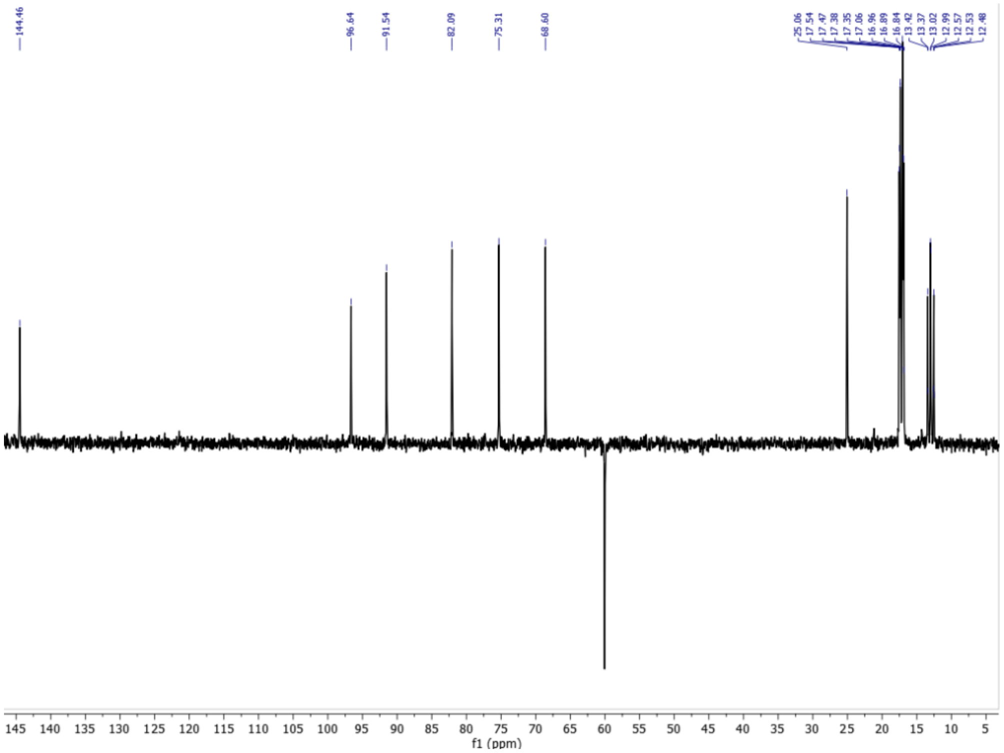

Figure S8.3: ^{13}C DEPT with decoupling NMR spectrum of compound **2-C**.

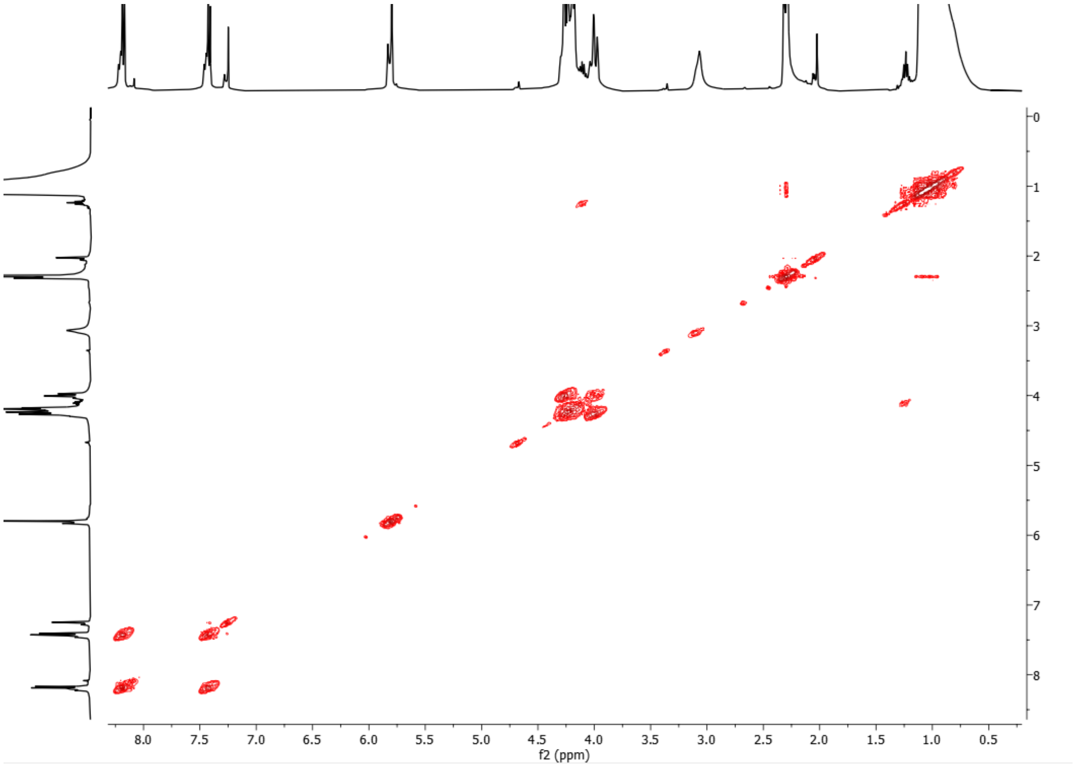

Figure S8.4: gCOSY spectra of compound **2-C**.

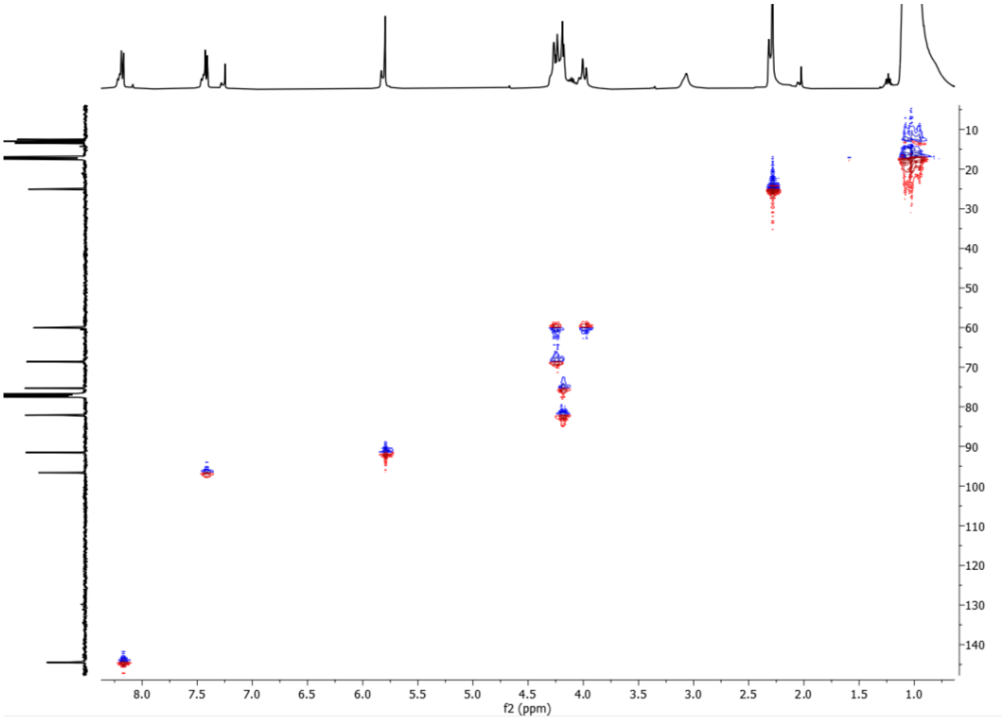

Figure S8.5: HSQC spectra of compound **2-C**.

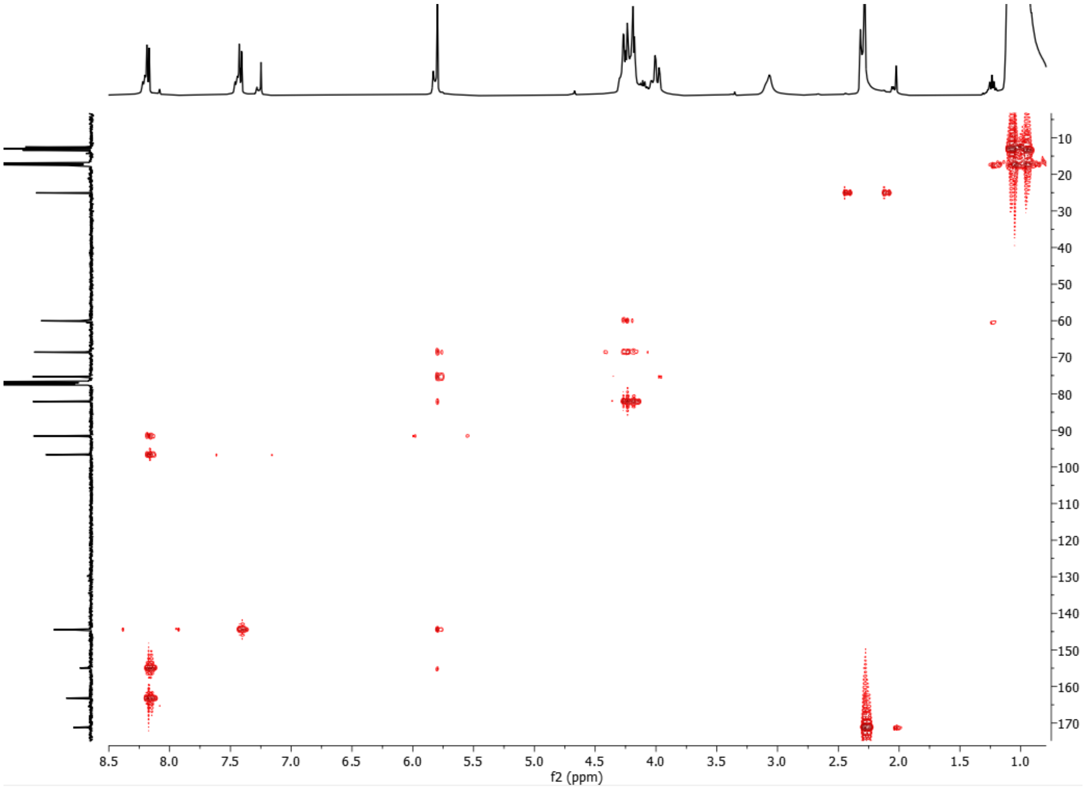

Figure S8.6: HMBC spectra of compound **2-C**.

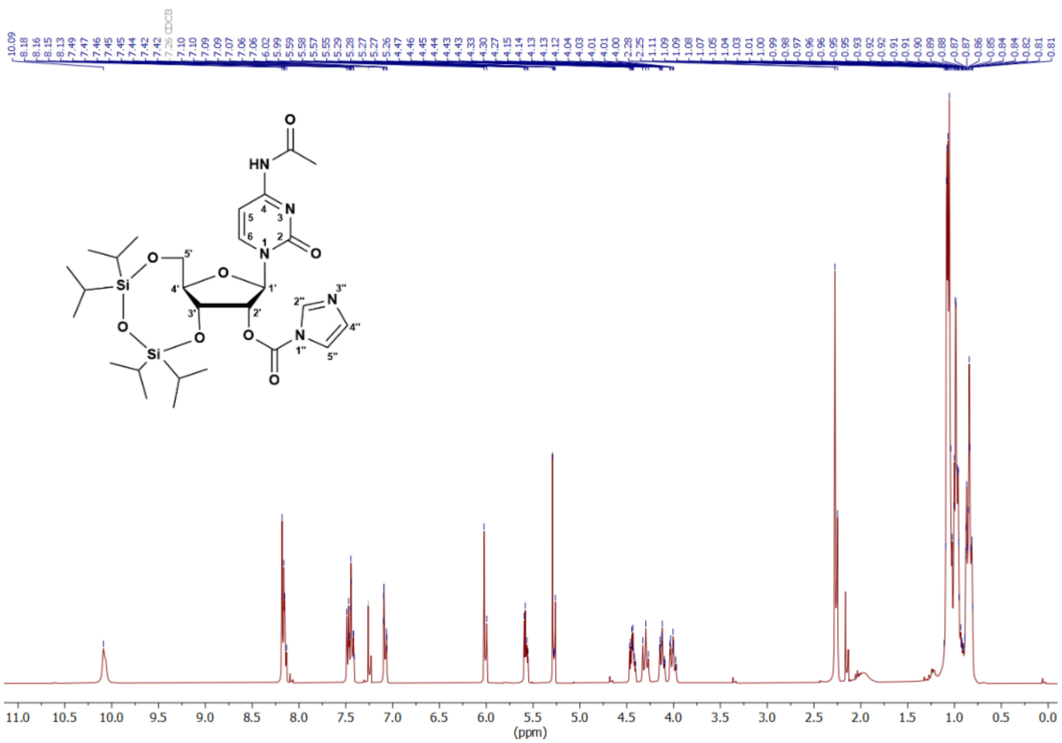

Figure S9.1: ^{1}H NMR spectrum (400 MHz, $CDCl_3$) of compound **3-C**.

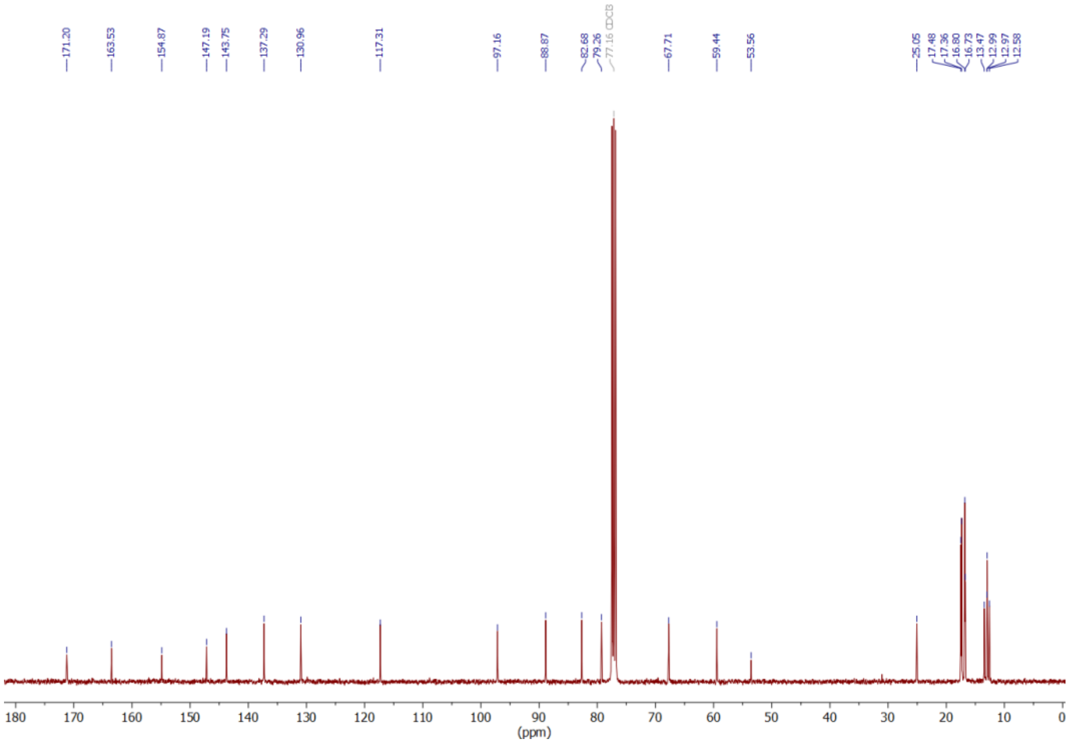

Figure S9.2: ^{13}C NMR spectrum (101 MHz, $CDCl_3$) of compound **3-C**.

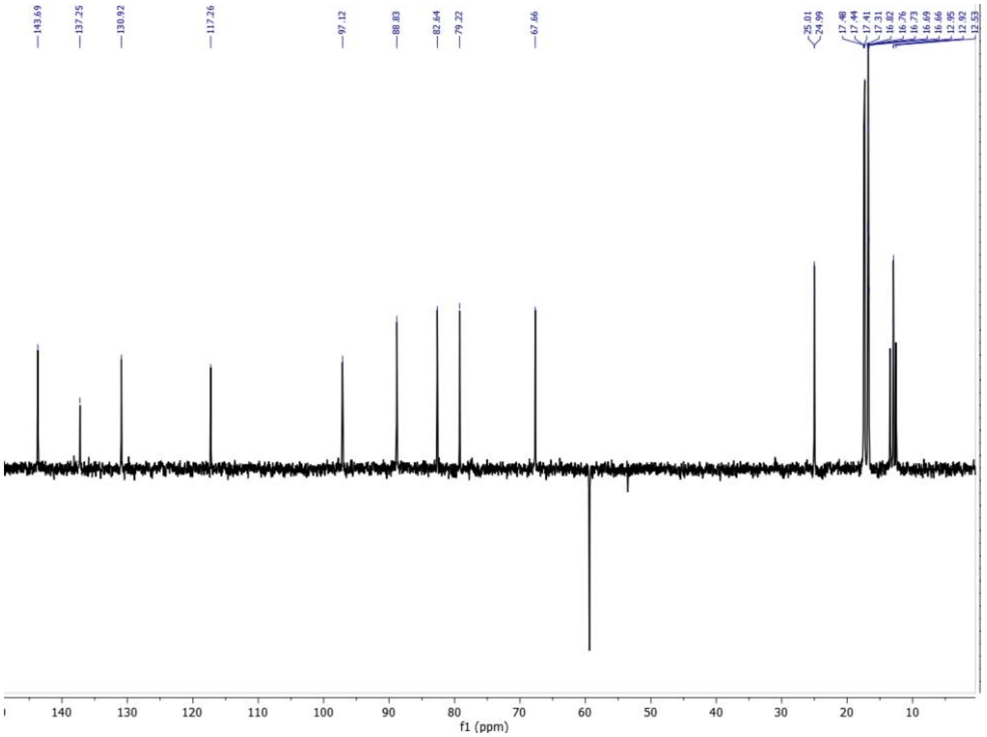

Figure S9.3: ^{13}C DEPT with decoupling NMR spectrum of compound **3-C**.

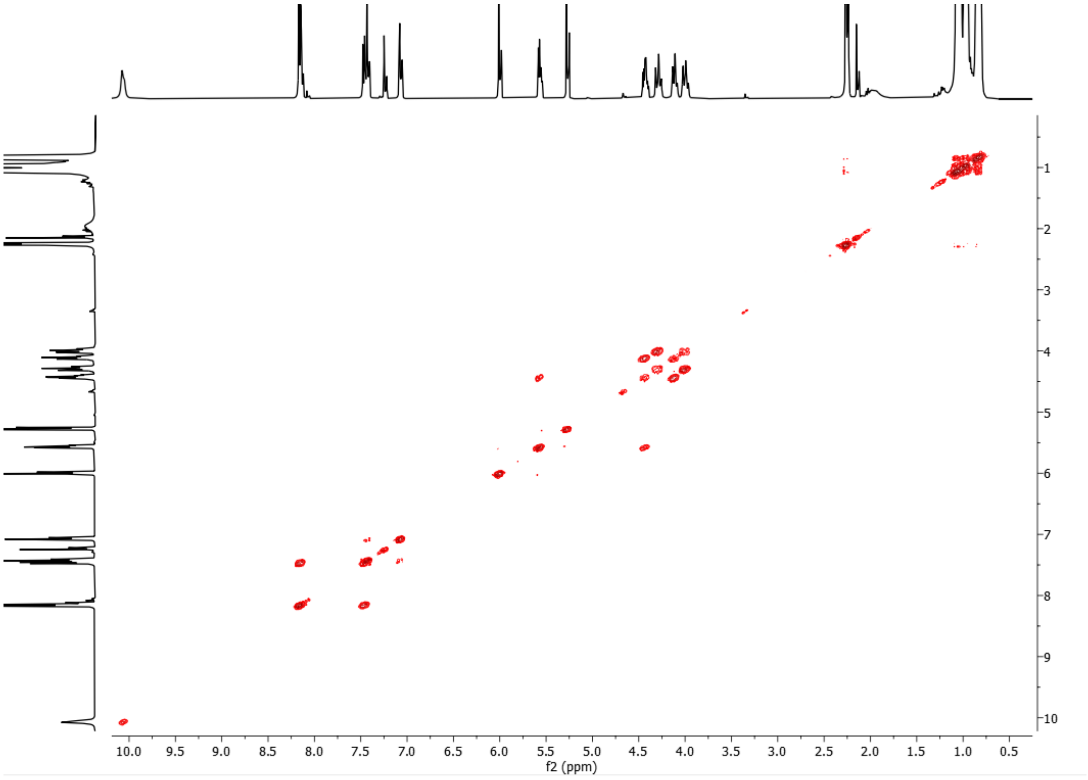

Figure S9.4: gCOSY spectra of compound **3-C**.

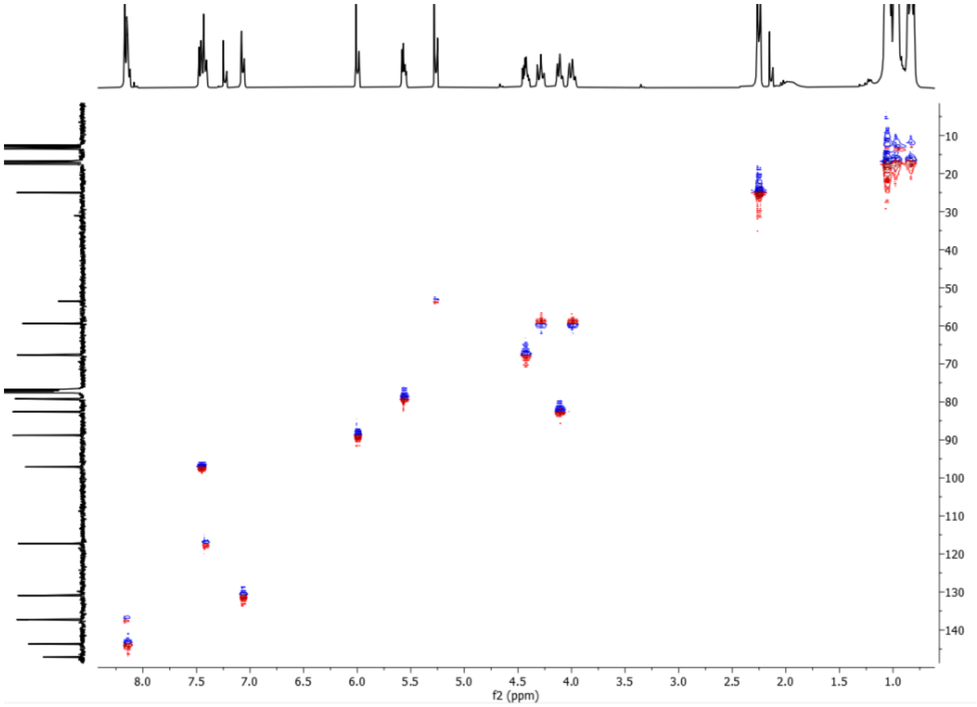

Figure S9.5: HSQC spectra of compound **3-C**.

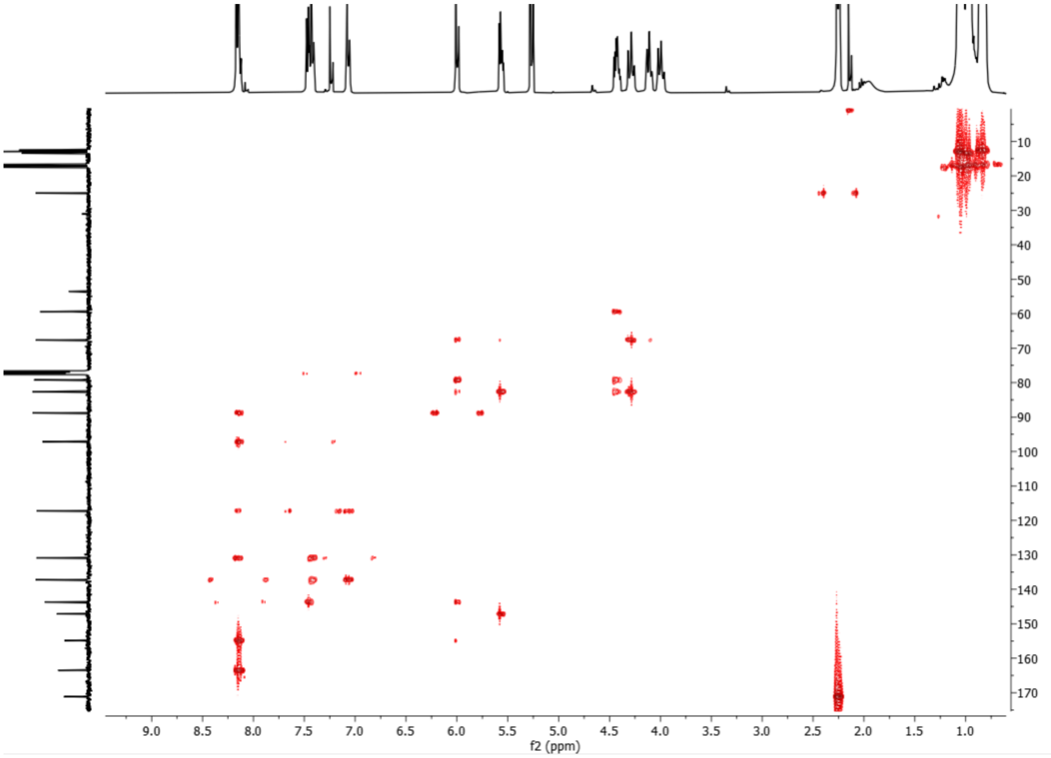

Figure S9.6: HMBC spectra of compound **3-C**.

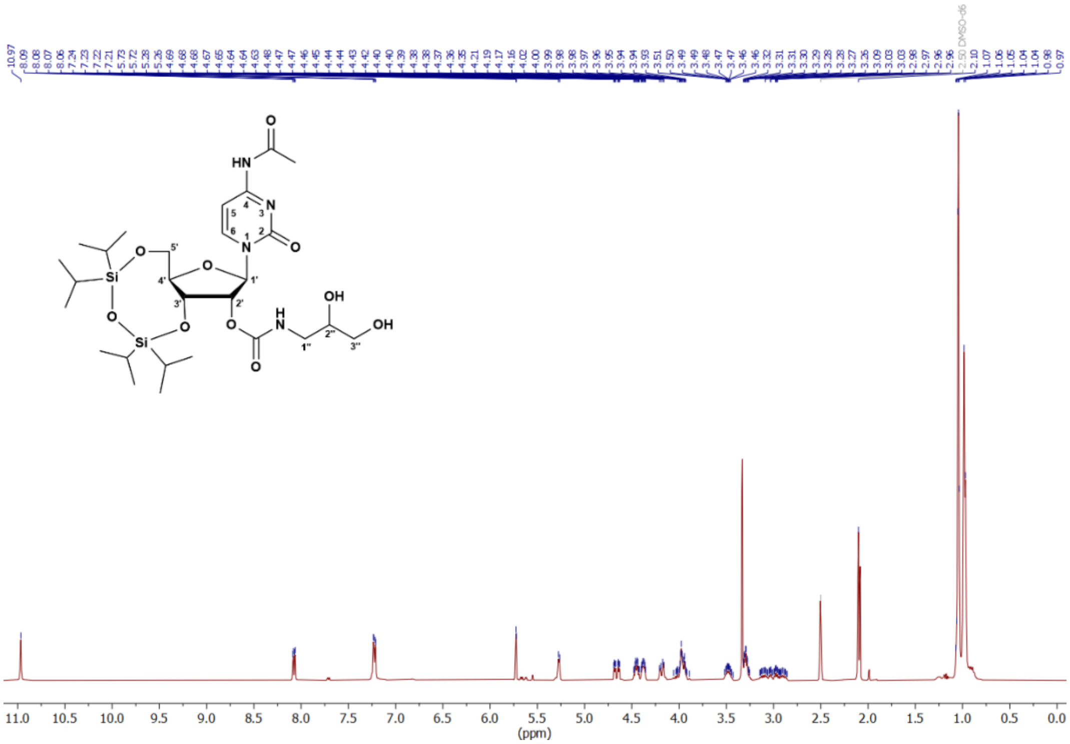

Figure S10.1: ^{1}H NMR spectrum (400 MHz, DMSO-d_6) of compound **4-C**.

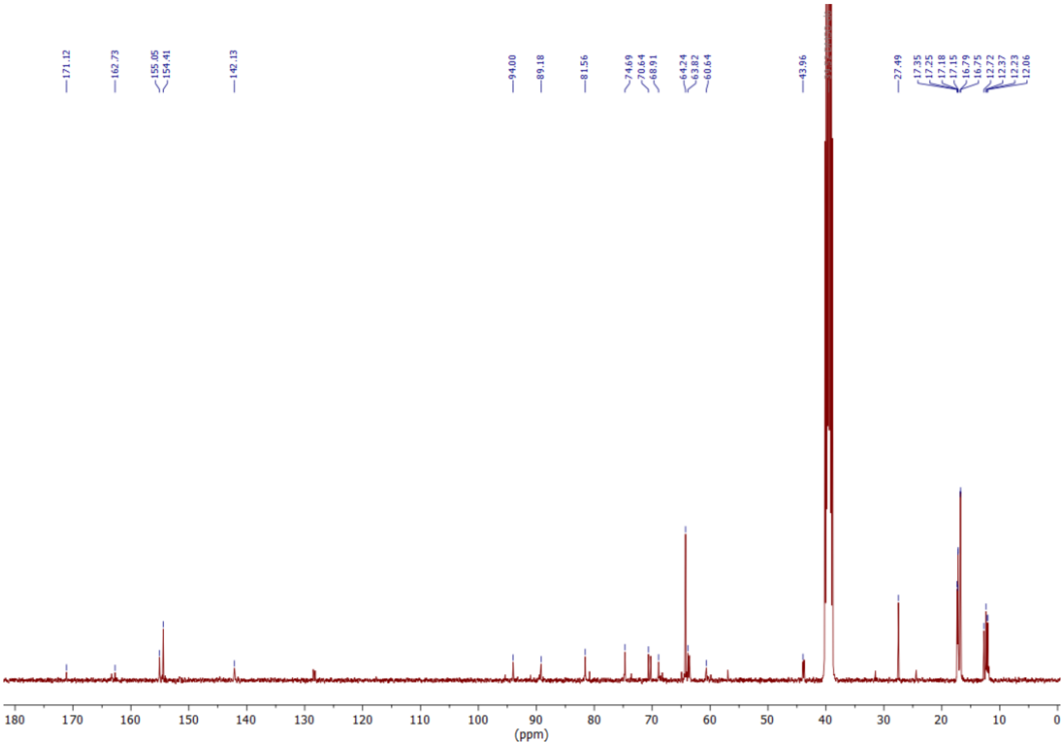

Figure S10.2: ^{13}C NMR spectrum (101 MHz, DMSO-d_6) of compound **4-C**.

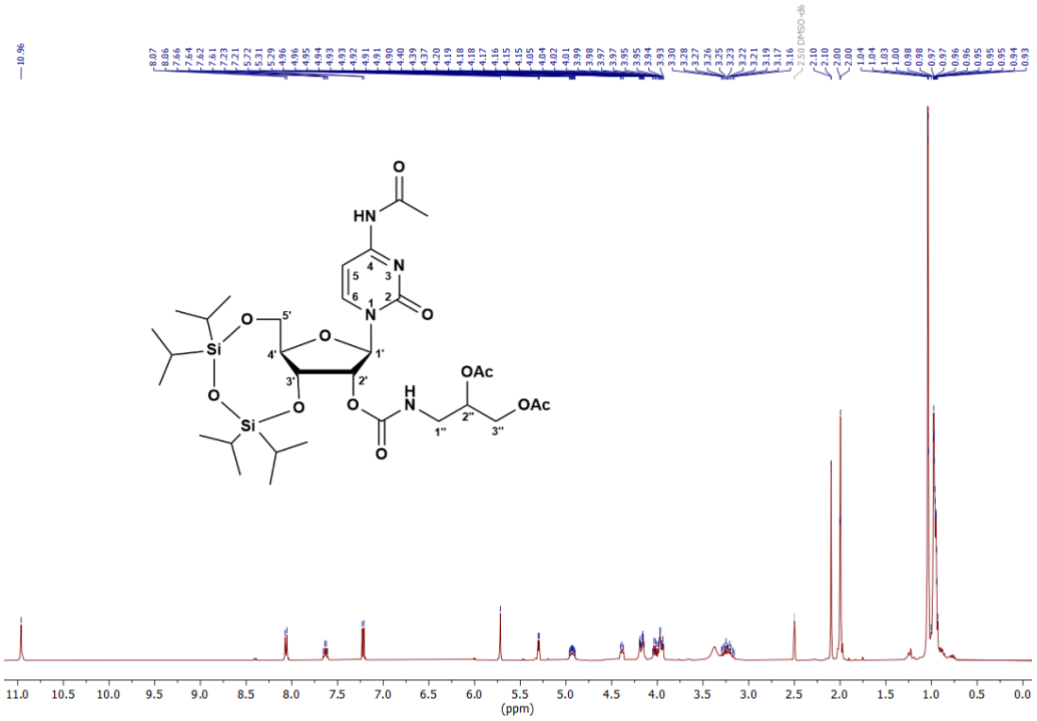

Figure S11.1: ^{1}H NMR spectrum (400 MHz, DMSO-d_6) of compound **5-C**.

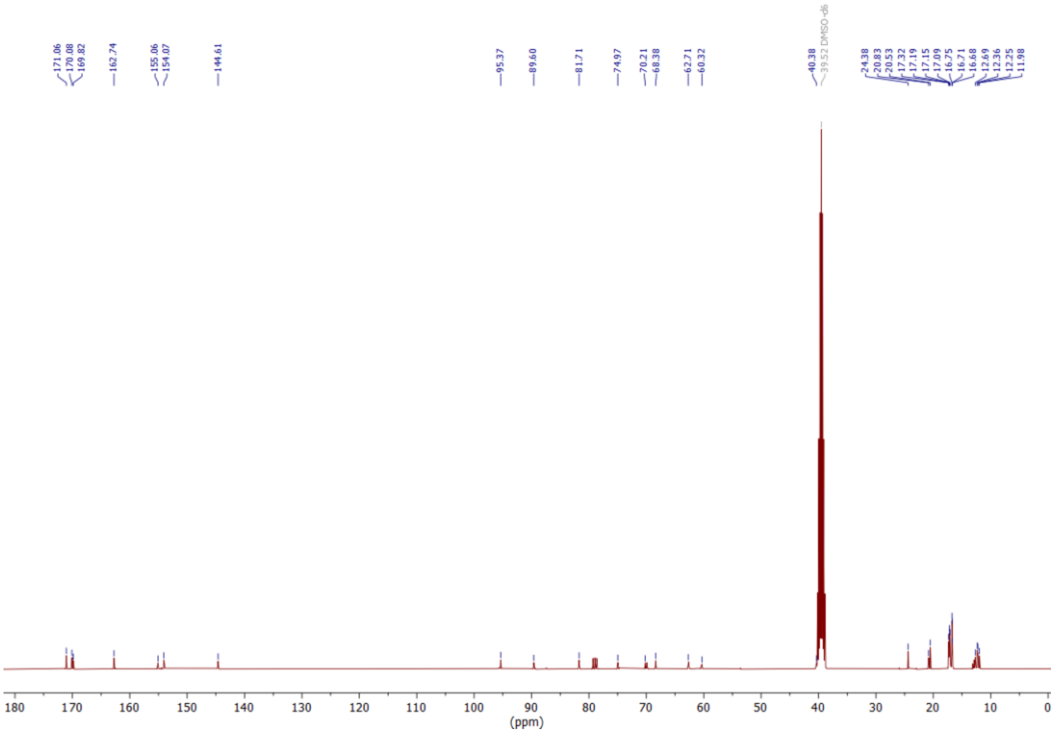

Figure S11.2: ^{13}C NMR spectrum (101 MHz, DMSO-d_6) of compound **5-C**.

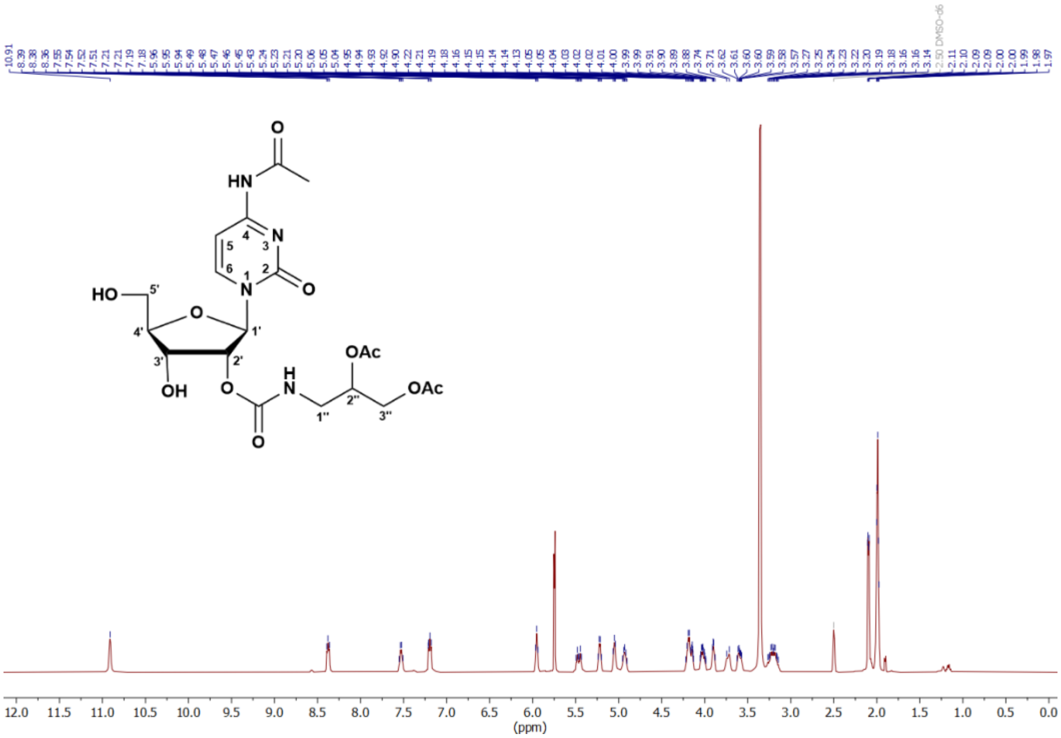

Figure S12.1: ^{1}H NMR spectrum (400 MHz, DMSO-d_6) of compound **6-C**.

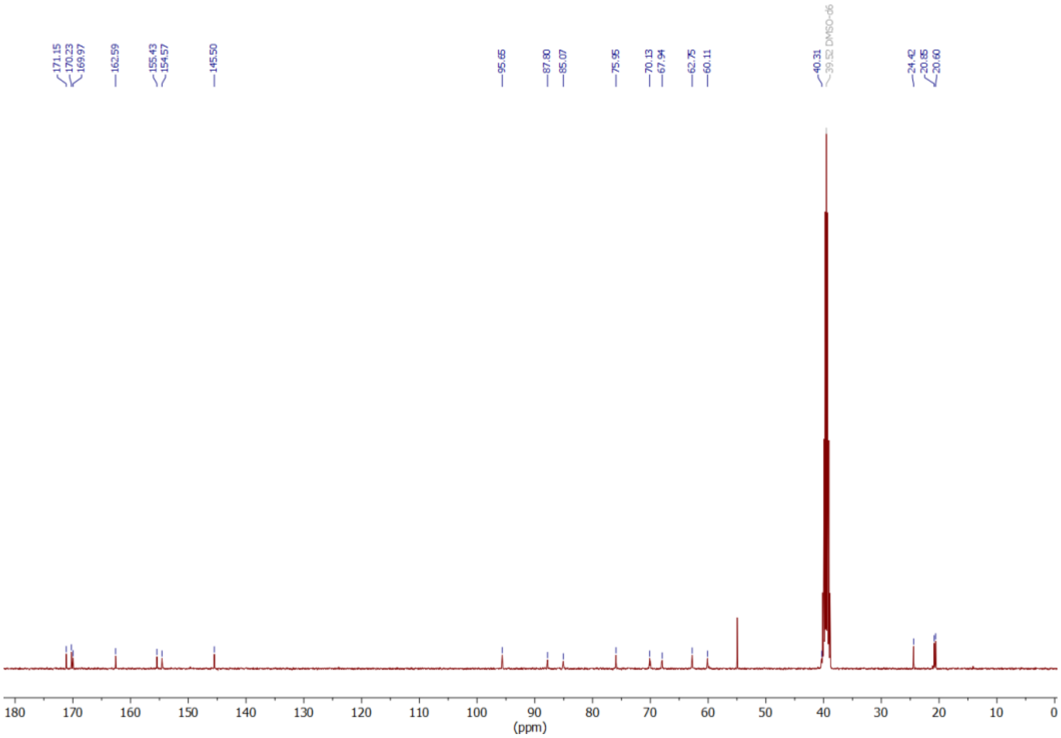

Figure S12.2: ^{13}C NMR spectrum (101 MHz, DMSO-d_6) of compound **6-C**.

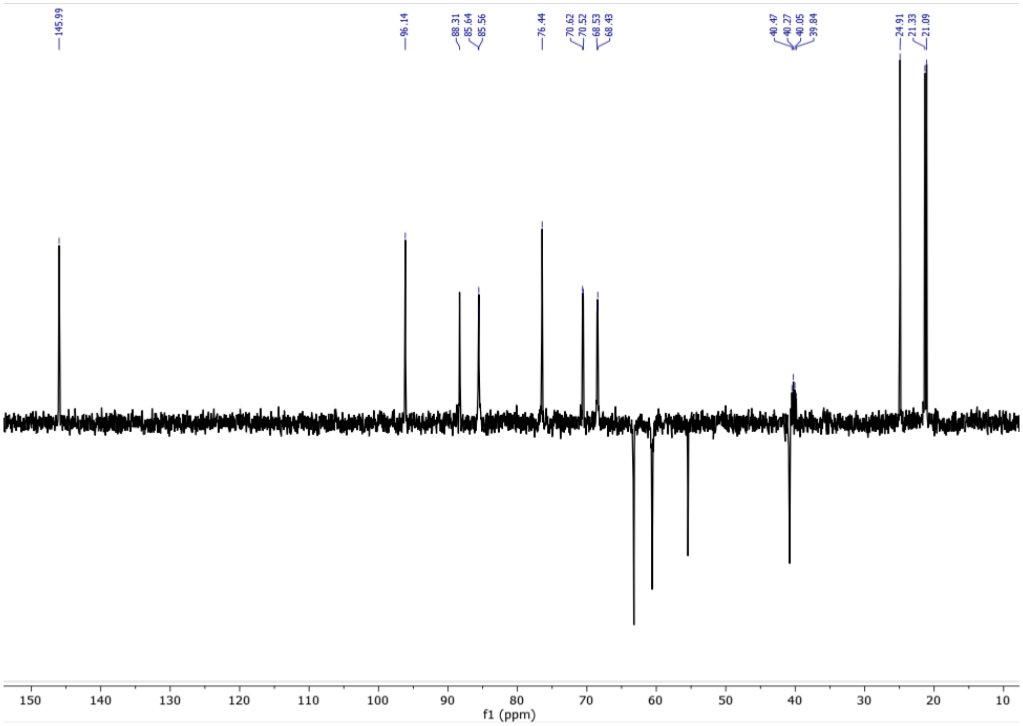

Figure S12.3: ^{13}C DEPT with decoupling NMR spectrum of compound **6-C**.

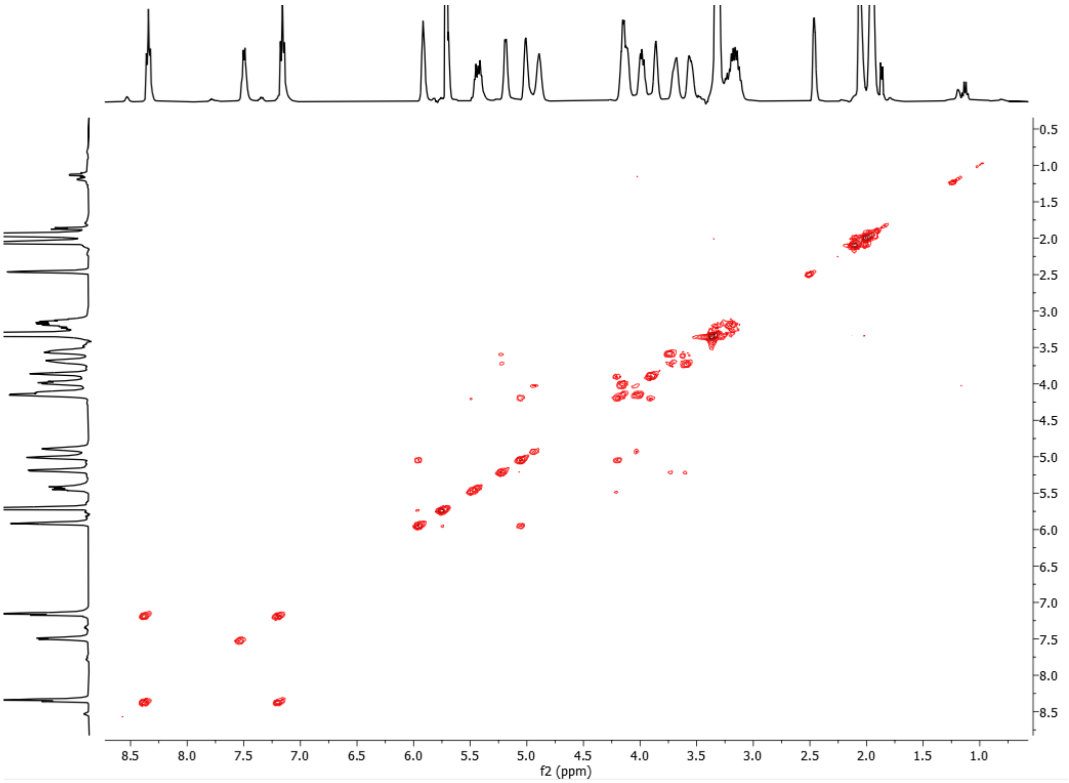

Figure S12.4: gCOSY spectra of compound **6-C**.

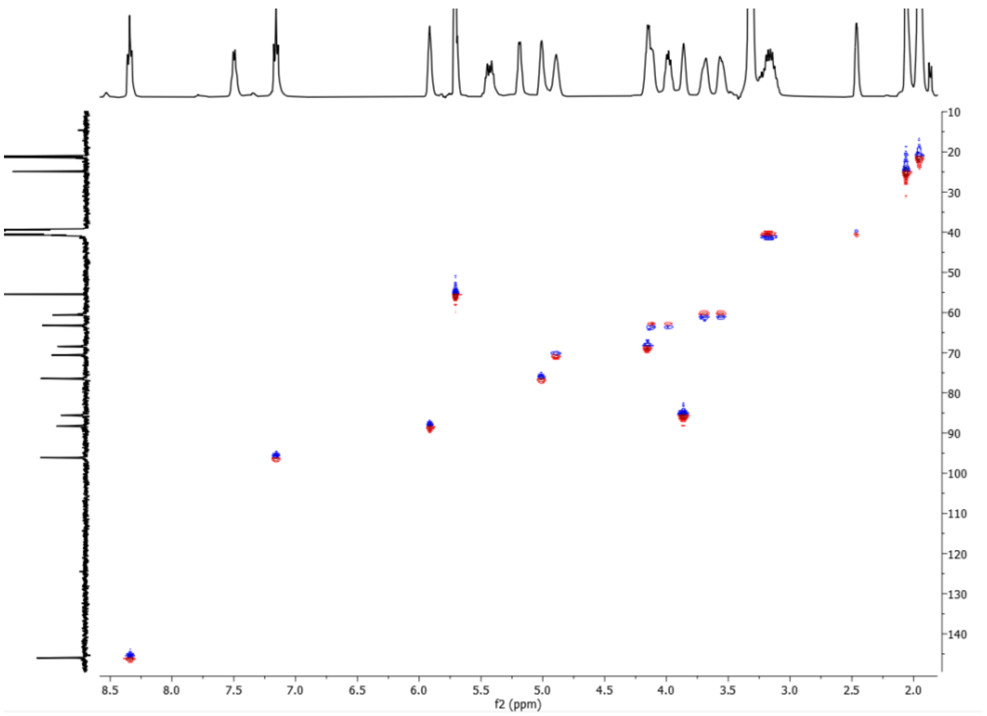

Figure S12.5: HSQC spectra of compound **6-C**.

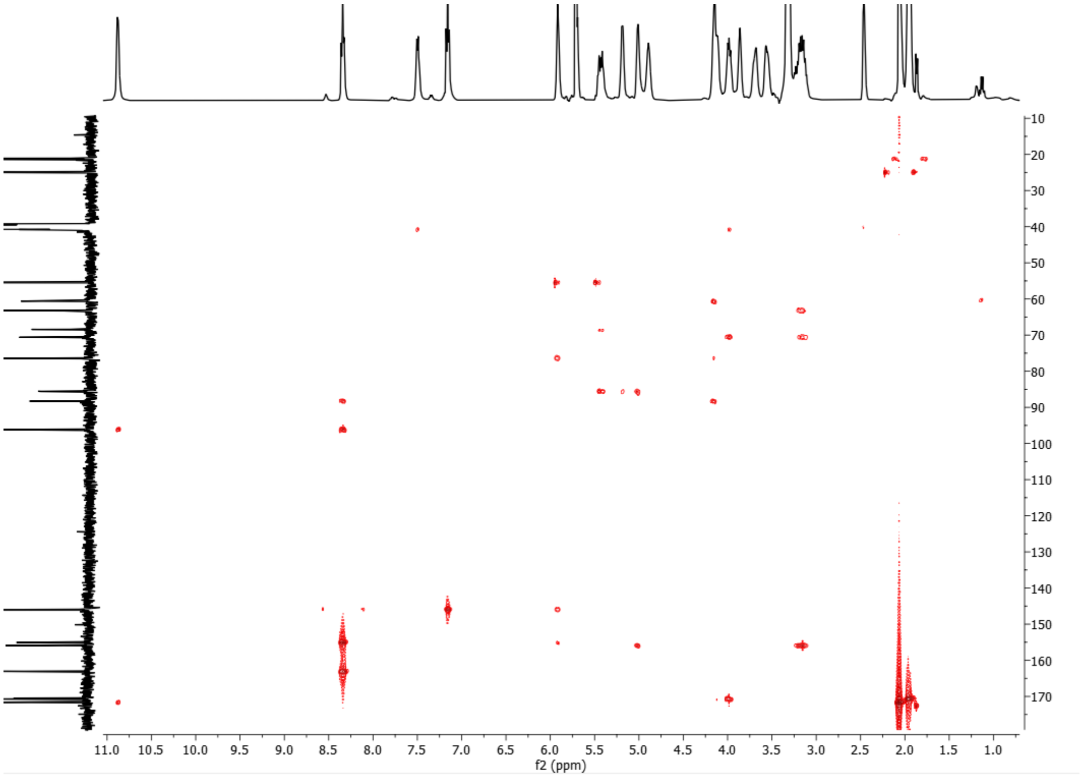

Figure S12.6: HMBC spectra of compound **6-C**.

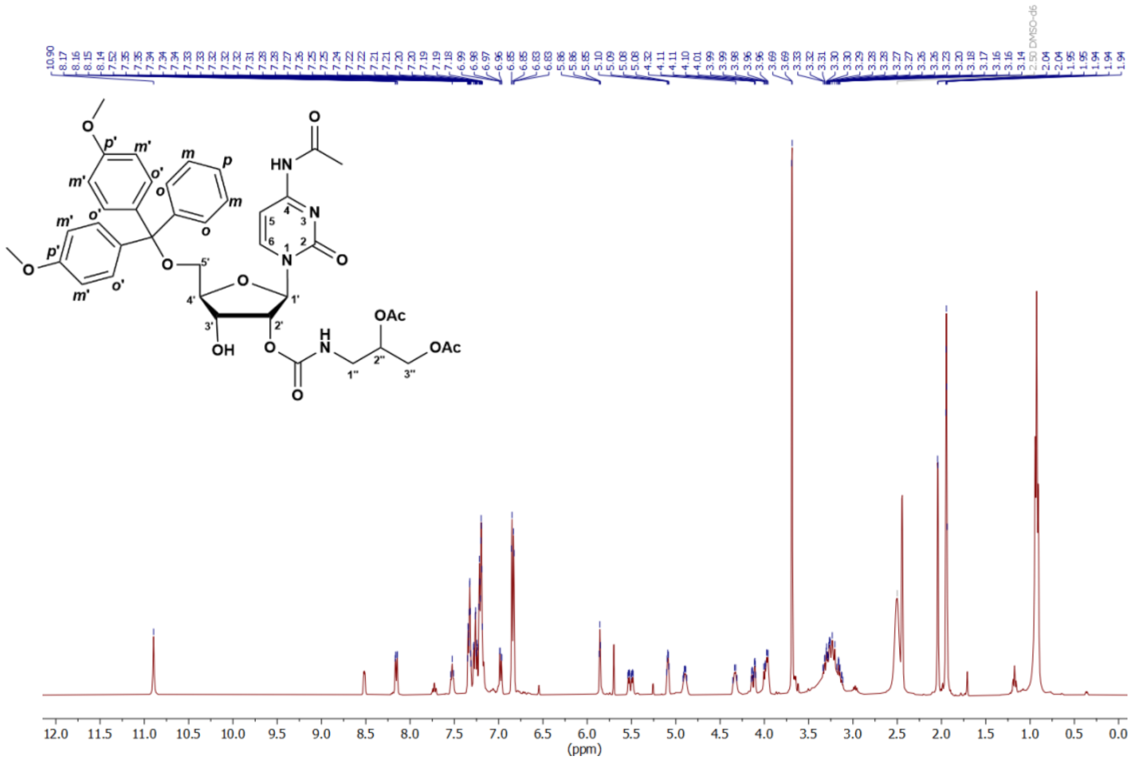

Figure S13.1: ^{1}H NMR spectrum (400 MHz, DMSO-d_6) of compound **7-C**.

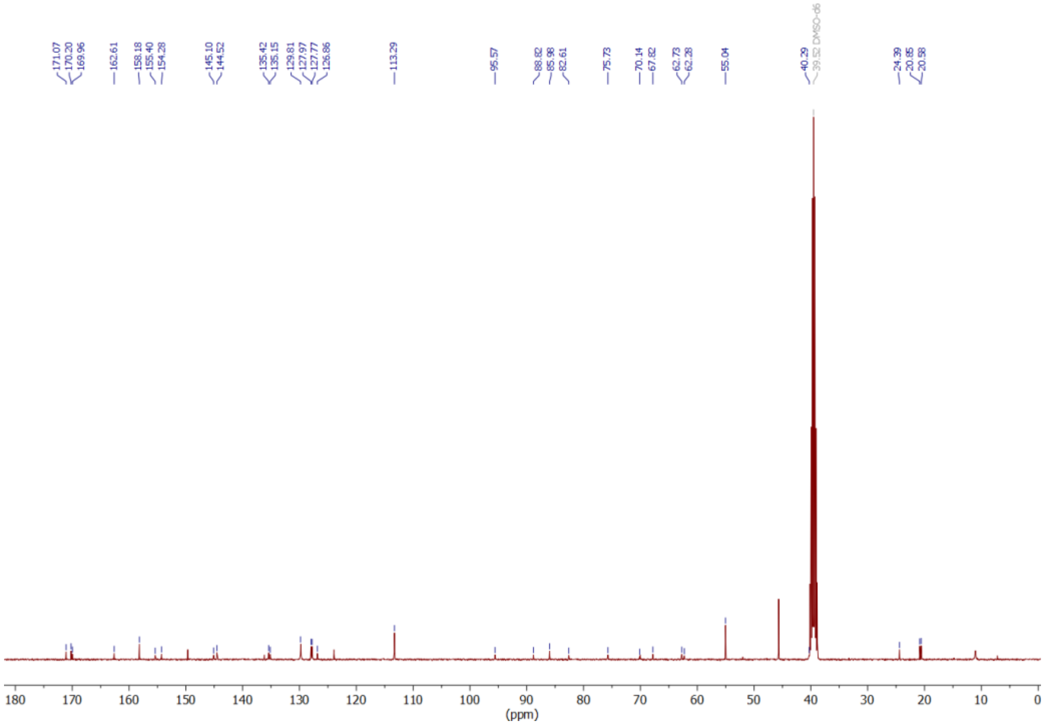

Figure S13.2: ^{13}C NMR spectrum (101 MHz, DMSO-d_6) of compound **7-C**.

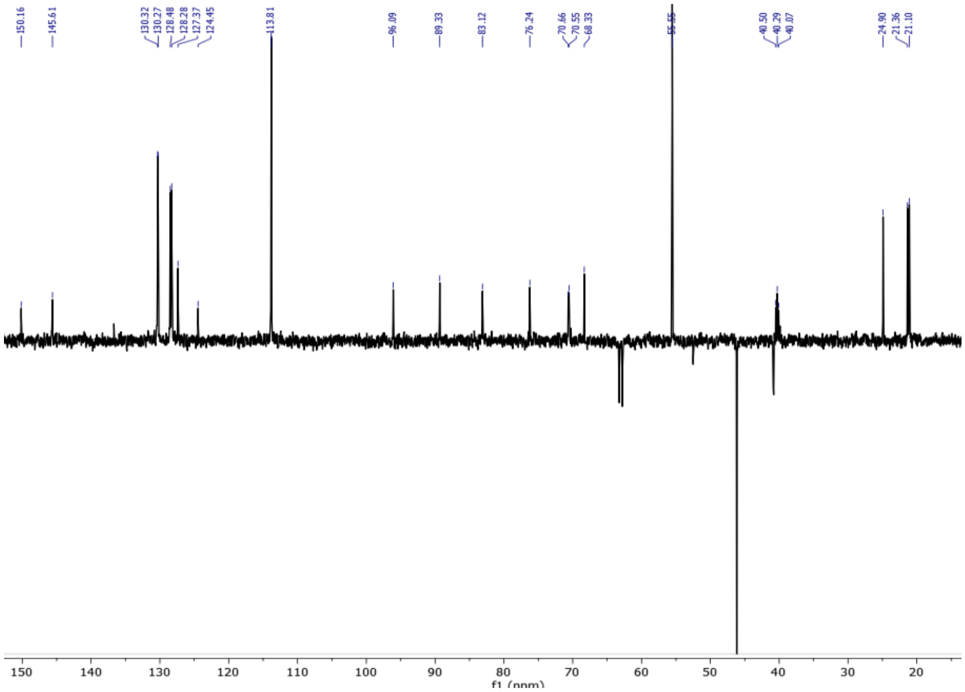

Figure S13.3: ^{13}C DEPT with decoupling NMR spectrum of compound **7-C**.

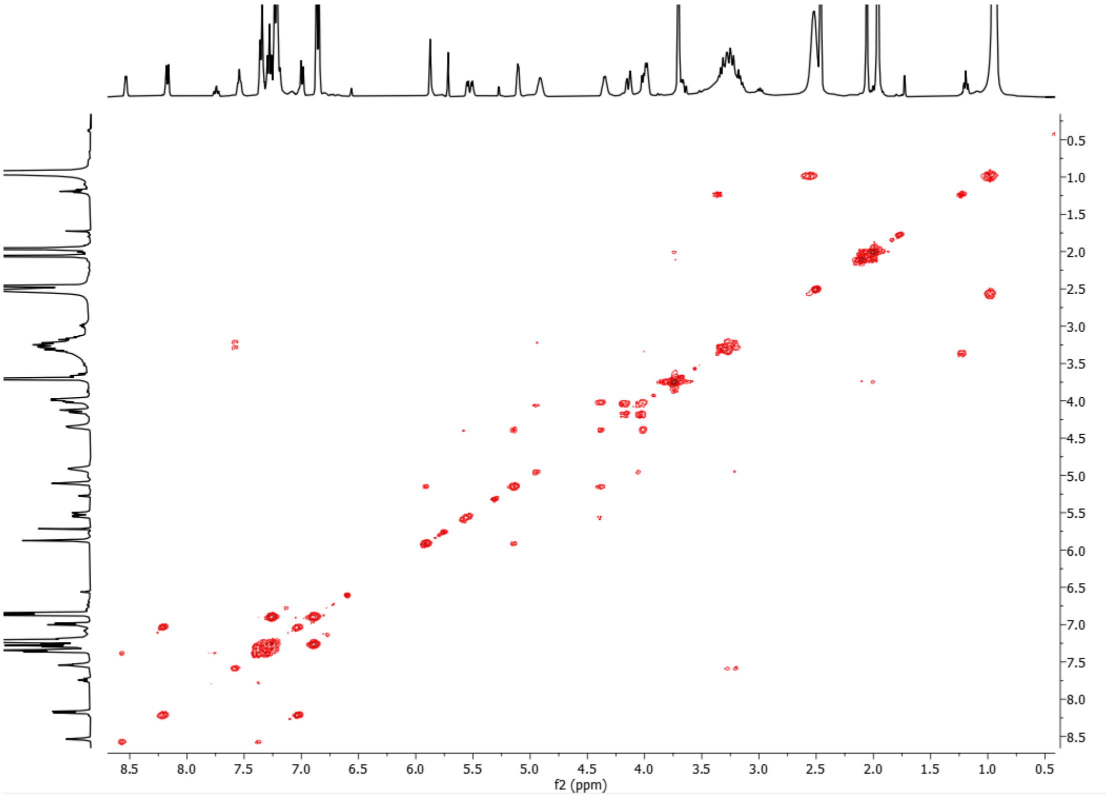

Figure S13.4: gCOSY spectra of compound **7-C**.

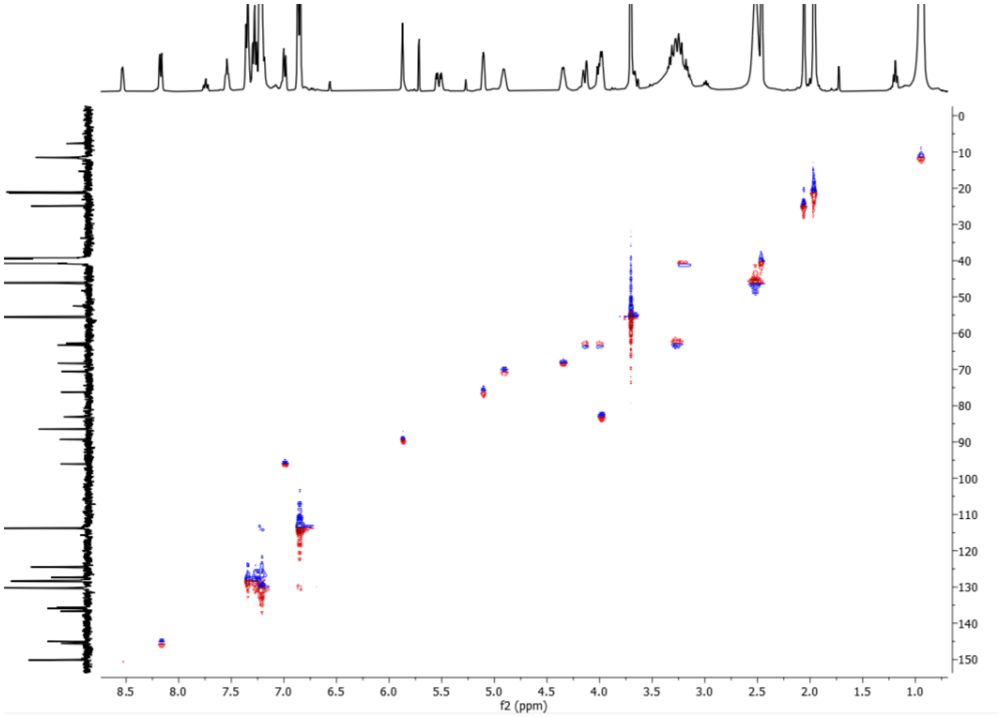

Figure S13.5: HSQC spectra of compound **7-C**.

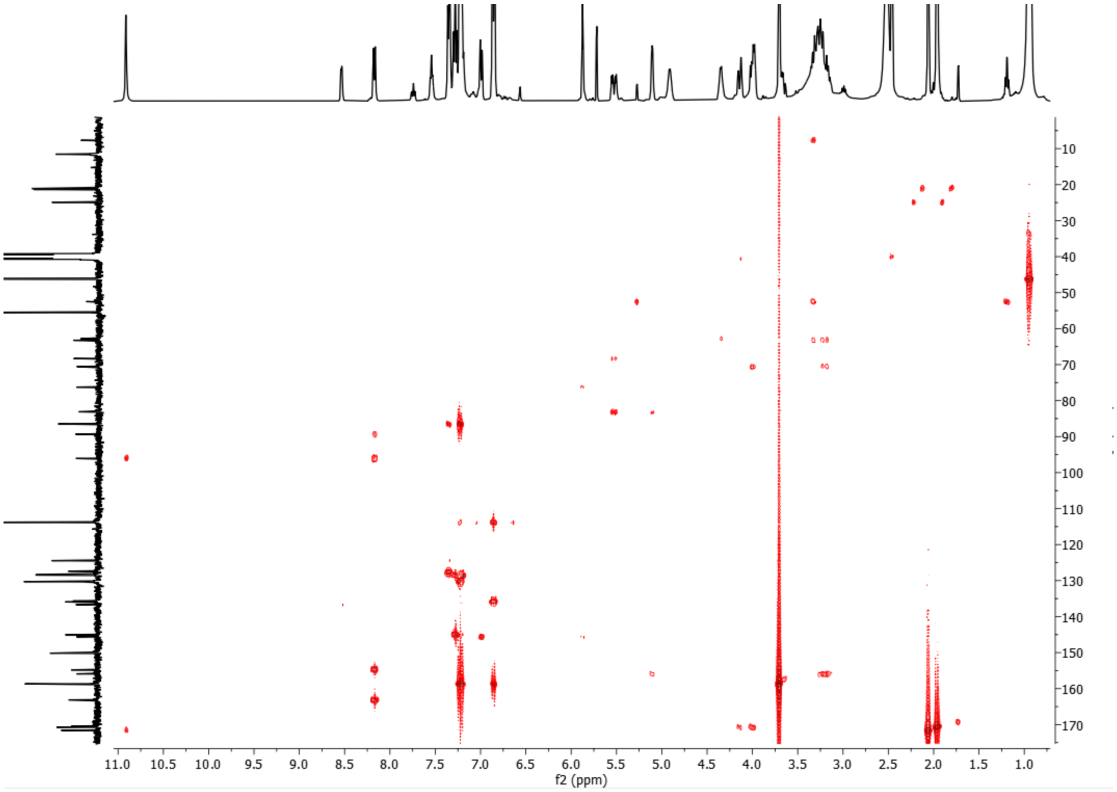

Figure S13.6: HMBC spectra of compound **7-C**.

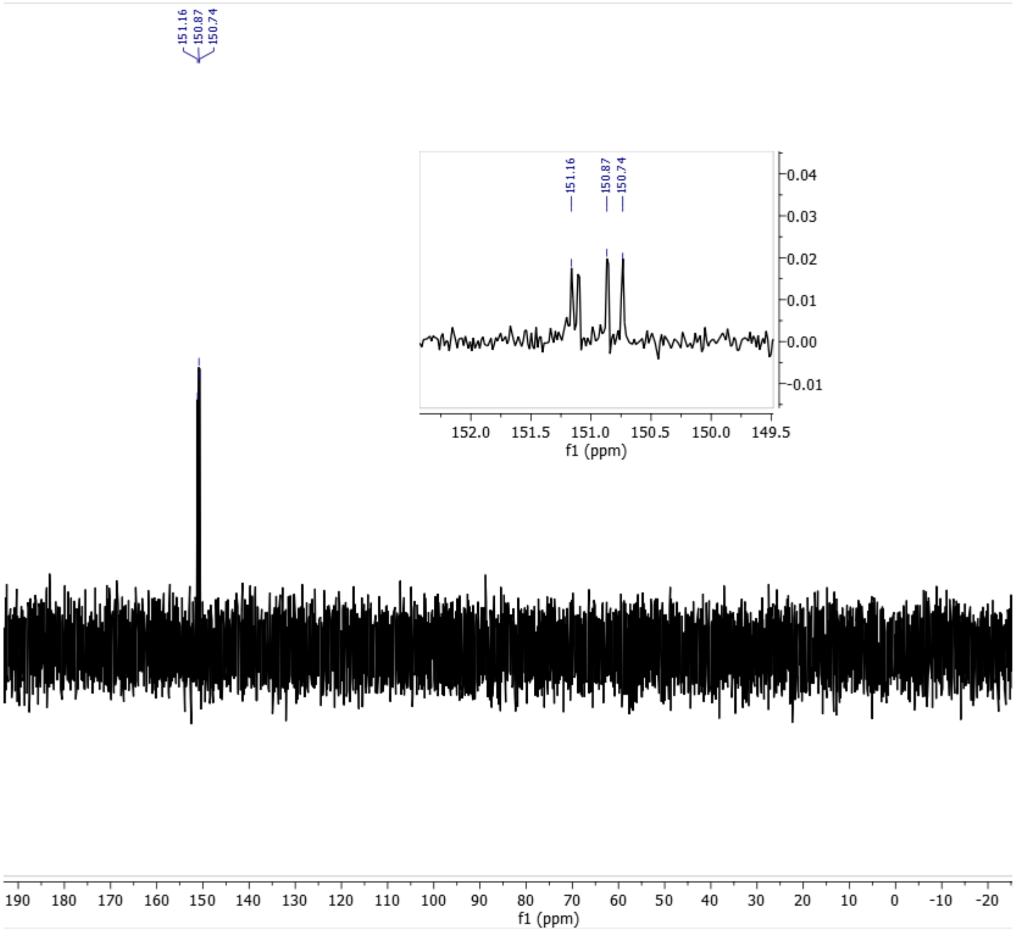

Figure S14: ^{31}P NMR spectrum (162 MHz, $CDCl_3$) of compound **8-C**.

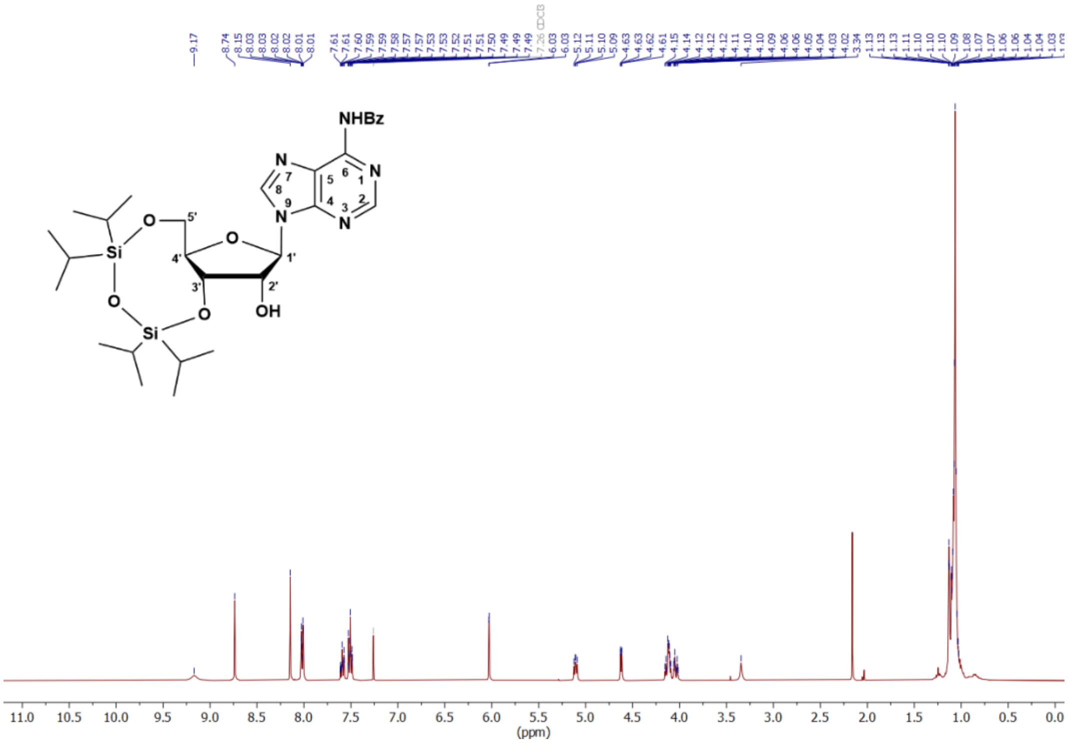

Figure S15.1: ^{1}H NMR spectrum (400 MHz, $CDCl_3$) of compound **2-A**.

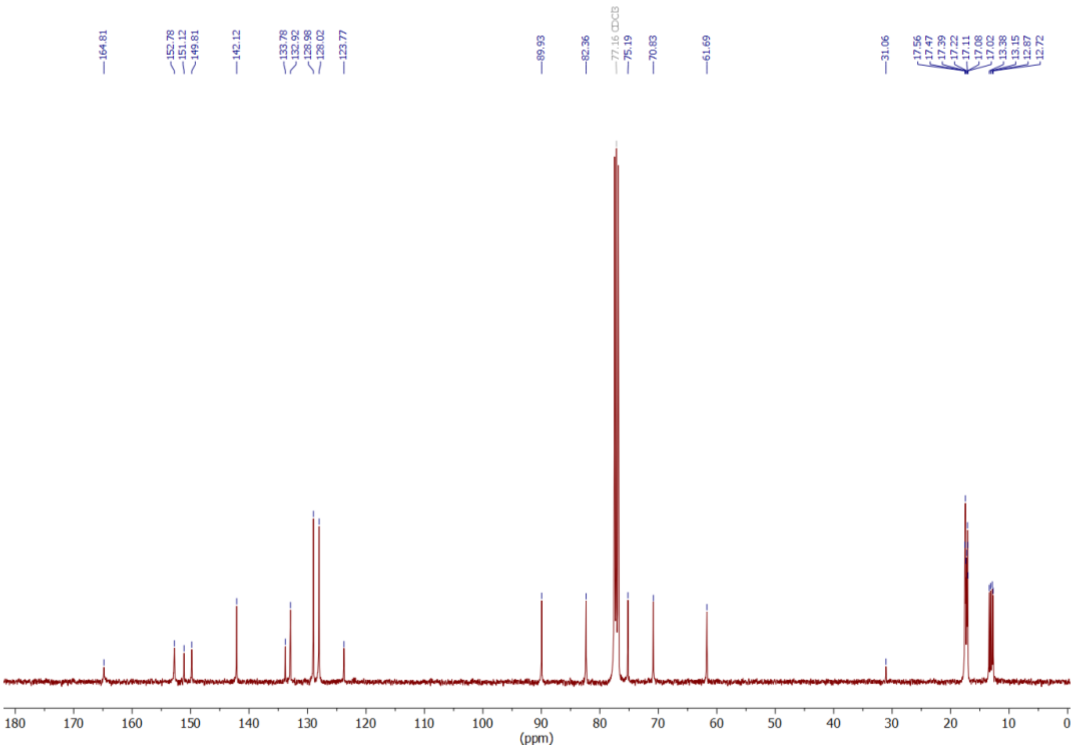

Figure S15.2: ^{13}C NMR spectrum (101 MHz, $CDCl_3$) of compound **2-A**.

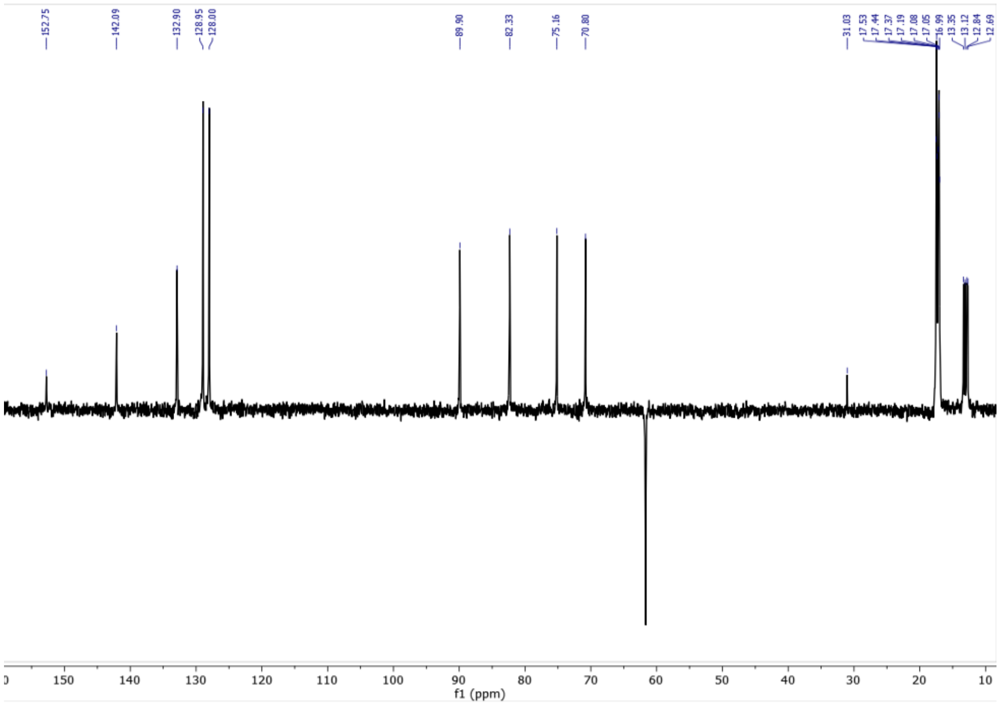

Figure S15.3: ^{13}C DEPT with decoupling NMR spectrum of compound **2-A**.

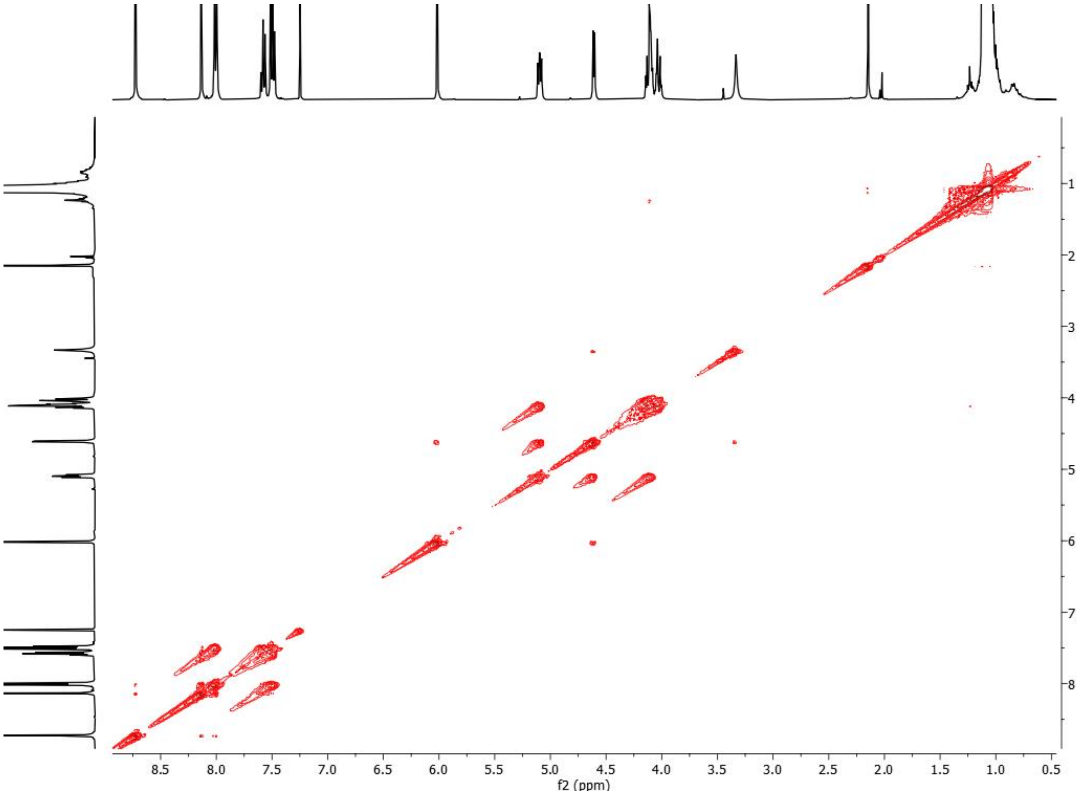

Figure S15.4: gCOSY spectra of compound **2-A**.

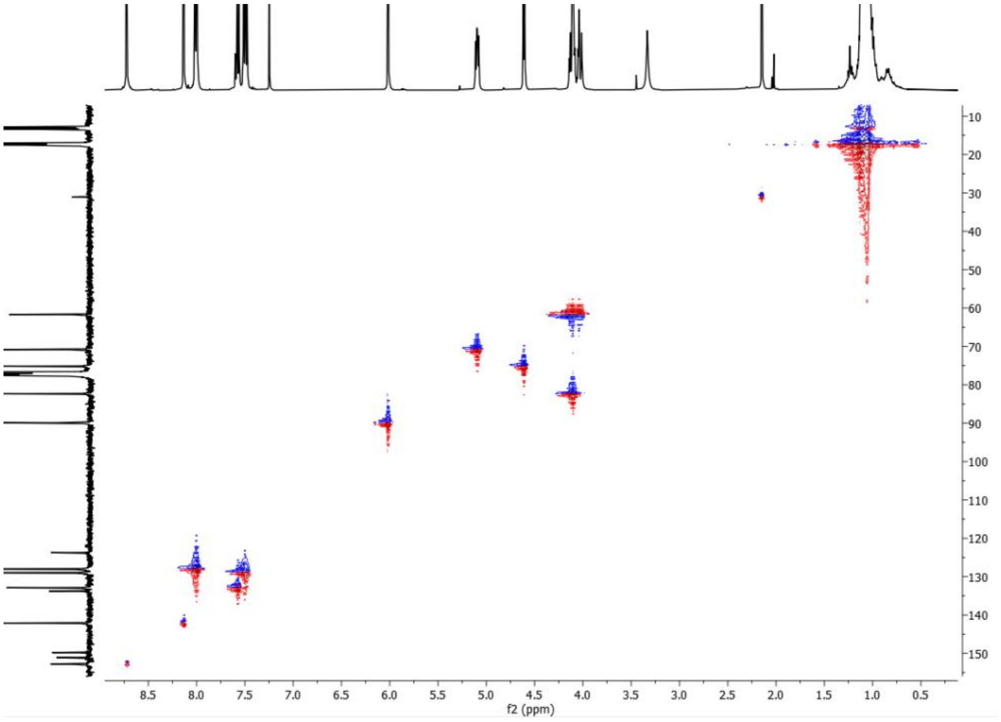

Figure S15.5: HSQC spectra of compound **2-A**.

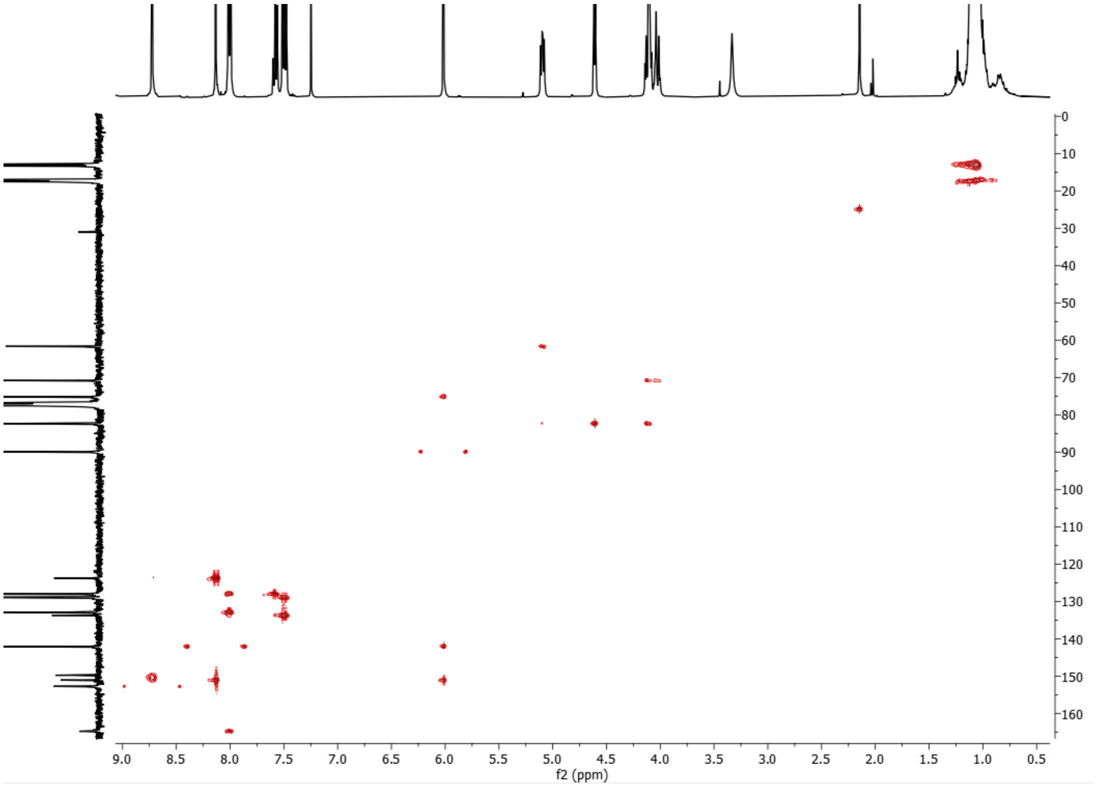

Figure S15.6: HMBC spectra of compound **2-A**.

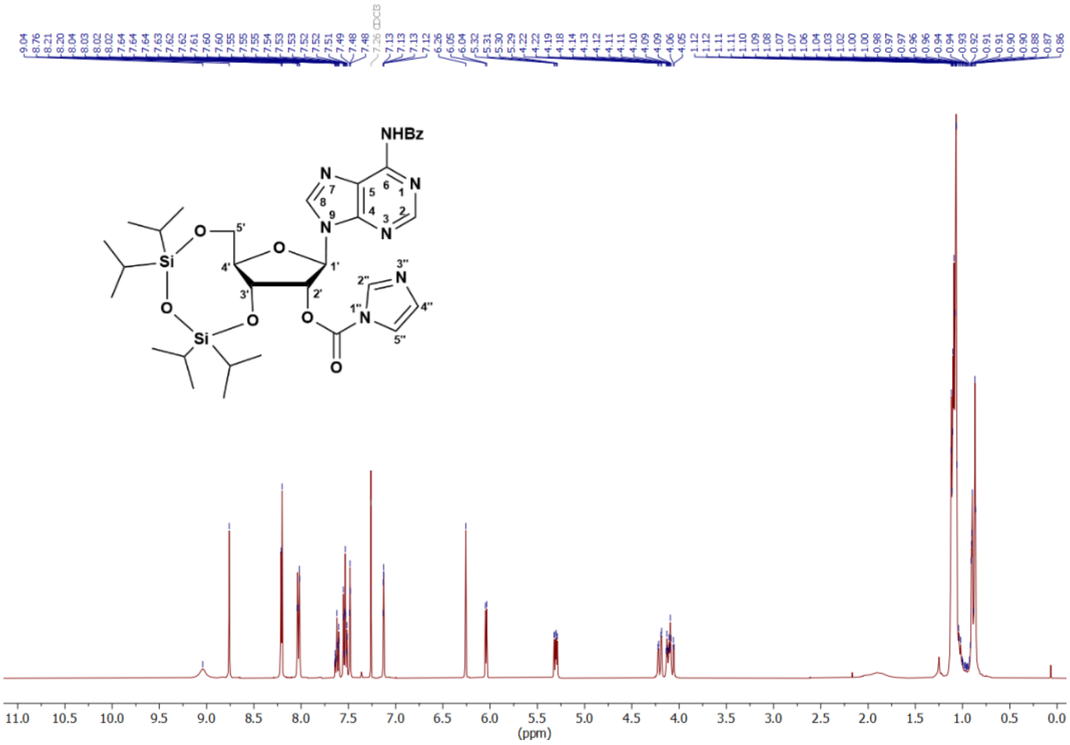

Figure S16.1: ^{1}H NMR spectrum (400 MHz, $CDCl_3$) of compound **3-A**.

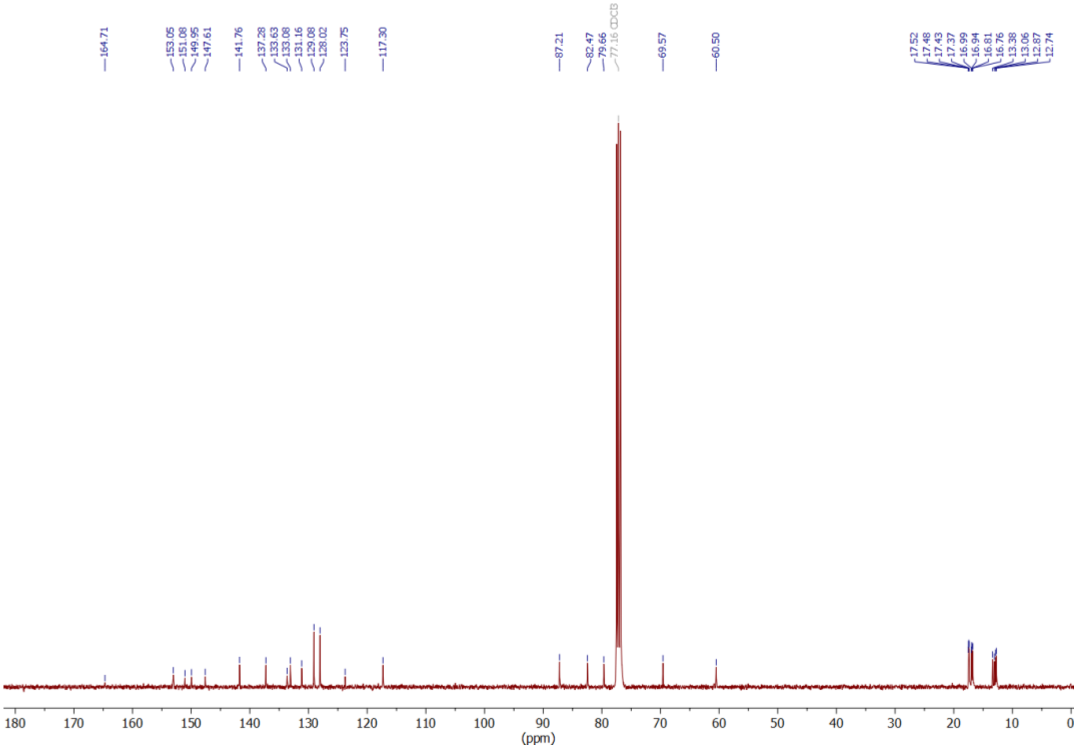

Figure S16.2: ^{13}C NMR spectrum (101 MHz, $CDCl_3$) of compound **3-A**.

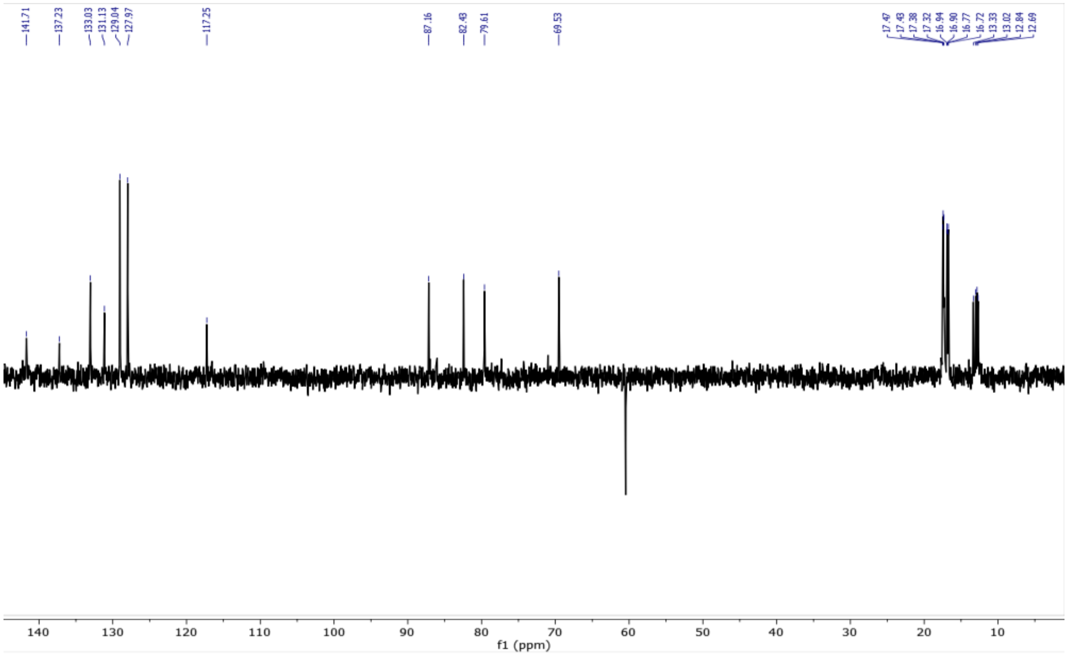

Figure S16.3: ^{13}C DEPT with decoupling NMR spectrum of compound **3-A**.

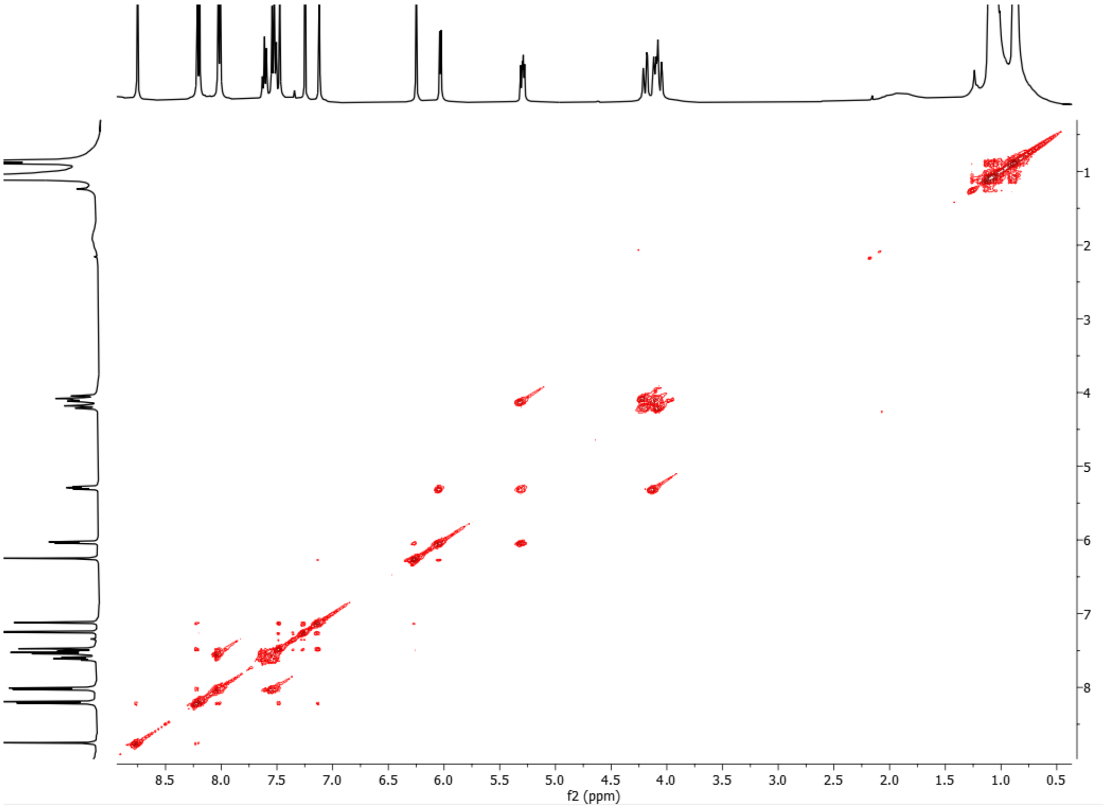

Figure S16.4: gCOSY spectra of compound **3-A**.

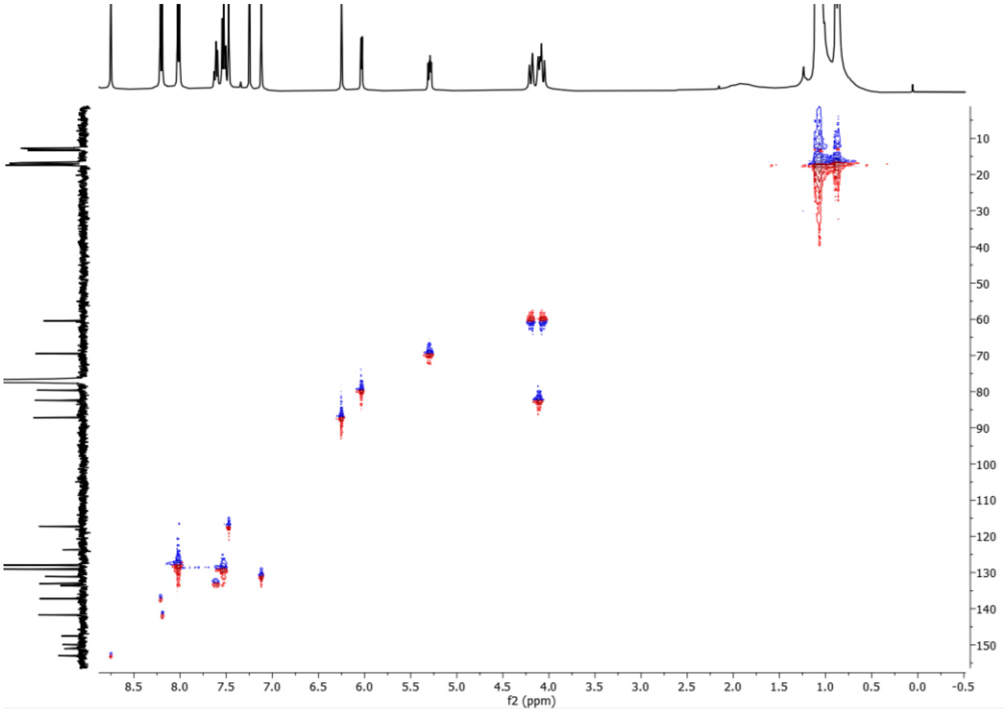

Figure S16.5: HSQC spectra of compound **3-A**.

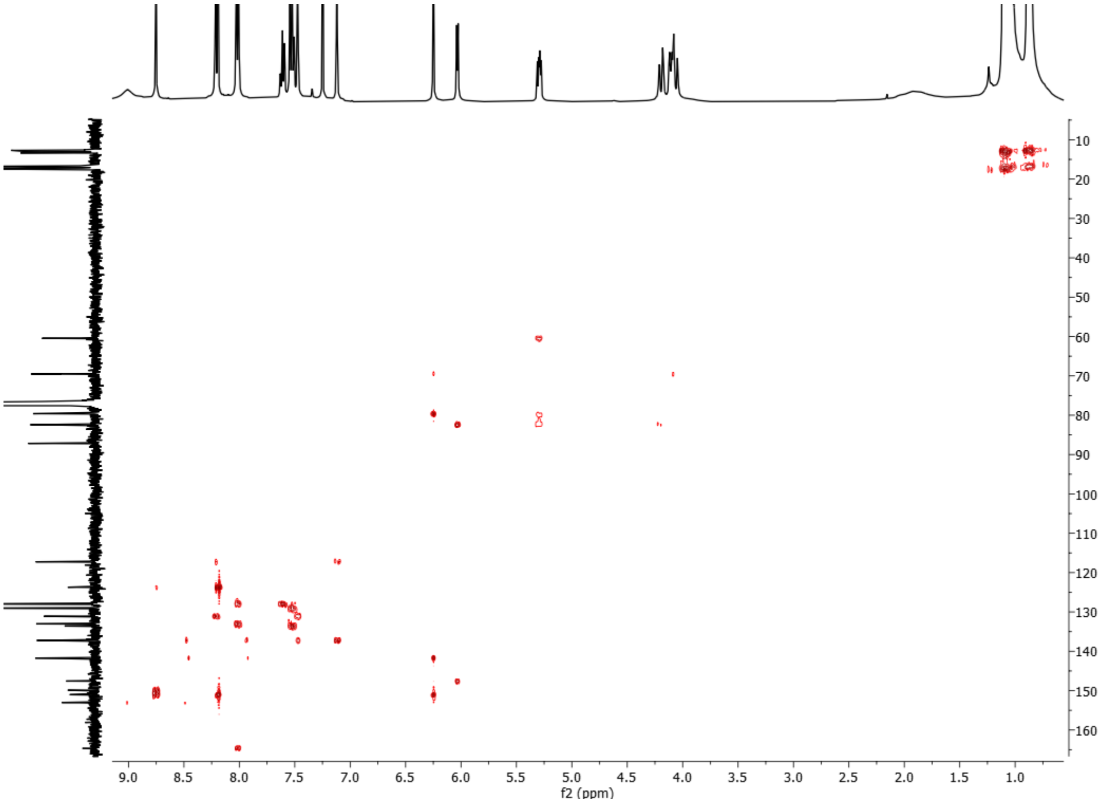

Figure S16.6: HMBC spectra of compound **3-A**.

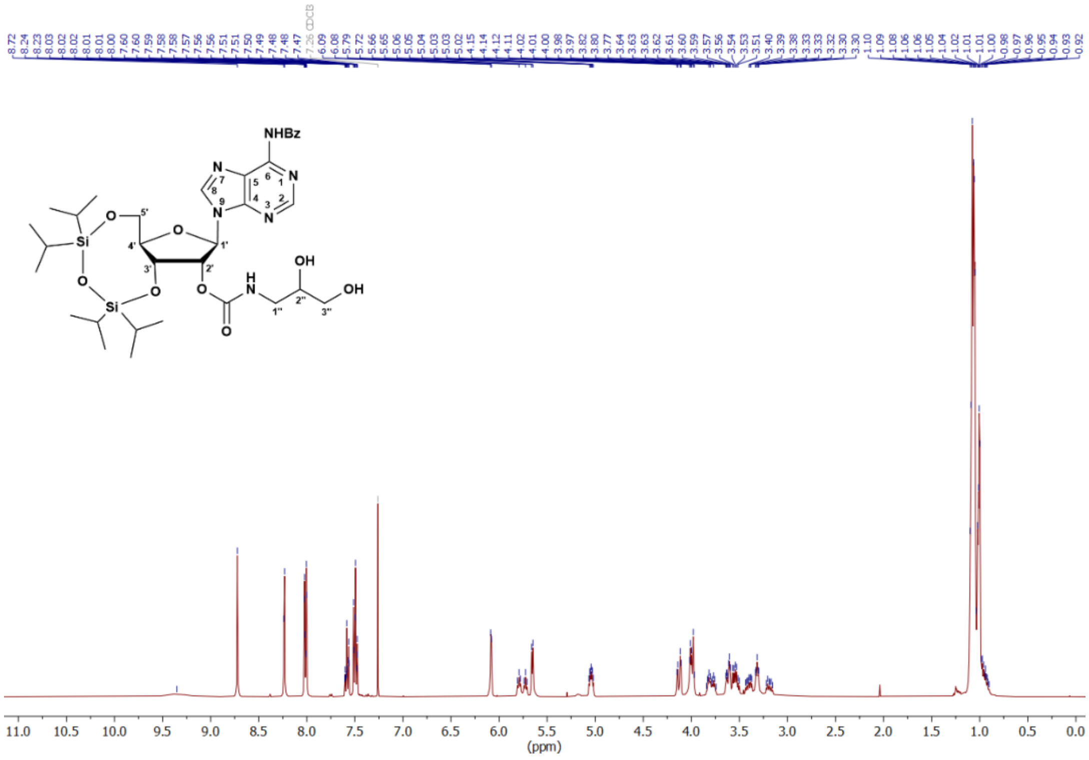

Figure S17.1: ^{1}H NMR spectrum (400 MHz, $CDCl_3$) of compound **4-A**.

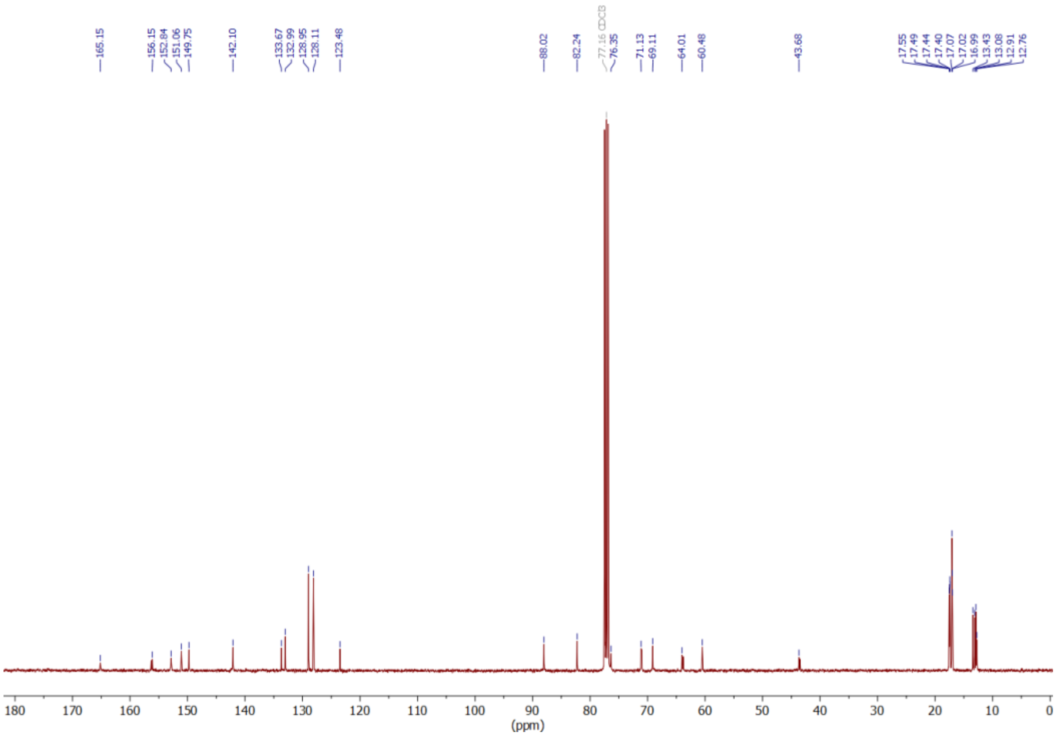

Figure S17.2: ^{13}C NMR spectrum (101 MHz, $CDCl_3$) of compound **4-A**.

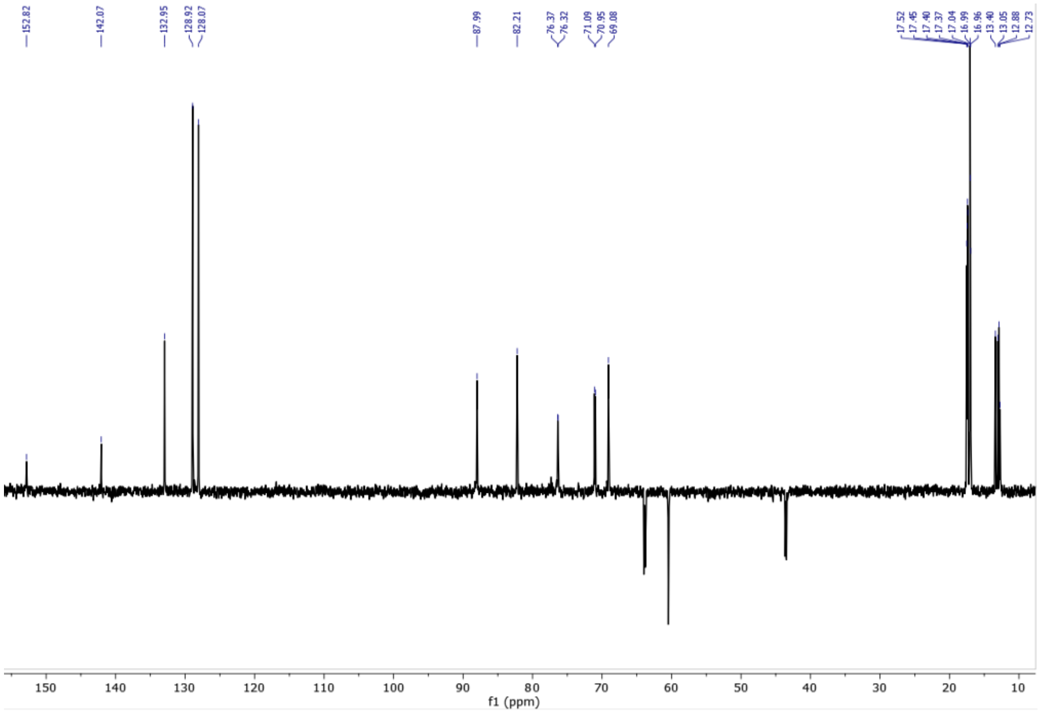

Figure S17.3: ^{13}C DEPT with decoupling NMR spectrum of compound **4-A**.

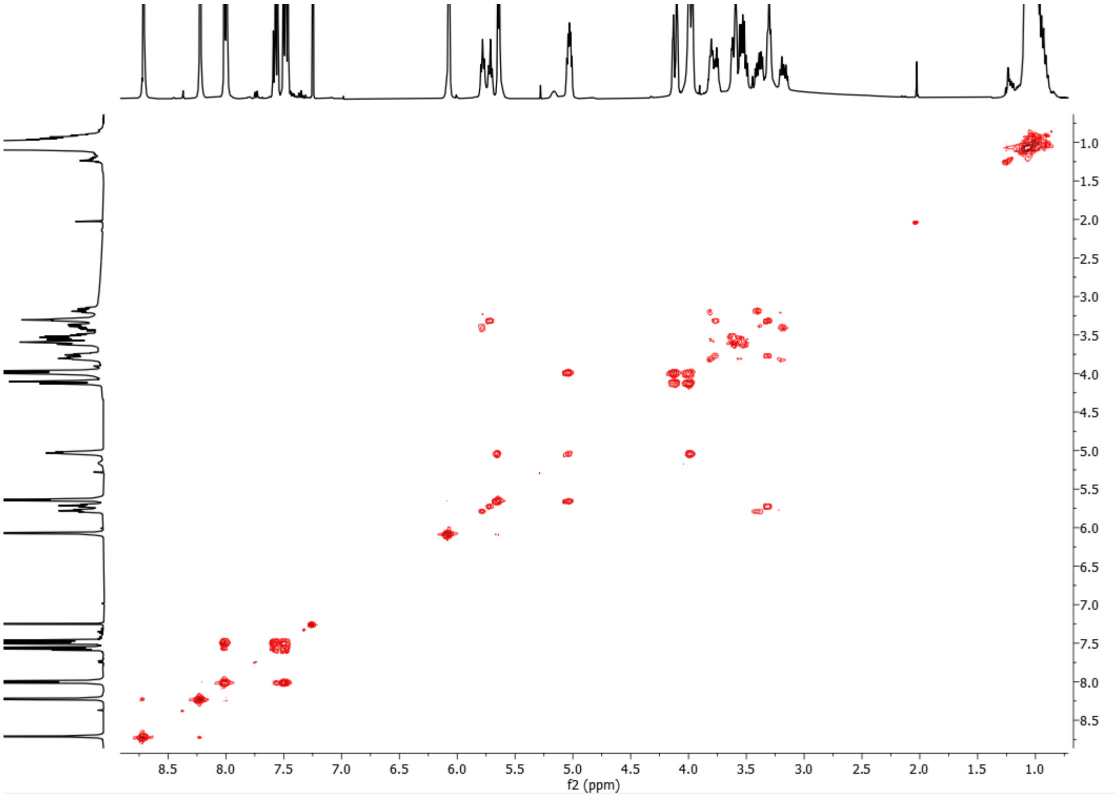

Figure S17.4: gCOSY spectra of compound **4-A**.

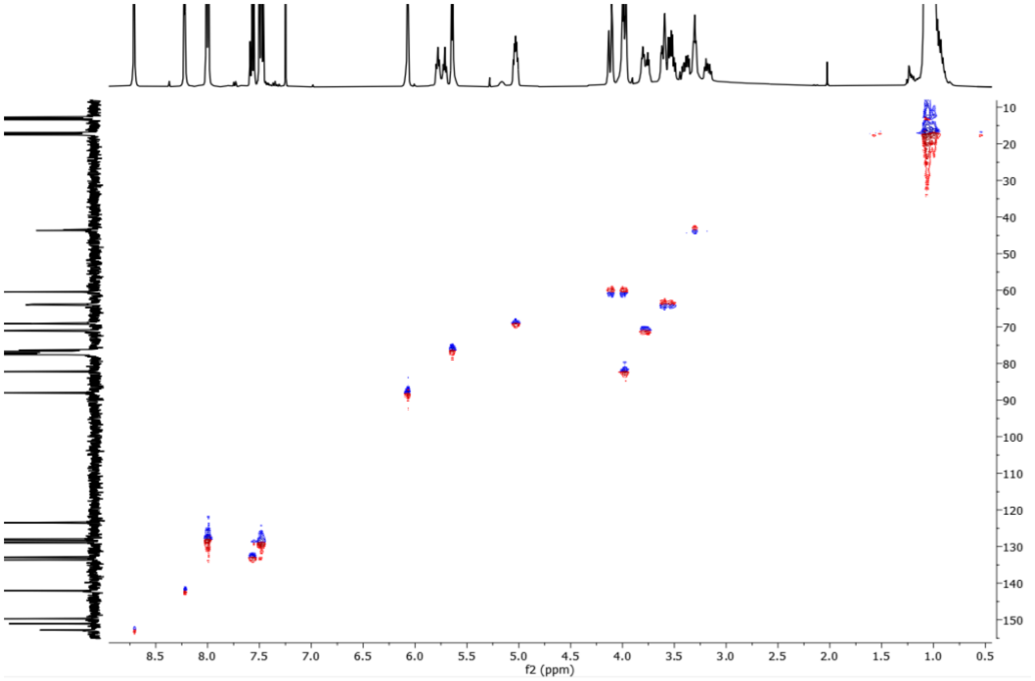

Figure S17.5: HSQC spectra of compound **4-A**.

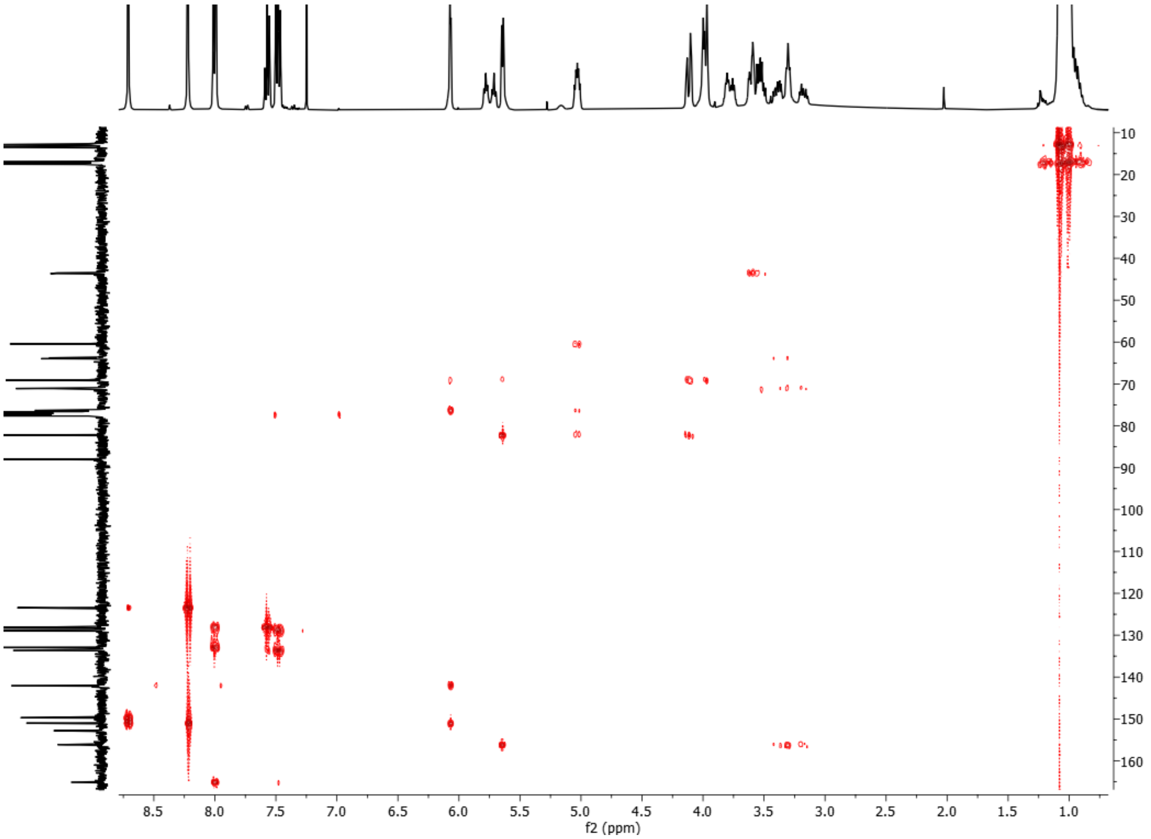

Figure S17.6: HMBC spectra of compound **4-A**.

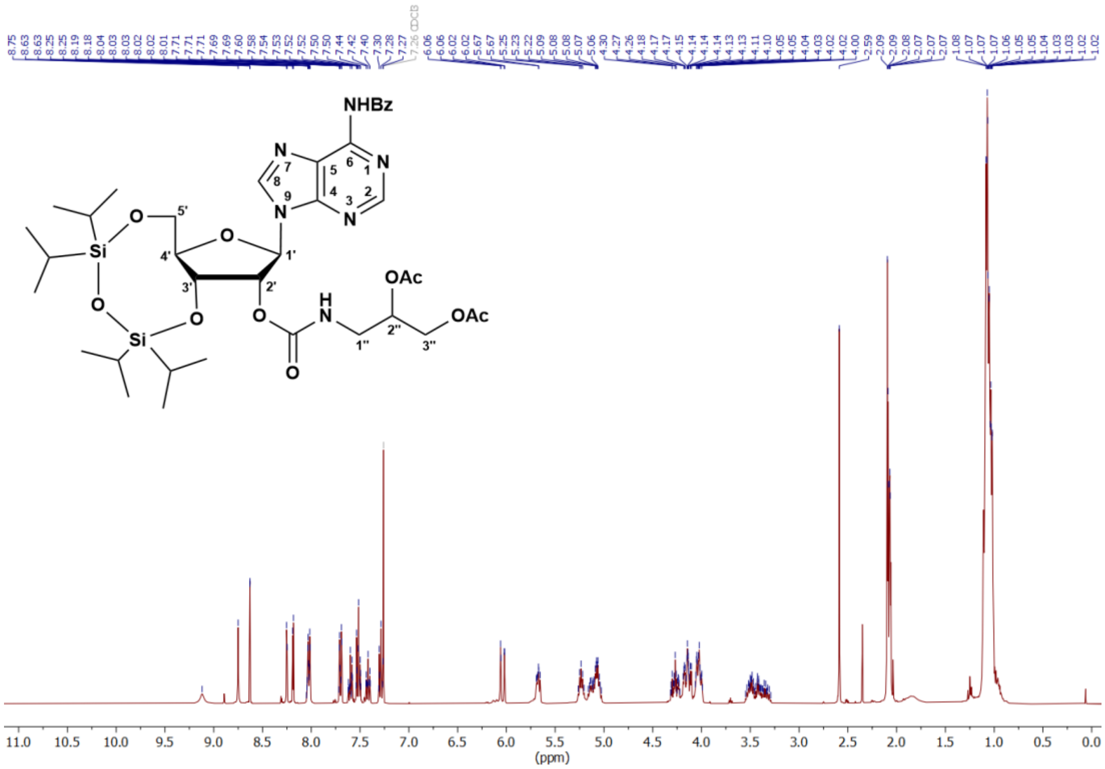

Figure S18.1: ^{1}H NMR spectrum (400 MHz, $CDCl_3$) of compound **5-A**.

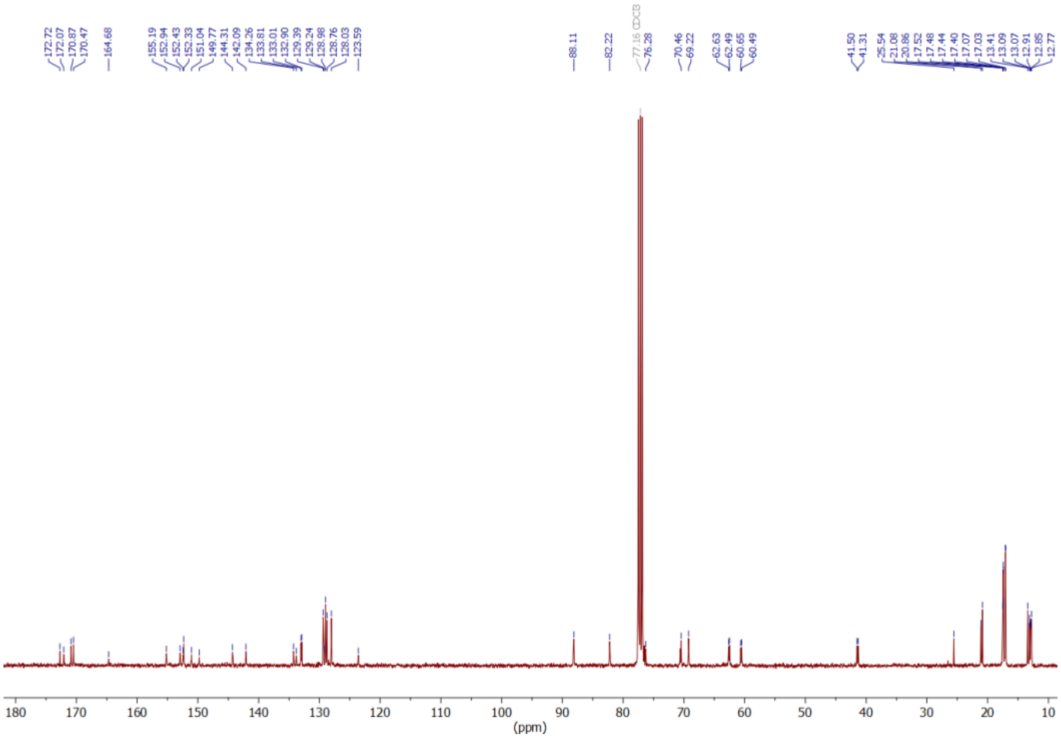

Figure S18.2: ^{13}C NMR spectrum (101 MHz, $CDCl_3$) of compound **5-A**.

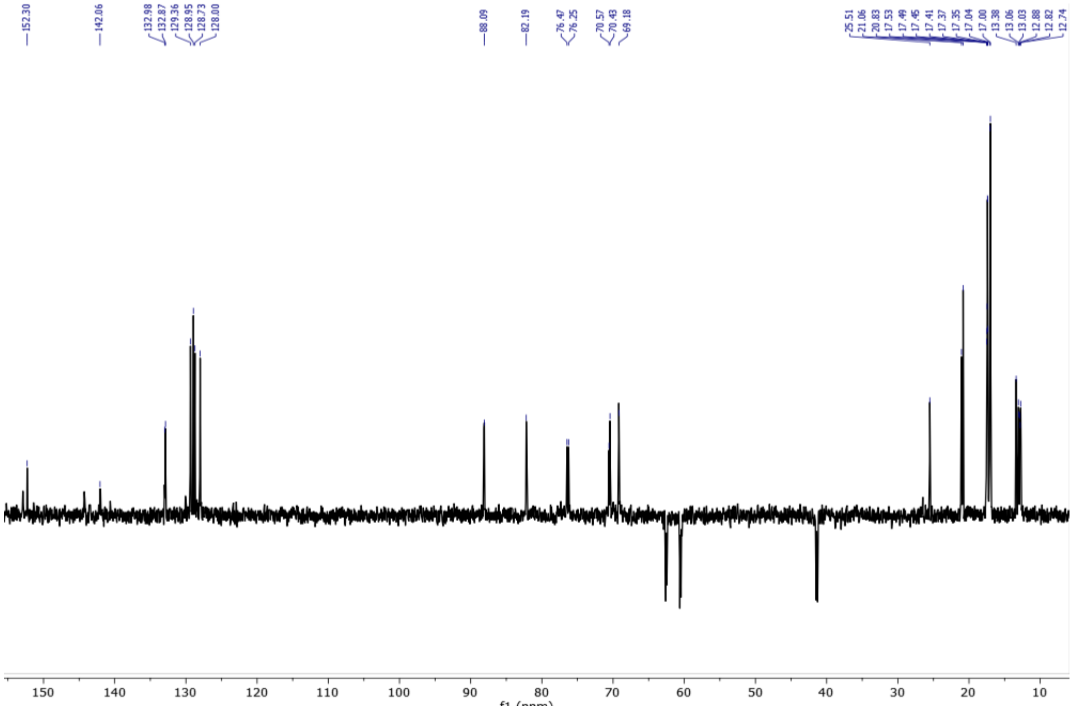

Figure S18.3: ^{13}C DEPT with decoupling NMR spectrum of compound **5-A**.

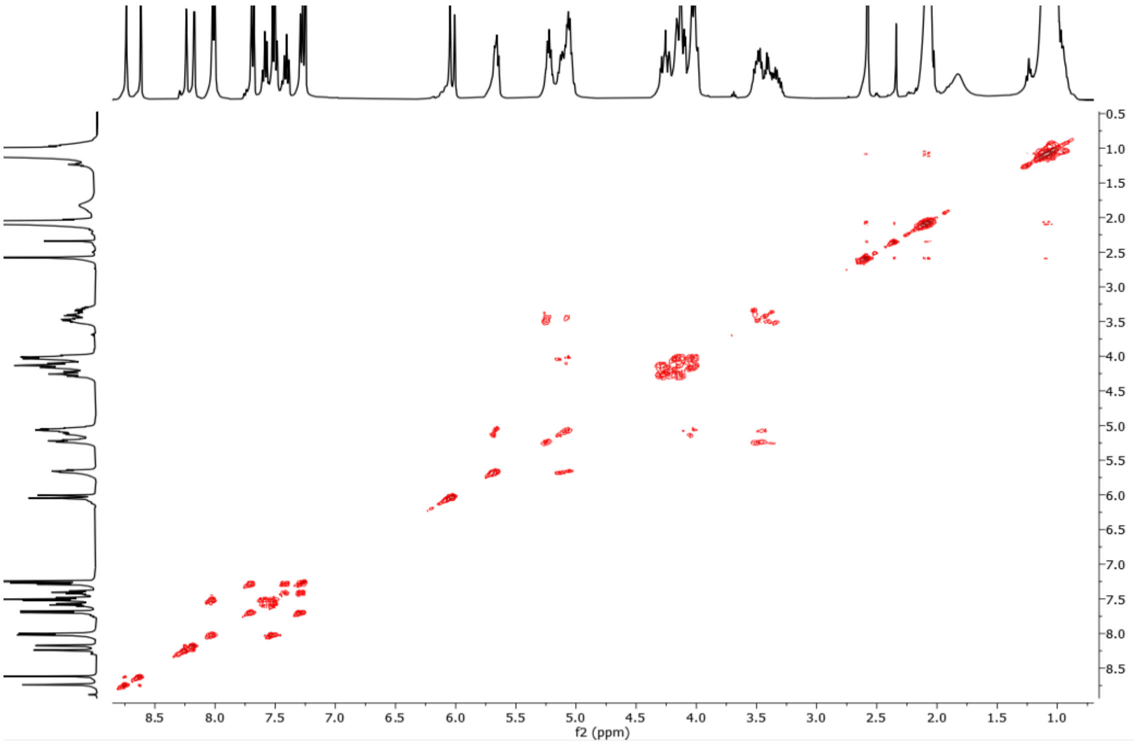

Figure S18.4: gCOSY spectra of compound **5-A**.

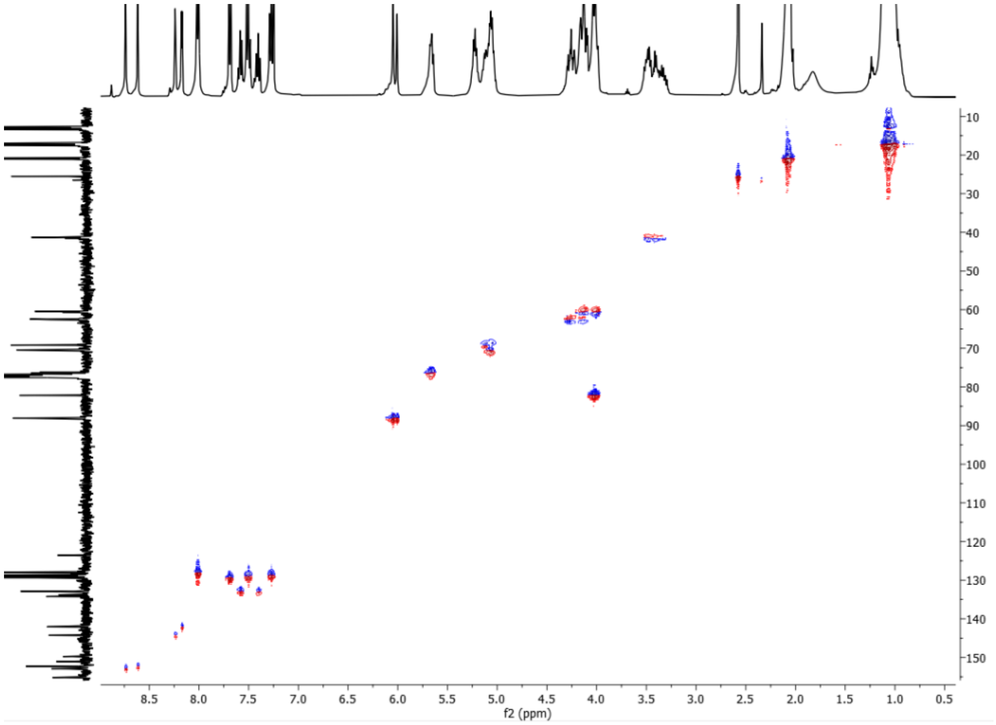

Figure S18.5: HSQC spectra of compound **5-A**.

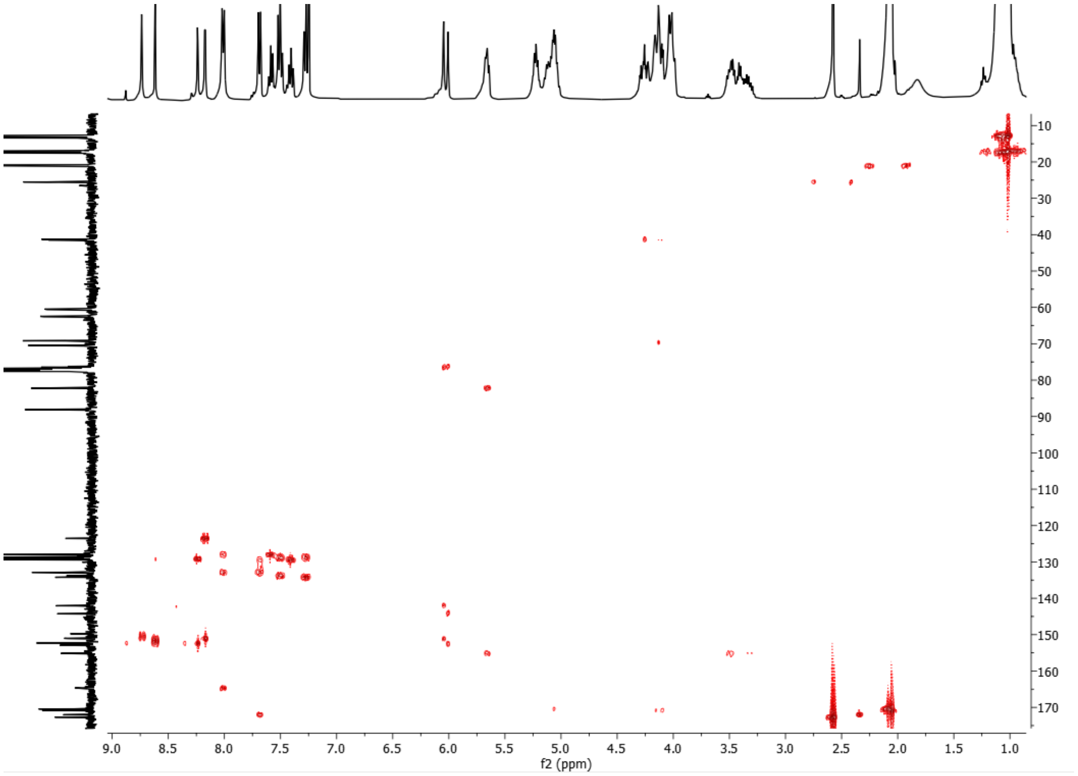

Figure S18.6: HMBC spectra of compound **5-A**.

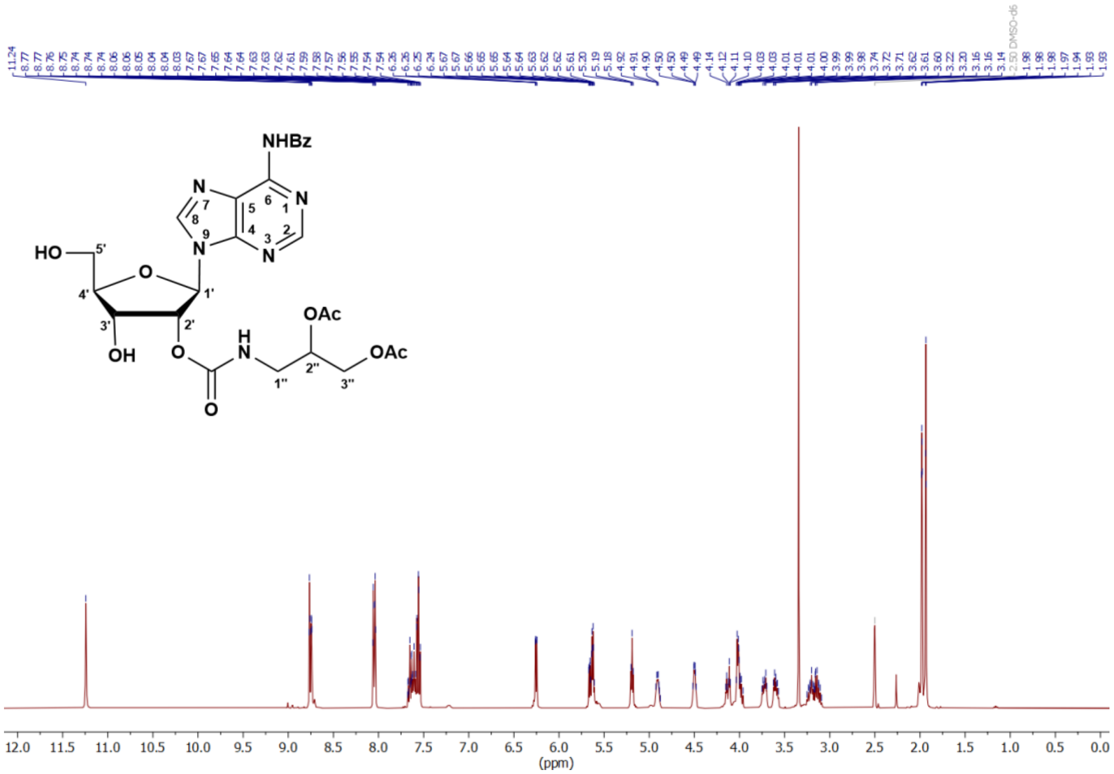

Figure S19.1: ^{1}H NMR spectrum (400 MHz, DMSO-d$_6$) of compound **6-A**.

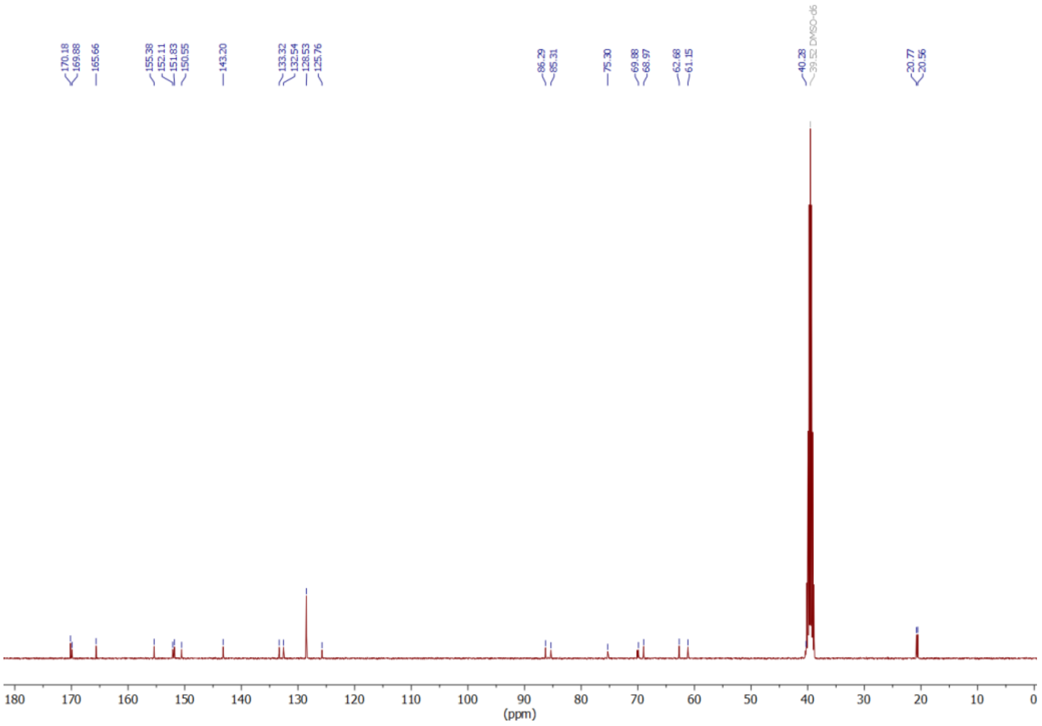

Figure S19.2: ^{13}C NMR spectrum (101 MHz, DMSO-d$_6$) of compound **6-A**.

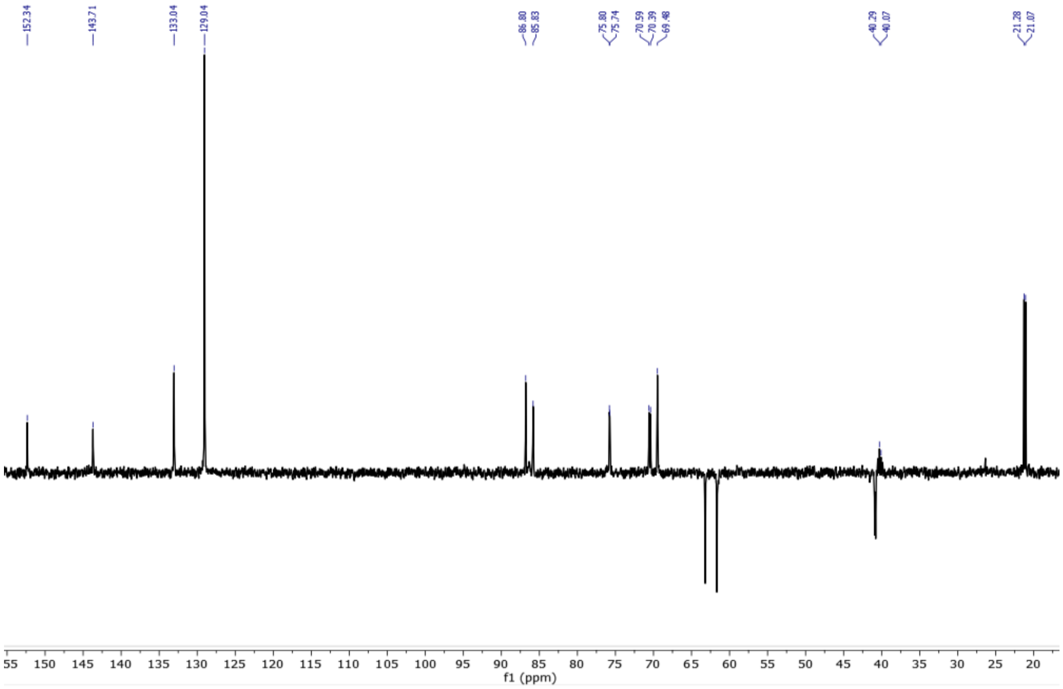

Figure S19.3: ^{13}C DEPT with decoupling NMR spectrum of compound **6-A**.

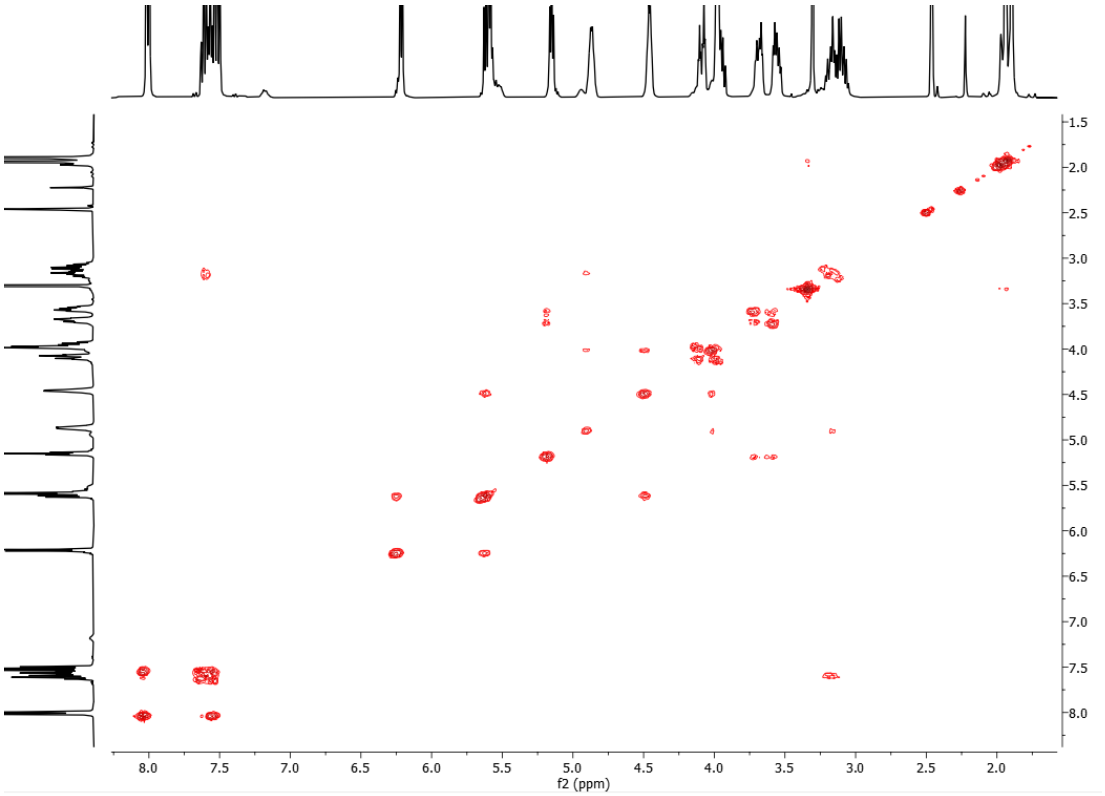

Figure S19.4: gCOSY spectra of compound **6-A**.

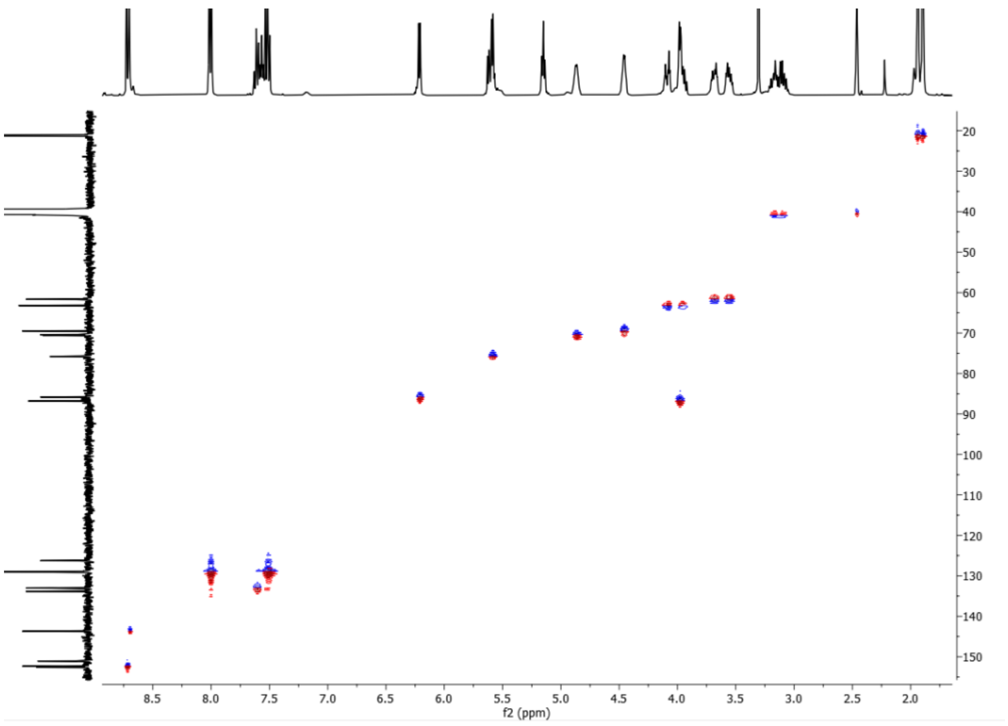

Figure S19.5: HSQC spectra of compound **6-A**.

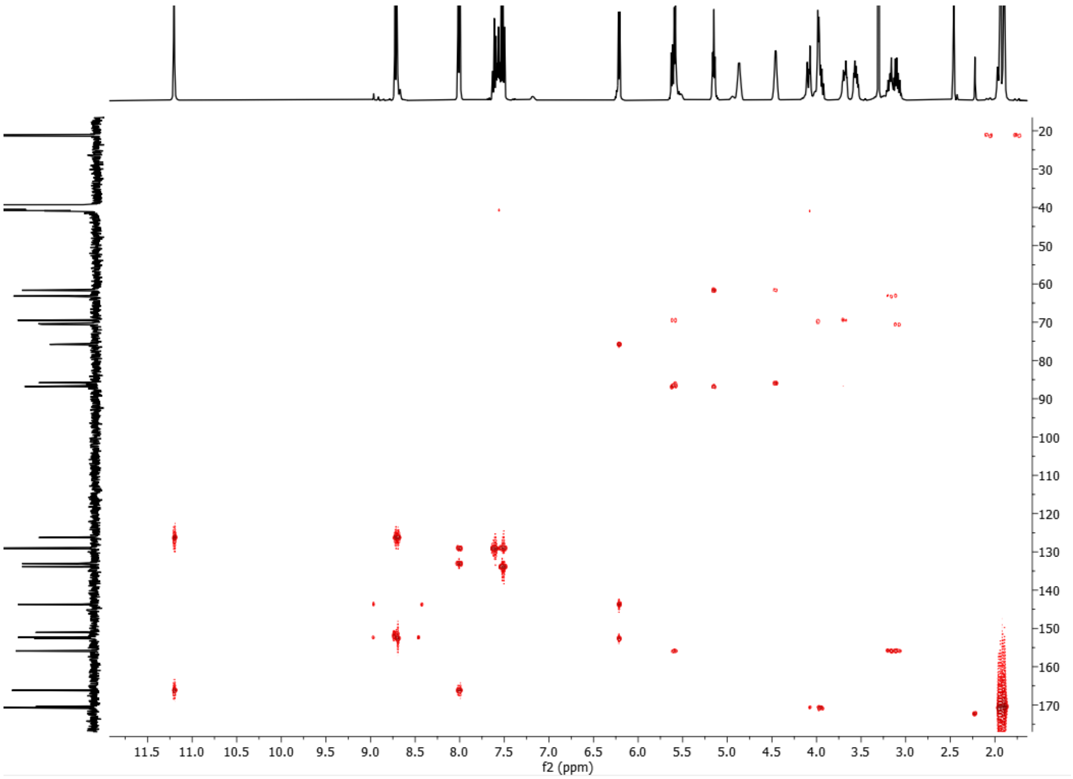

Figure S19.6: HMBC spectra of compound **6-A**.

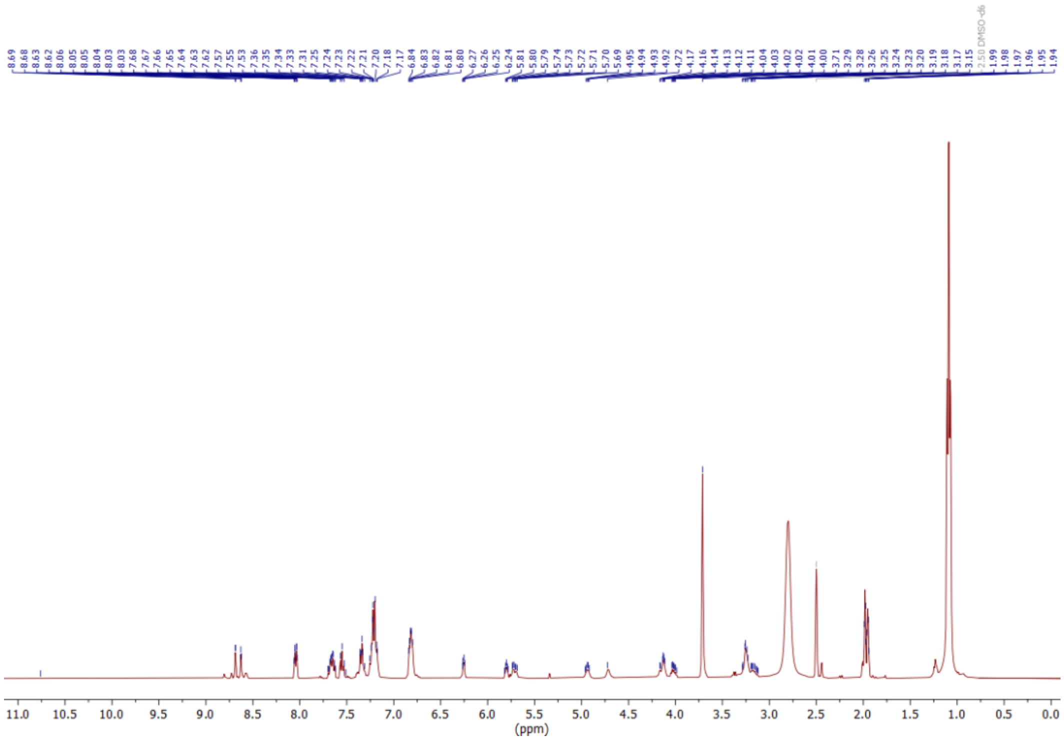

Figure S20.1: ^{1}H NMR spectrum (400 MHz, DMSO-d_6) of compound **7-A**.

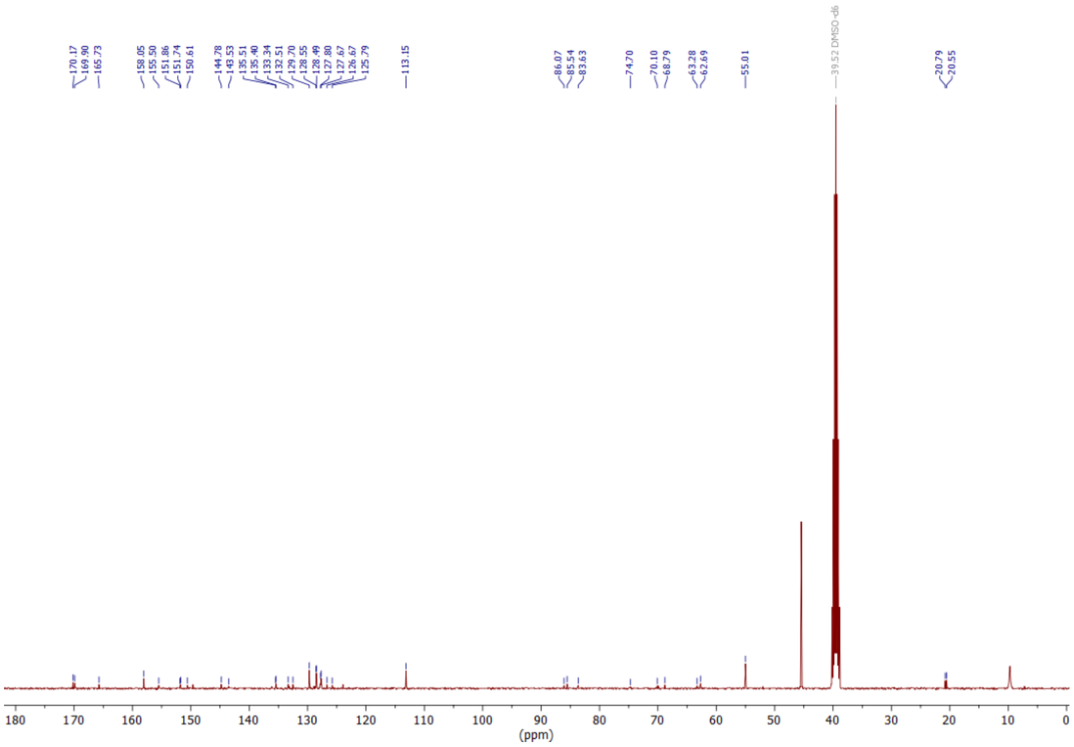

Figure S20.2: ^{13}C NMR spectrum (101 MHz, DMSO-d_6) of compound **7-A**.

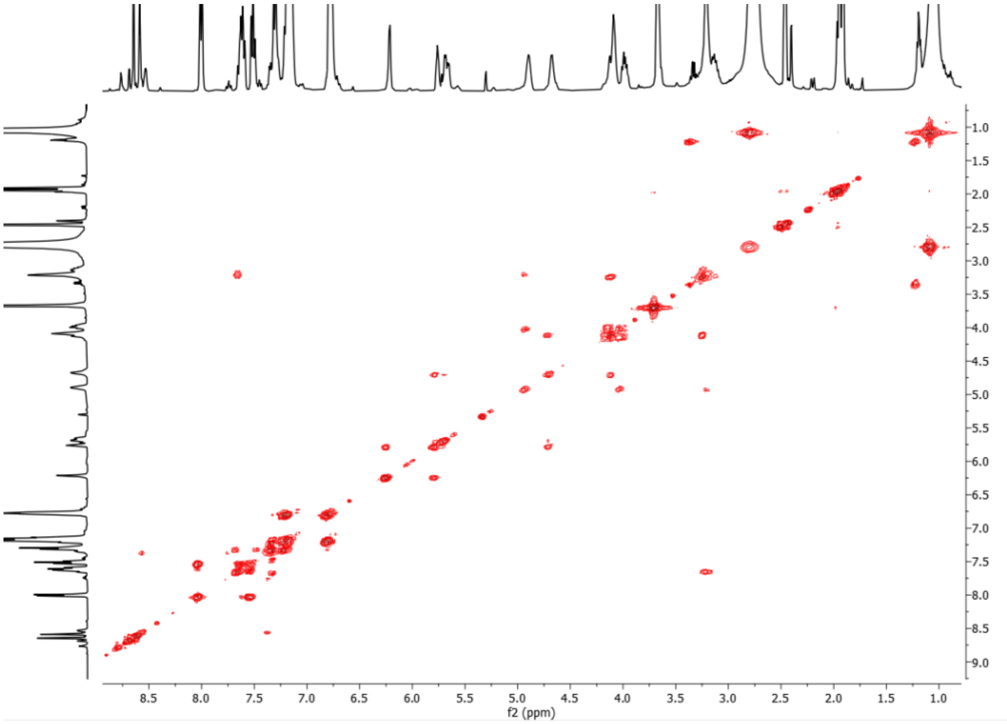

Figure S20.3: gCOSY spectra of compound **7-A**.

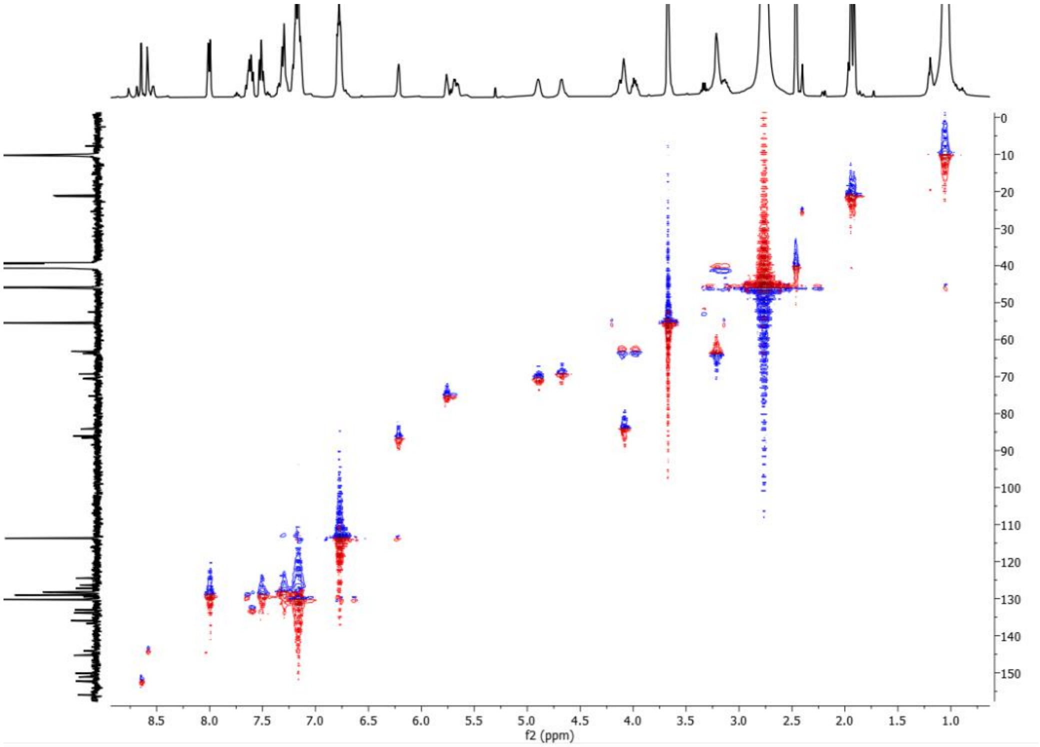

Figure S20.4: HSQC spectra of compound **7-A**.

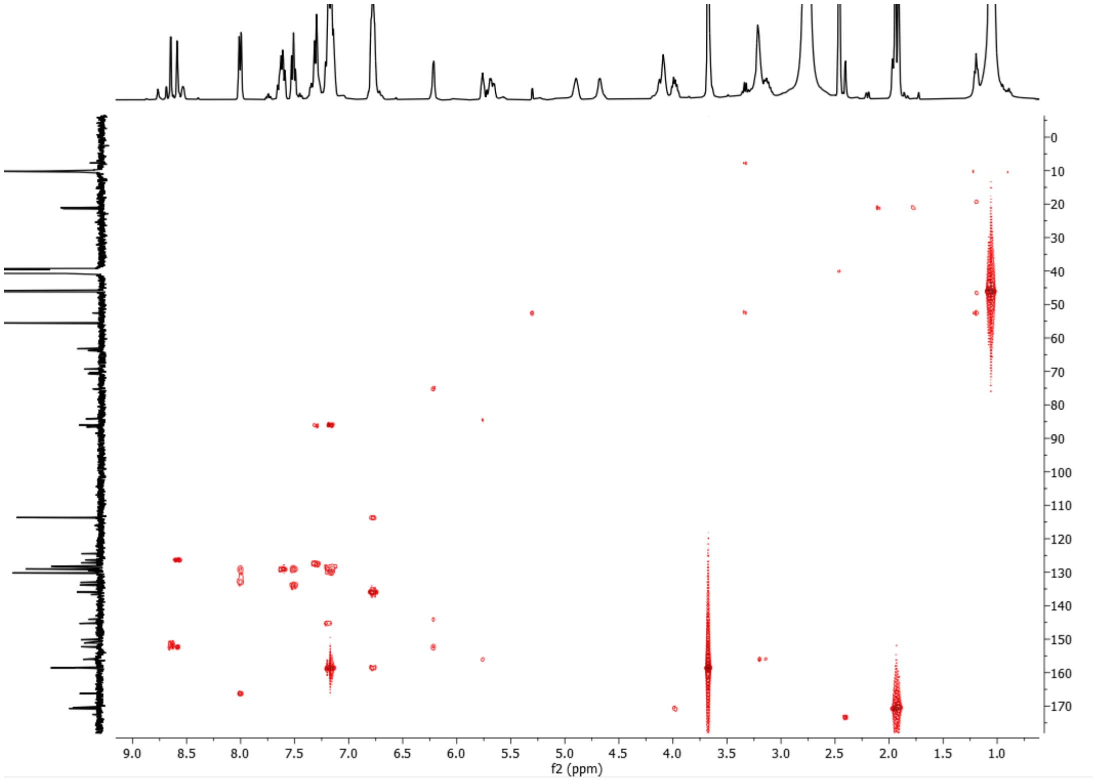

Figure S20.5: HMBC spectra of compound **7-A**.

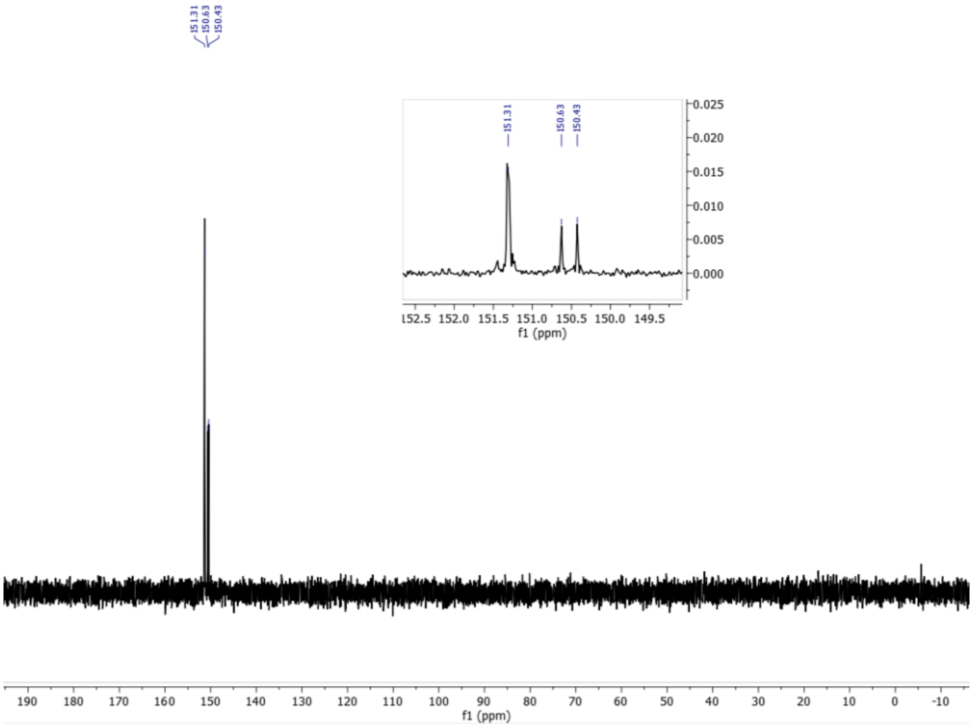

Figure S21: ^{31}P NMR spectrum (162 MHz, $CDCl_3$) of compound **8-A**.

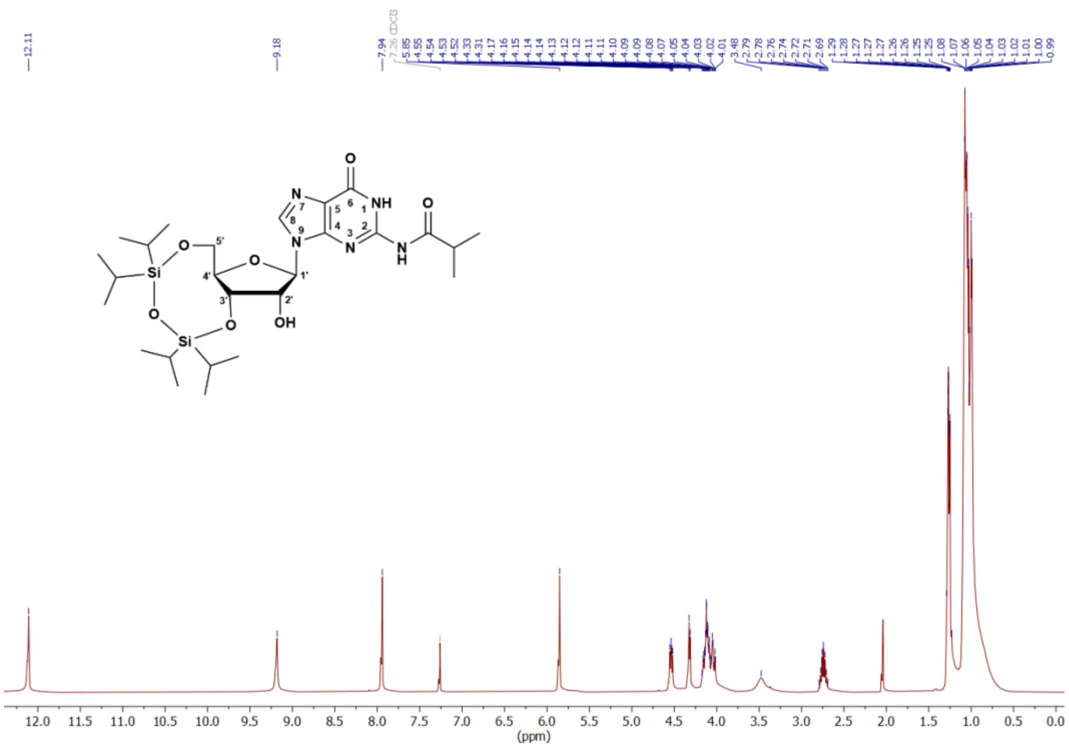

Figure S22.1: ^{1}H NMR spectrum (400 MHz, $CDCl_3$) of compound **2-G**.

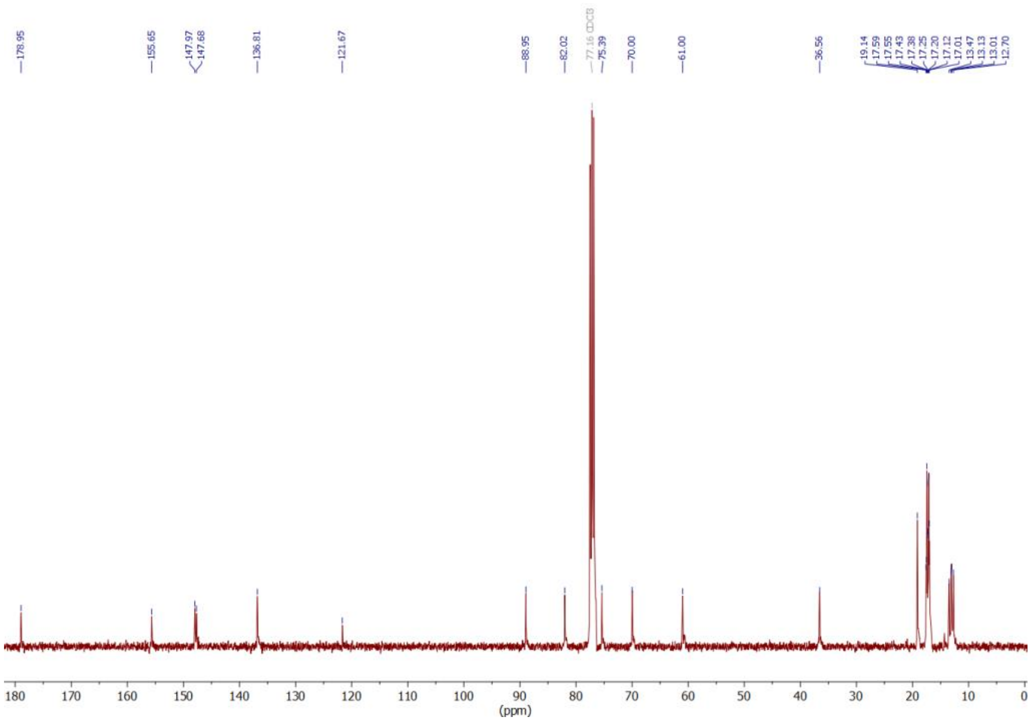

Figure S22.2: ^{13}C NMR spectrum (101 MHz, $CDCl_3$) of compound **2-G**.

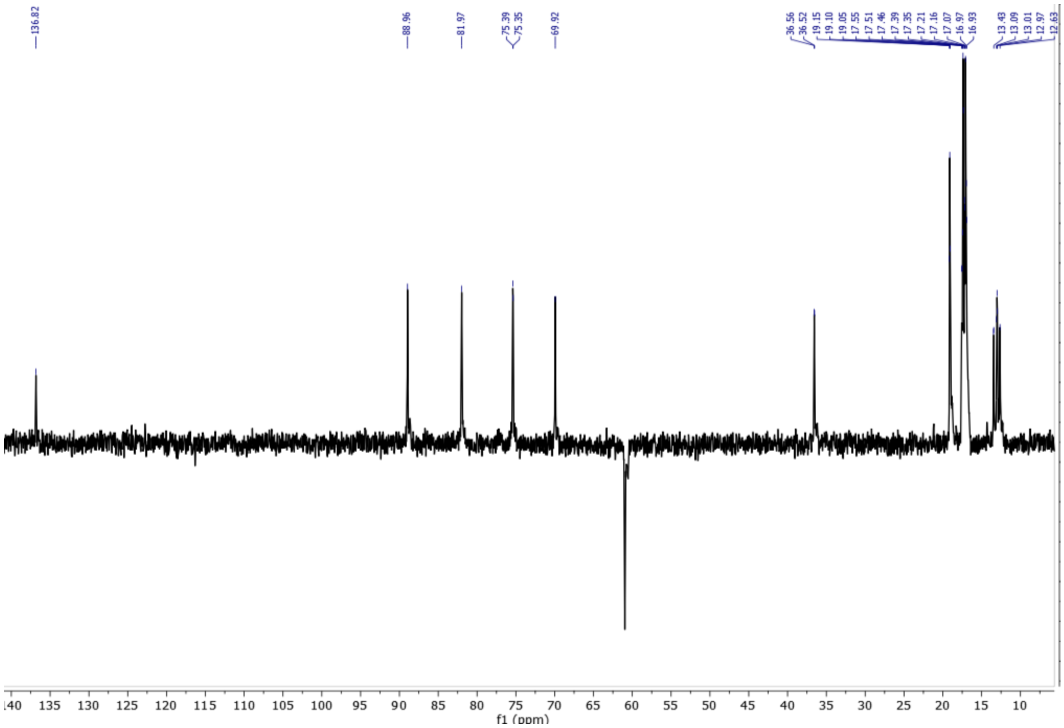

Figure S22.3: ^{13}C DEPT with decoupling NMR spectrum of compound **2-G**.

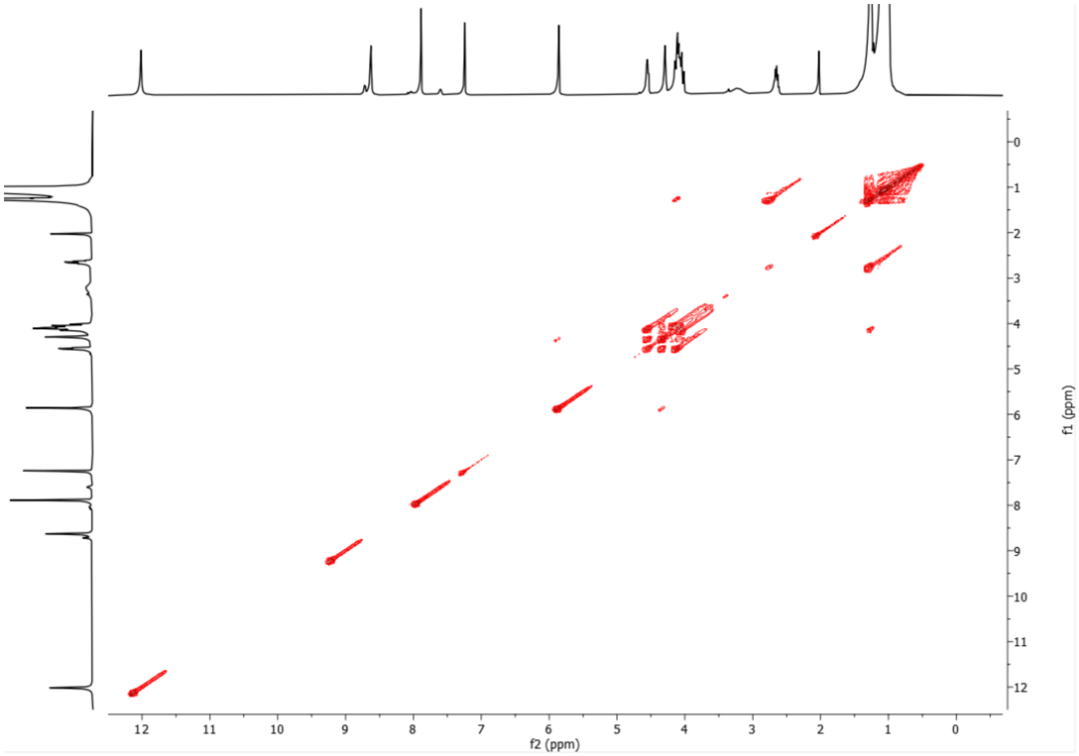

Figure S22.4: gCOSY spectra of compound **2-G**.

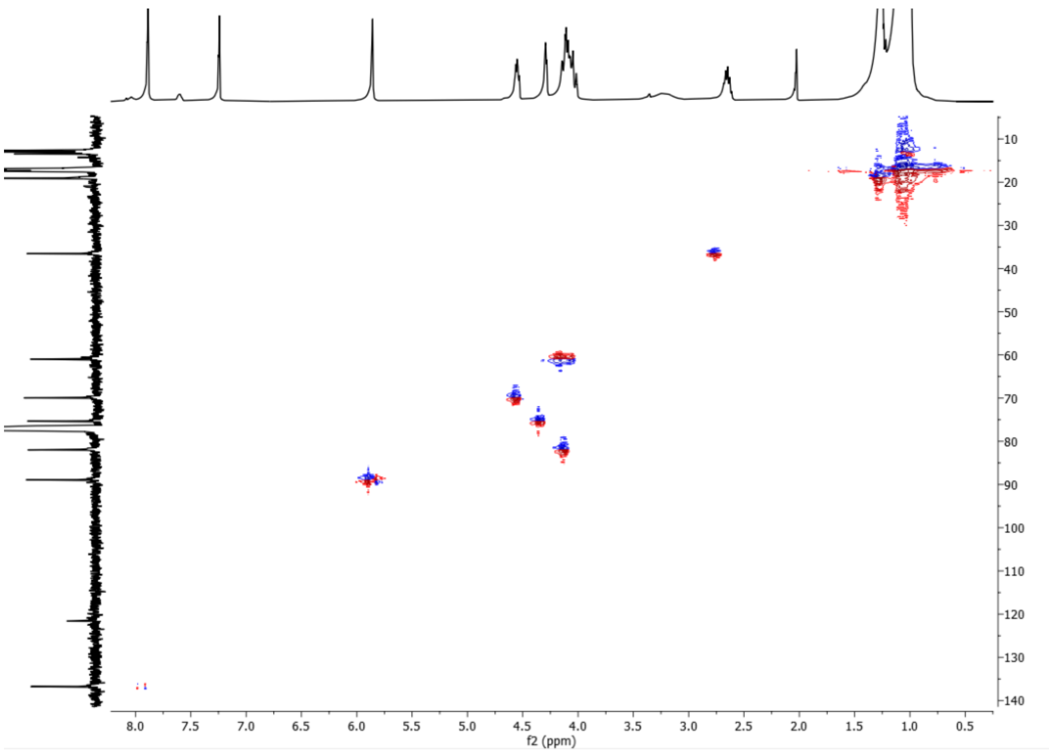

Figure S22.5: HSQC spectra of compound **2-G**.

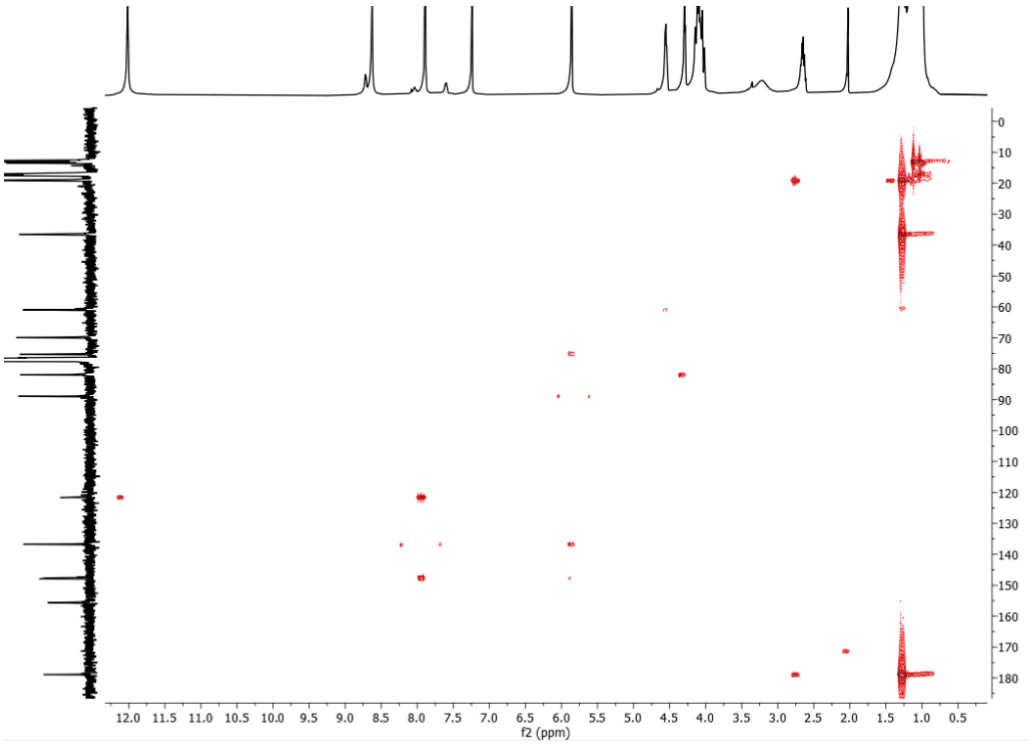

Figure S22.6: HMBC spectra of compound **2-G**.

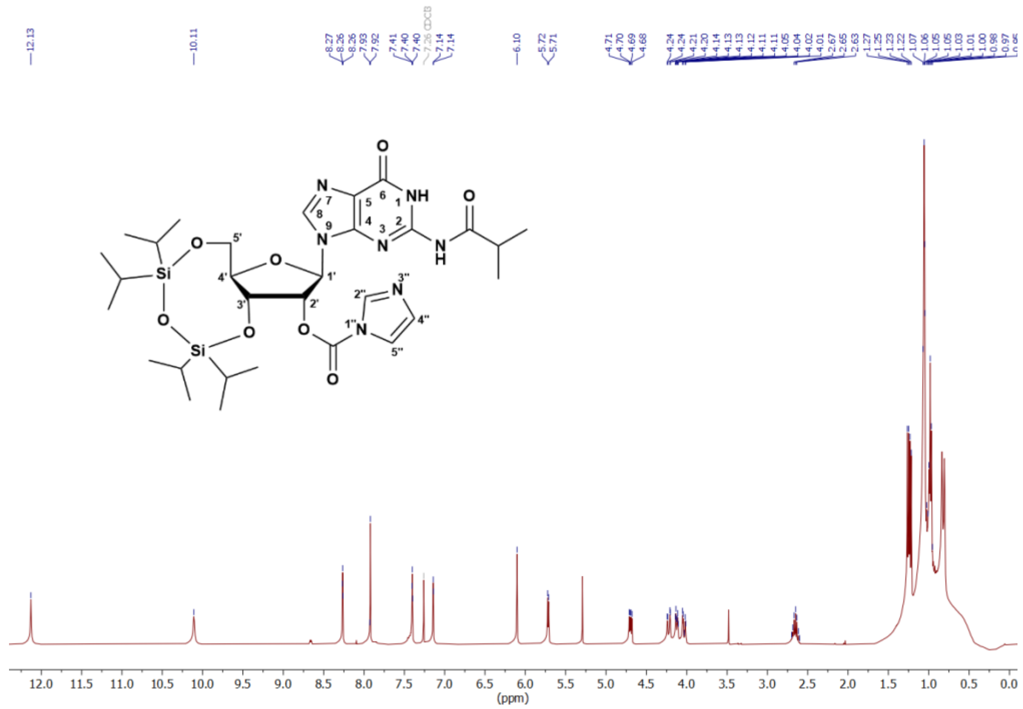

Figure S23.1: ^{1}H NMR spectrum (400 MHz, $CDCl_3$) of compound **3-G**.

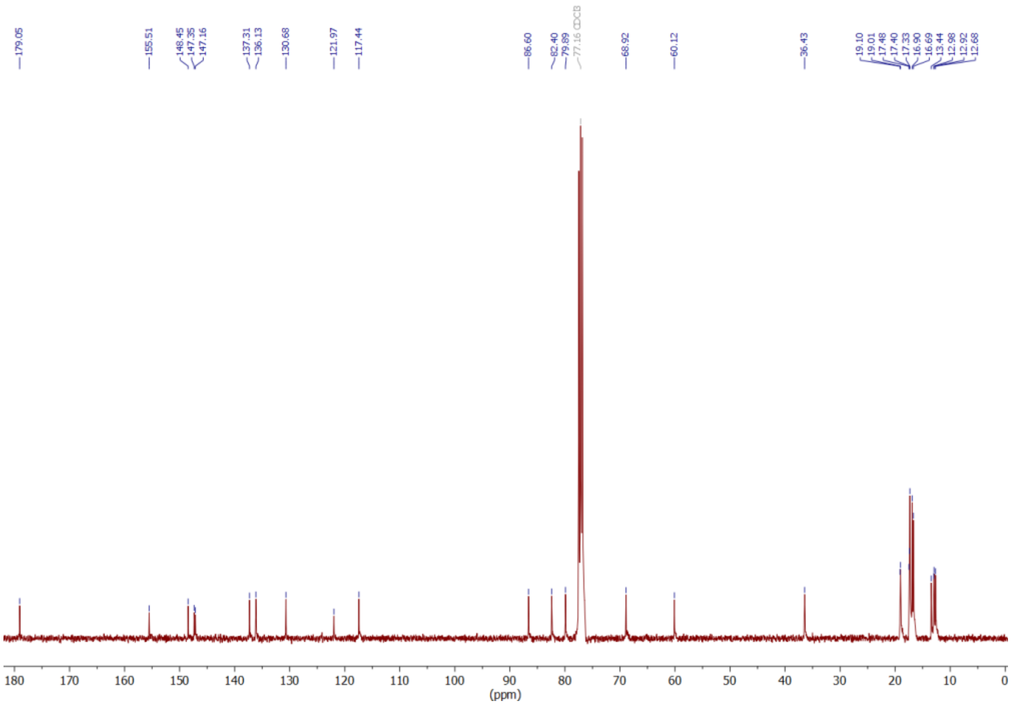

Figure S23.2: ^{13}C NMR spectrum (101 MHz, $CDCl_3$) of compound **3-G**.

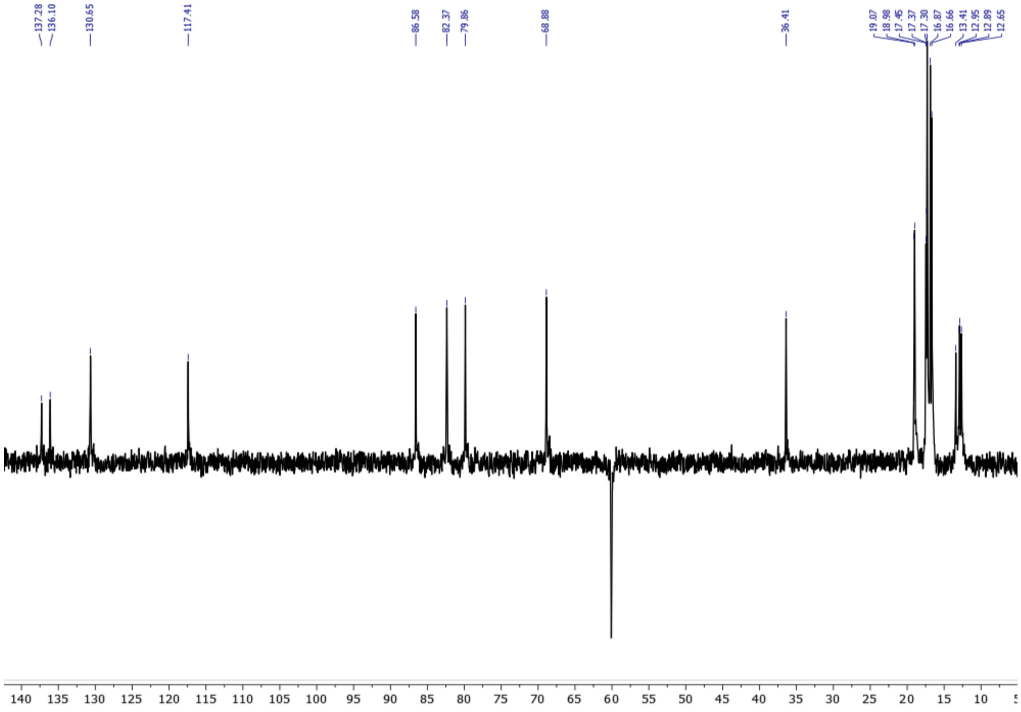

Figure S23.3: ^{13}C DEPT with decoupling NMR spectrum of compound **3-G**.

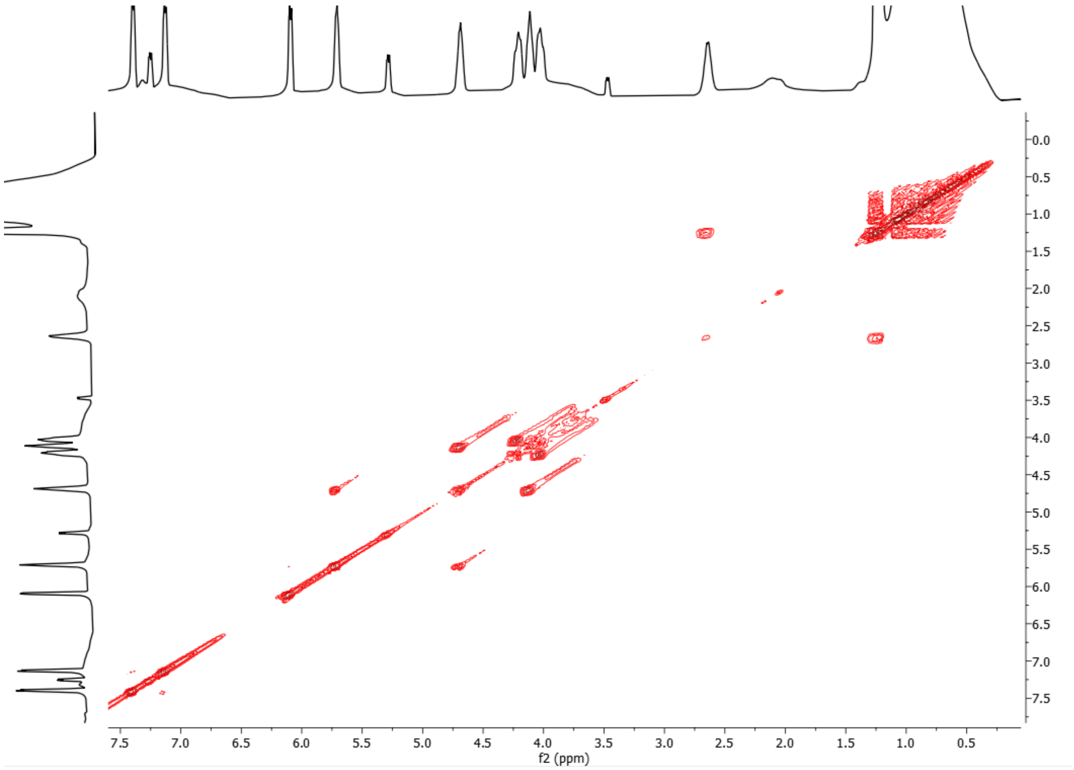

Figure S23.4: gCOSY spectra of compound **3-G**.

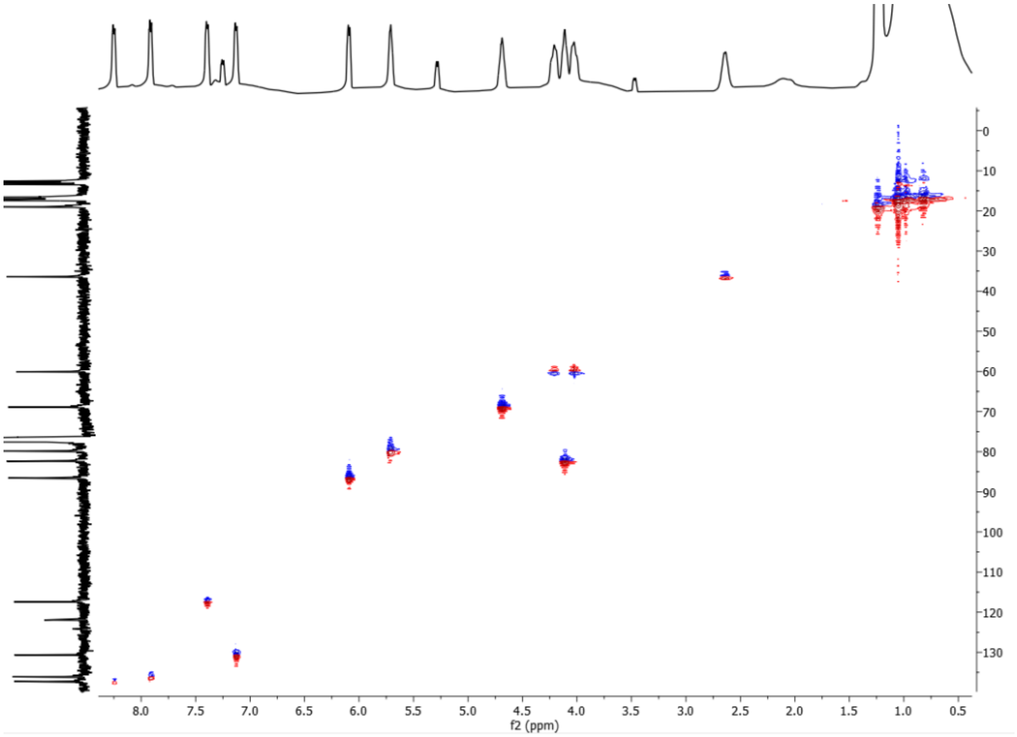

Figure S23.5: HSQC spectra of compound **3-G**.

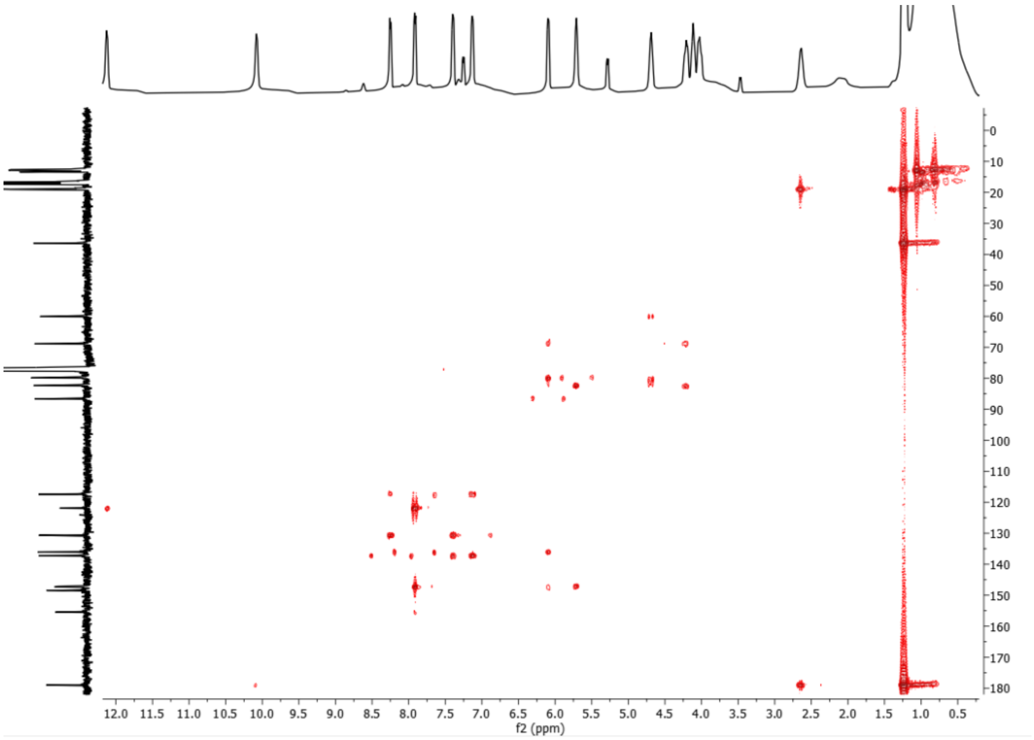

Figure S23.6: HMBC spectra of compound **3-G**.

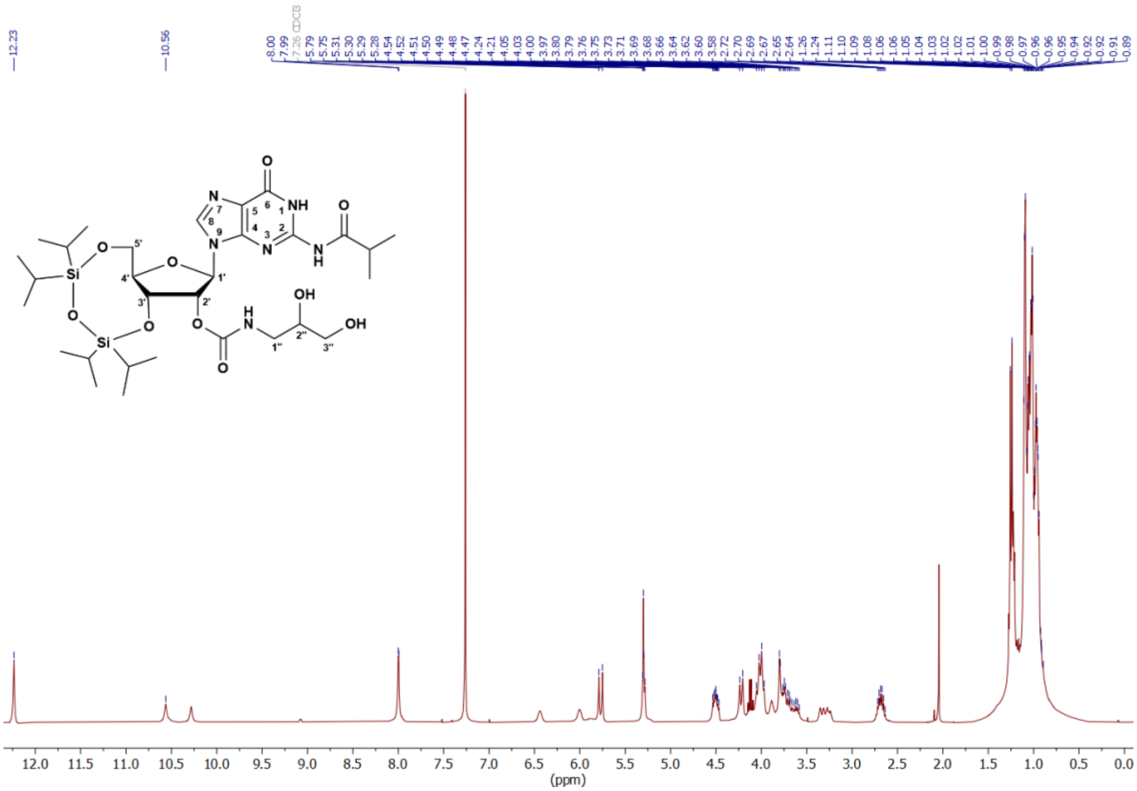

Figure S24.1: ^{1}H NMR spectrum (400 MHz, $CDCl_3$) of compound **4-G**.

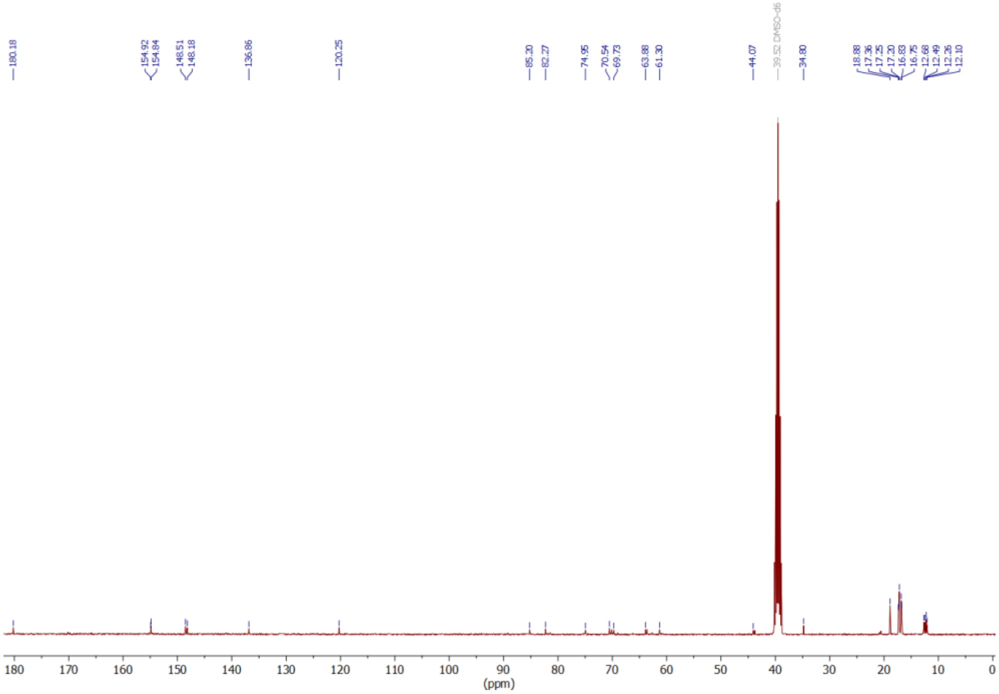

Figure S24.2: ^{13}C NMR spectrum (101 MHz, DMSO-d_6) of compound **4-G**.

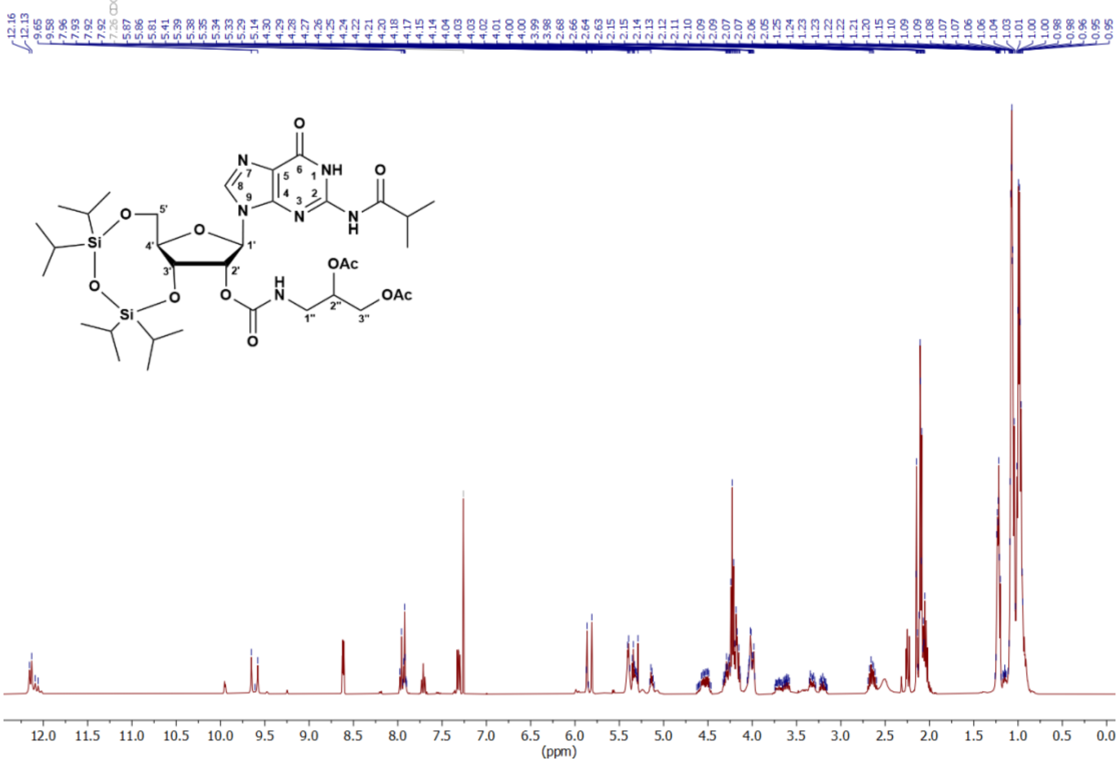

Figure S25.1: ^{1}H NMR spectrum (400 MHz, $CDCl_3$) of compound **5-G**.

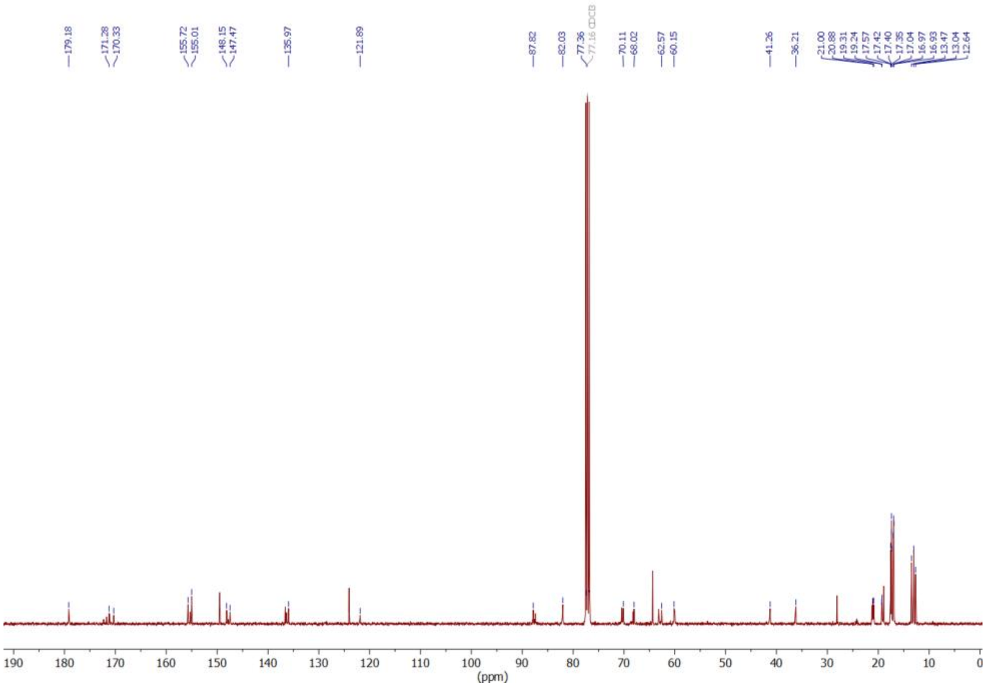

Figure S25.2: ^{13}C NMR spectrum (101 MHz, $CDCl_3$) of compound **5-G**.

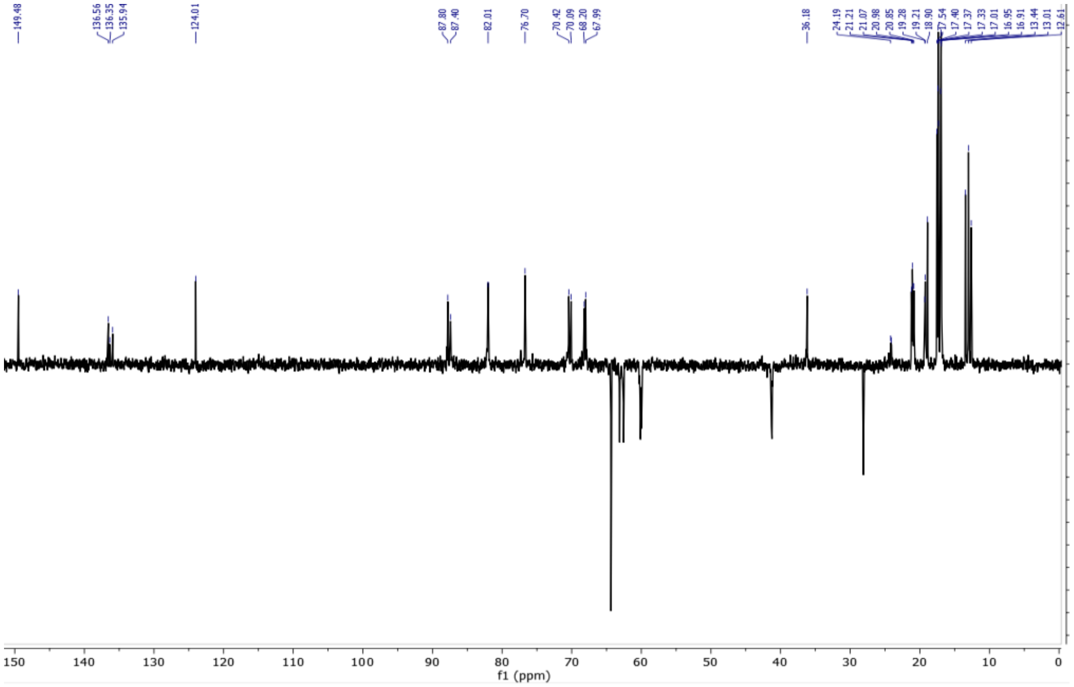

Figure S25.3: ^{13}C DEPT with decoupling NMR spectrum of compound **5-G**.

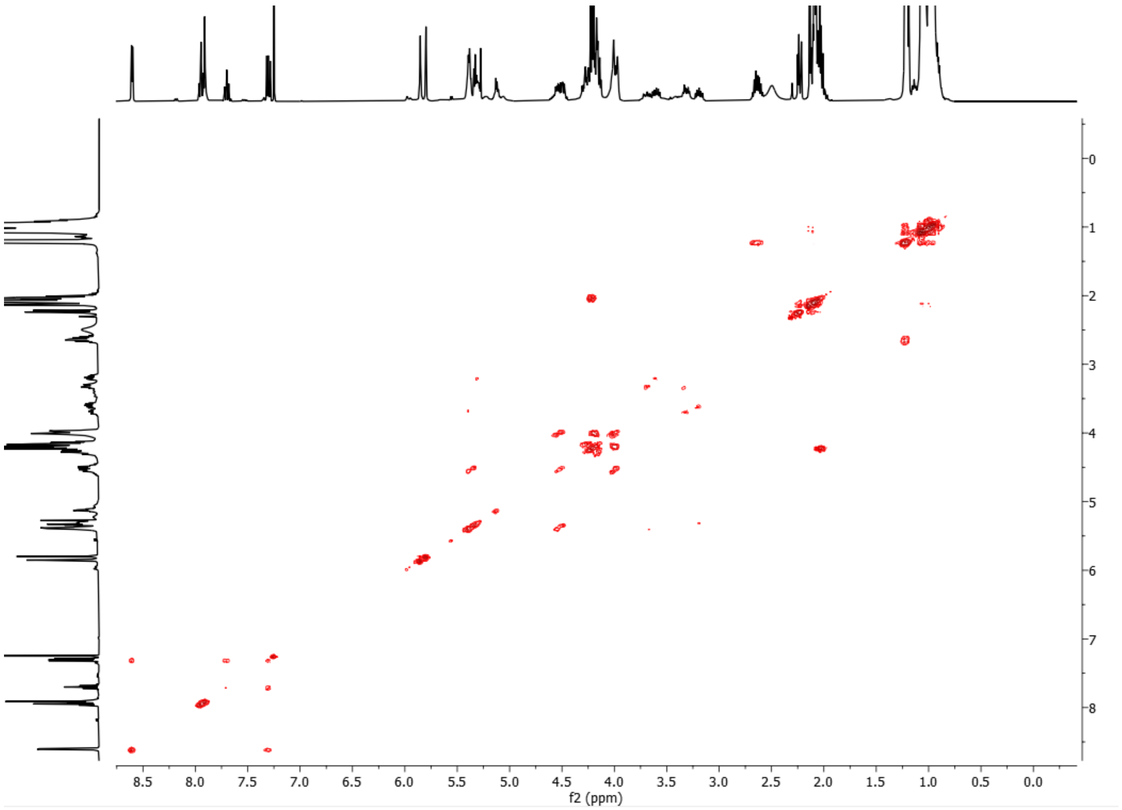

Figure S25.4: gCOSY spectra of compound **5-G**.

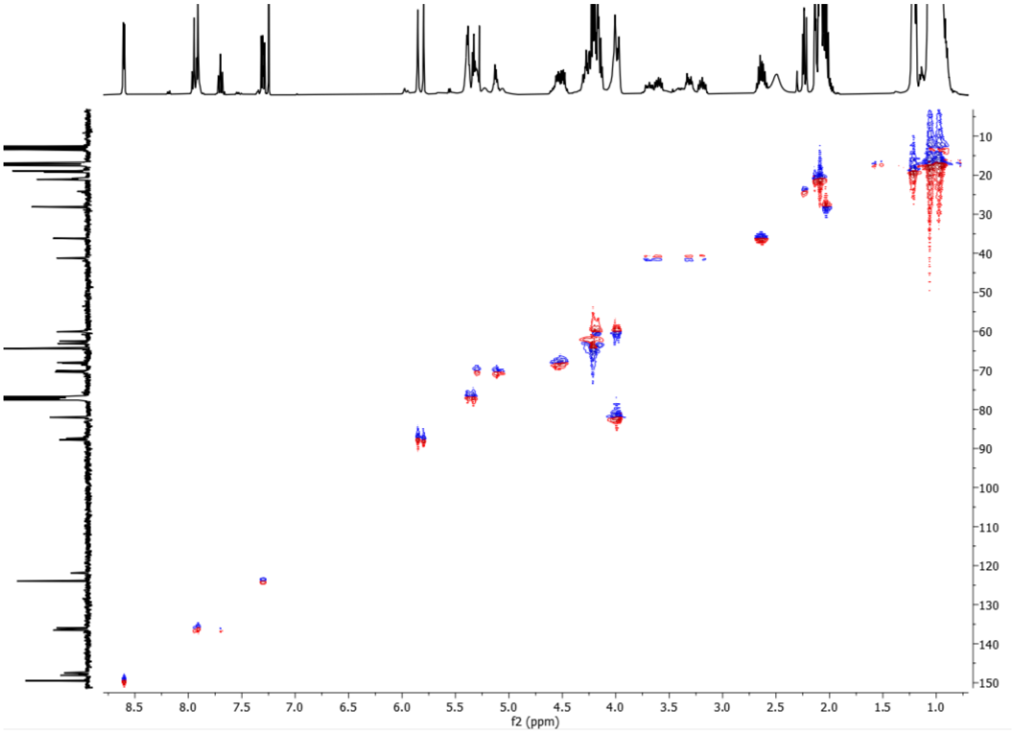

Figure S25.5: HSQC spectra of compound **5-G**.

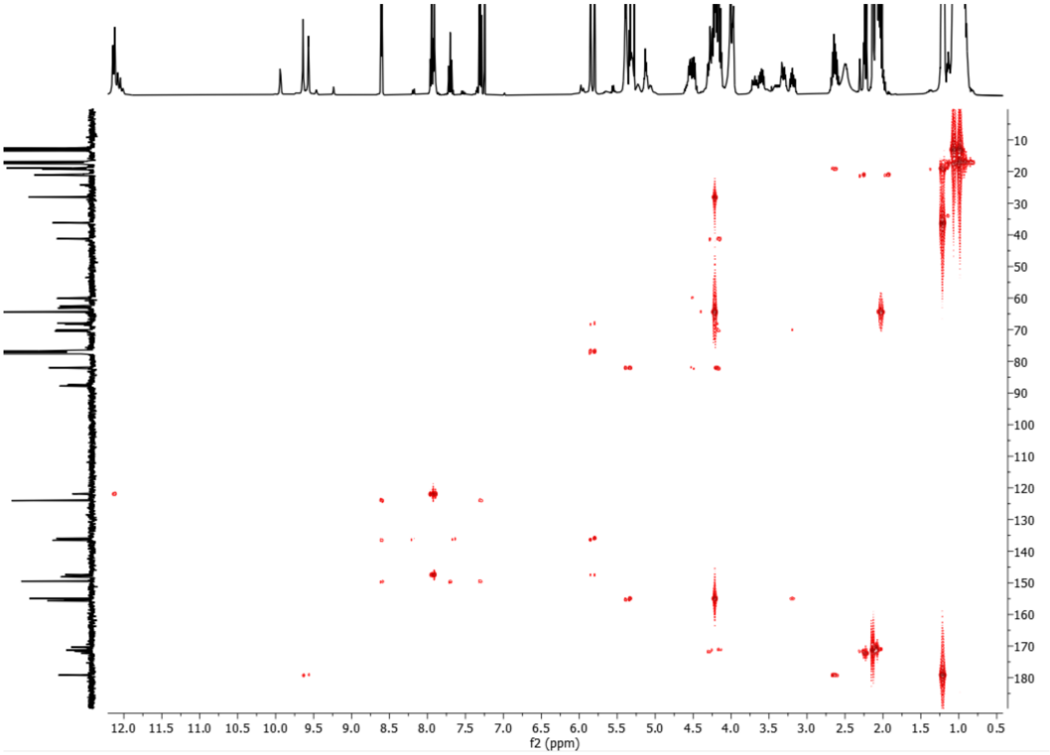

Figure S25.6: HMBC spectra of compound **5-G**.

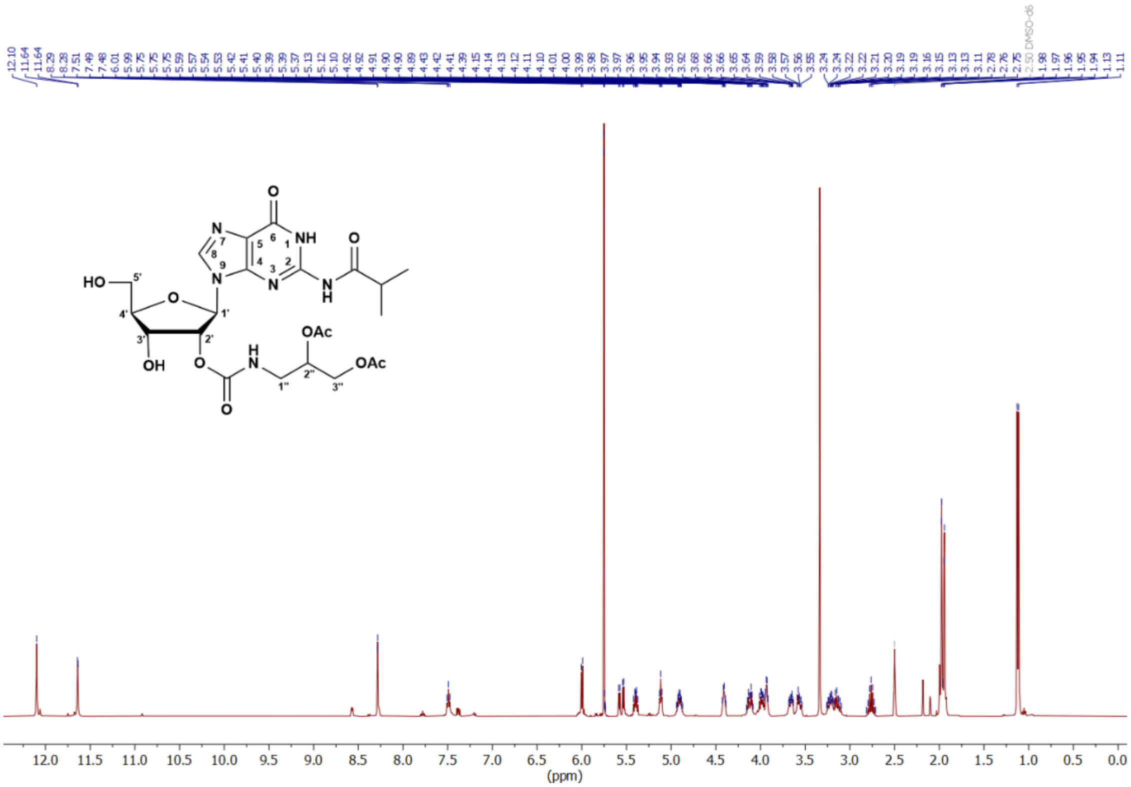

Figure S26.1: ^{1}H NMR spectrum (400 MHz, DMSO-d_6) of compound **6-G**.

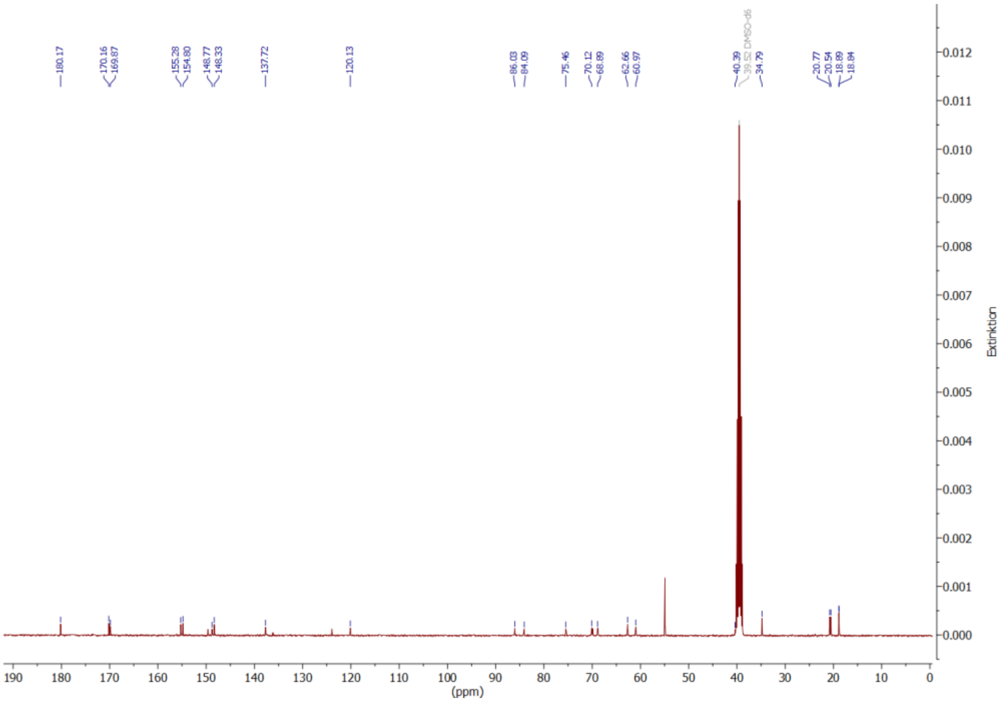

Figure S26.2: ^{13}C NMR spectrum (101 MHz, DMSO-d_6) of compound **6-G**.

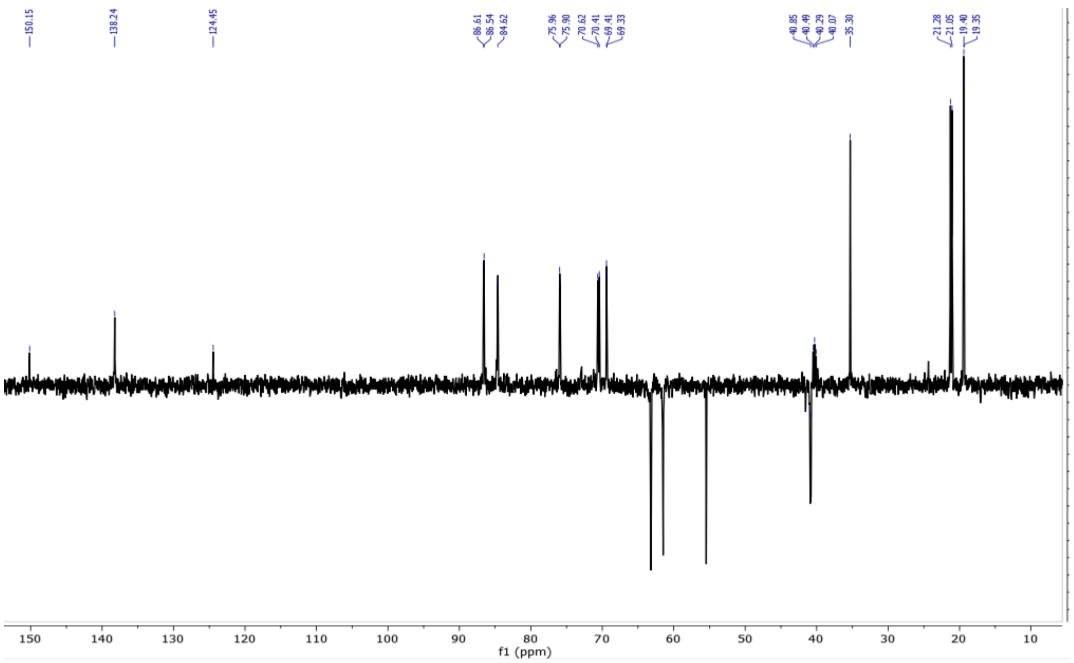

Figure S26.3: ^{13}C DEPT with decoupling NMR spectrum of compound **6-G**.

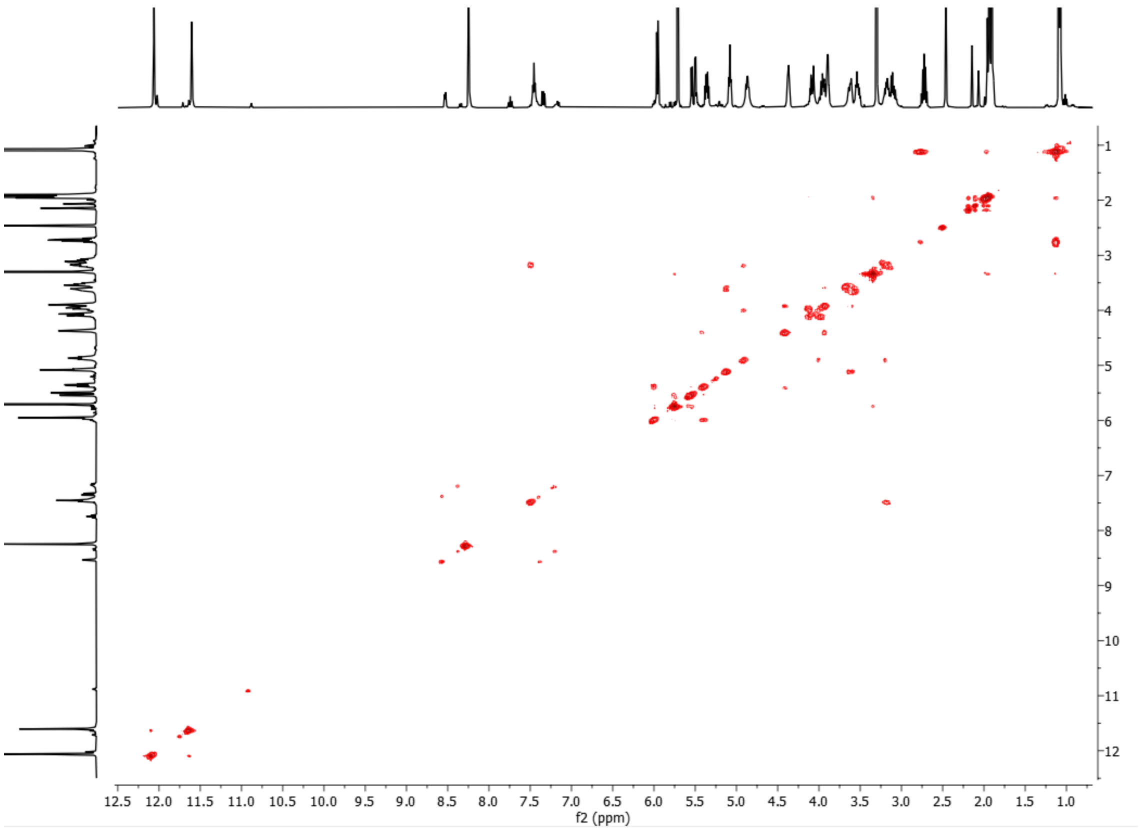

Figure S26.4: gCOSY spectra of compound **6-G**.

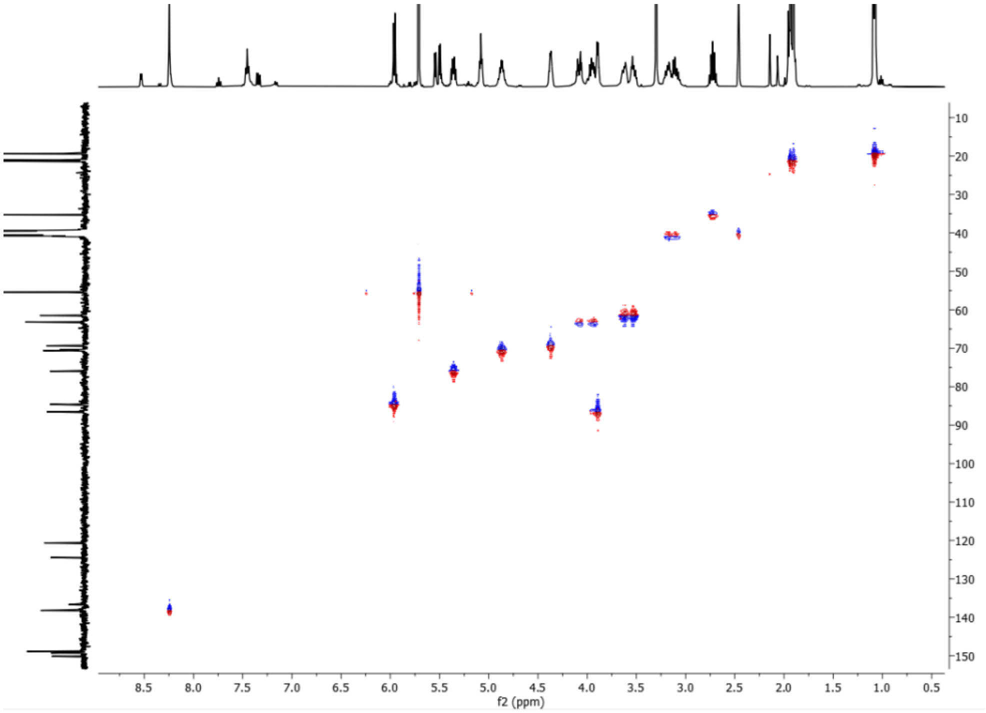

Figure S26.5: HSQC spectra of compound **6-G**.

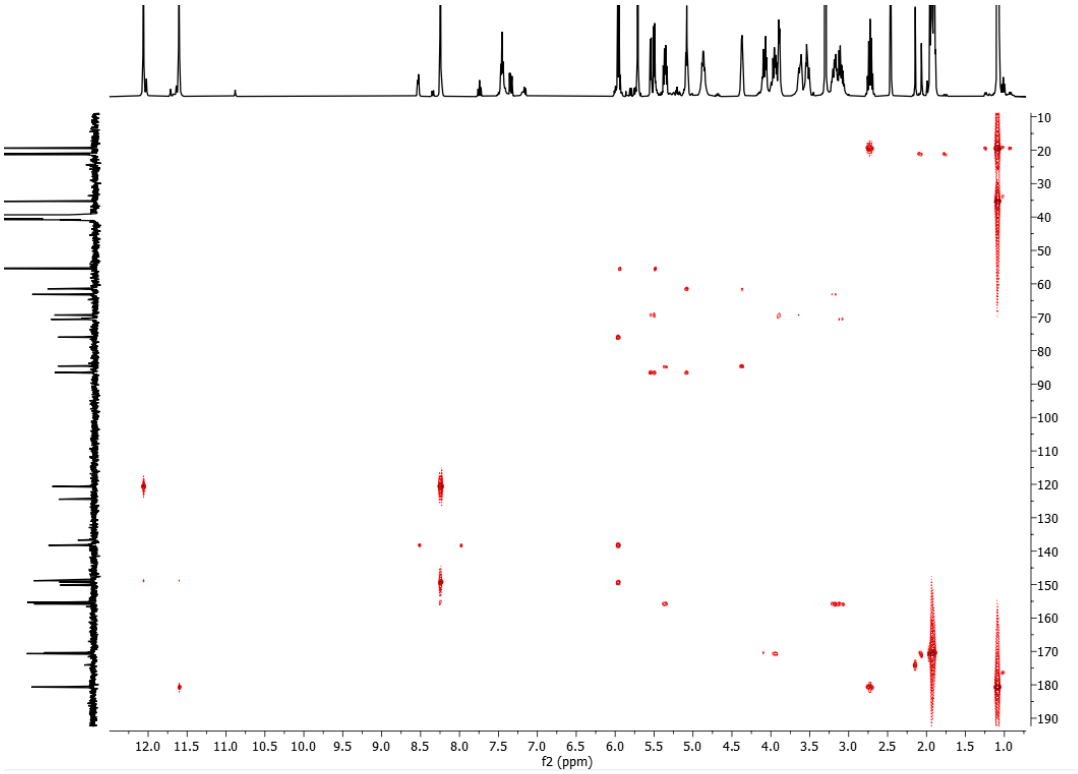

Figure S26.6: HMBC spectra of compound **6-G**.

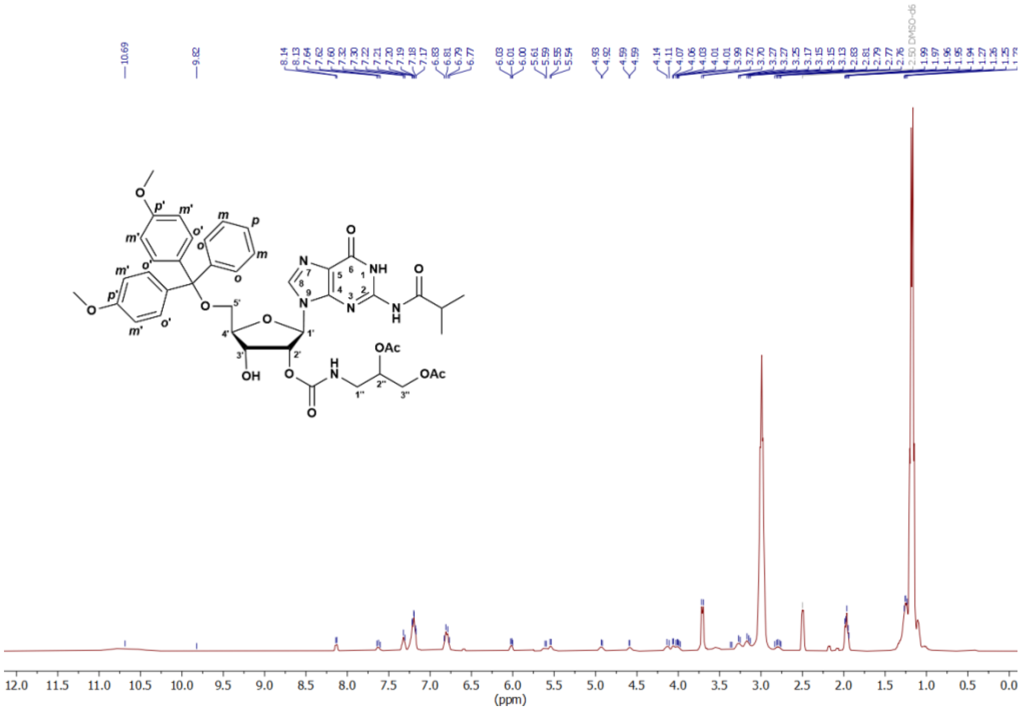

Figure S27.1: ^{1}H NMR spectrum (400 MHz, DMSO-d_6) of compound **7-G**.

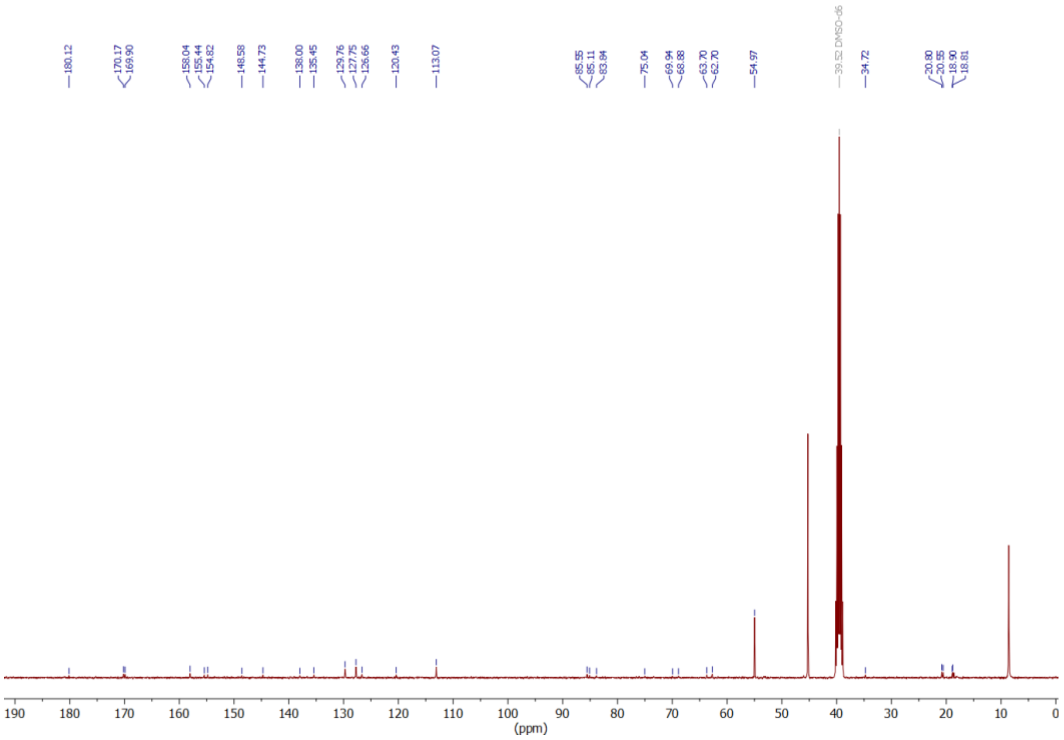

Figure S27.2: ^{13}C NMR spectrum (101 MHz, DMSO-d_6) of compound **7-G**.

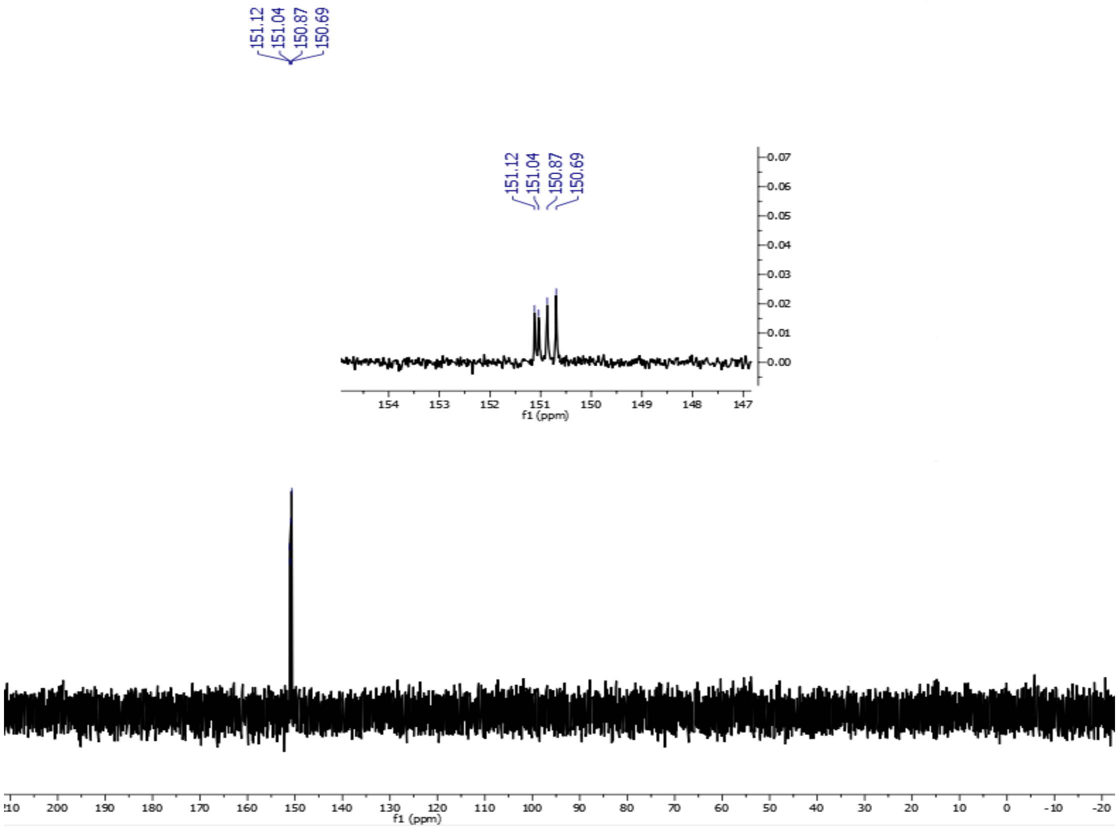

Figure S28: ^{31}P NMR spectrum (162 MHz, $CDCl_3$) of compound **8-G**.

Deconvoluted ESI-MS spectra of synthesized oligonucleotides:

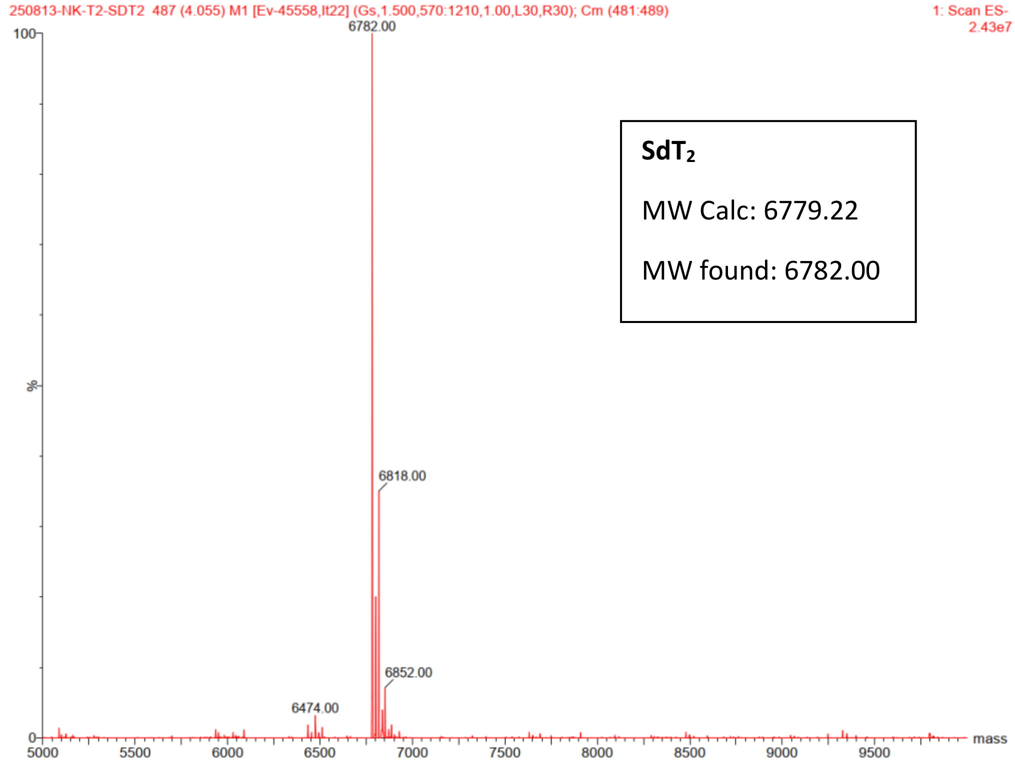

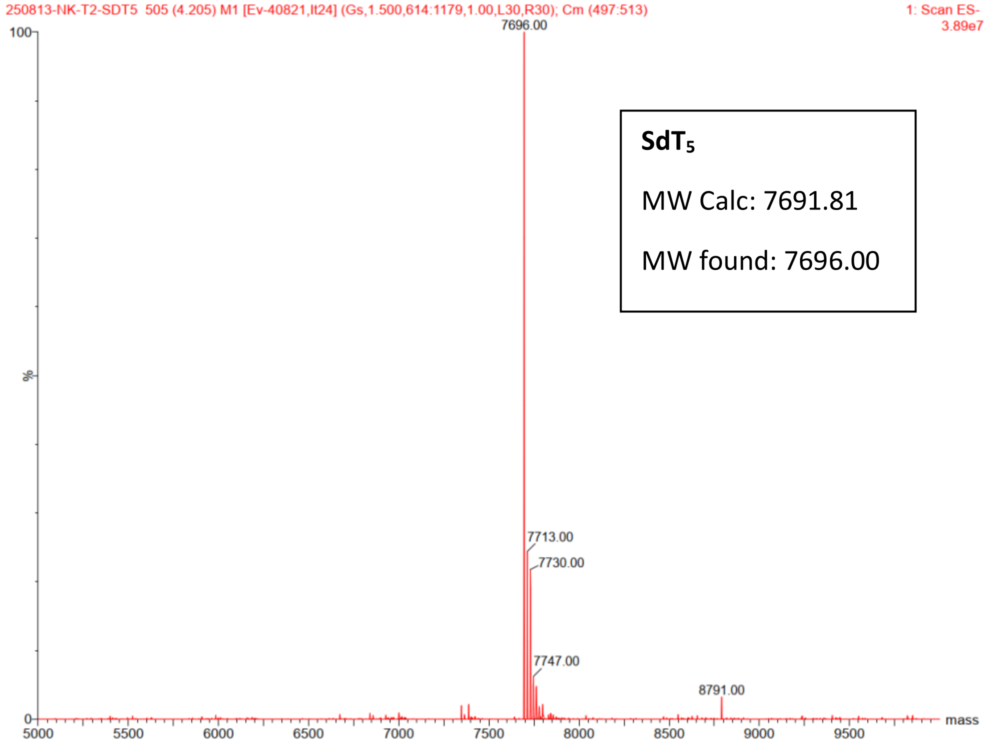

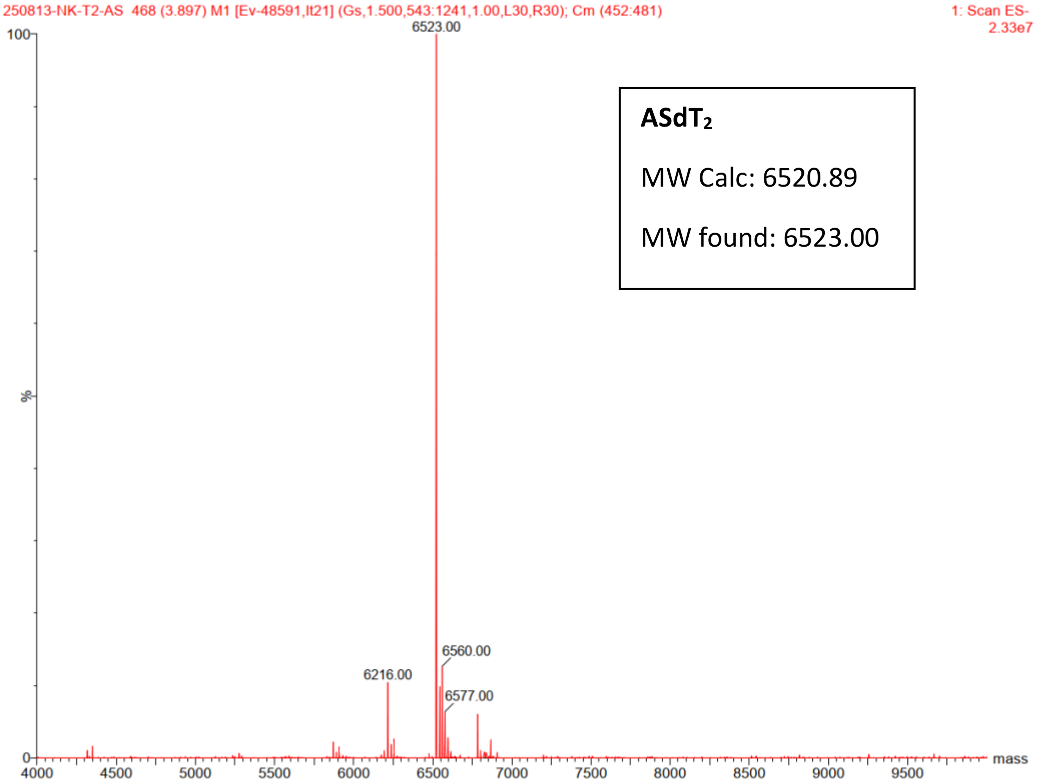
250813-NK-T2-AS 468 (3.897) M1 [Ev-48591,It21] (Gs,1.500,543:1241,1.00,L30,R30); Cm (452:481)
1: Scan ES-
2.33e7
6523.00
6560.00
6216.00
6577.00
100
%
0
mass
4000
4500
5000
5500
6000
6500
7000
7500
8000
8500
9000
9500
ASdT2
MW Calc: 6520.89
MW found: 6523.00

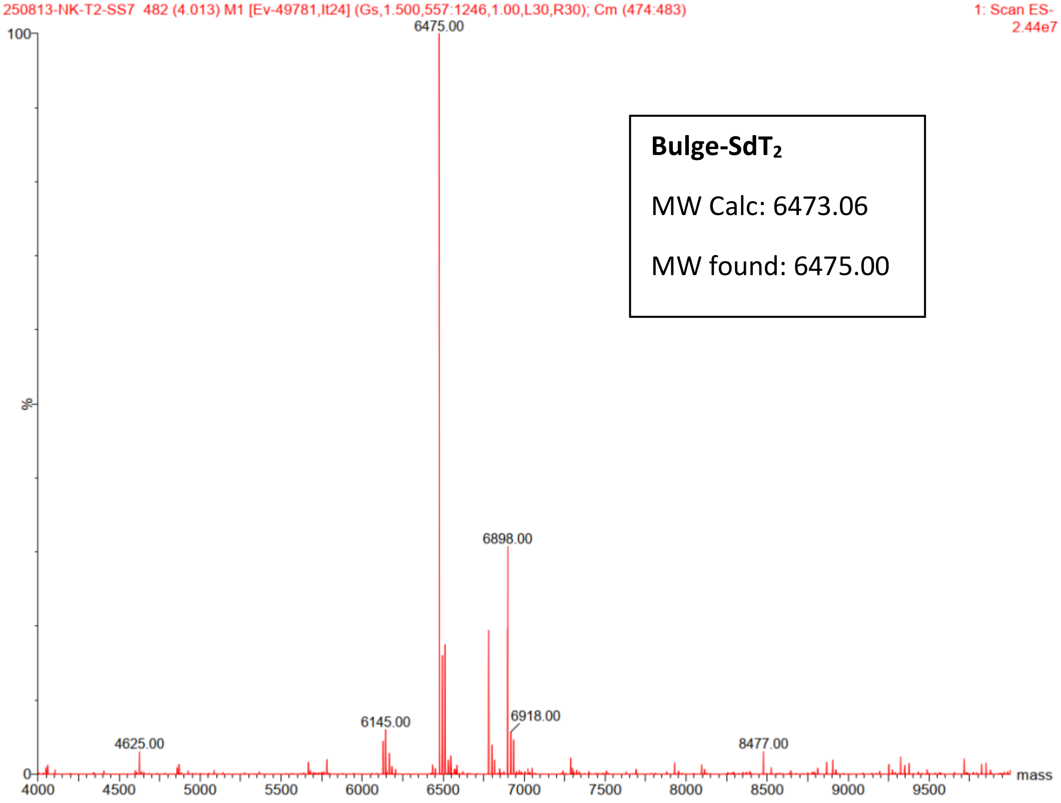
250813-NK-T2-SS7 482 (4.013) M1 [Ev-49781,It24] (Gs,1.500,557:1246,1.00,L30,R30); Cm (474:483)
1: Scan ES-
2.44e7
6475.00
6898.00
6918.00
6145.00
4625.00
8477.00
100
%
0
mass
4000
4500
5000
5500
6000
6500
7000
7500
8000
8500
9000
9500
Bulge-SdT2
MW Calc: 6473.06
MW found: 6475.00

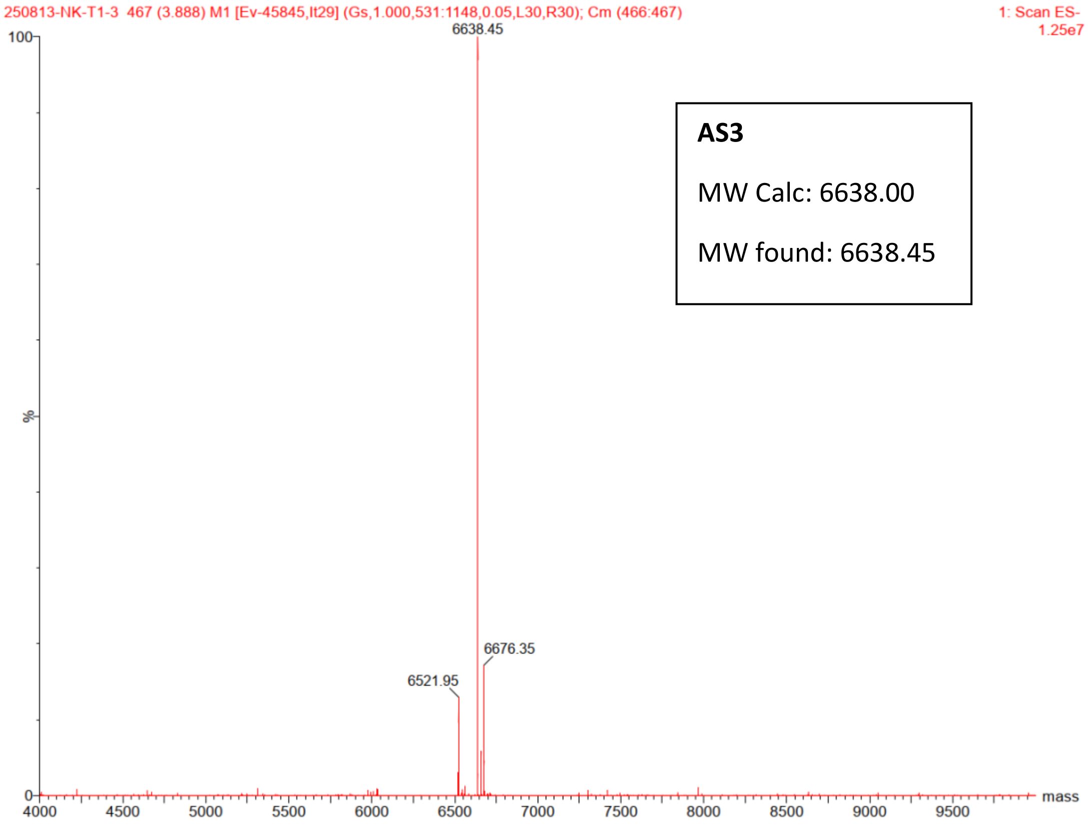
250813-NK-T1-3 467 (3.888) M1 [Ev-45845,It29] (Gs,1.000,531:1148,0.05,L30,R30); Cm (466:467)
1: Scan ES-
1.25e7
6638.45
AS3
MW Calc: 6638.00
MW found: 6638.45
6676.35
6521.95
mass

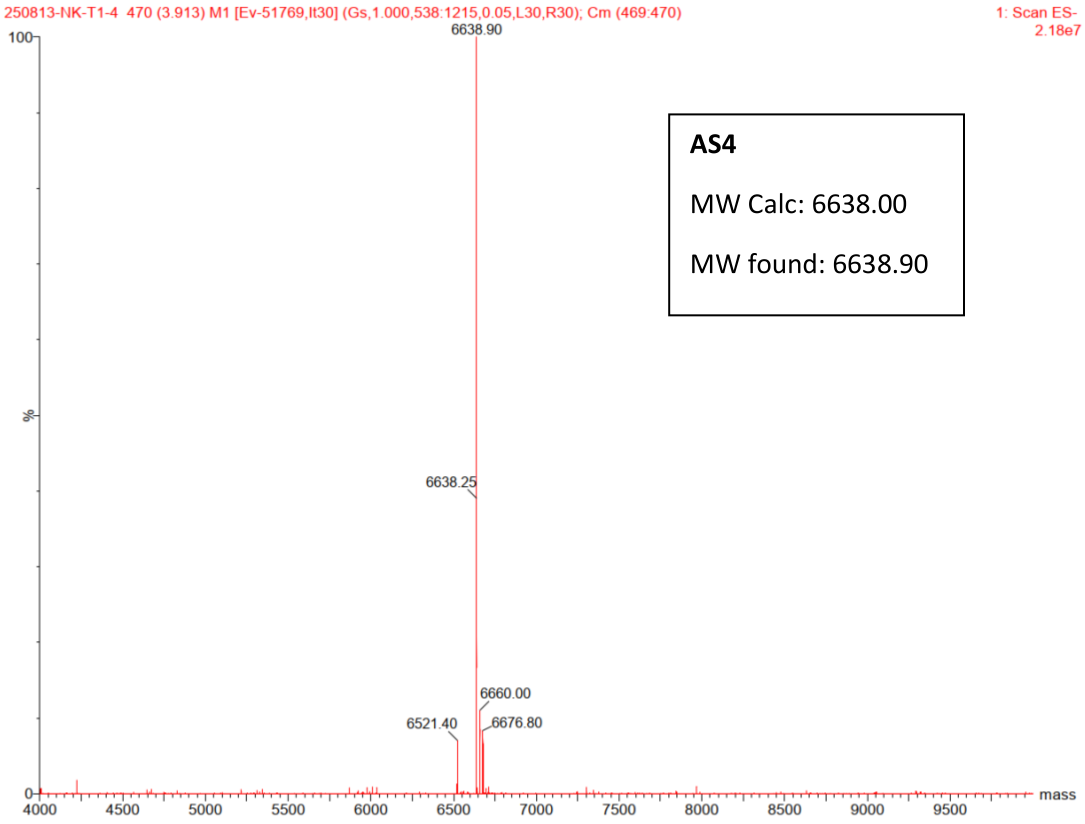
250813-NK-T1-4 470 (3.913) M1 [Ev-51769,It30] (Gs,1.000,538:1215,0.05,L30,R30); Cm (469:470)
1: Scan ES-
2.18e7
6638.90
AS4
MW Calc: 6638.00
MW found: 6638.90
6638.25
6660.00
6521.40
6676.80
mass

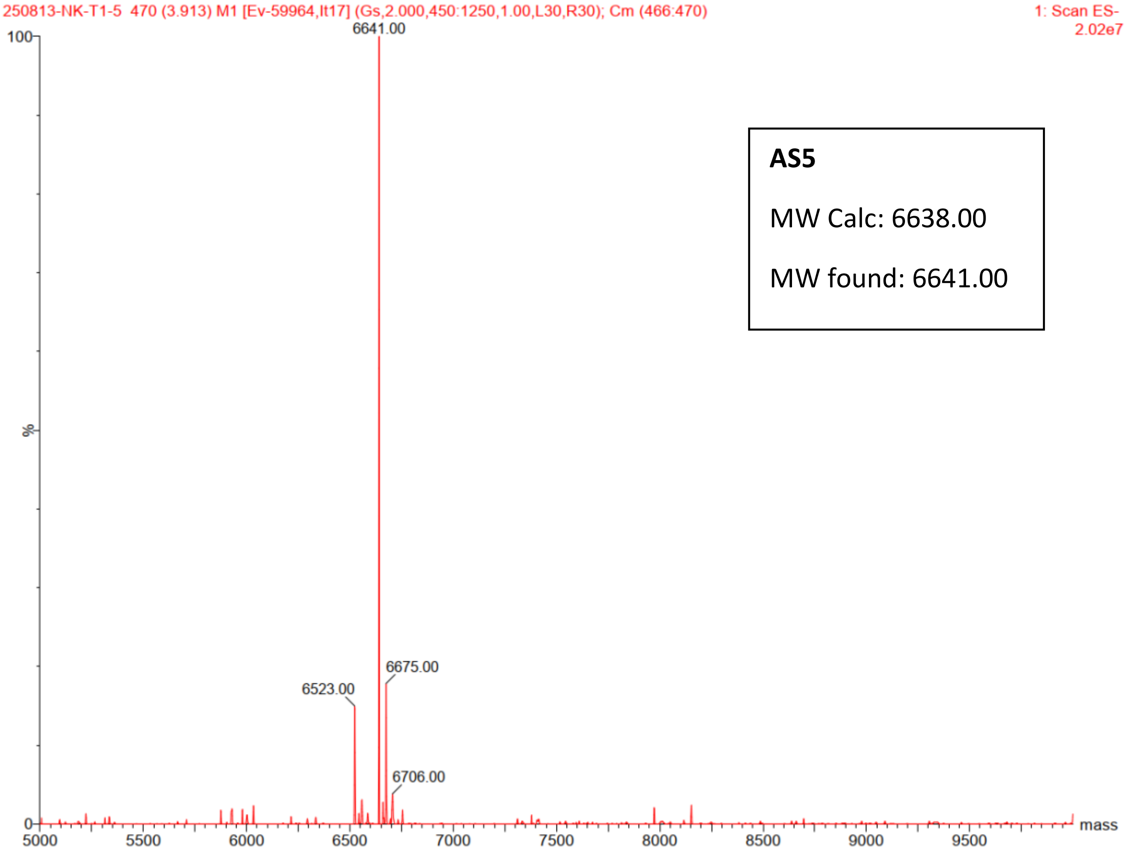

AS5

MW Calc: 6638.00

MW found: 6641.00

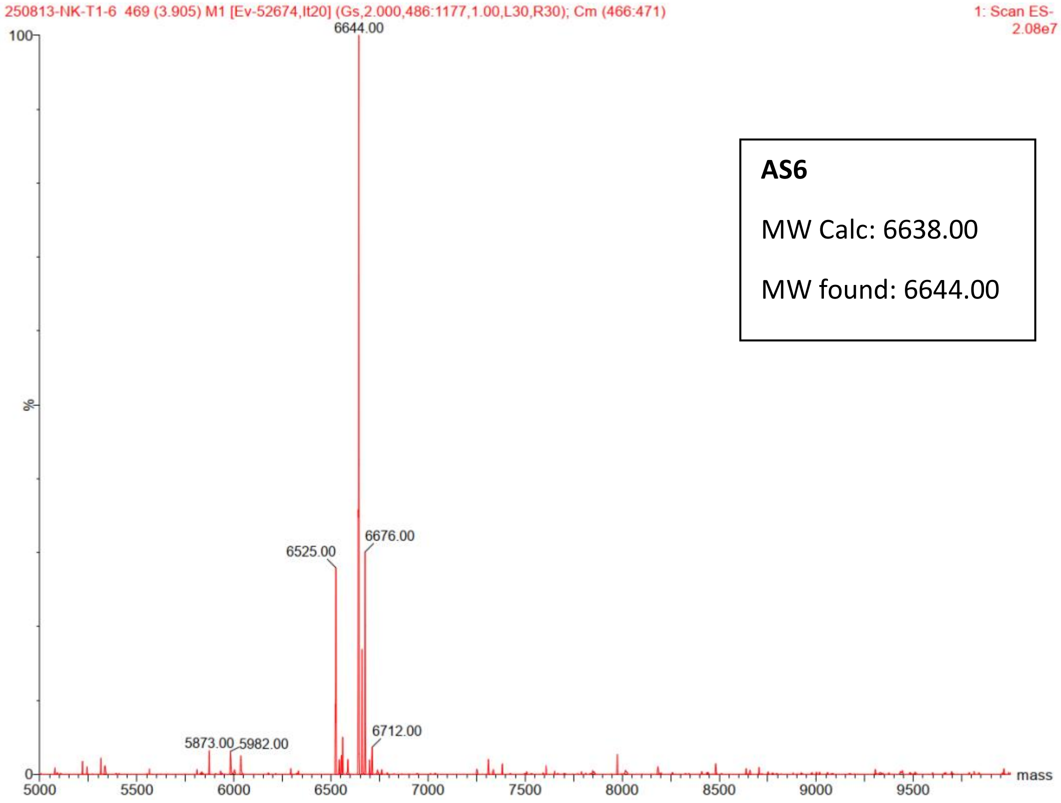

AS6

MW Calc: 6638.00

MW found: 6644.00

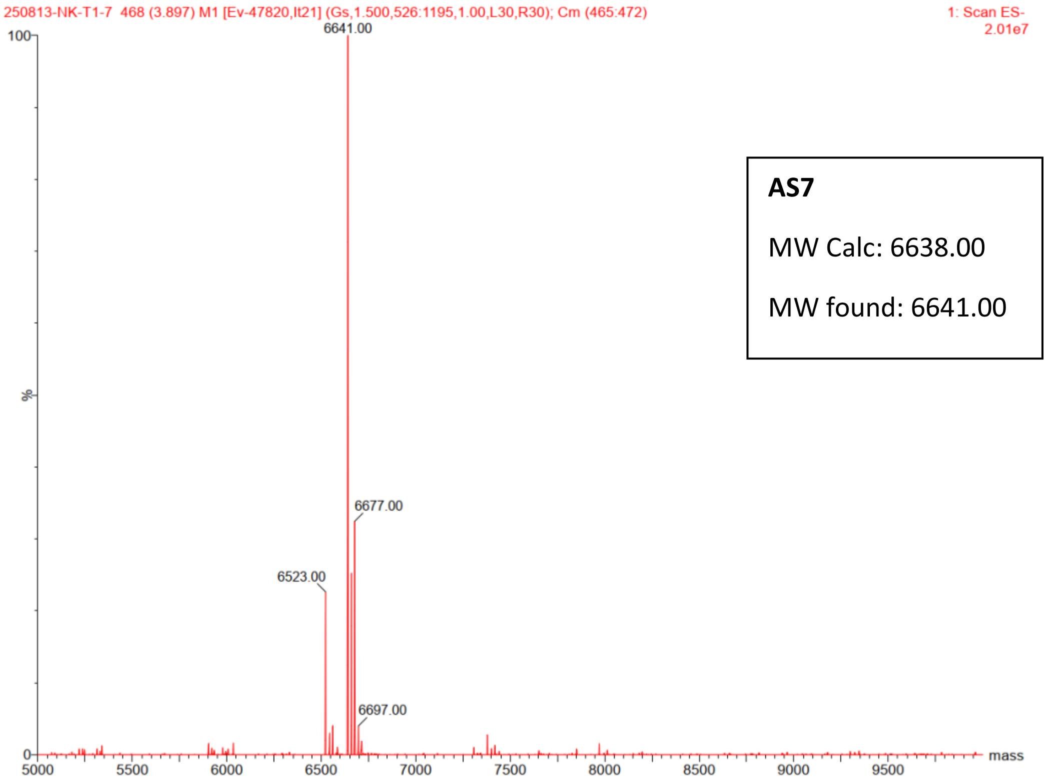

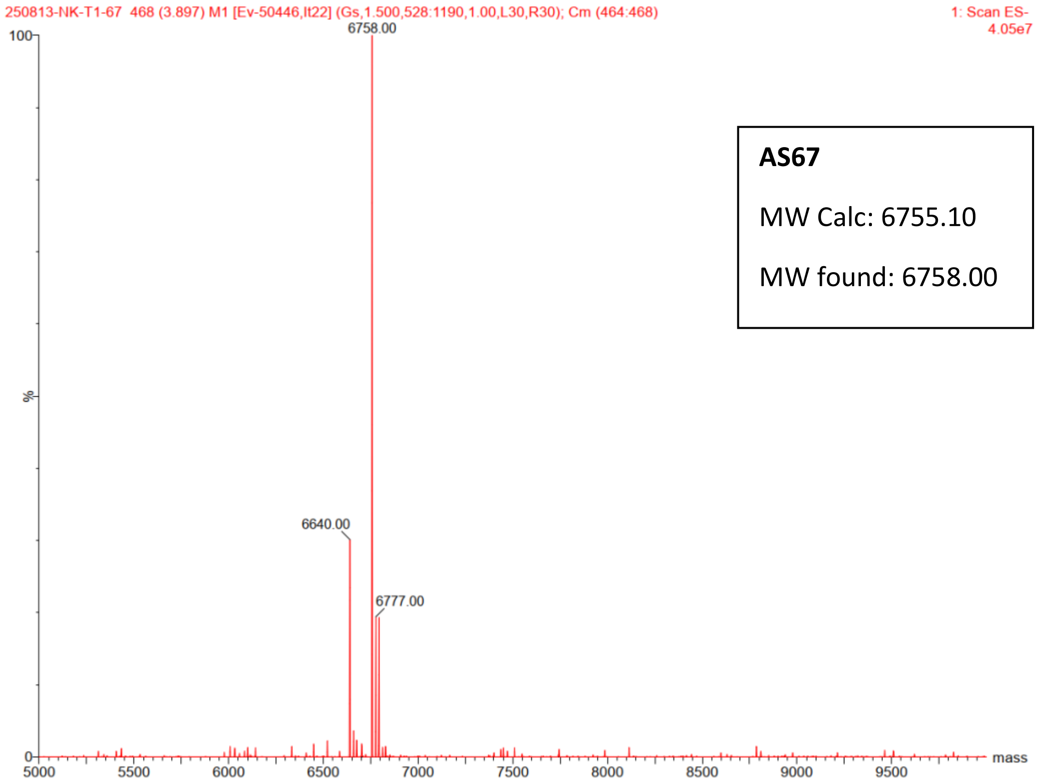

Figure S29: ESI-MS deconvoluted spectra of synthesized oligonucleotides.

HPLC analysis of modified chemically modified Oligonucleotides:

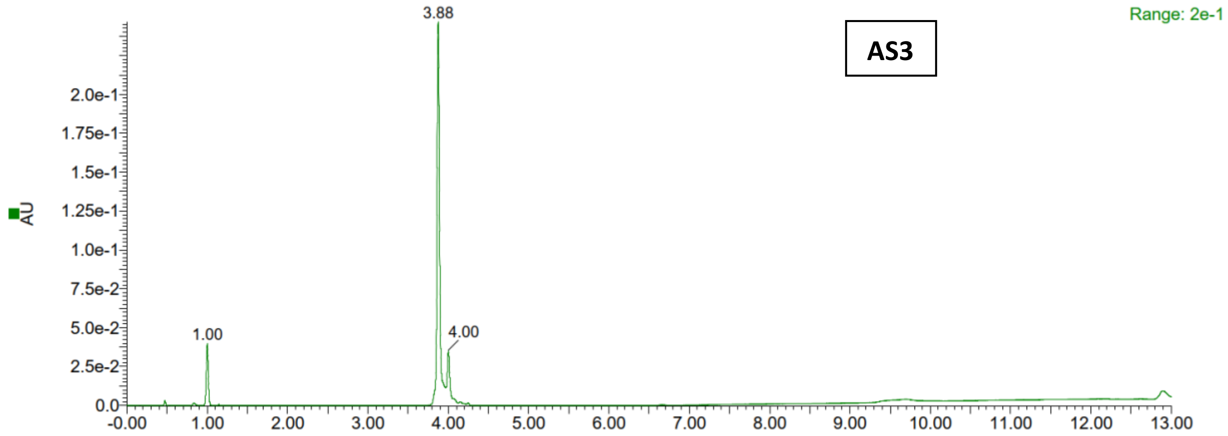

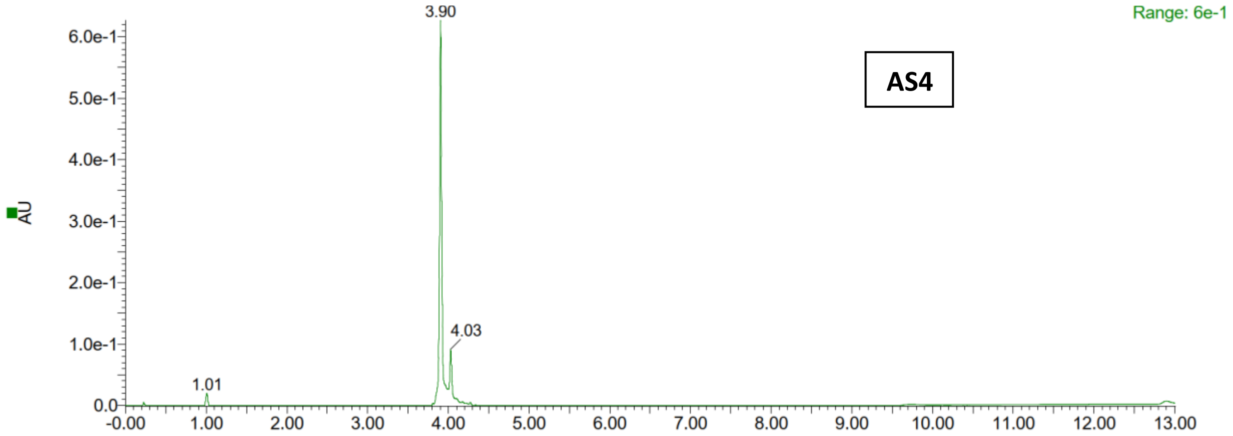

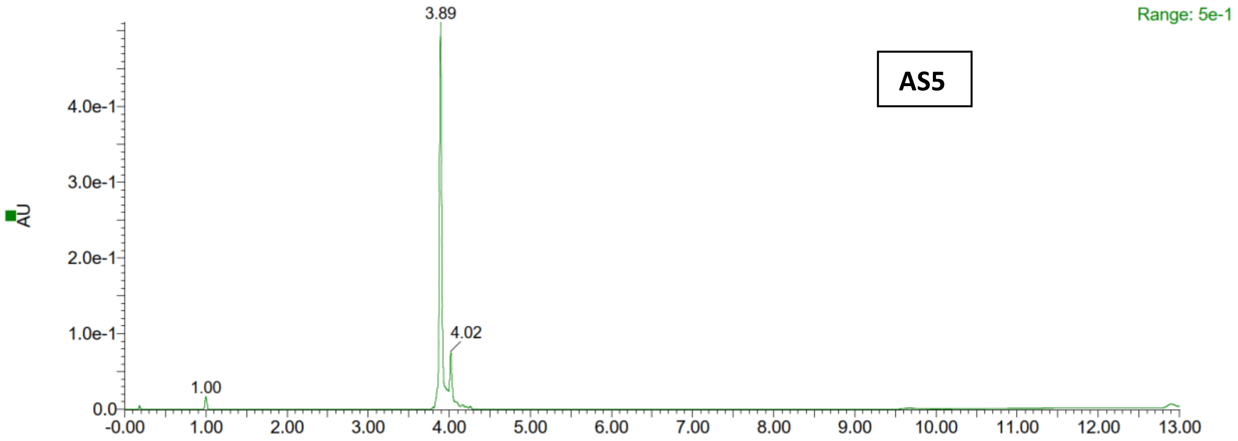

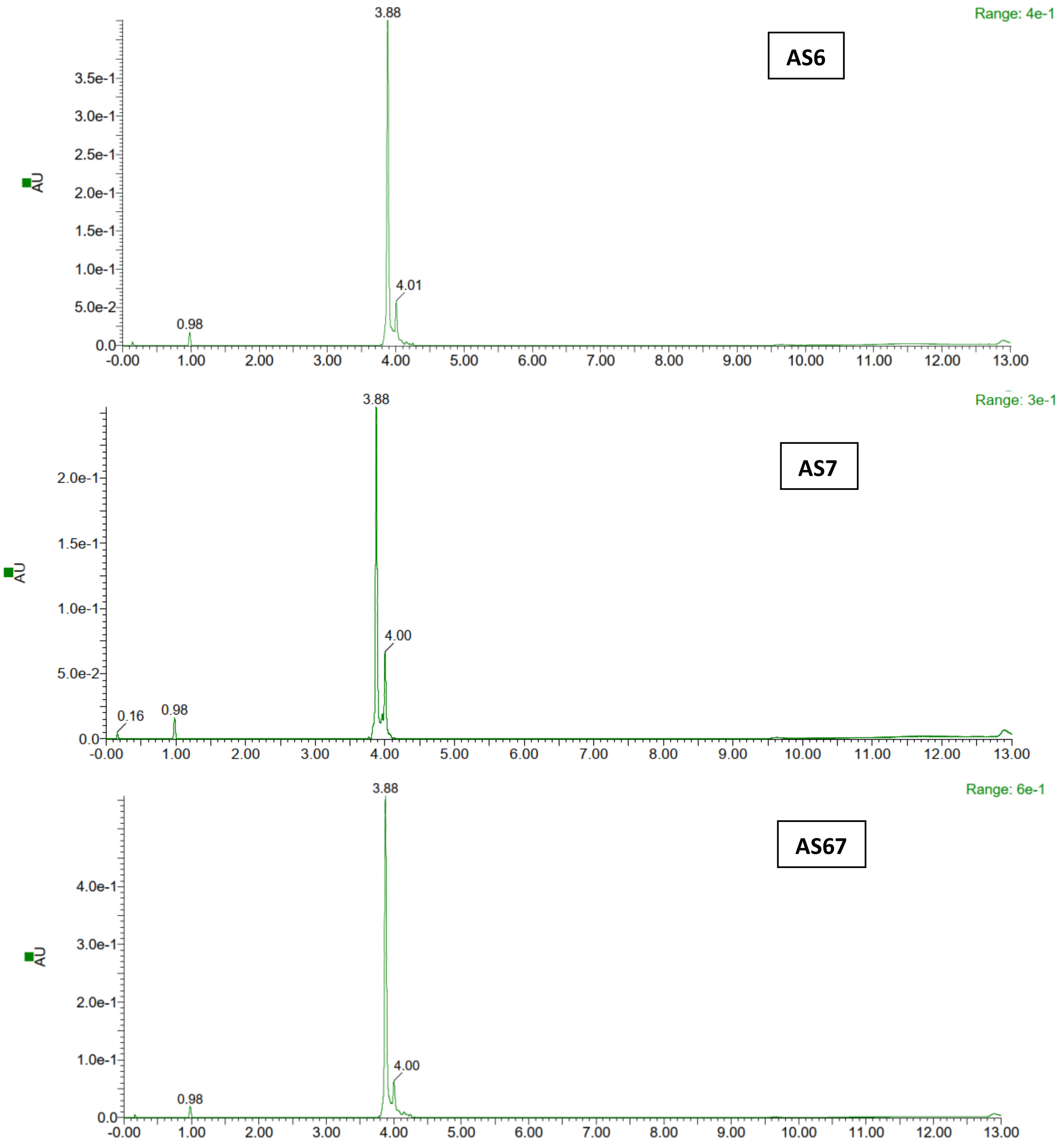

Figure S30: HPLC traces of chemically modified oligonucleotides.

Mass analysis of synthesized compounds (small molecules):

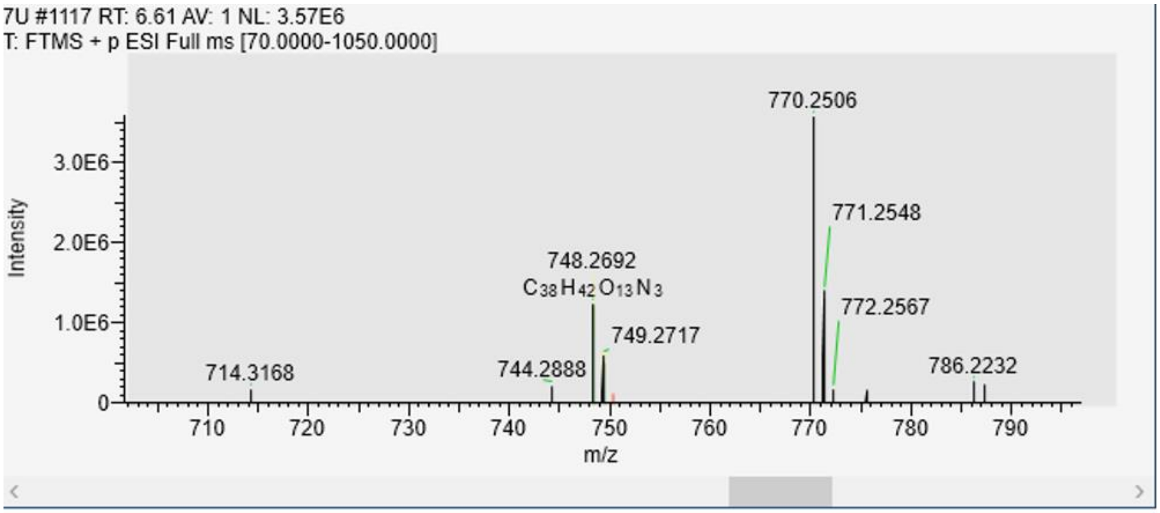

Elemental Composition Results - Spectrum 1

Pe...	Display Formula	S Fit	RDB	Delta [ppm]	Theo. mass	Rank	Combin...	# Matched Is
748.2692	$C_{38}H_{42}O_{13}N_3$	28.28	19.50	-2.74	748.27121	1	96.23	[illegible]

Figure S31.1: HRMS analysis of compound **7U (with [M+H] and [M+Na] peaks)**.

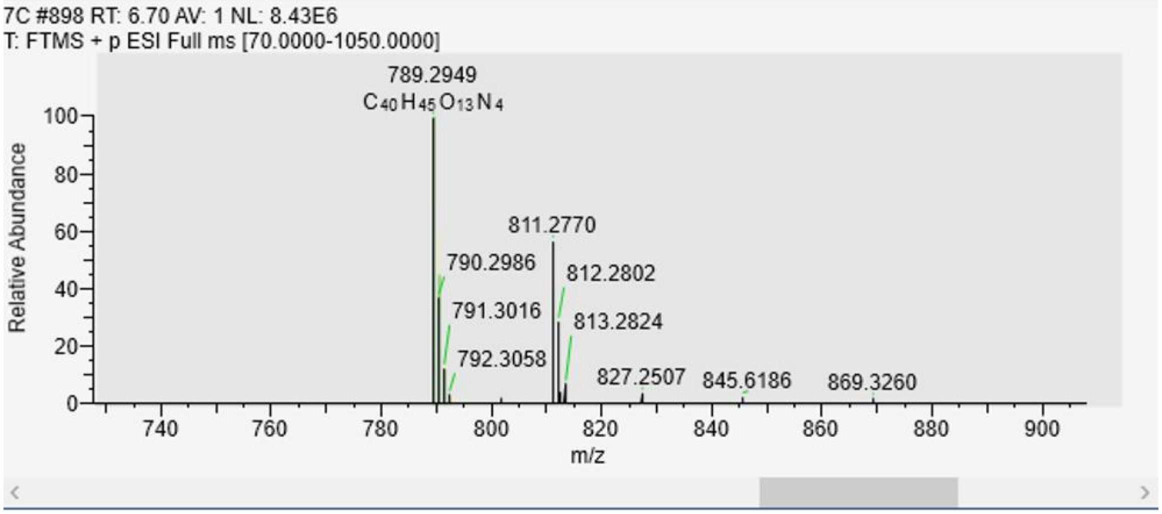

Elemental Composition Results - Spectrum 1

Pe...	Display Formula	S Fit	RDB	Delta [ppm]	Theo. mass	Rank	Combin...	# Matche
789.2949	$C_{40}H_{45}O_{13}N_4$	33.11	20.50	-3.68	789.29776	1	94.52	

Figure S31.2: HRMS analysis of compound **7C (with [M+H] and [M+Na] peaks)**

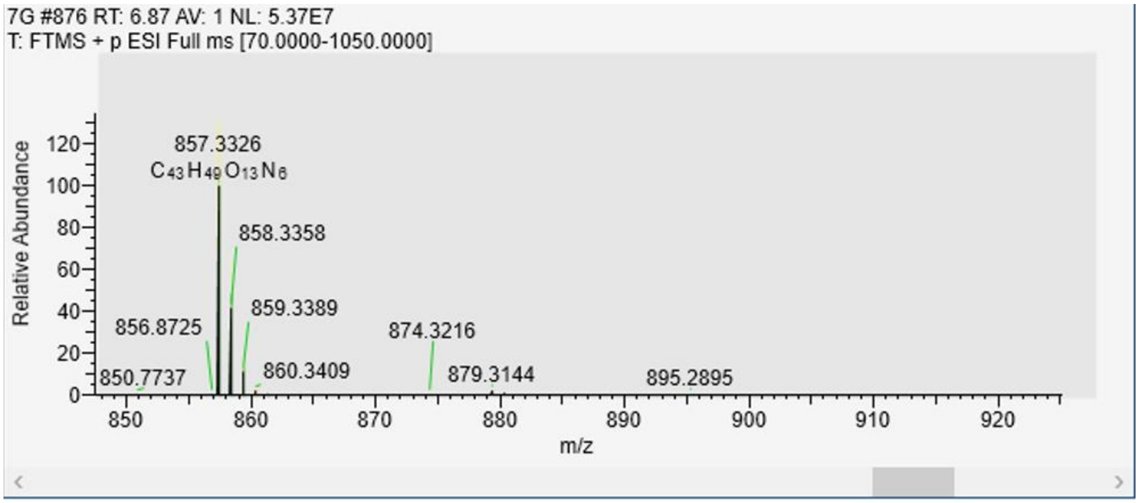

Elemental Composition Results - Spectrum 1

Pe...	Display Formula	S Fit	RDB	Delta [ppm]	Theo. mass	Rank	Combin...	# Matc
857.3326	$C_{43}H_{49}O_{13}N_6$	37.42	22.50	-3.00	857.33521	1	95.34	

Figure S31.3: HRMS analysis of compound **7G (with [M+H] peak).**

In Vitro analysis:

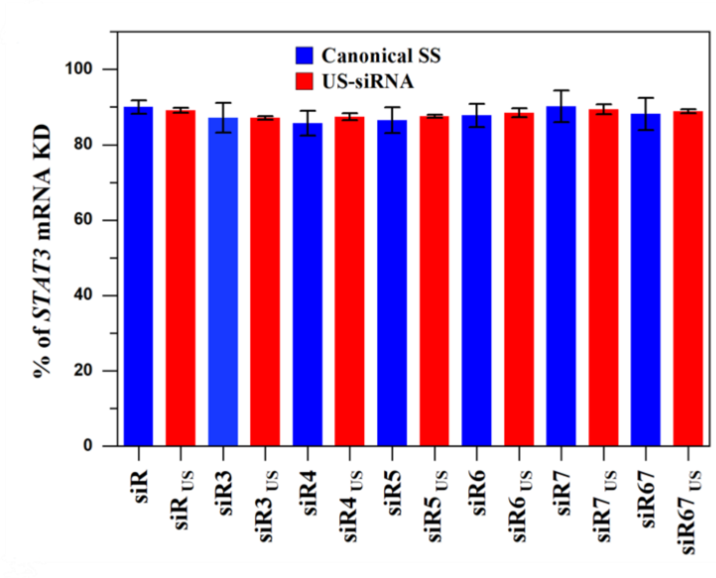

Figure S32: In vitro gene knockdown analysis of chemically and structurally modified siRNA's at 1 nM.

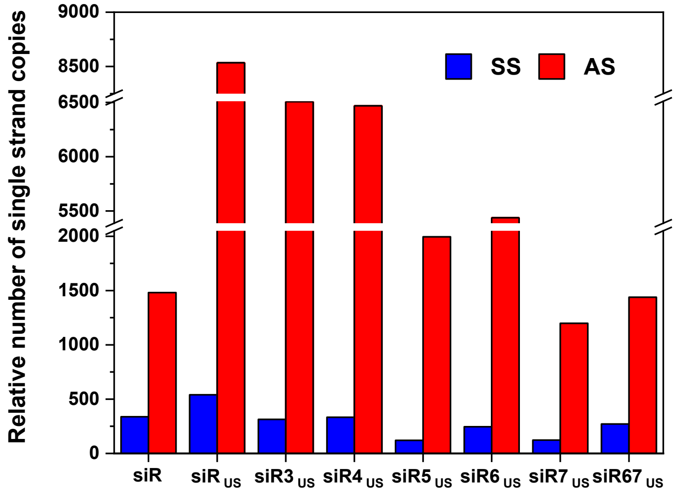

Figure S33: Relative number of single strand copies (Sense Strand (SS) and Antisense Strand (AS)) with respect to internal control in stem loop RT-qPCR experiment.

Thermal analysis - UV plots and Melting curves:

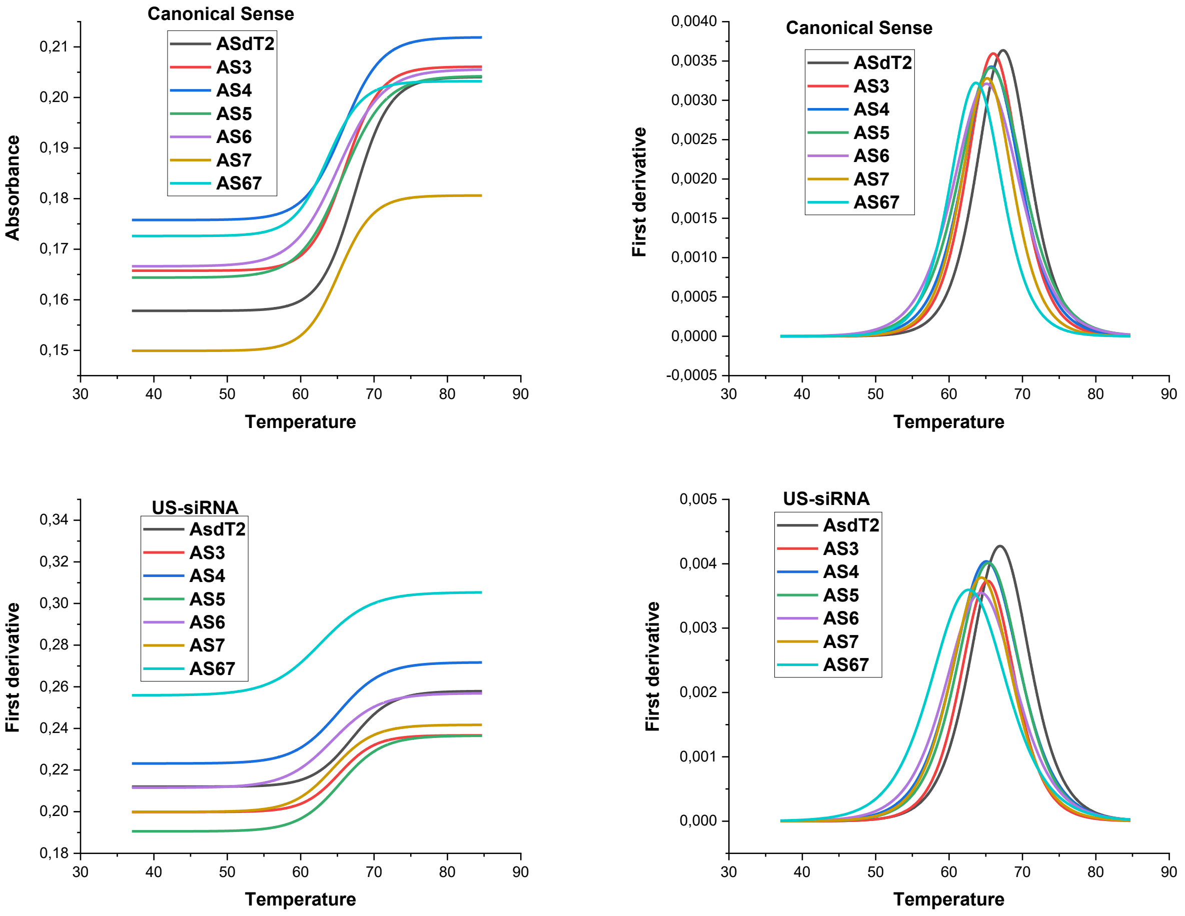

Figure S34.1: UV absorbance plotted against temperature (left) and thermal melting curve (right) of chemically and structurally modified siRNA duplexes.

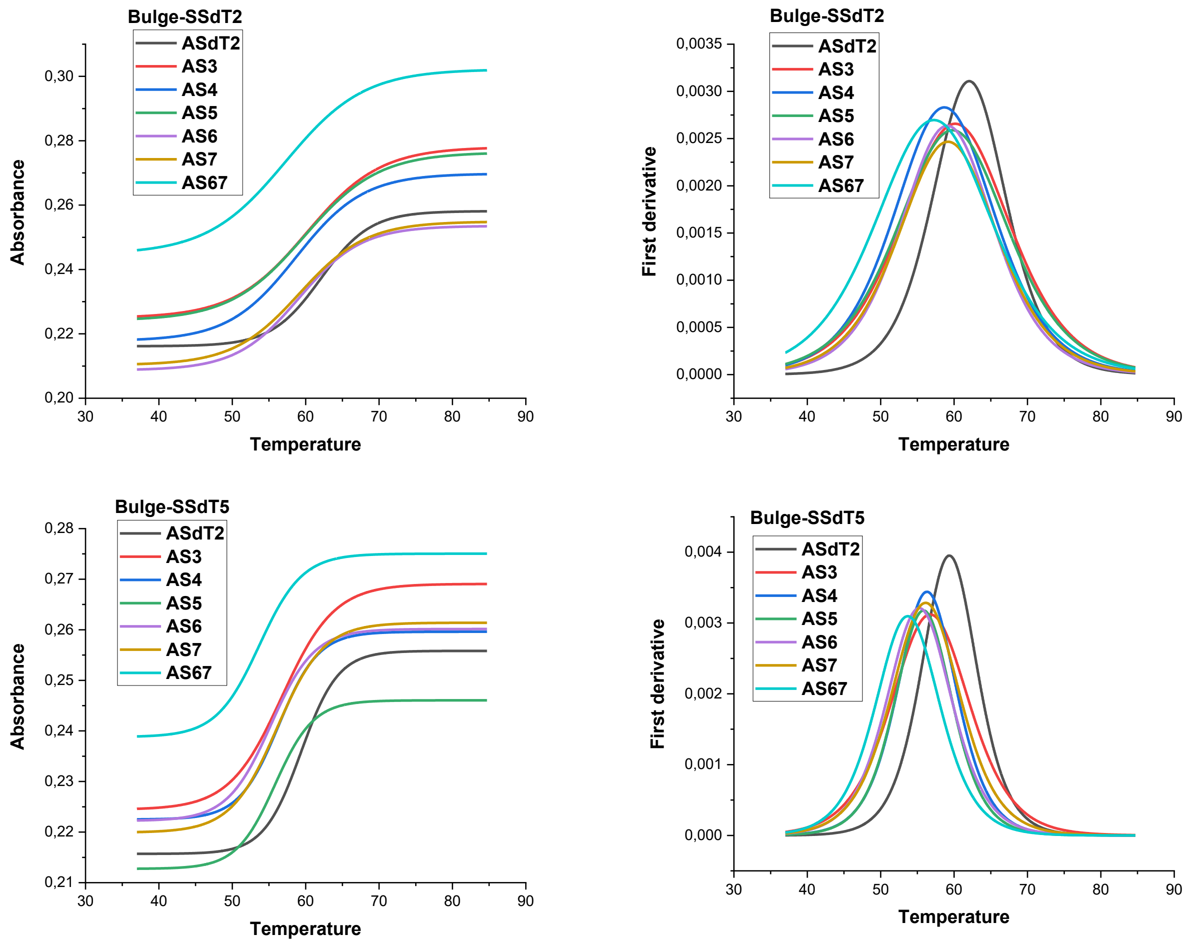

Figure S34.2: UV absorbance plotted against temperature (left) and thermal melting curve (right) of chemically and structurally modified bulged-siRNA duplexes.
